# Use of Mukbang in Health Promotion: Scoping Review

**DOI:** 10.2196/56147

**Published:** 2025-03-27

**Authors:** Xiao Wang, Yuxue Xiao, Sujin Nam, Ting Zhong, Dongyan Tang, William Ho Cheung Li, Peige Song, Wei Xia

**Affiliations:** 1 School of Nursing Sun Yat-Sen University Guangzhou China; 2 Peking University Sixth Hospital Beijing China; 3 College of Nursing Yonsei University Seoul Republic of Korea; 4 Sun Yat-sen Memorial Hospital Sun Yat-Sen University Guangzhou China; 5 Nethersole School of Nursing The Chinese University of Hong Kong Hong Kong China; 6 School of Public Health Zhejiang University School of Medicine Hangzhou China

**Keywords:** mukbang, health promotion, eating behaviors, appetite, scoping review

## Abstract

**Background:**

Mukbang is a recent internet phenomenon in which anchors publicly record and show their eating through short video platforms. Researchers reported a tangible impact of mukbang on the psychological and physical health, appetite, and eating behavior of the public, it is critical to obtain clear and comprehensive insights concerning the use of mukbang to promote the viewers’ appetite, eating behaviors, and health to identify directions for future work.

**Objective:**

This scoping review aims to comprehensively outline the current evidence regarding the impact of mukbang consumption on dietary behaviors, appetite regulation, flavor perception, and physical and psychological well-being. Specifically, we conducted an analysis of public perceptions and attitudes toward mukbang while summarizing the reciprocal influence it has on health promotion.

**Methods:**

This study was conducted as a scoping review following the Joanna Briggs Institute guideline and the PRISMA-ScR (Preferred Reporting Items for Systematic Reviews and Meta-Analyses extension for Scoping Reviews) checklist. We comprehensively searched 8 electronic databases in Chinese, English, and Korean languages. We also searched gray literature sources like Google Scholar and ProQuest. We used a data extraction chart to extract information relevant to the impact of mukbang on health. The extracted data were qualitatively analyzed to form different themes related to health, categorizing and integrating the results based on the type of study (qualitative, observational, and experimental).

**Results:**

This scoping review finally included 53 studies; the annual distribution exhibited a consistent upward trend across all categories since their initial publication in 2017. Based on the results of the analysis, we have summarized 4 themes, which showed that mukbang may have positive effects on viewers’ appetite, food choices, and weight control; it can also meet the psychological needs of viewers and provide digital companionship and happiness. However, excessive viewing may also be harmful to viewer’s health, which has also caused health concerns for some viewers.

**Conclusions:**

This study conducted a comprehensive search, screening, and synthesis of existing studies focusing on mukbang and health across various languages and varying levels of quality, which has presented the analytical evidence of the relationship between mukbang and dietary behaviors, appetite, flavor perception, and health. According to the results, future research could consider analyzing the beneficial and harmful factors of mukbang, thereby further optimizing the existing mukbang videos accordingly to explore the potential of using mukbang for health intervention or promotion, so as to improve or customize the content of mukbang based on this scoping review, maximize the appetite and health promotion effects of mukbang videos.

**Trial Registration:**

INPLASY INPLASY2022120109; https://inplasy.com/inplasy-2022-12-0109/

## Introduction

### Background

Mukbang (or meokbang), a portmanteau of the Korean words “eat” (*meokneun*) and “broadcast” (*bangsong*), also known as “eating broadcast,” is an emergent new media phenomenon that first appeared in 2008 [1]; in this phenomenon, mukbang hosts sit in front of the camera for a few hours and live stream their consumption of vast quantities of food while interacting with viewers [2]. Mukbang became popular in South Korea in 2010 and was broadcast through a video streaming platform AfreecaTV (now called “SOOP”) [3]. Due to the advancement of the internet and smartphones, short video platforms, such as YouTube and TikTok are gradually gaining worldwide popularity. Short video platform development has enabled the evolution of the presentation form of mukbang from live broadcasts to prerecorded videos by facilitating the editing and release of these videos to a larger audience [4], enhancing the popularity of mukbang [5], and making mukbang a global trend in 2015 [6]. Currently, mukbang enjoys enormous web-based engagement. Popular mukbang channels have more than 5 million YouTube subscribers, and a single video often attracts hundreds of millions of views [7]. An investigation of the characteristics of mukbang audiences showed that mukbang is more attractive to adolescents aged between 18-28 years [8], and the male-to-female viewer ratio is approximately 1:2 [8]. The YouTube keyword tool indicates the average monthly numbers of searches of the term mukbang over the previous 12 months of data for the United States of America and South Korea to be 365,000 and 9600, respectively [9]. This suggests that mukbang is ubiquitous; it spreads into our living environments and imperceptibly affects people’s lives and daily interactions, particularly among young people. In this study, according to the definition and origin of mukbang, we classify videos with “eating” behavior as their content as mukbang; in other words, mukbang is no longer limited to the display of eating in a live broadcast but covers all web-based videos having eating-related contents.

Earlier studies have extensively investigated and analyzed the components and contents of mukbang. According to these studies, mukbang comprises 2 essential elements: auditory and visual sensory stimulation [10] and web-based interaction [11,12]. In sensory stimulation, distinct chomping, chewing sounds made by hosts can evoke a sensation known as an autonomous sensory meridian response (ASMR) [3,13-15], which refers to experiencing a pleasantly warm and tingling sensation starting at the crown of one’s head and spreading down the body [16]. In addition, the display of food in mukbang also stimulates viewers’ desire for the specific food shown in the videos [17-19]. Another element of mukbang is web-based interaction, which is established by bullet chatting, giving gifts, or liking posts [10,20]. Web-based interaction enables viewers to create emotional relationships with other viewers and the host [21,22]. Further, mukbang’s 2 essential elements can both affect viewers’ emotions and viewing motivations [23]; closely linked to their different psychological needs; and bring them happiness, peace, and vicarious satisfaction [20].

The current trend of mukbang viewing suggests a potential correlation between watching mukbang and specific physiological or psychological needs. Many studies adopt an empirical approach to examine people’s motivation to watch mukbang. Such studies divide the main motivations for watching mukbang into pursuing the thrills of hunger, vicarious eating, emotional establishment, alleviating loneliness, and relieving anorexia [24]. Due to the fact that most of the young viewers are highly educated and experiencing high social pressure and social isolation [25], alleviating the feelings of loneliness associated with eating alone is the most frequently mentioned reason for watching mukbang [21]. Mukbang hosts share fascinating aspects of their lives with their audience while eating, which alleviates the viewers’ feelings of social loneliness. This was particularly relevant during the COVID-19 pandemic [26,27]. Another common motivation to watch mukbang during the pandemic period was to obtain information about food, such as its taste, nutritional value, and cooking procedures [27,28]. Today, social demands to maintain body shape are on the rise; hence, an increasing number of people watch mukbang to experience vicarious satiation, satiety, and enjoyment through visual and auditory stimuli [29]. Furthermore, some viewers watch mukbang to avoid the problems and negative feelings caused by real-life experiences [30]. Therefore, mukbang is often considered to relieve loneliness, reduce negative emotions, and provide psychological pleasure and satisfaction, all of which attract scholars’ attention to the relationship between mukbang and physical and psychological health. Compared with the studies on mukbang, research on food-related videos is more comprehensive. Further, scholars use experimental designs to examine the effects of food-related videos on viewers’ psychology, appetite, and eating behavior from an objective perspective [15,17,31]. According to the results of 4 separate meta-analyses on neural responses to food advertising conducted by Arrona-Cardoza et al [32], van der Laan et al [33], Yeung [34], and Yang et al [35], food images or commercials can cause stronger brain responses than nonfood images or commercials in different areas of the brain. Furthermore, a comparison between high- and low-calorie foods revealed higher neural activation on viewing high-calorie foods than low-calorie foods, particularly among individuals with overweight or obesity [32,33,35,36]. This confirmed that, by affecting neurofeedback in the brain’s food-reward system, food-related videos significantly influence viewers’ appetite, food intake, and eating behaviors [37-39]. Further, Boyland et al [40] and Kidd and Loxton [41] designed a randomized controlled trial (RCT) that suggested that watching food advertisements increases viewers’ food intake. On this basis, some studies indicate that watching mukbang can increase food consumption by augmenting taste sensations [26]. Further, mukbang increases flavor perceptions, help individuals suppress their desire to substitute healthy food with unhealthy ones, and, thereby, improves individuals’ appetite [14].

There is a growing body of research and informational studies on mukbang that encompasses various topics such as audience psychology, food culture, communication effects, food waste, physical and mental health, and the exploration of new mukbang trends. However, the divergent research on mukbang and health yields contradictory findings and conclusions. Researchers consider mukbang a useful tool to improve appetite and food intake [13,42], while in other studies, viewers believe that mukbang has a negative impact on health and dietary behaviors [31]. Furthermore, studies on mukbang’s health effects are scattered across academic fields and fail to provide a general summary or description of the topic; the quality of these studies also varies considerably; thus, due to the complexity of information, readers may struggle to obtain effective insights into the impact of mukbang on physical and mental health. They might overlook the benefits of mukbang in stimulating appetite and increasing food intake or excessively rely on it for weight loss, leading to severe consequences such as overeating. Consequently, there is an urgent need for a comprehensive review that integrates key findings from both academic and nonacademic studies on mukbang’s influence on health based on a thorough screening of existing research. After conducting a comprehensive search of academic and nonacademic studies on both mukbang and health, we integrate the main aspects of each paper to identify and discuss the impact of mukbang on viewers’ physical and psychological health. Further, this study aims to systematically map the research on mukbang and health promotion, identify any research gaps, and reveal the impact of watching mukbang on viewers’ physical and psychological health by combining, comparing, and integrating relevant literature to lay a foundation for the application of mukbang to promote the physical and psychological health of viewers.

### Research Goals

Research reveals that mukbang may affect the physical and psychological health of mukbang hosts and viewers. This study systematically maps the positive aspects of and gaps in research on mukbang’s impact on health to help prevent overweight and obesity and improve the eating behaviors and nutritional status of the public. This scoping analysis addresses the following research questions (RQs).

- RQ1: What are the specific effects of mukbang watching on physical and psychological health?

We reviewed and summarized studies exploring the relationship between mukbang and health or the effects of mukbang on health. Further, we identified the potential effects of mukbang on physical and psychological health promotion and clarify different mukbang-related ideas and health-related issues.

- RQ2: What are viewers’ opinions regarding the impact of mukbang on their physical and psychological health?

We examined mukbang user groups and their perceptions of the relationship between mukbang and health to identify the specific requirements that should be satisfied or improved by mukbang.

- RQ3: What are the future applications of mukbang in the field of health?

Finally, based on the arrangement of the content on the health effects of mukbang in existing studies, we discussed the future prospects of mukbang in promoting appetite, improving dietary behaviors, and enhancing mental health to lay the foundation for its application in medical and health fields.

The findings of this study are intended to benefit health care professionals, researchers, technology providers, and all (both current and potential) viewers in designing, conducting, and evaluating mukbang videos.

## Methods

### Study Design

This scoping review is conducted and reported following the Joanna Briggs Institute (JBI) guideline and the PRISMA-ScR (Preferred Reporting Items for Systematic Reviews and Meta-Analyses extension for Scoping Reviews) checklist (Multimedia Appendix 1) [43]. Further, this scoping review protocol was prospectively registered (INPLASY2022120109) to limit reporting bias. The initial search was conducted in March 2023, and the literature screening and data extraction processes were completed in July 2023.

### Search Strategy

To conduct a comprehensive review, we considered both academic and nonacademic studies on mukbang and health issues. Textbox 1 presents the inclusion and exclusion criteria for this study. We used the keywords mukbang, meokbang, “eating broadcast,” “culinary videos,” “online eating,” “eating show,” “food media,” “health,” “health promotion,” “eating disorder,” “eating behaviors,” “disordered eating,” “binge eating,” and “obesity” to search studies that were published before June 30, 2023, in academic databases. The same keywords in Chinese and Korean were also searched in Chinese and Korean databases, respectively, using similar strategies adjusted based on the database. For gray literature sources, we used simple terms mukbang, “eating broadcast,” “online eating,” ASMR, and health for more comprehensive results. The academic databases were PubMed, Embase, Web of Science, Researching Information Sharing Service (Korean), DBpia Scholarly Database (Korean), China National Knowledge Infrastructure (Chinese), China Science and Technology Journal Database (VIP Database, Chinese), and Wan Fang Data (Chinese). The following study types were retrieved from these databases using optimized searches: review, meta-analysis, observational quantitative, qualitative, mixed, and interventional studies on mukbang and health issues. Gray literature searches were conducted of databases Open Access theses and dissertations, ProQuest, OpenGrey, and Google Scholar, including commentaries, magazine papers, news, blogs, books or book chapters, and ongoing studies, to map the complete academic scope of the impact of mukbang watching on viewers’ health and eating behaviors. We used “Title/Abstract” or “All Fields” as retrieval fields and a Boolean strategy to connect each identified keyword to an appropriate search strategy. We used the conjunction “or” to connect synonyms, while parentheses were used for prioritized searches, and all the keywords with distinct meanings were linked by “and” (Multimedia Appendix 2). The bibliographies of all relevant retrieved studies were also examined to identify further relevant studies. The searched studies were written in English, Chinese, or Korean languages. The decision to consider these languages was motivated by the following consensus since mukbang originated in South Korea, the literature on mukbang in Korean is informative and, hence, should be searched. In addition, since the researchers, who are from China, were interested in identifying possible studies (in Chinese) from China and mukbang has extensive dissemination and audience in China, we decided to include studies from Chinese and Korean databases.

**Inclusion and exclusion criteria.**

**Inclusion criteria**

- Studies describing the relationship between mukbang watching and dietary behaviors, disordered eating, or physical and psychological health.
- Studies demonstrating the impact of mukbang on health (eg, physical health, dietary behaviors, psychological health).
- Studies exploring the reasons for mukbang watching from the perspective of psychological or personal needs, while paying attention to the impact of mukbang on public health.
- Studies focusing on the design, development, or availability of the interventions using mukbang to demonstrate the usefulness of mukbang in the health field.
- Studies focusing on the challenges and obstacles of integrating mukbang videos into clinical practice.
- Food advertisements and food-related videos must include images of celebrities eating.

**Exclusion criteria**

- Studies only involve the basic concepts and situations such as the definition, origin, development, and epidemic situation of mukbang.
- Studies only cover the impact of shooting mukbang videos on the host’s own physical and psychological health but do not mention the effect on the viewers.
- Studies only mention digital media, social software, or social media, but do not clearly indicate mukbang in the full text. Studies only contain food pictures (static representations of food).
- The main field of study was communication, literature, or aesthetics, rather than medicine, psychology, or health.

### Study Selection

In this phase, we evaluated the retrieved studies’ relevance to the review according to the independent inclusion and exclusion criteria presented in Textbox 1. All identified records from the academic databases and gray literature websites were imported into Rayyan (Qatar Computing Research Institute) and EndNote 20 (version 4.1; Clarivate) for further analysis and screening. The Rayyan platform enables multiple collaborators to independently and synchronously screen a shared set of studies, ensuring the confidentiality of each collaborator’s results until the final stage of each step of the screening process. After importing all the data, duplicate results were removed. Two researchers independently screened the titles and abstracts of the retrieved studies and excluded the studies that did not contain mukbang or health elements. The studies that definitely and potentially satisfied the inclusion criteria were tagged “included” and “maybe” using the screening and classification function of Rayyan, respectively. Subsequently, an independent assessment of full-text studies was performed. The full texts of the studies that did not match the search theme or review’s scope were excluded from the study. All the studies whose full texts were unavailable were excluded, as well. Disagreements were solved through discussions and consensus with a third researcher. After the full-text selection, JBI critical appraisal tools were used to evaluate the quality of the included cross-sectional studies, RCTs, quasiexperimental studies, systematic reviews, and qualitative research. We did not conduct a quality appraisal for commentaries since there is no unified writing format or standard requirements.

### Data Extraction and Charting

According to a framework of the JBI template data extraction instrument [44], researchers developed and modified a data extraction chart based on the inclusion criteria, evidence source details and characteristics, information on research methods or tools, mukbang characteristics, and health-related elements (Multimedia Appendix 3). The data extraction chart was used as a research tool to manually extract and integrate useful information from the included studies. Search results were described and narratively synthesized to clarify the effects of mukbang watching on viewers’ physical and psychological health and the comments of the viewers on the relationship between mukbang and health. Further, in line with the purpose of this review, the retrieved studies were sorted, analyzed, and synthesized to clarify the effects of mukbang watching on viewers’ eating behaviors, appetite, food preferences, body weight, and psychological health. Accordingly, we highlighted the numbers and proportions of different types of publications and studies and the target audience, language, country of origin, publication year, disciplinary areas, specific research methods, and key findings of all the included studies (Multimedia Appendix 4). To present the results clearly, we used the year filter of each database to classify the retrieved studies by publication year and counted the numbers and types of studies published in different years. We then plotted the data of the included studies using Excel to graphically show the types of included studies and their trends over the years. Our goal was to collect the contents of the studies describing the health effects of mukbang watching, as well as clarifying the public’s views and attitudes regarding mukbang videos. Through inductive analysis, we characterized the influence of mukbang watching on viewers’ physical and psychological health and described the different attitudes of viewers toward the health-related benefits of mukbang watching.

## Results

### Search Results

**Figure 1:**
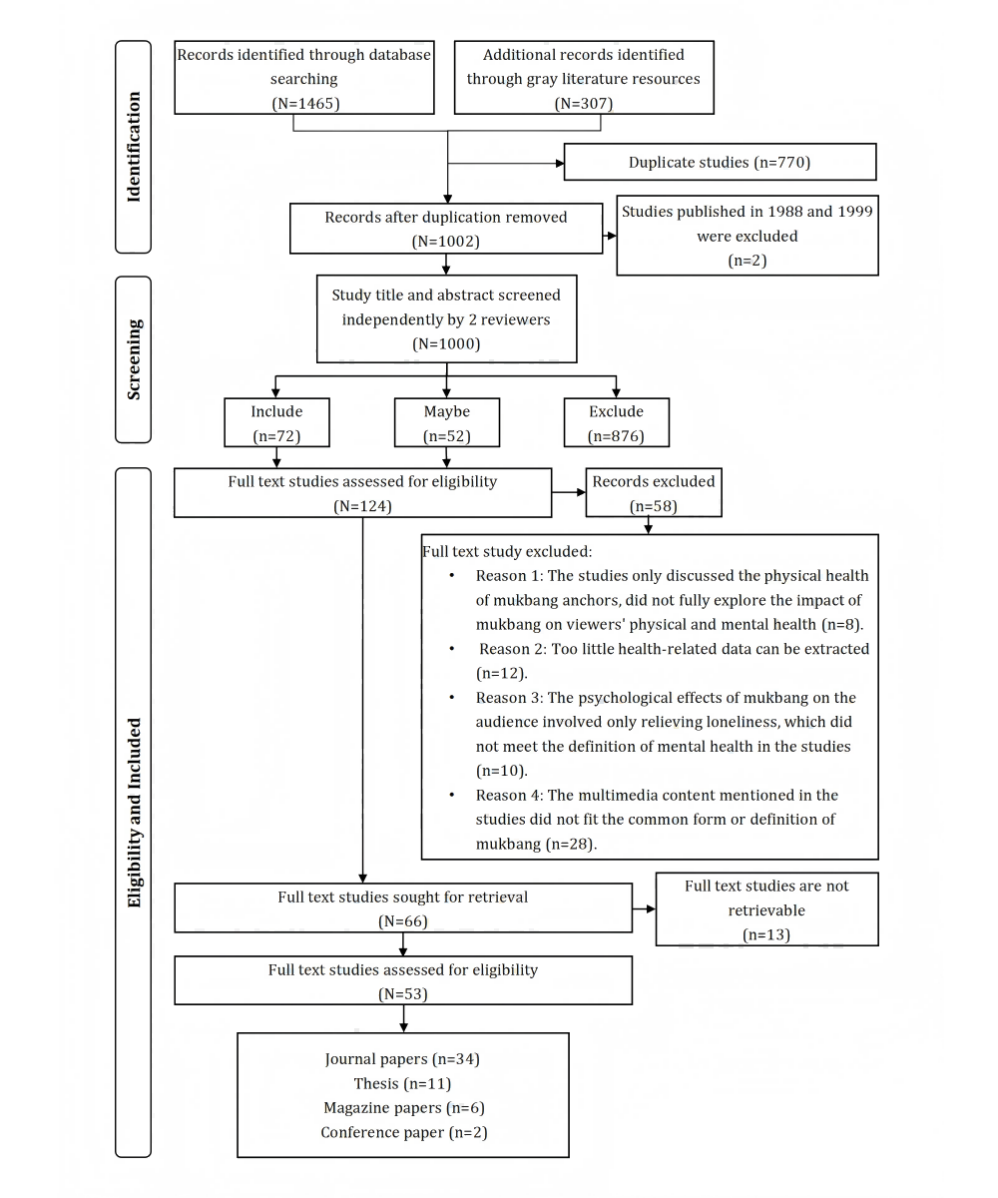
PRISMA (Preferred Reporting Items for Systematic Reviews and Meta-Analyses) flow diagram for studies screening.

Our study search found 1465 publications in databases; 307 additional records were identified through gray literature resources, as well. Following the removal of duplicates, 1002 titles and abstracts were screened independently by 2 reviewers, and 278 ambiguous studies were discussed by reviewers together after the screening. In total, 876 studies were excluded since they did not examine mukbang watching or health; further, 72 studies were included because their titles or abstracts explicitly mentioned mukbang watching and health. In addition, 52 publications were allocated to the maybe list since these studies’ eligibility criteria could not be determined completely from their titles or abstracts. Among them, 124 full-text studies were screened, and 66 publications were included in the review for result extraction and analysis based on the eligibility criteria. Among these 66 studies, since 13 were not available due to limitations such as incomplete full-text coverage, restricted access rights, or inability to establish contact with the original author, 53 publications were finally included in the review. According to the evaluation criteria of JBI [45], the quality evaluation studies basically conform to the writing format and standards of the corresponding research types, the quality evaluation of the included studies is presented in Multimedia Appendix 5 [13,15,17,18,22,24,25,27-30,46-70]. Given that the scoping review aims to ensure the most comprehensive understanding and characterization of existing research by searching for studies relevant to the topic to the greatest extent and including as many studies as possible that meet the inclusion criteria, quality evaluation results have not been used as exclusion criteria. The search and screening procedure is depicted in a PRISMA (Preferred Reporting Items for Systematic Reviews and Meta-Analyses) flow diagram (Figure 1), and some important details of the included studies are presented in Multimedia Appendix 6 [2,13-15,17,18,22,24-31,46,47-69,71-83].

### Study Characteristics

Overall, our search results indicated that studies on mukbang first appeared in 2015, whereas those on mukbang and health first appeared in 2017. Table 1 indicates the number of included studies classified based on different research types. In general, the types of studies on the relationship between mukbang and health mainly include cross-sectional studies, qualitative studies, reviews, commentaries, and intervention studies, which indicates that research focuses on the relationship between mukbang and health becomes more comprehensive, scholars are attempting to explore mukbang’s effects on health using intervention studies with higher levels of evidence. Simultaneously, however, the commentary also accounted for a high proportion of the included studies (n=10, 19%), suggesting that despite the growing academic interest in studying mukbang’s effects on health, many scholars also favor commentary as a genre that allows for greater expression of opinions and insights.

**Table 1:** The number of the included studies, grouped by different research types.

Research types	Studies, n (%)
Cross-sectional study	20 (38)
Interventional study	10 (19)
Commentary	10 (19)
Qualitative study	9 (17)
Review	4 (7)

**Figure 2:**
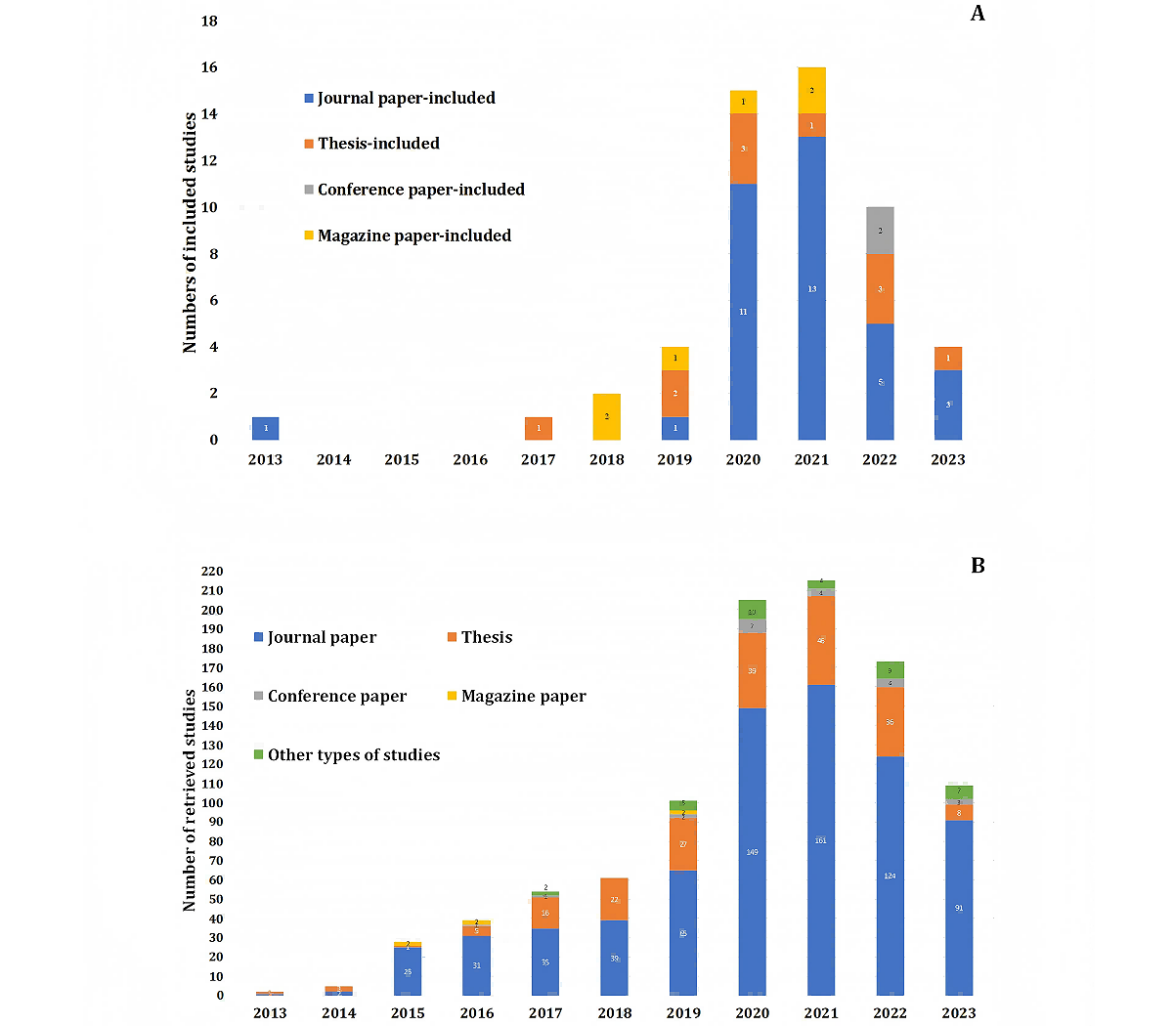
The number of mukbang-related studies per year grouped by different characteristics: (A) all the included studies and (B) all the retrieved studies.

This review considered studies published in scientific journals (n=34, 64.2%), master’s theses (n=12, 22.6%), magazine papers (n=4, 7.5%), conference papers (n=1, 1.9%), book chapters (n=1, 1.9%), and study registrations (n=1, 1.9%). Figure 2 presents combined information on the characteristics and publication years of the included studies focusing on mukbang and health and retrieved studies. Since the earliest mukbang appeared in 2010, we excluded the studies published before 2010 (a total of 10 studies) from the search results. Consequently, the number of “all retrieved studies” amounts to 992. The stacked bar chart below represents the characteristics of all the retrieved resources; The stacked bar chart above represents the characteristics of the included studies. Moreover, to enhance the clarity of the comparison between all retrieved studies and included studies in Figure 2A, we have consolidated newspaper, book, and website studies under a category labeled “Other types of studies.” According to Figure 2B, most studies examining the effects of mukbang on health and nutrition were journal papers and theses, which indicated that the content of mukbang related to dietary habits and health gradually attracted scholarly attention. The number of journal papers and theses on mukbang, which has been increasing since 2015, peaked in 2020 and 2021. South Korea was the first country to publish a mukbang-related study, probably because mukbang originated in Korea and became popular in Korean society earlier than in other countries. Korea is followed by China, which has been publishing a large number of studies every year since 2016. Most Chinese studies on mukbang were published in communication and journalism journals, whereas most English-language studies were published in journals on psychology, nutrition, and health. In this review, only the information relevant to mukbang’s impact on physical and psychological health was extracted from the included studies.

### Mukbang’s Impact on Physical Health

Studies found that mukbang has the potential to provide health-related information to the viewer, and may have positive effects on viewers’ appetite [71], food choices [47], and weight control [48,72]. Based on the included cross-sectional studies, food-related programs such as mukbang and cooking shows, viewing frequency and duration [49] are commonly regarded having a positive impact on food choices, food intake, dietary behaviors [50], and overall health of the viewers [47,51], especially during the COVID-19 pandemic that many people decided to watch mukbang and cooking videos to entertain themselves and cook by themselves to diversify their meals and maintain a healthy lifestyle [27]. A cross-sectional survey also tentatively explored the potential for experiencing vicarious satisfaction through watching mukbang, which indicated that nearly a quarter of the audience watched mukbang when feeling hungry to experience satiation, satisfy their desire for food, and enjoy web-based food spuriously without gaining weight [52]. To explore the aforementioned findings further, some scholars have carried out qualitative and mixed studies. The studies have turned out that the nutritional content of food-related videos may have a positive effect on viewers’ healthy eating behaviors, viewers tend to obtain interesting information about healthy eating from food-related videos [25]. Many observational studies indicated certain impacts of mukbang consumption on viewers’ appetite, food intake, food choice, and dietary preference, some scholars subsequently conducted interventional studies to validate these causal relations. Kim [71] pointed out that watching mukbang with normal emotion may increase viewers’ appetite and food intake. RCTs have been conducted to compare the effects of mukbang and nonfood videos, mukbang and cooking shows, and mukbang with different contents respectively on satiation, disgust, and desire for food, which found that mukbang reduced the viewer’s sense of satiety and promoted their appetite to varying degrees, but it also increased the dieters’ disgust to stabilize their food intake in a relatively balanced state. Interestingly, the study revealed that regardless of the food presented, watching mukbang invariably increased viewers' desire to eat fruits and vegetables [48]. The findings are further supported by other similar studies. Review and commentary studies suggested that mukbang can help people with eating disorder symptoms experience a sense of vicarious satiation to control their appetite and, to some extent, stop endless eating [73]. In contrast, for individuals who engage in excessive dieting, watching mukbang could help them relax their strict dietary requirements and reduce their anxiety regarding dieting to avoid malnutrition [2]. These results confirm that mukbang can increase satiety, control food intake, and avoid overeating and, thereby, help viewers lose weight [2,53].

Nonetheless, mukbang was reported to licit negative effects on certain viewers in some cases. Preliminary investigations of cross-sectional studies indicated that mukbang contains large amounts of high-calorie food may cause concern that it can increase high-calorie food intake and impair the dietary behaviors of mukbang viewers [27,53,54], especially for those watching mukbang frequently [49,55]. von Ash et al [56] investigated the relationship between mukbang viewing and disordered eating behaviors using the Mukbang Addiction Scale and the eating disorders examination questionnaire, which showed a positive relationship between binge eating or purging tendencies and problematic mukbang viewing; moreover, mukbang viewing, and additional characteristics of mukbang viewing (eg, frequency of watching, average watch time, eating while watching), also be found that may be associated with eating disorder symptoms. On this basis, qualitative studies using content analysis and textual analysis to further explore the possible negative impact of mukbang on viewers found that about 83.5% of mukbang presented substantial content showing harmful eating habits like overeating, excessive exposure to it may change the original dietary preferences and eating habits of viewers and increasing their desire to consume amounts of unhealthy foods [18,57]. While some famous mukbang hosts demonstrate excessive food consumption, they are still energetic and keep in shape (particularly in Big Eater Mukbang videos), this contrast may mislead viewers to believe that overeating does not pose any risks to their health and physique [24]. These perspectives above are supported by some commentary studies that look upon mukbang in an unfavorable manner [72,74,75]. Intervention studies shed insights on the impact of food-related videos (like mukbang) and nonfood-related videos on viewers’ eating behavior, food choices, and food intake. They found that mukbang watching increased the consumption of unhealthy food and negatively affected the eating behaviors and physical health of viewers, particularly unsuccessful restrained dieters [17,31,48]. Over time, such unhealthy eating habits can injure the viewers’ stomachs and make them overweight and obese, which seriously affects their health and leads to binge eating, bulimia nervosa, and anorexia nervosa [76].

### Mukbang’s Impact on Psychological Health

Numerous studies have examined mukbang’s impact on mental health. Cross-sectional studies demonstrated that mukbang viewers often choose to watch videos when they feel lonely and desire companionship to satisfy their spiritual needs [58]. Based on the cross-sectional study, the researchers conducted qualitative research by analyzing comments and web-based posts of mukbang videos and conducting semistructured interviews. The findings not only supported that mukbang can release pressure and loneliness and provide enjoyable entertainment, but also revealed that mukbang can digitally enhance viewers’ immersive experience and taste sensations, and help them experience a sense of vicarious satisfaction [30,59]. Researchers also conducted several interventional studies to explore mukbang’s potential effect on the viewer's taste sensations and found that mukbang has the potential to improve viewers’ perceptions of the taste of food and make the food taste delicious [22,26,60,61]. One of the interventional studies has investigated how different video types (including mukbang) affect the taste sensations of viewers eating plain white rice. The trial’s results revealed that both mukbang and cooking videos can enhance viewers’ taste sensation of spiciness and help them find the food is mouthwatering, satisfy their cravings, and attain psychological satiation [14]. Further, reviews and commentary studies both discussed how mukbang meets the psychological needs of its viewers. The integrated results showed that mukbang watching can provide digital companionship to viewers, and relieve their loneliness and daily life pressure, making them feel relaxed and happy [2,62,63,77-80]. In addition to relieving negative emotions, the distinct chomping sounds made by hosts can help relax viewers’ brains and make them fall asleep easily [64,81], the sound made by ASMR mukbang hosts, the attractiveness of mukbang hosts and the presentation of food elicit visual and auditory contentment has been found can make the viewers feel involved in the warm dining atmosphere shown in the video, which further enhances the food’s taste and viewers’ appetites to some extent [62,72,81,82].

### Public Perceptions of the Role of Mukbang in Health Promotion

By using the bullet chatting and commenting functions of short video platforms, people can interact with mukbang hosts and express their thoughts and opinions on the relationship between mukbang and health. Scholars have conducted interviews and text analyses to clarify viewers’ attitudes toward mukbang [13,24,25,46]. An et al [83] used big data and text analysis methods to examine viewers’ comments on popular mukbang videos on YouTube; results indicated that mukbang contents stimulating unhealthy eating behaviors led to negative reactions from viewers, whereas contents featuring healthy eating gained more viewers’ favor and sympathy. The results of some studies on the comments for mukbang content reveal that solitary viewers generally consider mukbang a dining companion who creates a warm dining atmosphere and relieves their feelings of loneliness and social isolation [62]. Other viewers consider mukbang a tool that provides psychological gratification to help relieve immediate hunger and help them control their diet and lose weight [24]. However, regarding Big Eater Mukbang, some viewers felt that the excessive amount of food consumed at one time by mukbang hosts caused food wastage, was extremely harmful to the body, and promoted extremely unhealthy behaviors [13,24,46]. Moreover, they questioned whether the mukbang hosts ate all the food shown in the video since their slim figures suggested otherwise [46]. Along with being concerned about the hosts’ health, the viewers warn themselves and appeal to others not to blindly follow the unhealthy eating behavior shown in the videos. Further, the results of qualitative interviews suggest that most viewers watch mukbang mainly for entertainment and to gain positive emotional value [65]. They were curious about the taste of the food presented in the videos, developed cravings for these specific foods after watching mukbang, and expressed their doubts regarding the very high food intake of mukbang hosts [46].

### Future Applications of Mukbang

Scholars have started analyzing and discussing the advantages and disadvantages of these videos from different perspectives and indicating the future direction of mukbang research by analyzing the shortcomings of their own research in their studies. Some scholars opine that future research should consider various aspects of the mukbang-watching experience, particularly the mechanisms of mukbang-induced overeating and problematic eating [66]. Other scholars clarify that the different dietary statuses and watching motivations of mukbang viewers strongly affect health. James et al [14] and Yoo et al [55] found that extreme and failed dieters are more likely than other dieters to damage their physical health and lead to eating disorders after watching mukbang videos. They indicate that future research can further examine the factors and elements that can help promote healthy behaviors in mukbang videos, spread the concept of a healthy diet through these videos, and incorporate such concepts in medical and health care fields [14,55]. Scholars should focus on the creation of enjoyable and useful content that encourages people to continue watching mukbang and making dietary changes [67]. In addition, mukbang can be considered for future clinical use to address the nutritional problems caused by loss of appetite and picky eating [26].

## Discussion

### Overview

This scoping review addressed and resolved all its research questions, including examining the specific effects (RQ1) and viewers’ opinions (RQ2) of mukbang watching on physical and psychological health, and examined the future applications of mukbang in health promotion (RQ3). In summary, this review addressed and resolved all its research questions. This scoping review synthesized the evidence of the impact of mukbang watching on viewers’ dietary behavior, eating disorders (if any), and overall health. Mukbang is both beneficial and harmful to the viewer’s health and eating behavior, which mainly depends on the viewer's own attitude toward food and weight loss, as well as their attention to their own body. Additionally, it identifies the viewers’ attitudes toward and concerns about mukbang watching, which facilitates the regulation and improvement of mukbang video content and lays a foundation for the videos’ application in health promotion.

### Principal Findings

Many studies cited in this review agree that watching mukbang can positively affect viewers’ dietary behaviors, appetite, food preferences, and physical and psychological health. This may be due to the fact that mukbang can elicit a communal dining experience that contributes to the viewer’s relaxation during consumption; and the visual, auditory, and other sensory stimuli may stimulate the viewer’s brain to synthesize hunger-inducing hormones, consequently reducing satiety and promoting appetite [15,64,68,81]. This result suggests the possibility of using mukbang positively to develop healthy eating habits and appetites by enhancing taste experiences and reducing negative emotions [15,30,81], which might be beneficial for people attempting to improve the taste of a bland diet without indulging in overeating or using unhealthy food additives [13]. The impact of mukbang watching on dietary behaviors and health promotion primarily may depend on the characteristics of mukbang viewers and the content of mukbang videos [46]. Several studies indicate that the main viewers of mukbang are young individuals, who often develop food cravings, dissatisfaction with their body shape, and an obsession with weight loss or dieting behaviors [68]. This may be due to the dietary and sociocultural characteristics of teenagers because they tend to prioritize taste over nutritional value when making food choices, thus they can be easily influenced by mukbang content [69]. For individuals suffering from anorexia nervosa, mukbang can be an effective physical therapy method to increase food intake by stimulating appetite [30]. In addition to the characteristics of mukbang viewers, the content of mukbang videos plays an important role in shaping eating habits, appetite levels, mood states, and overall health [46]. Hence, it is important to change the types of food shown in mukbang videos and gradually incorporate nutritional information to enhance the nutritional value of displayed food and the dietary behaviors of viewers. Additionally, the eating behaviors exhibited by hosts potentially influence viewer behavior. Extreme practices, such as binge eating, should be avoided or corrected to display clean and harmonious dining scenes so that mukbang videos positively influence the audience's eating behavior [59].

However, the impact of mukbang on its viewers is not uniformly positive. Some mukbang videos continue to propagate unhealthy concepts and behaviors, such as excessive consumption of high-calorie foods, misleading viewers to blindly imitate and lead to obesity and gastrointestinal disorders [28,42,72]; further, the overreliance on psychological gratification can lead to mukbang video addiction and endless dieting [29,53]. This means that individuals who achieve weight loss through dieting frequently may choose mukbang as a substitute for conventional eating. However, long-term compensation can engender binge eating, which may culminate in guilt and even depression. This pattern can initiate a cycle of weight loss attempts and mukbang indulgence, ultimately culminating in excessive dietary intake. Thus, mukbang is a double-edged sword that may cause both positive and negative influences on viewers’ dietary habits and mental health. To elucidate this phenomenon, several scholars have presented their own perspectives in the research, a potential rationale for this may be posited as the ambivalence of young individuals toward the pursuit of a desirable physique and the indulgence in delicious food [59]. However, there is a lack of studies investigating the mechanisms underlying the influence of mukbang on eating behavior from an objective perspective, thus making it unclear whether watching mukbang has positive or negative effects on viewers. Based on the findings of this study, these 2 opposite effects of mukbang may be attributed to factors such as content variation, exposure levels, viewer’s self-discipline, and dieting status. Future studies can further explore these influencing factors that contribute to the dual nature of mukbang, helping viewers watch mukbang in a healthier way [48]. Thus, mukbang must be used cautiously in health promotion.

### Implications for Future Research

According to the results of our study, the majority of studies on mukbang and its impact on health primarily focus on the perspective of mukbang anchors or clarify the deleterious effect of performing mukbang on anchors’ health, and only a few studies examine the negative effects of mukbang on viewers' eating behavior and health by using questionnaires and interviews. Future research can further investigate the variations in psychological states among individuals of diverse age groups and genders when exposed to mukbang, as well as examine the impact of mukbang on their psychological well-being [68]. Besides, to date, no scholar has thoroughly investigated whether the negative effects of mukbang on health are real, or clarified which aspects of mukbang videos are responsible for these negative effects; existing studies also do not provide accurate conclusions on the relationship between mukbang and health, researchers have not yet conducted experimental studies from a physiological perspective to examine the objective effects of mukbang videos on viewers’ eating habits, food preferences, and physical and mental health and clarify the mechanisms by which the videos affect viewers’ health. Future studies can focus on exploring the effects of mukbang watching on the physical health of the viewers, or the relationship between disordered eating psychopathology and different mukbang viewing characteristics (eg, frequency of watching, average watch time, eating while watching), using laboratory methods or other rigorous experimental designs with the aid of objective instruments or tools [56]. As excessive problematic food intake is increasingly viewed as an addictive disorder, mukbang is thought to have a potential risk of inducing binge eating, the relationship between mukbang watching and addiction, as well as the influencing factors both worth further investigations to keep mukbang viewers away from addictive behaviors [29,30]. Meanwhile, excessive or problematic mukbang viewing may serve as an indicative signal of potential eating disorders, which can guide clinicians to identify and diagnose eating disorders more effectively. Therefore, mukbang content that may cause the development of eating disorders could be explored and establish criteria for evaluation, so as to help mukbang viewers stay away from those unhealthy mukbang and maintain healthy eating habits while enjoying the pleasant dining atmosphere presented by mukbang [56].

Future research can provide insights into the effect of different mukbang content on different taste perceptions, which can help clarify which mukbang content enhances a particular taste sensation among viewers [14,26]. Additionally, given the specific effect of mukbang on food preference, scholars can further test whether some particular types of mukbang might be useful for dieting and whether mukbang with fast food is the most harmful given these likely increase unhealthy food intake; more work is also needed to address the impact of the feature of web-based communication between anchors and viewers, the figure of the host and the food stimuli in mukbang [48]. In addition, according to Yoon [50], future research can focus on the development of mukbang that integrates nutrition and medicine and assess its impact and satisfaction levels. Simultaneously, studies have found that ASMR mukbang has the potential to reduce viewer’s stress levels, and improve their sleep quality, future research may consider exploring the mechanisms involved or comparing the differences in the physical or mental health of viewers between ASMR videos and ASMR mukbang.

### Strengths and Limitations

This is the first scoping review that systematizes studies on the relationship between mukbang and health and is, therefore, the first study to expand the research possibilities in this field. Studies relevant to the review were identified from both academic databases and gray literature websites in different languages. Since mukbang originated in Korea, Korean databases were specifically searched to obtain a more comprehensive perspective on mukbang-related studies, which might have contributed to the inclusion of studies with different contexts and origins. The term mukbang is not yet a descriptor indexed in databases; however, this was not an impediment to identifying the main studies related to the research objective. Our study comprehensively concluded the potential impact of mukbang on viewers’ health and synthesized the possible causes of mukbang's negative effects on health by integrating and analyzing the included studies; however, we also found that there is a lack of rigorous investigations into the influencing factors of these negative impacts or potential relationships between positive and negative effects of mukbang on health. Meanwhile, this review identified methodological limitations in existing studies, suggesting that future studies could consider using clinical trials and other objective indicators to further explore the actual implications of mukbang on viewers. Furthermore, this review clarifies the role of mukbang in health promotion and, hopefully, eliminates some viewers’ prejudice against mukbang videos and guides the country and media to supervise and manage unhealthy content in mukbang videos.

However, this scoping review has some limitations. First, the major limitation of this study is the uneven quality of the included studies. Considering the potential possibility of using mukbang as a future health intervention, existing high-quality studies on the health effects of mukbang are insufficient to support the conduct of systematic reviews or meta-analyses, thus, we tried to use scoping review to provide direction for future research. Second, in addition to the limitations of the number and quality of mukbang-related studies, this review included several studies focusing on food-related videos (or advertisements), which might have affected the accuracy and specificity of the review’s conclusions. This is because a clear definition of differences between food-related and mukbang videos was not available in the early stages of research. Therefore, we redefined mukbang and clearly distinguished it from food-related videos (or advertisements) to ensure the relevance of our conclusions to the field of study. Third, the categories and associated keywords (eg, health, eating behavior, and eating disorder) might have limited the search results. Hence, we adopted a method to search the results and simultaneously performed manual secondary screening to capture more results pertaining to health promotion. However, we might have missed relevant materials that use other terms or do not explicitly use the searched keywords. Nevertheless, we could present a representative overview of the existing studies on the subject in question.

### Conclusions

This scoping review comprehensively searched databases and other sources for studies on mukbang and health and extracted and summarized the effects of mukbang on viewers’ dietary behaviors, appetite, flavor perception, food preference, and health promotion, as well as their perceptions of and attitudes toward mukbang. The results of this study indicate that mukbang has both positive and negative implications for health. Factors such as the content of mukbang videos and the characteristics and physical states of viewers play crucial roles in influencing health outcomes and dietary behaviors. These findings provide valuable guidance to mukbang hosts and health care providers and promote their collaboration in developing healthy mukbang content and assisting viewers in adopting appropriate viewing habits. Finally, this scoping review underscores the potential benefits of using mukbang videos to promote healthy eating habits and emphasizes the need for future research, which will contribute to our understanding of these videos’ impact on human health.

## Data Availability

All data generated or analyzed during this study are included in this published paper (and Multimedia Appendices 1-6).

## Appendix

Multimedia Appendix 1 PRISMA-ScR (Preferred Reporting Items for Systematic Reviews and Meta-Analyses extension for Scoping Reviews) fillable checklist.

Multimedia Appendix 2 Proposed sample search strategies.

Multimedia Appendix 3 Data extraction chart.

Multimedia Appendix 4 Data extraction chart for all included studies.

Multimedia Appendix 5 Quality evaluation of part of the included articles.

Multimedia Appendix 6 Main information of the included studies.

## Abbreviations

ASMR: autonomous sensory meridian response
JBI: Joanna Briggs InstitutePRISMA: Preferred Reporting Items for Systematic Reviews and Meta-Analyses
PRISMA-ScR: Preferred Reporting Items for Systematic Reviews and Meta-Analyses extension for Scoping Reviews
RQ: research question
RCT: randomized controlled trial

## References

[1] Styawan Z, Buwana DS (2023). Watching attitude factors in delivering mukbang shows. J Humanit Soc Sci Bus.

[2] Donnar G (2017). ‘Food porn’ or intimate sociality: committed celebrity and cultural performances of overeating in meokbang. Celebr Stud.

[3] Jenging R, Mohamad F (2022). Mukbang and me: implications on cognition and physical well-being among undergraduates. J Cogn Sci Hum Dev.

[4] Wu Y (2022). Research on Governance of Network Mukbang Phenomenon [dissertation].

[5] Simth J What is mukbang? And why is it so popular?. fenced.ai.

[6] (2023). Mukbang. Wikipedia.

[7] Harris M (2020). 'I don't like to eat alone': Inside the world of 'mukbangs,' extreme-eating videos that are making YouTubers rich. Insider.

[8] Cheng M (2020). Research on Audience Use and Satisfaction in "Mukbang" Programs [dissertation].

[9] (2023). YouTube keyword tool. Ahrefs.

[10] Reddy NV, Mohabbat AB (2020). Autonomous sensory meridian response: your patients already know, do you?. Cleve Clin J Med.

[11] Bruno AL, Chung S (2017). Mŏkpang: pay me and I’ll show you how much I can eat for your pleasure. J Jpn Korean Cine.

[12] Song H (2018). The making of microcelebrity: AfreecaTV and the younger generation in neoliberal South Korea. Soc Media Soc.

[13] Strand M, Gustafsson SA (2020). Mukbang and disordered eating: a netnographic analysis of online eating broadcasts. Cult Med Psychiatry.

[14] James M, Ranasinghe N, Tang A, Oehlberg L (2022). Watch your flavors: augmenting people's flavor perceptions and associated emotions based on videos watched while eating.

[15] Han Y (2022). Study on emotional and physiological changes according to food content types and scenes—ASMR mukbang vs nomal mukbang. J Image Cult Contents.

[16] Poerio GL, Blakey E, Hostler TJ, Veltri T (2018). More than a feeling: autonomous sensory meridian response (ASMR) is characterized by reliable changes in affect and physiology. PLoS One.

[17] Bodenlos JS, Wormuth BM (2013). Watching a food-related television show and caloric intake. A laboratory study. Appetite.

[18] Castelló-Martínez A, Tur-Viñes V (2020). Obesity and food-related content aimed at children on YouTube. Clin Obes.

[19] Halford JC, Boyland EJ, Hughes GM, Stacey L, McKean S, Dovey TM (2008). Beyond-brand effect of television food advertisements on food choice in children: the effects of weight status. Public Health Nutr.

[20] Kircaburun K, Stavropoulos V, Harris A, Calado F, Emirtekin E, Griffiths MD (2020). Development and validation of the mkbang addiction scale. Int J Ment Health Addiction.

[21] Chen R (2019). Study on the use of eating webcast' and its impact on mood loneliness—taking XX university students as an example [dissertation]. Anhui University.

[22] Wang C, Peng Y, Qiu L, Wan X (2021). Cloud-based commensality: enjoy the company of co-diners without social facilitation of eating. Front Psychol.

[23] Frankel A Mukbang is changing digital communications. Anthropology News.

[24] Wang S (2020). A Study on Potential Health Issues Behind the Popularity of "Mukbang" in China [dissertation].

[25] Teng S, Khong KW, Pahlevan Sharif S, Ahmed A (2020). YouTube video comments on healthy eating: descriptive and predictive analysis. JMIR Public Health Surveill.

[26] James MN, Ranasinghe N, Tang A, Oehlberg L (2022). Flavor-videos: Enhancing the flavor perception of food while eating with videos.

[27] Kang H, Yun S, Lee H (2021). Dietary life and mukbang- and cookbang-watching status of university students majoring in food and nutrition before and after COVID-19 outbreak. J Nutr Health.

[28] Yun S, Kang H, Lee H (2020). Mukbang- and cookbang-watching status and dietary life of university students who are not food and nutrition majors. Nutr Res Pract.

[29] Kircaburun K, Savcı M, Emirtekin E, Griffiths MD (2022). Uses and gratifications of problematic mukbang watching—the role of eating and social gratification: a pilot study. J Psychiatr Res.

[30] Kircaburun K, Balta S, Emirtekin E, Demetrovics Z, Griffiths MD, Tosuntas (2021). Compensatory usage of the internet: the case of mukbang watching on youTube. Psychiatry Investig.

[31] Alblas MC, Mollen S, Fransen ML, van den Putte B (2021). See the cake and have it too? Investigating the effect of watching a TV cooking show on unhealthy food choices. Physiol Behav.

[32] Arrona-Cardoza P, Labonté K, Cisneros-Franco JM, Nielsen DE (2023). The effects of food advertisements on food intake and neural activity: a systematic review and meta-analysis of recent experimental studies. Adv Nutr.

[33] van der Laan LN, de Ridder DTD, Viergever MA, Smeets PAM (2011). The first taste is always with the eyes: a meta-analysis on the neural correlates of processing visual food cues. Neuroimage.

[34] Yeung AWK (2021). Brain responses to watching food commercials compared with nonfood commercials: a meta-analysis on neuroimaging studies. Public Health Nutr.

[35] Yang Y, Wu Q, Morys F (2021). Brain responses to high-calorie visual food cues in individuals with normal-weight or obesity: an activation likelihood estimation meta-analysis. Brain Sci.

[36] Belfort-DeAguiar R, Seo D (2018). Food cues and obesity: overpowering hormones and energy balance regulation. Curr Obes Rep.

[37] Bruce AS, Pruitt SW, Ha O, Cherry JBC, Smith TR, Bruce JM, Lim S (2016). The influence of televised food commercials on children's food choices: evidence from ventromedial prefrontal cortex activations. J Pediatr.

[38] Gearhardt AN, Yokum S, Harris JL, Epstein LH, Lumeng JC (2020). Neural response to fast food commercials in adolescents predicts intake. Am J Clin Nutr.

[39] Masterson TD, Stein WM, Beidler E, Bermudez M, English LK, Keller KL (2019). Brain response to food brands correlates with increased intake from branded meals in children: an fMRI study. Brain Imaging Behav.

[40] Boyland EJ, Burgon RH, Hardman CA (2017). Reactivity to television food commercials in overweight and lean adults: physiological, cognitive and behavioural responses. Physiol Behav.

[41] Kidd C, Loxton NJ (2018). Junk food advertising moderates the indirect effect of reward sensitivity and food consumption via the urge to eat. Physiol Behav.

[42] Sanskriti S, Guglani I, Joshi S, Anjankar A (2023). The spectrum of motivations behind watching mukbang videos and its health effects on its viewers: a review. Cureus.

[43] Tricco AC, Lillie E, Zarin W, O'Brien KK, Colquhoun H, Levac D, Moher D, Peters MD, Horsley T, Weeks L, Hempel S, Akl EA, Chang C, McGowan J, Stewart L, Hartling L, Aldcroft A, Wilson MG, Garritty C, Lewin S, Godfrey CM, Macdonald MT, Langlois EV, Soares-Weiser K, Moriarty J, Clifford T, Tunçalp Ö, Straus SE (2018). PRISMA extension for Scoping Reviews (PRISMA-ScR): checklist and explanation. Annals of Internal Medicine.

[44] Peters MDJ, Godfrey C, McInerney P, Munn Z, Tricco AC, Khalil H, Aromataris E, Lockwood C, Porritt K, Pilla B, Jordan Z (2024). Scoping Reviews (2020). JBI Manual for Evidence Synthesis.

[45] Critical appraisal tools. JBI.

[46] Sultana SFS, Das P (2022). Content analysis of mukbang videos: preferences, attitudes and concerns. J Posit Sch Psychol.

[47] Shin K (2021). A Study on Food and Nutrition-Related Media Consumption and its Influence on Dietary Habits of Adolescents and Adults in Daegu and Gyeongbuk Regio [dissertation].

[48] Xu W (2019). Does Watching Mukbangs Help You Diet? The Effect of the Mukbang on the Desire to Eat [dissertation].

[49] Kang D (2022). Some Elementary School Students in Ulsan Mukbang Viewing Status and Eating Behavior [dissertation].

[50] Yoon S (2017). A Study on the Influence of Information Characteristics of TV Cooking Program on its Information Acceptance and Dietary Change [dissertation].

[51] Bang SY (2022). A Study on the Eating Habits and Health Behavior of People in Their 20s and 30s According to the Use of Social Media Food Content [dissertation].

[52] Di Y (2022). A Study on the Motivation and Influence of Watching Network Mukbang Programs [dissertation].

[53] Kircaburun K, Yurdagül C, Kuss D, Emirtekin E, Griffiths MD (2020). Problematic mukbang watching and its relationship to disordered eating and internet addiction: a pilot study among emerging adult mukbang watchers. Int J Ment Health Addiction.

[54] Park S (2022). A Study on the Use of YouTube Food Content and the Actual Consumption of Delivery Food by University Students in Gwangju [dissertation].

[55] Yoo S, Shin G, Kim S (2021). Does mukbang watching really affect obesity?: Focusing on the factors related to health and mukbang watching. Korean J Journal Commun Stud.

[56] von Ash T, Huynh R, Deng C, White MA (2023). Associations between mukbang viewing and disordered eating behaviors. Int J Eat Disord.

[57] Kang E, Lee J, Kim KH, Yun YH (2020). The popularity of eating broadcast: content analysis of "mukbang" YouTube videos, media coverage, and the health impact of "mukbang" on public. Health Informatics J.

[58] Li B (2020). Correlation analysis of watching motivation of mukbang and the influence of mukbang on audience. PR Mag.

[59] Zhang L, Cui L (2020). [What satisfaction can you get from watching others eat?—A qualitative study based on the audience of Bilibili]. J News Res.

[60] Kawai N, Guo Z, Nakata R (2021). Watching a remote-video confederate eating facilitates perceived taste and consumption of food. Physiol Behav.

[61] Ngqangashe Y, Backer CJSD (2021). The differential effects of viewing short-form online culinary videos of fruits and vegetables versus sweet snacks on adolescents' appetites. Appetite.

[62] Ma M, Yang J (2021). [The rise of digital table: a review of foreign researches on mukbang]. News Res.

[63] Han C, Yin X (2018). Psychological exploration of "Mukbang" audience from the perspective of structuralism. J Hebei Univ Econ Bus.

[64] Zhou J (2023). Research on the relieving effect of ASMR chewing sounds on anxiety in food video. Int J Educ Humanit.

[65] Ngqangashe Y, Maldoy K, De BC, Vandebosch H (2022). Exploring adolescents' motives for food media consumption using the theory of uses and gratifications. Communications.

[66] Kim J, Choi S, Kim H, An S (2021). Binge drinking and obesity-related eating: the moderating roles of the eating broadcast viewing experience among Korean adults. Int J Environ Res Public Health.

[67] Jo S, Choi H (2021). [A study on the cookbang YouTube program use and dietary change based on the technology acceptance model]. Culinary Sci Hosp Res.

[68] Lee D (2019). A Research on the Causal Factor to Binge Eating That Affects Dieting Deriving From One Person Media Viewership—Focused on Females Between the Ages of 20-30 [dissertation].

[69] Lee S, Lee SH (2022). Actual status of mukbang viewing and food habits of university students in Wonju area. Korean J Community Living Sci.

[70] Alblas MC (2021). Consuming Media, Consuming Food? Reactivity to Palatable Food Cues in Television Content.

[71] Kim SY (2020). The Effect of Mukbang Show and Induced Anxiety on Eating Behavior [dissertation].

[72] Li Y (2020). [Get out of the emotional hunger—psychological and behavioral analysis of the popularity of mukbang]. Psychol Health.

[73] Pan M (2020). An analysis of mukbang watching and watching behaviors of restricted eating groups: based on vicarious gratification theory. Radio TV J.

[74] Wei J (2018). [The reflect on the negative impact of "mukbang fever" on teenagers]. Youth Soc.

[75] Gao Z (2021). The impacts and countermeasure of the novelty seeking live videos chaos on teenagers. Sci Technol Inf.

[76] Nam NH (2020). A Study on the Prevalence of Watching Mukbang and Factors Related Food Behaviors in Adults [dissertation].

[77] Kircaburun K, Harris A, Calado F, Griffiths MD (2020). The psychology of mukbang watching: a scoping review of the academic and non-academic literature. Int J Ment Health Addiction.

[78] Pereira-Castro MR, Pinto AG, Caixeta TR, Monteiro RA, Bermúdez XPD, Mendonça AVM (2022). Digital forms of commensality in the 21st century: a scoping review. Int J Environ Res Public Health.

[79] Fangxiu Liu (2018). [Research on audience psychology and profit model of mukbang from the perspective of communication]. News Dissem.

[80] Tian R (2020). [Psychological research of mukbang audience from the perspective of use and satisfaction theory]. West Broadcast Telev.

[81] Zhong X (2021). [The study of ASMR mukbang on network platforms]. Satellite TV IP Multimedia.

[82] Wu M (2019). [The reason of "Mukbang Heat" phenomenon from the perspective of communication science]. West Broadcast Telev.

[83] An S, Lim Y, Lee H (2020). A study of viewers' comments on online mukbang videos: a big-data analysis of perceptions toward eating behavior. Korean J Journal Commun Stud.

