# Supplementary material for: Use of Mukbang in Health Promotion: Scoping Review
Source: J Med Internet Res. 2025 Mar 27;27:e56147. doi: 10.2196/56147 (PMC11986381; doi:10.2196/56147)
Supplement: Multimedia Appendix 2 [file jmir_v27i1e56147_app2.docx]

**Multimedia Appendix 2. Proposed sample search strategies in English databases and Google Scholar**

## Search Strategy for MEDLINE (PubMed)

| **Search** | **Keywords** | | **Query** | **Record Retrieved** |
| --- | --- | --- | --- | --- |
| #1 |  | “mukbang” | ((((((((mukbang) OR (meokbang)) OR ("eating broadcast")) OR ("culinary videos")) OR ("online eating")) OR ("eating show")) OR ("food media")) OR (mukbang[Title/Abstract])) OR ("eating broadcast"[Title/Abstract])) AND (((((((health) OR ("health promotion")) OR ("disordered eating")) OR ("eating disorder")) OR ("binge eating")) OR ("eating behaviours")) OR (obesity) | 59 |
|  |  | “meokbang” |  |  |
|  |  | “eating broadcast” |  |  |
|  |  | “culinary videos” |  |  |
|  |  | “online eating” |  |  |
| #2 |  | “eating show” | (("autonomous"[All Fields] OR "autonomously"[All Fields]) AND ("sensorial"[All Fields] OR "sensorially"[All Fields] OR "sensory"[All Fields]) AND ("meridians"[MeSH Terms] OR "meridians"[All Fields] OR "meridian"[All Fields]) AND ("response"[All Fields] OR "responses"[All Fields] OR "responsive"[All Fields] OR "responsiveness"[All Fields] OR "responsivenesses"[All Fields] OR "responsives"[All Fields] OR "responsivities"[All Fields] OR "responsivity"[All Fields])) | 67 |
|  |  | “food media” |  |  |
|  |  | “health” |  |  |
|  |  | “health promotion” |  |  |
|  |  | “eating disordered” |  |  |
|  |  | “eating behaviors” |  |  |
|  |  | “disordered eating” |  |  |
|  |  | “binge eating” |  |  |
|  |  | “obesity” |  |  |
|  |  | “autonomous sensory meridian response” |  |  |

## Search Strategy for Embase

| **Search** | **Keywords** | | **Query** | **Record Retrieved** |
| --- | --- | --- | --- | --- |
| #1 |  | “mukbang” | (mukbang OR meokbang OR 'eating broadcast' OR 'culinary videos' OR 'online eating' OR 'eating show' OR 'food media' OR mukbang:ab,ti OR 'eating broadcast':ab,ti) AND (health OR 'health promotion' OR 'eating behaviors' OR 'eating disorder' OR 'disordered eating' OR 'eating behaviors' OR 'binge eating' OR obesity) | 61 |
|  |  | “meokbang” |  |  |
|  |  | “eating broadcast” |  |  |
|  |  | “culinary videos” |  |  |
|  |  | “online eating” |  |  |
| #2 |  | “eating show” | autonomous AND ('sensory'/exp OR sensory) AND ('meridian'/exp OR meridian) AND ('response'/exp OR response) OR 'autonomous sensory meridian response'/exp OR 'autonomous sensory meridian response' | 142 |
|  |  | “food media” |  |  |
|  |  | “health” |  |  |
|  |  | “health promotion” |  |  |
|  |  | “eating disordered” |  |  |
|  |  | “eating behaviors” |  |  |
|  |  | “disordered eating” |  |  |
|  |  | “binge eating” |  |  |
|  |  | “obesity” |  |  |
|  |  | “autonomous sensory meridian response” |  |  |
| #3 |  |  | #1 AND #2 | 1 |

## Search Strategy for Web of Science (All Databases)

| **Search** | **Keywords** | | **Query** | **Record Retrieved** |
| --- | --- | --- | --- | --- |
| #1 |  | “mukbang” | ((((((TS=(mukbang)) OR TS=(meokbang)) OR TS=("eating broadcast")) OR TS=("culinary videos")) OR TS=("online eating")) OR TS=("eating show")) OR TS=("food media") AND ((((((TS=(health)) OR TS=("health promotion")) OR TS=("eating behaviors")) OR TS=("eating disorder")) OR TS=("disordered eating")) OR TS=("binge eating")) OR TS=(obesity) | 143 |
|  |  | “meokbang” |  |  |
|  |  | “eating broadcast” |  |  |
|  |  | “culinary videos” |  |  |
|  |  | “online eating” |  |  |
| #2 |  | “eating show” | TS= “Autonomous Sensory Meridian Response” | 181 |
|  |  | “food media” |  |  |
|  |  | “health” |  |  |
|  |  | “health promotion” |  |  |
|  |  | “eating disordered” |  |  |
|  |  | “eating behaviors” |  |  |
|  |  | “disordered eating” |  |  |
|  |  | “binge eating” |  |  |
|  |  | “obesity” |  |  |
|  |  | “autonomous sensory meridian response” |  |  |
| #3 |  |  | #1 AND #2 | 3 |

## Search Strategy for Researching Information Sharing Service (RISS, Korean)

| **Search** | **Keywords** | | **Query** | **Record Retrieved** |
| --- | --- | --- | --- | --- |
| #1 |  | “먹방”(Mukbang) | 먹방 (Mukbang) | 228 |
| #2 |  | “먹방유투브”  (Mukbang youtube) | 전체 (All Fields): 먹방유투브(Mukbang youtube) \| 유투브 먹방(YouTube Mukbang) | 70 |
| #3 |  | “유투브 먹방”  (YouTube Mukbang) | 먹방 채널 (Mukbang channel) | 41 |
| #4 |  | “먹방 채널”  (Mukbang channel) | 먹방 콘텐츠 (Mukbang contents) | 119 |
| #5 |  | “먹방 콘텐츠”  (Mukbang contents) | 먹방 Asmr (Mukbang ASMR) | 16 |
| #6 |  | “먹방 Asmr”  (Mukbang ASMR) | 전체(All Fields): 쿡방 (Cooking broadcasts) \| 먹방 (Eating broadcasts) | 87 |
|  |  | “쿡방”  (Cooking broadcasts) |  |  |
|  |  | “먹방”  (Eating broadcasts) |  |  |

Search Strategy for DBpia Scholarly Database (DBpia, Korean)

| **Search** | **Keywords** | | **Query** | **Record Retrieved** |
| --- | --- | --- | --- | --- |
| #1 |  | “먹방”(Mukbang) | All = 먹방 (Mukbang) OR (All = 먹방유투브 (Mukbang youtube) OR All = 유투브 먹방 (YouTube Mukbang)) OR All = 먹방 채널 (Mukbang channel) OR All = 먹방 콘텐츠 (Mukbang contents) OR All = 먹방 Asmr (Mukbang ASMR) OR (All = 쿡방 (Cooking broadcasts) OR 먹방 (Eating broadcasts)) | 119 |
|  |  | “먹방유투브”  (Mukbang youtube) |  |  |
|  |  | “유투브 먹방”  (YouTube Mukbang) |  |  |
|  |  | “먹방 채널”  (Mukbang channel) |  |  |
|  |  | “먹방 콘텐츠”  (Mukbang contents) |  |  |
|  |  | “먹방 Asmr”  (Mukbang ASMR) |  |  |
|  |  | “쿡방”  (Cooking broadcasts) |  |  |
|  |  | “먹방”  (Eating broadcasts) |  |  |

## Search Strategy for China National Knowledge Infrastructure (CNKI, Chinese)

| **Search** | **Keywords** | | **Query** | **Record Retrieved** |
| --- | --- | --- | --- | --- |
| #1 |  | “mukbang” (吃播) | SU %= '吃播'OR SU %= '吃播视频' OR SU %= '网络吃播' OR SU %= '吃播节目' OR SU %= '吃播秀' OR SU %= '吃播短视频' OR SU %= '吃饭直播' OR SU %= '饮食秀' OR SU %= '烹饪视频' | 128 |
|  |  | “meokbang”(吃播) |  |  |
|  |  | “eating broadcast”  (吃饭直播) |  |  |
|  |  | “culinary videos”  (烹饪视频) |  |  |
|  |  | “online eating”  (吃饭直播) |  |  |
|  |  | “eating show”  (吃播秀) |  |  |
|  |  | “food media”  (网络吃播) |  |  |
| #2 |  | “autonomous sensory meridian response”  (自主感觉经络反应, ASMR) | SU %= '自主感觉经络反应' OR SU %= 'ASMR' | 101 |
| #3 |  | “health” (健康) | SU %= '健康' OR SU %= '健康促进' OR SU %= '饮食行为' OR SU %= '进食障碍' OR SU %= '暴饮暴食' OR SU %= '肥胖' | 1528200 |
| #4 |  | “health promotion”  (健康促进) | (#1 OR #2) AND #3 | 15 |
|  |  | “eating disordered”  (进食障碍) |  |  |
|  |  | “eating behaviors”  (饮食行为) |  |  |
|  |  | “disordered eating”  (进食障碍) |  |  |
|  |  | “binge eating”  (暴饮暴食) |  |  |
|  |  | “obesity” (肥胖) |  |  |

## Search Strategy for China Science and Technology Journal Database (VIP Database, Chinese)

| **Search** | **Keywords** | | **Query** | **Record Retrieved** |
| --- | --- | --- | --- | --- |
| #1 |  | “mukbang” (吃播) | U=吃播 OR U=吃饭直播 OR U=网络吃播 OR U=吃播秀 OR U=烹饪视频 | 900 |
|  |  | “meokbang”(吃播) |  |  |
|  |  | “eating broadcast”  (吃饭直播) |  |  |
|  |  | “culinary videos”  (烹饪视频) |  |  |
|  |  | “online eating”  (吃饭直播) |  |  |
|  |  | “eating show”  (吃播秀) |  |  |
|  |  | “food media”  (网络吃播) |  |  |
| #2 |  | “autonomous sensory meridian response”  (自主感觉经络反应, ASMR) | U=自主感觉经络反应 OR U=ASMR | 209 |
| #3 |  | “health” (健康) | U=健康 OR U=健康促进 OR U=饮食行为 OR U=进食障碍 OR U=暴饮暴食 OR U=肥胖 | 8924692 |
| #4 |  | “health promotion”  (健康促进) | (#1 OR #2) AND #3 | 187 |
|  |  | “eating disordered”  (进食障碍) |  |  |
|  |  | “eating behaviors”  (饮食行为) |  |  |
|  |  | “disordered eating”  (进食障碍) |  |  |
|  |  | “binge eating”  (暴饮暴食) |  |  |
|  |  | “obesity” (肥胖) |  |  |

Search Strategy for Wan Fang Data (Chinese)

| **Search** | **Keywords** | | **Query** | **Record Retrieved** |
| --- | --- | --- | --- | --- |
| #1 |  | “mukbang” (吃播) | 吃播 or 吃饭直播 or 网络吃播 or 吃播秀 or 烹饪视频 | 8327 |
|  |  | “meokbang”(吃播) |  |  |
|  |  | “eating broadcast”  (吃饭直播) |  |  |
|  |  | “culinary videos”  (烹饪视频) |  |  |
|  |  | “online eating”  (吃饭直播) |  |  |
|  |  | “eating show”  (吃播秀) |  |  |
|  |  | “food media”  (网络吃播) |  |  |
| #2 |  | “autonomous sensory meridian response”  (自主感觉经络反应, ASMR) | 自主感觉经络反应 or ASMR | 380 |
| #3 |  | “health” (健康) | U=健康 OR U=健康促进 OR U=饮食行为 OR U=进食障碍 OR U=暴饮暴食 OR U=肥胖 | 4950753 |
| #4 |  | “health promotion”  (健康促进) | (#1 OR #2) AND #3 | 1243 |
|  |  | “eating disordered”  (进食障碍) |  |  |
|  |  | “eating behaviors”  (饮食行为) |  |  |
|  |  | “disordered eating”  (进食障碍) |  |  |
|  |  | “binge eating”  (暴饮暴食) |  |  |
|  |  | “obesity” (肥胖) |  |  |

## Search strategy for Google Scholar: searching for grey literature

| **Website** | **Keywords** | | **Results** |
| --- | --- | --- | --- |
| https://scholar.google.com/ |  | #1. mukbang and health mukbang mukbang OR "eating broadcast" OR "online eating" OR "mukbang video*" | 244 |
|  |  | #2. "Autonomous Sensory Meridian Response" OR ASMR | 63 |
