# Supplementary material for: Use of Mukbang in Health Promotion: Scoping Review
Source: J Med Internet Res. 2025 Mar 27;27:e56147. doi: 10.2196/56147 (PMC11986381; doi:10.2196/56147)
Supplement: Multimedia Appendix 3 [file jmir_v27i1e56147_app3.docx]

**Multimedia Appendix 3. Data extraction chart**

|  | **Items** | **Data** | | | | |
| --- | --- | --- | --- | --- | --- | --- |
| Inclusion criteria | Population/Main research object |  | | | | |
|  | Concept (Tick)  Studies focusing on | 1. | *mukbang* watching (Food ads, food related videos*) | |  | |
|  |  | 2. | the impact of *mukbang* (Food ads, food related videos) on health | |  | |
|  |  | 3. | the relationship between *mukbang* (Food ads, food related videos) and health | |  | |
|  |  | 4. | the design, development, or usability of *mukbang* (Food ads, food related videos) watching interventions to demonstrate the usefulness of *mukbang* in the health field | |  | |
|  |  | 5. | evaluating the effectiveness of various types of *mukbang* (Food ads, food related videos) for public health | |  | |
|  |  | 6. | the challenges and barriers of integrating *mukbang* (Food ads, food related videos) videos into clinical practice | |  | |
|  |  | 7. | the advantages and/or disadvantages of *mukbang* (Food ads, food related videos) watching | |  | |
|  | Types of evidence source |  | | | | |
| Evidence source details and characteristics | Citation details (eg, author/s, date, title, journal, volume, issue, pages) | Title | |  | | |
|  |  | Journal | |  | | |
|  |  | Author/s | |  | | |
|  |  | Date | |  | | |
|  | Main content of the evidence |  | | | | |
|  | Participant details (eg, age/sex and number) | Age | |  | | |
|  |  | Sex | |  | | |
|  |  | Final enrollment | |  | | |
| Details/results extracted from source of evidence | Review question addressed  (Tick) | 1. | The health-related elements in *mukbang* (Food ads, food related videos) | |  | |
|  |  | Source text |  | | | |
|  |  | 2. | The relationship between *mukbang* (Food ads, food related videos) and health | |  | |
|  |  | Source text |  | | | |
|  |  | 3. | Impacts may *mukbang* (Food ads, food related videos) have on eating habits or eating behaviors | |  | |
|  |  | Source text |  | | | |
|  |  | 4. | The design, development, or usability of *mukbang* (Food ads, food related videos) watching interventions to demonstrate the usefulness of *mukbang* in the health field | | |  |
|  |  | Source text |  | | | |
|  |  | 5. | Evaluation of the health effects of various types of *mukbang* (Food ads, food related videos) | | |  |
|  |  | Source text |  | | | |
|  |  | 6. | The challenges and barriers of integrating *mukbang* (Food ads, food related videos) videos into clinical practice | | |  |
|  |  | Source text |  | | | |
|  |  | 7. | The advantages and/or disadvantages of *mukbang* (Food ads, food related videos) watching | | |  |
|  |  | Source text |  | | | |
|  | Research method/Tools |  | | | | |
|  | Characteristics of *mukbang* |  | | | | |
|  | Health-related elements |  | | | | |
|  | Summary of key findings related to health |  | | | | |

*Food ads and food related videos must include images of celebrities eating.
