# Supplementary material for: Use of Mukbang in Health Promotion: Scoping Review
Source: J Med Internet Res. 2025 Mar 27;27:e56147. doi: 10.2196/56147 (PMC11986381; doi:10.2196/56147)
Supplement: Multimedia Appendix 4 [file jmir_v27i1e56147_app4.docx]

1. YouTube Video Comments on Healthy Eating Descriptive and Predictive Analysis

|  | Items | Data | | | | |
| --- | --- | --- | --- | --- | --- | --- |
| Inclusion criteria | Population/Main research object | The objectives of the study are to explore the determinants, motives, and barriers to healthy eating behaviors in online communities and provide insight into YouTube video commenters’ perceptions and sentiments of healthy eating through text mining techniques.  The study addresses the following questions:  1) Can we identify the relevant topics and categories of healthy eating comments meaningfully?  2) Can we interpret people’s sentiments related to healthy eating? | | | | |
|  | Concept (Tick)  Studies focusing on | 1. | *mukbang* watching (Food ads*) | | √ | |
|  |  | 2. | the impact of *mukbang* (Food ads) on health | | √ | |
|  |  | 3. | the relationship between *mukbang* (Food ads) and health | | √ | |
|  |  | 4. | the design, development, or usability of *mukbang* (Food ads) watching interventions to demonstrate the usefulness of *mukbang* in the health field | | NA | |
|  |  | 5. | evaluating the effectiveness of various types of *mukbang* (Food ads) for public health | | NA | |
|  |  | 6. | the challenges and barriers of integrating *mukbang* (Food ads) videos into clinical practice | | √ | |
|  |  | 7. | the advantages and/or disadvantages of *mukbang* (Food ads) watching | | √ | |
|  | Types of evidence source | A qualitative study | | | | |
| Evidence source details and characteristics | Citation details (eg, author/s, date, title, journal, volume, issue, pages) | Title | | YouTube Video Comments on Healthy Eating: Descriptive and Predictive Analysis | | |
|  |  | Journal | | JMIR Public Health and Surveillance | | |
|  |  | Author/s | | Shasha Teng, PhD; Kok Wei Khong, PhD; Saeed Pahlevan Sharif, PhD; Amr Ahmed, PhD | | |
|  |  | Date | | 2020-10-1 | | |
|  | Main content of the evidence | This study conducted an in-depth content analysis of YouTube comments. The text analytics method was employed as it is useful for discussing and exploring large-scale YouTube-specific phenomenon. This study provides 10 main categories related to healthy eating, including food choice, food price, cooking method, cultural influence, weight management, and so on. There to be significant relationships between these categories and healthy eating. Through the lens of text analytics and sentiment analysis, our results suggest that people have a largely positive attitude toward healthy eating. This study contributes to the perceptions of healthy eating by categorizing the determinants, benefits, and barriers to healthy eating such as food choice, food price, weight management, and cultural influence. | | | | |
|  | Participant details (eg, age/sex and number) | Age | | NA | | |
|  |  | Sex | | NA | | |
|  |  | Final enrollment | | 10 videos, 5756 comments and replies posted under the 10 videos were scraped from YouTube. | | |
| Details/results extracted from source of evidence | Review question addressed  (Tick) | 1. | The health-related elements in *mukbang* (Food ads) | | √ | |
|  |  | Source text | Comments from: videos related to healthy eating in YouTube. In line with the relevance of YouTube video contents of healthy eating in Malaysia.  Healthy eating: 10 clusters identified with regard to food ingredients, food price, food choice, food portion, well-being, cooking, and culture in the concept of healthy eating. | | | |
|  |  | 2. | The relationship between *mukbang* (Food ads) and health | | √ | |
|  |  | Source text | According to eating videos related to healthy elements, people can learn a healthier way to cook or eat.  Watching eating videos related to healthy elements, the viewers can choose food and ingredients related to healthy eating. | | | |
|  |  | 3. | Impacts may *mukbang* (Food ads) have on eating habits or eating behaviors | | √ | |
|  |  | Source text | Watching mukbang indirectly influenced the audience's view of food, resulting in its influence on the concept of healthy eating. By watching various types of mukbang, the viewers were more concentrate on food choice, food price, cooking method, cultural influence, and weight management, and these were thought to be related to healthy eating. | | | |
|  |  | 4. | The design, development, or usability of *mukbang* (Food ads) watching interventions to demonstrate the usefulness of *mukbang* in the health field | | | NA |
|  |  | Source text | NA | | | |
|  |  | 5. | Evaluation of the health effects of various types of *mukbang* (Food ads) | | | NA |
|  |  | Source text | NA | | | |
|  |  | 6. | The challenges and barriers of integrating *mukbang* (Food ads) videos into clinical practice | | | NA |
|  |  | Source text | NA | | | |
|  |  | 7. | The advantages and/or disadvantages of *mukbang* (Food ads) watching | | | √ |
|  |  | Source text | What matters is whether the content is healthy or what kind of healthy eating message is conveyed.  Expensive healthy food and unhealthy environment in the mukbang videos may cause unhealthy eating behaviors, or make viewers think that it is difficult to keep a healthy eating habit (or just keep in health).  Disadvantage: ①Resources and environment prevented people from eating healthily.  ②Healthy food is much more expensive than unhealthy food such as fast food. | | | |
|  | Research method/Tools | A qualitative study with text mining techniques | | | | |
|  | Characteristics of *mukbang* | In this study, the types of mukbang comprise cooking show, health challenge, vlog, etc., but they were all about healthy eating. | | | | |
|  | Health-related elements | Homemade food: people associated homemade food with healthy food.  Healthy environment: college students tend to prepare their food, and they avoid fast food sold on campus.  Weight management | | | | |
|  | Summary of key findings related to health | This study is not a direct investigation of the impact of healthy eating related YouTube videos on people's eating cognition and behavior, but through people's comments while watching these videos, in-depth analysis of people's real thoughts about healthy eating related videos, as well as their own views on healthy eating.  In conclusion, food ingredients, food price, food choice, food portion, well-being, cooking, and culture is the positive factors in promoting healthy eating choices after watching health related mukbang.  According to this study, people’s attitudes toward healthy eating are generally positive. A principal finding of this study is that people hold complex and multifaceted beliefs about healthy eating in the context of YouTube videos.  Another conclusion that can be drawn is that the more health-related information presented in mukbang, the more health-related knowledge viewers can gain from the video, and the viewers' health beliefs will also be correspondingly enhanced. | | | | |

*Food ads must include images of celebrities eating.

2. A STUDY ON POTENTIAL HEALTH ISSUES BEHIND THE POPULARITY OF MUKBANG IN CHINA

|  | Items | Data | | | | |
| --- | --- | --- | --- | --- | --- | --- |
| Inclusion criteria | Population/Main research object | This thesis examined popular mukbang videos on Kuaishou.The top three most-followed mukbangers were the focus of this research,Maomeimei, Langweixian, A Hao. | | | | |
|  | Concept (Tick)  Studies focusing on | 1. | *mukbang* watching (Food ads* | | √ | |
|  |  | 2. | the impact of *mukbang* (Food ads) on health | | √ | |
|  |  | 3. | the relationship between *mukbang* (Food ads) and health | |  | |
|  |  | 4. | the design, development, or usability of *mukbang* (Food ads) watching interventions to demonstrate the usefulness of *mukbang* in the health field | | NA | |
|  |  | 5. | evaluating the effectiveness of various types of *mukbang* (Food ads) for public health | | NA | |
|  |  | 6. | the challenges and barriers of integrating *mukbang* (Food ads) videos into clinical practice | | NA | |
|  |  | 7. | the advantages and/or disadvantages of *mukbang* (Food ads) watching | | √ | |
|  | Types of evidence source | master thesis | | | | |
| Evidence source details and characteristics | Citation details (eg, author/s, date, title, journal, volume, issue, pages) | Title | | A STUDY ON POTENTIAL HEALTH ISSUES BEHIND THE POPULARITY OF “MUKBANG” IN CHINA | | |
|  |  | Journal | | Middle Tennessee State University ProQuest Dissertations Publishing | | |
|  |  | Author/s | | Wang, Shan; | | |
|  |  | Date | | 2020-01-01 | | |
|  | Main content of the evidence | Mukbang, in which people broadcast themselves eating large quantities, started to enter the Chinese market around 2014 and gradually became popular. In this thesis, by analyzing the encodings of mukbang videos on Kuaishou, the shared themes across videos were discovered. Also, the decodings of mukbang videos by viewers were examined so as to figure out their different attitudes and emotions toward mukbang videos and mukbangers. Findings suggested that mukbang videos and its viewers have built up a complex relationship. Pursuing the thrill of hunger, vicarious eating, emotional establishment, and relieving anorexia were found as main reasons the viewers choose to watch mukbang videos. However, some viewers also expressed their shock, even disbelief on unusual food intake. Others expressed their desires on owning a slim figure that never becoming overweight even overeating. This thesis also argued that mukbang has indeed caused a certain harmful impact on the perception of eating norms and eating behaviors of some viewers. | | | | |
|  | Participant details (eg, age/sex and number) | Age | | NA | | |
|  |  | Sex | | Both | | |
|  |  | Final enrollment | | The top three most-followed mukbangers were the focus of this research,Maomeimei, Langweixian, A Hao. | | |
| Details/results extracted from source of evidence | Review question addressed  (Tick) | 1. | The health-related elements in *mukbang* (Food ads) | | √ | |
|  |  | Source text | Eating habit | | | |
|  |  | 2. | The relationship between *mukbang* (Food ads) and health | | NA | |
|  |  | Source text | NA | | | |
|  |  | 3. | Impacts may *mukbang* (Food ads) have on eating habits or eating behaviors | | √ | |
|  |  | Source text | The encoding of diet in mukbang videos has a certain temptation on the viewer, affecting the viewer’s perception and behavior of eating habits in a subtle way.  For example, the three selected mukbangers eat a lot of food at a time. This would convey to the audience an idea that excessive eat is allowed. Similarly, mukbanger’s way of eating also conveys an unhealthy concept to the viewer. For example, A Hao usually challenges himself to eat a lot of food. This kind of challenging behavior could arouse the competition psychology of the audience, meaning they want to imitate A Hao to do challenge style eating, so as to satisfy their desire to conquer. When Maomeimei was eating, her joyful expression and body languages left room for viewers to be curious and imagine the taste of food, which stimulated their appetite. As Spence et al. (2019) has pointed out that eating norms perceived by the audience will be affected by mukbang: audience would more easily eat more than usual, and even imitate mukbangers’ way of eating. In addition, in the choice of food, mukbanger tends to choose high-calorie and rich-sugar meals, such as, meats, desserts, sodas, etc., but with no vegetables on the side.  This would convey and enhance the idea of inadequate and imbalanced diets. Similarly, Hong and Park (2018) claim that viewers’ food choices can be affected by mukbang, tending to consume more high-calorie and oily foods. By constantly promoting this unhealthy diet as well as intentionally or unintentionally promoting the way mukbanger eat, viewers may ignore the existing concept of healthy eating so as to imitate the new dietary habits that full of novelty and pleasure. From the comments of the viewer, similar patterns were observed. Some viewers expressed their improved appetite and hoped to eat as much as mukbanger. Even some viewers have begun to imitate the mukbangers’ eating behavior and practice to be a mukbanger. It can be seen that mukbang has indeed caused a certain adverse effect on the eating habits of some viewers. | | | |
|  |  | 4. | The design, development, or usability of *mukbang* (Food ads) watching interventions to demonstrate the usefulness of *mukbang* in the health field | | | NA |
|  |  | Source text | NA | | | |
|  |  | 5. | Evaluation of the health effects of various types of *mukbang* (Food ads) | | | NA |
|  |  | Source text | NA | | | |
|  |  | 6. | The challenges and barriers of integrating *mukbang* (Food ads) videos into clinical practice | | | NA |
|  |  | Source text | NA | | | |
|  |  | 7. | The advantages and/or disadvantages of *mukbang* | | | √ |
|  |  | Source text | Pereira et al., 2019; Choi, 2015); others consider mukbang as “vicarious diets,” which means they regard mukbanger’s eating behavior as their own, and thus get a sense of satisfaction so as to dispel loneliness (Hakimey & Yazdanifard, 2015; Choe, 2019; Gillespie, 2019; Bruno & Chung, 2017); and some have established an emotional association with mukbangers. By watching mukbang live broadcasts or mukbang videos regularly, the viewer gains a sense of companionship and intimacy (Hakimey & Yazdanifard, 2015; Hong & Park, 2018; Choe, 2019; Donnar, 2017; Spence et al., 2019; Hakimey & Yazdanifard, 2015). | | | |
|  | Research method/Tools | The texts of mukbang videos, the follow up comments, and the intersection between mukbangers and viewers were analyzed (per the procedure given later) to explore the relationship between mukbang videos and viewers’ perception of food and health. | | | | |
|  | Characteristics of mukbang | This thesis examined popular mukbang videos on Kuaishou. This platform has two main formats: short previously recorded video posts and live broadcasts. Only focuses on pre-recorded, since they likely have higher viewership because compared to live broadcasting, short videos have less time limit and can be watched at any time. | | | | |
|  | Health-related elements | (4)As some scholars point out that distribution of food-related content through the media will encourage overeating (Bodenlos & Wormuth,2013; Boyland et al., 2011;Halford JC, 2008). Therefore, the actions of mukbangers in their video may affect the audience’s perception and behavior in a subtle way, making them believe that excessive diet is normal, and then begin to imitate the dietary habits of mukbangers. | | | | |
|  | Summary of key findings related to health | （1）examined the impact of mukbangers on the health and diet habits of their viewers.  （2）Opponents, mainly from the health point of view, believe that mukbang is not conducive to health, and does not conform to the laws of nature, as well as question the authenticity of mukbang. This thesis also argues that mukbang videos can affect or change some viewers’ eating habits and healthy eating concepts to a certain extent.  (5) Donnar (2017) believes that the imbalance of food and hunger may further exacerbated some social problems, like eating disorders, obesity, and other health issues | | | | |

*Food ads must include images of celebrities eating.

3. The popularity of eating broadcast Content analysis of mukbang YouTube videos, media coverage, and the health impact of mukbang on public

|  | Items | Data | | | | |
| --- | --- | --- | --- | --- | --- | --- |
| Inclusion criteria | Population/Main research object | This study aimed to analyze the content of mukbang YouTube videos, as well as news articles related to mukbang and the association between watching mukbang videos and health habits.  To provide useful information on the content of mukbang and generate information for predicting the popular impact of watching mukbang videos, and to investigated the effect of watching mukbang videos on health habits which could reflect the public perceptions. | | | | |
|  | Concept (Tick)  Studies focusing on | 1. | *mukbang* watching (Food ads*) | | √ | |
|  |  | 2. | the impact of *mukbang* (Food ads) on health | | √ | |
|  |  | 3. | the relationship between *mukbang* (Food ads) and health | | √ | |
|  |  | 4. | the design, development, or usability of *mukbang* (Food ads) watching interventions to demonstrate the usefulness of *mukbang* in the health field | | NA | |
|  |  | 5. | evaluating the effectiveness of various types of *mukbang* (Food ads) for public health | |  | |
|  |  | 6. | the challenges and barriers of integrating *mukbang* (Food ads) videos into clinical practice | | NA | |
|  |  | 7. | the advantages and/or disadvantages of *mukbang* (Food ads) watching | | √ | |
|  | Types of evidence source | Content analysis Qualitative study | | | | |
| Evidence source details and characteristics | Citation details (eg, author/s, date, title, journal, volume, issue, pages) | Title | | The popularity of eating broadcast: Content analysis of "mukbang" YouTube videos, media coverage, and the health impact of "mukbang" on public | | |
|  |  | Journal | | Health Informatics Journal | | |
|  |  | Author/s | | EunKyo Kang, Jihye Lee, Kyae Hyung Kim, Young Ho Yun | | |
|  |  | Date | | 2020-09 | | |
|  | Main content of the evidence | This study analyzed 5952 YouTube mukbang videos, 5265 news articles, and a survey of 1200 people in Korea. In this study, we confirmed that the provocative content of mukbang YouTube videos, such as overeating, was related to video popularity (p < 0.001). In addition, more exposure to mukbang was associated with greater effects on dietary health due to mukbang (p < 0.001). The prevalence of news articles on the negative effects of mukbang showed an increasing trend over time, while the articles on “Mukbang is funny” were most common in all the years evaluated. To cope with public health problems such as obesity, it will be necessary to continue to investigate the content and effects of mukbang on public health. | | | | |
|  | Participant details (eg, age/sex and number) | Age | | NA | | |
|  |  | Sex | | NA | | |
|  |  | Final enrollment | | YouTube videos: 52 YouTube videos with 10,000 or more views in the top 1000 pages were analyzed.  News articles: from 10 online newspapers over a 6-year period, a final total of 5265 articles were included in the analysis. | | |
| Details/results extracted from source of evidence | Review question addressed  (Tick) | 1. | The health-related elements in *mukbang* (Food ads) | | √ | |
|  |  | Source text | Overeating, eating quickly within the time limit, eating extremely spicy or irritating food, Eating non-hygienic or dangerous food.  About 83.5 percent of the videos presented overeating, and 1.3 percent of videos showed the creator eating quickly within a time limit. About 5.6 percent of the mukbang videos contained the content of eating extremely spicy or irritating food, and 0.6 percent of videos showed eating non-hygienic or dangerous food.  15.7 percent show the creator eating fast food or junk food. | | | |
|  |  | 2. | The relationship between *mukbang* (Food ads) and health | | √ | |
|  |  | Source text | Topics of news articles have covered the disadvantages of mukbang in promoting overeating and obesity and the effects on eating habits. | | | |
|  |  | 3. | Impacts may *mukbang* (Food ads) have on eating habits or eating behaviors | | √ | |
|  |  | Source text | 1.3 percent of the respondents said that watching mukbang had a strong influence on their health habits; in contrast, 32.6 percent of the people who always watch mukbang indicated a strong influence on their health habits.  Mukbang videos have substantial content showing harmful eating habits, and watching mukbang is negatively related to healthy eating habits. | | | |
|  |  | 4. | The design, development, or usability of *mukbang* (Food ads) watching interventions to demonstrate the usefulness of *mukbang* in the health field | | | NA |
|  |  | Source text | NA | | | |
|  |  | 5. | Evaluation of the health effects of various types of *mukbang* (Food ads) | | | NA |
|  |  | Source text | NA | | | |
|  |  | 6. | The challenges and barriers of integrating *mukbang* (Food ads) videos into clinical practice | | | NA |
|  |  | Source text | NA | | | |
|  |  | 7. | The advantages and/or disadvantages of *mukbang* (Food ads) watching | | | √ |
|  |  | Source text | Advantages: some people experience surrogate satisfaction while watching mukbang YouTube, and others feel the impulse to eat food while watching mukbang videos. This may indicate that watching mukbang can help to increase appetite.  Disadvantages: an increased attentional bias to food cues may contribute to overeating and weight gain. | | | |
|  | Research method/Tools | Using the latent Dirichlet allocation (LDA) as a topic modeling method, a model with a three levels hierarchical Bayesian model. | | | | |
|  | Characteristics of *mukbang* | Overeating, eating quickly within the time limit, Eating extremely spicy or irritating food, Eating non-hygienic or dangerous food. | | | | |
|  | Health-related elements | Overeating, obesity. | | | | |
|  | Summary of key findings related to health | Similar to previous understanding that the creators of mukbang videos are predominantly eating unhealthy food or overeat, it was confirmed that many mukbang creators showed overeating, as well as other unhealthy eating behaviors. The fact that mukbang YouTube videos that showed overeating or unhealthy eating habits had a higher number of views that this stimulating content is used as a means to worsen their health and eating habits. | | | | |

*Food ads must include images of celebrities eating.

4. An analysis of mukbang watching and watching behaviors of restricted eating groups Based on vicarious gratification theory

|  | Items | Data | | | | |
| --- | --- | --- | --- | --- | --- | --- |
| Inclusion criteria | Population/Main research object | NA | | | | |
|  | Concept (Tick)  Studies focusing on | 1. | *mukbang* watching (Food ads*) | | NA | |
|  |  | 2. | the impact of *mukbang* (Food ads) on health | | √ | |
|  |  | 3. | the relationship between *mukbang* (Food ads) and health | | √ | |
|  |  | 4. | the design, development, or usability of *mukbang* (Food ads) watching interventions to demonstrate the usefulness of *mukbang* in the health field | | NA | |
|  |  | 5. | evaluating the effectiveness of various types of *mukbang* (Food ads) for public health | | NA | |
|  |  | 6. | the challenges and barriers of integrating *mukbang* (Food ads) videos into clinical practice | | NA | |
|  |  | 7. | the advantages and/or disadvantages of *mukbang* (Food ads) watching | | √ | |
|  | Types of evidence source | Commentary | | | | |
| Evidence source details and characteristics | Citation details (eg, author/s, date, title, journal, volume, issue, pages) | Title | | An analysis of mukbang watching and watching behaviors of restricted eating groups: Based on vicarious gratification theory | | |
|  |  | Journal | | RADIO & TV JOURNAL | | |
|  |  | Author/s | | Mengting Pan | | |
|  |  | Date | | 2020 | | |
|  | Main content of the evidence | This paper adopts Freud's alternative satisfaction theory to explain the different behavioral characteristics of restrictive eating groups watching mukbang. | | | | |
|  | Participant details (eg, age/sex and number) | Age | | NA | | |
|  |  | Sex | | NA | | |
|  |  | Final enrollment | | NA | | |
| Details/results extracted from source of evidence | Review question addressed  (Tick) | 1. | The health-related elements in *mukbang* (Food ads) | | NA | |
|  |  | Source text | NA | | | |
|  |  | 2. | The relationship between *mukbang* (Food ads) and health mukbang | | NA | |
|  |  | Source text | NA | | | |
|  |  | 3. | Impacts may *mukbang* (Food ads) have on eating habits or eating behaviors | | √ | |
|  |  | Source text | 1) In addition to satisfying the audience's curiosity and emotional comfort, as a kind of food reality TV program, mukbang can also alleviate the food cravings and anxiety of restrictive eating groups for the purpose of dieting.  2) Viewers who eat very little per day are more likely to prefer the Big Eater Mukbang videos, which were rich in food variety. Individuals who don’t eat lunch or only eat one meal a day are much more likely to crave food than those who eat more regularly and at a mildly restricted diet. As a result, these individuals who are on a very strict diet have a stronger desire for alternative gratification by watching mukbang, which leads them to prefer Big Eater Mukbang videos that feature large amounts of food at one time and a wide variety of food items.  3) Restrictive eating groups watch mukbang to alleviate their appetite and regulate their self-suppressed psyche, and the resulting alternative satisfaction is a sense of spiritual and psychological satiation rather than physiological satiety, which is essentially a psychological defense mechanism motivated by self-protection. However, this psychological defense mechanism is self-deceptive in nature and is designed to disguise the true motives of the self as well as to act on the things and cravings that trigger the individual's anxiety.  4) The suppression of hunger and the increased appetite that accompanies weight loss in the restrictive eating group also prompts a frequent desire to eat, but they force themselves to repress the hunger that grows within them, yet this simultaneously exacerbates their overeating behavior. At the same time, the longer the suppression lasts and the stricter the control of one's diet, the greater the likelihood of subsequent revenge eating and triggering erroneous weight loss behaviors, resulting in a vicious cycle. | | | |
|  |  | 4. | The design, development, or usability of *mukbang* (Food ads) watching interventions to demonstrate the usefulness of *mukbang* in the health field | | | NA |
|  |  | Source text | NA | | | |
|  |  | 5. | Evaluation of the health effects of various types of *mukbang* (Food ads) | | | NA |
|  |  | Source text | NA | | | |
|  |  | 6. | The challenges and barriers of integrating *mukbang* (Food ads) videos into clinical practice | | | NA |
|  |  | Source text | NA | | | |
|  |  | 7. | The advantages and/or disadvantages of *mukbang* (Food ads) watching | | | √ |
|  |  | Source text | 1) As the filters, comments and expressions of the hosts give the viewers unlimited space for fantasy and imagination, it will make the viewers have great expectations for the food, so when they actually taste the food, it will create a sense of discrepancy and dissatisfaction.  2) An individual's appetite in reality can have an effect on mukbang viewing. Viewers who watch mukbangs for reasons of seeking alternative gratification are more likely to eat emotionally and overeat due to the gap between fantasy and reality. For restrictive eaters who have suppressed their appetite for a long period of time, the self-anxiety of having an uncontrollable appetite will cause them to eat a large amount of food at one time to relieve their boredom. | | | |
|  | Research method/Tools | Text analysis | | | | |
|  | Characteristics of *mukbang* | NA | | | | |
|  | Health-related elements | NA | | | | |
|  | Summary of key findings related to health | According to this study, mukbang may help weight loss, but relying too much on mukbang for losing weight can lead to the wrong behaviors, which can harm the viewers’ health. People who take an unhealthy approach to weight control and diet restriction would rely more on mukbang for vicarious satisfaction, which is actually an unhealthy way to use mukbang and is more likely to lead to emotional eating and overeating due to the gap between illusion and reality. | | | | |

*Food ads must include images of celebrities eating.

5. What satisfaction can you get from watching others eat

|  | Items | Data | | | | |
| --- | --- | --- | --- | --- | --- | --- |
| Inclusion criteria | Population/Main research object | The respondents in this study were viewers who had watched mukbang videos on Bilibili, and a total of 24 respondents were recruited, of which 8 were face-to-face interviewees and 16 were online interviewees. | | | | |
|  | Concept (Tick)  Studies focusing on | 1. | mukbang watching (Food ads*) | | √ | |
|  |  | 2. | the impact of mukbang (Food ads) on health | | √ | |
|  |  | 3. | the relationship between mukbang (Food ads) and health | | √ | |
|  |  | 4. | the design, development, or usability of mukbang (Food ads) watching interventions to demonstrate the usefulness of mukbang in the health field | | NA | |
|  |  | 5. | evaluating the effectiveness of various types of mukbang (Food ads) for public health | | NA | |
|  |  | 6. | the challenges and barriers of integrating mukbang (Food ads) videos into clinical practice | | NA | |
|  |  | 7. | the advantages and/or disadvantages of mukbang (Food ads) watching | | √ | |
|  | Types of evidence source | Qualitative study | | | | |
| Evidence source details and characteristics | Citation details (eg, author/s, date, title, journal, volume, issue, pages) | Title | | What satisfaction can you get from watching others eat? -- A qualitative study based on the audience of Bilibili | | |
|  |  | Journal | | Journal of News Research | | |
|  |  | Author/s | | Ludan Zhang, Li Cui | | |
|  |  | Date | | 2020.3 | | |
|  | Main content of the evidence | Some female viewers will refrain from eating because of the pursuit of a perfect body; some viewers will be filled with loneliness and stress because of the pursuit of higher education and more efficient work; and mukbang viewers will gather in the circle of the same people, seeking happiness in homogenized content. However, the problems cannot be really solved by watching mukbang, such as the ambivalence of some audiences about "to eat or not to eat" behind the vicarious satisfaction; the dissatisfaction with their body image behind the satisfaction of curiosity; the loneliness and stress in real life behind the virtual companionship; the entertainment and recreation behind the entertainment and recreation; and the lack of a sense of satisfaction in real life. Behind the satisfaction of curiosity, it also reflects the dissatisfaction of some audiences with their body image; behind the virtual companionship, it reflects the real-life loneliness and pressure of some recipients; behind the pleasure of entertainment, it is the attention attracted by the homogenized and superficial content of some recipients. It is also found that some audiences watch mukbang without any purpose, but they can also get pleasure and satisfaction from the videos. | | | | |
|  | Participant details (eg, age/sex and number) | Age | | NA | | |
|  |  | Sex | | NA | | |
|  |  | Final enrollment | | Respondents were viewers who had watched the recorded video of mukbang on Bilibili, and a total of 24 respondents were recruited, 19 of whom were found through their circle of friends and 5 through Bilibili. | | |
| Details/results extracted from source of evidence | Review question addressed  (Tick) | 1. | The health-related elements in mukbang (Food ads) | | √ | |
|  |  | Source text | Anchor's good figure contrasts with the amount of food consumption - Body Anxiety, Binge Eating | | | |
|  |  | 2. | The relationship between mukbang (Food ads) and health | | √ | |
|  |  | Source text | Mukbang is used as a substitute for real eating for aesthetic and health reasons, and alternative gratification is obtained by watching the mukbang.  Mukbang can have a soothing effect and its unique sound can be pleasing to some viewers. | | | |
|  |  | 3. | Impacts may mukbang (Food ads) have on eating habits or eating behaviors | | √ | |
|  |  | Source text | Most of the respondents had a great appetite because of the food presented in the video, and the type, color, and shape of the food were the key factors to arouse the audience's visual satisfaction. Nearly half of the respondents reported that they gained vicarious satisfaction from watching the mukbang and managed to restrain their appetite and reduce the number of times they ate in real life, especially high-calorie, high-fat foods. Mukbang videos could reduce the likelihood that viewers eat unhealthy foods. | | | |
|  |  | 4. | The design, development, or usability of mukbang (Food ads) watching interventions to demonstrate the usefulness of mukbang in the health field | | | NA |
|  |  | Source text | NA | | | |
|  |  | 5. | Evaluation of the health effects of various types of mukbang (Food ads) | | | NA |
|  |  | Source text | NA | | | |
|  |  | 6. | The challenges and barriers of integrating mukbang (Food ads) videos into clinical practice | | | NA |
|  |  | Source text | NA | | | |
|  |  | 7. | The advantages and/or disadvantages of mukbang (Food ads) watching | | | √ |
|  |  | Source text | Disadvantages: The contrast between the mukbang anchor's slim figure and his much higher-than-normal food intake has the potential to distort young people's view of health, negatively affecting the formation of proper health concepts among young people.  Advantages: Mukbang is able to provide virtual companionship to alleviate the viewer's sense of isolation and make them feel relaxed and happy. | | | |
|  | Research method/Tools | This study used in-depth interviews to understand the motivations of eating podcast audiences to use and the satisfaction they receive. | | | | |
|  | Characteristics of mukbang | Mukbang is a mix of the Korean words for "eating" and "broadcasting," which refers to people sitting in front of a webcam at home and broadcasting their meals live to the internet. | | | | |
|  | Health-related elements | High-calorie, high-fat foods, restrained eating; soothing moods, easing loneliness | | | | |
|  | Summary of key findings related to health | Most of the respondents would increase their appetite due to the presentation of food in the videos, with the type, color, and form of food being key factors in eliciting visual satisfaction from the audience.  Nearly half of the respondents reported that they gained vicarious satisfaction from watching mukbang and managed to restrain their appetite and reduce the number of times they ate in real life, especially high-calorie and high-fat foods. --The -mukbang video reduces the likelihood that the viewer will eat unhealthy foods.  For aesthetic and health reasons, mukbangs are used as a substitute for real eating, and alternative gratification is obtained by watching mukbangs.  Mukbang can have a soothing effect and its unique sound can be pleasurable for some viewers. | | | | |

*Food ads must include images of celebrities eating.

6. The reflect on the negative impact of “Mukbang Fever” on teenagers

|  | Items | Data | | | | |
| --- | --- | --- | --- | --- | --- | --- |
| Inclusion criteria | Population/Main research object | NA | | | | |
|  | Concept (Tick)  Studies focusing on | 1. | *mukbang* watching (Food ads*) | | NA | |
|  |  | 2. | the impact of *mukbang* (Food ads) on health | | NA | |
|  |  | 3. | the relationship between *mukbang* (Food ads) and health | | NA | |
|  |  | 4. | the design, development, or usability of *mukbang* (Food ads) watching interventions to demonstrate the usefulness of *mukbang* in the health field | | NA | |
|  |  | 5. | evaluating the effectiveness of various types of *mukbang* (Food ads) for public health | | NA | |
|  |  | 6. | the challenges and barriers of integrating *mukbang* (Food ads) videos into clinical practice | | NA | |
|  |  | 7. | the advantages and/or disadvantages of *mukbang* (Food ads) watching | | √ | |
|  | Types of evidence source | Commentary | | | | |
| Evidence source details and characteristics | Citation details (eg, author/s, date, title, journal, volume, issue, pages) | Title | | The reflect on the negative impact of “Mukbang Fever” on teenagers | | |
|  |  | Journal | | Young Society | | |
|  |  | Author/s | | Wei Jiangping | | |
|  |  | Date | | 2018-12 | | |
|  | Main content of the evidence | Originating from South Korea, mukbang has rapidly become a craze in China in recent years. However, in this low-threshold, popularized, entertaining and vulgarized craze, we see that it has brought negative impacts on the growth of young people, such as the distortion of health concepts, the erosion of social morale, and the deviation of values. In this article, the author analyzes the negative impact of "Mukbang Fever" on the youth and the inspiration behind this phenomenon. | | | | |
|  | Participant details (eg, age/sex and number) | Age | | NA | | |
|  |  | Sex | | NA | | |
|  |  | Final enrollment | | NA | | |
| Details/results extracted from source of evidence | Review question addressed  (Tick) | 1. | The health-related elements in *mukbang* (Food ads) | | NA | |
|  |  | Source text | NA | | | |
|  |  | 2. | The relationship between *mukbang* (Food ads) and health | | NA | |
|  |  | Source text | NA | | | |
|  |  | 3. | Impacts may *mukbang* (Food ads) have on eating habits or eating behaviors | | NA | |
|  |  | Source text | NA | | | |
|  |  | 4. | The design, development, or usability of *mukbang* (Food ads) watching interventions to demonstrate the usefulness of *mukbang* in the health field | | | NA |
|  |  | Source text | NA | | | |
|  |  | 5. | Evaluation of the health effects of various types of *mukbang* (Food ads) | | | NA |
|  |  | Source text | NA | | | |
|  |  | 6. | The challenges and barriers of integrating *mukbang* (Food ads) videos into clinical practice | | | NA |
|  |  | Source text | NA | | | |
|  |  | 7. | The advantages and/or disadvantages of *mukbang* (Food ads) watching | | | √ |
|  |  | Source text | 1) Promote the formation of distorted notions of health. In the adult mukbang video, most of the anchors have a good figure, beautiful face, but they can tolerant under the huge food, body and appetite between the strong contrast attracted the envy of teenagers, adult anchors to convey to the teenagers can eat not fat signals, attracting teenagers’ blind worship and follow the trend, however, behind this visual contrast, is actually the anchor's hidden secret - emetic. Under the distorted view of health, there are teenagers' swollen lips and obese bodies, and even the more "awesome" the food is, the more popular it is, and the more it can become a way for teenagers to seek knowledge, make friends, and achieve social acceptance.  2) Some mukbang videos have vulgar performances, which directly affects the performance of teenagers, who are likely to bring this social crooked wind into the campus and into the class. Teenagers are in the confused stage of self-knowledge, self-control ability is poor, if a large amount of energy and time into the network live broadcast, will directly affect the study or even waste learning.  3) The ambiguous language and behavior of the anchor in mukbang has a huge impact on the teenagers who are establishing moral concepts and correct values. In the actual eating video, the strange content of mukbang seriously affects the study life, eating behavior and growth of teenagers. | | | |
|  | Research method/Tools | NA | | | | |
|  | Characteristics of *mukbang* | Exotic content and lots of delicious but unhealthy, high-calorie food | | | | |
|  | Health-related elements | Distorted health concepts | | | | |
|  | Summary of key findings related to health | Mukbang can distort adolescents’ concept of health. In adult mukbang, most of the anchors have slim bodies and beautiful faces, but they can "tolerate" huge food with their small bodies. The strong contrast between body and appetite attracts the envy of teenagers. Adult anchors convey the signal that they can eat and not be fat to teenagers, which attracts their blind worship and follow the trend. This can impair teenagers' eating behaviors and habits, leading to overeating. | | | | |

*Food ads must include images of celebrities eating.

7. The reason of “Mukbang Heat” phenomenon from the perspective of communication science

|  | Items | Data | | | | |
| --- | --- | --- | --- | --- | --- | --- |
| Inclusion criteria | Population/Main research object | NA | | | | |
|  | Concept (Tick)  Studies focusing on | 1. | mukbang watching (Food ads*) | | √ | |
|  |  | 2. | the impact of mukbang (Food ads) on health | | √ | |
|  |  | 3. | the relationship between mukbang (Food ads) and health | | √ | |
|  |  | 4. | the design, development, or usability of mukbang (Food ads) watching interventions to demonstrate the usefulness of mukbang in the health field | | NA | |
|  |  | 5. | evaluating the effectiveness of various types of mukbang (Food ads) for public health | | NA | |
|  |  | 6. | the challenges and barriers of integrating mukbang (Food ads) videos into clinical practice | | NA | |
|  |  | 7. | the advantages and/or disadvantages of mukbang (Food ads) watching | | NA | |
|  | Types of evidence source | Commentary | | | | |
| Evidence source details and characteristics | Citation details (eg, author/s, date, title, journal, volume, issue, pages) | Title | | The reason of “Mukbang Heat” phenomenon from the perspective of communication science | | |
|  |  | Journal | | West China Broadcast TV | | |
|  |  | Author/s | | Menghuan Wu | | |
|  |  | Date | | 2019 | | |
|  | Main content of the evidence | The purpose of this study is to investigate the reasons for the emergence of the "Mukbang Fever" phenomenon and how it is spread. The study firstly classifies the existing popular mukbangs according to the appearance of the hosts and the different ways of presenting the food content, and secondly, analyzes the reasons for the emergence of "Mukbang Fever" in the country. | | | | |
|  | Participant details (eg, age/sex and number) | Age | | NA | | |
|  |  | Sex | | NA | | |
|  |  | Final enrollment | | NA | | |
| Details/results extracted from source of evidence | Review question addressed  (Tick) | 1. | The health-related elements in mukbang (Food ads) | | √ | |
|  |  | Source text | Slim figure and amazing amount of food;  Eating extreme amounts of high-calorie food, portraying the image of not being able to eat fat and misleading the viewer. | | | |
|  |  | 2. | The relationship between mukbang (Food ads) and health | | √ | |
|  |  | Source text | 1) Watching other people eat can have a stress-relieving effect: the moment the anchor takes a big bite of the food, coupled with the auditory stimulation of the crisp chewing sound to the audience, can help the audience release stress.  2) Soothing the viewer's negative emotions: the anchors in mukbang soothe the viewer's lonely nowhere mind and help alleviate his or her loneliness.  3) Audiences look to mukbang for solace or joy as food itself has healing properties. | | | |
|  |  | 3. | Impacts may mukbang (Food ads) have on eating habits or eating behaviors | | NA | |
|  |  | Source text | NA | | | |
|  |  | 4. | The design, development, or usability of mukbang (Food ads) watching interventions to demonstrate the usefulness of mukbang in the health field | | | NA |
|  |  | Source text | NA | | | |
|  |  | 5. | Evaluation of the health effects of various types of mukbang (Food ads) | | | NA |
|  |  | Source text | NA | | | |
|  |  | 6. | The challenges and barriers of integrating mukbang (Food ads) videos into clinical practice | | | NA |
|  |  | Source text | NA | | | |
|  |  | 7. | The advantages and/or disadvantages of mukbang (Food ads) watching | | | NA |
|  |  | Source text | NA | | | |
|  | Research method/Tools | NA | | | | |
|  | Characteristics of mukbang | There are various types of mukbang, and this study categorizes the types of mukbang based on the appearance of the anchor, type of diet, and level of diet, respectively. | | | | |
|  | Health-related elements | Mukbang relieves stress, loneliness, and gives comfort to the viewer. It mainly describes the psychological benefits of watching mukbang videos for the viewers, which have a positive impact on their mental health. | | | | |
|  | Summary of key findings related to health | The content of mukbang tends to show the slim body of the anchors and the amazing amount of food they eat. The videos of the anchors eating extreme foods have spread unhealthy eating behaviors to the public. Studies have found that watching mukbang relieves stress and loneliness, soothes the mind and seeks solace and happiness. At the same time, this article also emphasized that the positive effect on psychology was based on the viewer's full respect for their body in real life. If the viewer blindly imitated the anchor's eating behaviors, it could only erase the function and significance of the mukbang in healing negative emotions and relieving pressure. | | | | |

*Food ads must include images of celebrities eating.

8. Mukbang- and Cookbang-watching status and dietary life

|  | Items | Data | | | | |
| --- | --- | --- | --- | --- | --- | --- |
| Inclusion criteria | Population/Main research object | This study was conducted from March to April 2019 on male and female students at a university in northern Gyeonggi, Korea. A total of 639 questionnaires were distributed and 621 were collected. | | | | |
|  | Concept (Tick)  Studies focusing on | 1. | *mukbang* watching (Food ads*) | | √ | |
|  |  | 2. | the impact of *mukbang* (Food ads) on health | | √ | |
|  |  | 3. | the relationship between *mukbang* (Food ads) and health | | NA | |
|  |  | 4. | the design, development, or usability of *mukbang* (Food ads) watching interventions to demonstrate the usefulness of *mukbang* in the health field | | NA | |
|  |  | 5. | evaluating the effectiveness of various types of *mukbang* (Food ads) for public health | | NA | |
|  |  | 6. | the challenges and barriers of integrating *mukbang* (Food ads) videos into clinical practice | | NA | |
|  |  | 7. | the advantages and/or disadvantages of *mukbang* (Food ads) watching | | √ | |
|  | Types of evidence source | cross-sectional study design | | | | |
| Evidence source details and characteristics | Citation details (eg, author/s, date, title, journal, volume, issue, pages) | Title | | Mukbang- and Cookbang-watching status and dietary life of university students who are not food and nutrition majors | | |
|  |  | Journal | | Nutrition Research and Practice | | |
|  |  | Author/s | | Sowon Yun, Hyunjoo Kang, Hongmie Lee | | |
|  |  | Date | | 2022-4-3 | | |
|  | Main content of the evidence | this study sought to compare the Mukbang- and Cookbang-watching status of university students with their dietary life. | | | | |
|  | Participant details (eg, age/sex and number) | Age | | Grade 1st-4th | | |
|  |  | Sex | | Both | | |
|  |  | Final enrollment | | 380 students who were not majoring in food and nutrition at a university in Gyeonggi, Korea. | | |
| Details/results extracted from source of evidence | Review question addressed  (Tick) | 1. | The health-related elements in *mukbang* (Food ads) | | √ | |
|  |  | Source text | dietary life，mukbang, cookbang-watching | | | |
|  |  | 2. | The relationship between *mukbang* (Food ads) and health | | NA | |
|  |  | Source text | NA | | | |
|  |  | 3. | Impacts may *mukbang* (Food ads) have on eating habits or eating behaviors | | √ | |
|  |  | 1. Source text | （1）In the case of Cookbang, participants in the FW group reported eating alone (P < 0.05) and cooking for themselves (P < 0.01) significantly more frequently than those in the MW and NW groups. Up to 50.0% of participants in the Cookbang FW group reported eating more than one meal per day alone, whereas these percentages were 34.9% in the MW group and 36.6% in the NW group. Also, up to 30.0% of participants in the Cookbang FW group reported cooking more than one meal per day for themselves, whereas these percentages were 13.2% in the MW group and 14.6% in the NW group.  （2）Tables 5 and 6 compares the participants' perceptions of how their diets were influenced by watching Mukbang or Cookbang. For Mukbang, 29.1% reported that watching Mukbang increased their intake through eating out and delivered and convenience foods, whereas only 3.2% reported that their viewership decreased it. In the FW and MW groups, 11.3% and 6.6%, respectively, reported that watching Mukbang worsened their diet, while 7.5% and 0%, respectively, reported that viewership improved their diet (P < 0.05).  (3) Only one subject of Cookbang answered that viewership worsened their diet, whereas 35.0% of the FW group and 10.4% of the NW group reported that viewership improved their diet. Compared to MW group, the FW group had a significantly more favorable perception of how watching Cookbang affected their diet, since a greater proportion of this group reported that watching Cookbang improved their diet (P < 0.05). Up to 65.1% answered that watching Cookbang increased their desire to cook and 41.3% reported that it actually increased their frequency of cooking; there was no significant difference between the FW and MW groups in this regard. | | | |
|  |  | 4. | The design, development, or usability of *mukbang* (Food ads) watching interventions to demonstrate the usefulness of *mukbang* in the health field | | | NA |
|  |  | Source text | NA | | | |
|  |  | 5. | Evaluation of the health effects of various types of *mukbang* (Food ads) | | | NA |
|  |  | Source text | NA | | | |
|  |  | 6. | The challenges and barriers of integrating *mukbang* (Food ads) videos into clinical practice | | | NA |
|  |  | Source text | NA | | | |
|  |  | 7. | The advantages and/or disadvantages of *mukbang* (Food ads) watching | | | √ |
|  |  | Source text | 1. Disadvantages:   The most notable finding from this study is that frequent watching of Cookbang and Mukbang may have different associations with the diet of university students. Using 4 categories, we found evidence suggesting that excessive watching of Mukbang, but not Cookbang, may adversely influence the diet of young adults.  1)Firstly, frequent watchers of Mukbang scored their diet significantly worse than the less frequent watchers of this genre, whereas the means were not significantly different between groups according to their Cookbang-watching frequency.  2)Secondly, the students had different perceptions of how Mukbang- and Cookbang-watching affected their diet. More participants felt that their diets improved rather than worsened by watching Cookbang, with more dramatically in FW group, while in case of Mukbang, far more participants felt that it worsened their diet rather than improved it. A significant positive change was previously associated with the acquisition of dietary information from TV cooking programs among adults.  3)Thirdly, as much as two thirds of the frequent watchers of Cookbang answered that Cookbang-watching increased their desire to cook and 55% answered that they actually cooked more frequently thanks to their viewership, whereas Mukbang-watching seemed to be associated with rather undesirable diets, since the participants felt that Mukbang watching prompted them to eat more of less-desirable foods, such as through eating out and consuming convenient and delivered foods. This perception was significantly greater in frequent watchers than in moderate watchers.  4）In contrast, our participants felt that viewing Mukbang increased their eating of foods that were usually considered unhealthy, such as delivered foods. | | | |
|  | Research method/Tools | used a cross-sectional study design and a self-administered questionnaire | | | | |
|  | Characteristics of *mukbang* | Food-related programming is an important broadcasting trend in Korea. Two types of such programming are Mukbang and Cookbang. Mukbang refers to a live or recorded audiovisual broadcast in which a host eats large amounts or delicious-looking foods while interacting with their audience; this format is receiving sociocultural attention worldwide [1-3]. Cookbang goes beyond Mukbang, in which the performers first cook the food themselves, and then eat it [4]. These food-related programs offer viewers a sense of intimacy and comfort, but also provide simple “food porn”-level sensations and pleasures that can lead to excessive consumption and unhealthy eating [5]. | | | | |
|  | Health-related elements | as the content available on mobile devices continues to increase at a rapid pace, college students’ dietary life. may become particularly vulnerable to the adverse effects of watching Mukbang and Cookbang. | | | | |
|  | Summary of key findings related to health | (1) In the case of Mukbang, but not Cookbang, the participants in the FW group scored their diet significantly worse than those in the NW group (P < 0.05). A greater proportion of participants felt that watching Cookbang improved their diets rather than worsened them (14.3% vs. 0.8%, respectively), while more participants said that watching Mukbang worsened their diets rather than improved them (8.1% vs. 2.4%, respectively). In both cases, greater differences were shown in the FW groups compared to the MW groups (P < 0.05 and P < 0.01 for Cookbang and Mukbang, respectively). Moreover, the participants answered that Mukbang-watching prompted them to eat more of less desirable food, such as through eating out and purchasing convenient and delivered foods, whereas Cookbang-watching made them want to cook more of their own food.  (2) Our results suggested that Korean university students who frequently watch Mukbang, but not Cookbang, may be a nutritionally vulnerable group that needs attention. | | | | |

*Food ads must include images of celebrities eating.

9. Research on audience psychology and profit model of mukbang from the perspective of communication

|  | Items | Data | | | | |
| --- | --- | --- | --- | --- | --- | --- |
| Inclusion criteria | Population/Main research object | NA | | | | |
|  | Concept (Tick)  Studies focusing on | 1. | *mukbang* watching (Food ads*) | | NA | |
|  |  | 2. | the impact of *mukbang* (Food ads) on health | | √ | |
|  |  | 3. | the relationship between *mukbang* (Food ads) and health | | NA | |
|  |  | 4. | the design, development, or usability of *mukbang* (Food ads) watching interventions to demonstrate the usefulness of *mukbang* in the health field | | NA | |
|  |  | 5. | evaluating the effectiveness of various types of *mukbang* (Food ads) for public health | | NA | |
|  |  | 6. | the challenges and barriers of integrating *mukbang* (Food ads) videos into clinical practice | | NA | |
|  |  | 7. | the advantages and/or disadvantages of *mukbang* (Food ads) watching | | √ | |
|  | Types of evidence source | Commentary | | | | |
| Evidence source details and characteristics | Citation details (eg, author/s, date, title, journal, volume, issue, pages) | Title | | Research on audience psychology and profit model of mukbang from the perspective of communication | | |
|  |  | Journal | | JOURNALISM COMMUNICATION | | |
|  |  | Author/s | | Fangxiu Liu | | |
|  |  | Date | | 2018 | | |
|  | Main content of the evidence | This paper takes the mukbang program as the main research object, summarizes the psychology of the audience who love the mukbang video through the collection of related video data, and makes an analysis of its profit mode. | | | | |
|  | Participant details (eg, age/sex and number) | Age | | NA | | |
|  |  | Sex | | NA | | |
|  |  | Final enrollment | | NA | | |
| Details/results extracted from source of evidence | Review question addressed  (Tick) | 1. | The health-related elements in *mukbang* (Food ads) | | √ | |
|  |  | Source text | audience psychology | | | |
|  |  | 2. | The relationship between *mukbang* (Food ads) and health | | NA | |
|  |  | Source text | NA | | | |
|  |  | 3. | Impacts may *mukbang* (Food ads) have on eating habits or eating behaviors | | NA | |
|  |  | Source text | NA | | | |
|  |  | 4. | The design, development, or usability of *mukbang* (Food ads) watching interventions to demonstrate the usefulness of *mukbang* in the health field | | | NA |
|  |  | Source text | NA | | | |
|  |  | 5. | Evaluation of the health effects of various types of *mukbang* (Food ads) | | | NA |
|  |  | Source text | NA | | | |
|  |  | 6. | The challenges and barriers of integrating *mukbang* (Food ads) videos into clinical practice | | | NA |
|  |  | Source text | NA | | | |
|  |  | 7. | The advantages and/or disadvantages of *mukbang* (Food ads) watching | | | √ |
|  |  | Source text | Advantages:  1) Relieving loneliness: Big Eater Mukbang's anchors are able to satisfy emotional needs by providing companionship to lonely individuals through a high level of chatting, bringing them closer to the audience, and freeing some of them from the feeling of loneliness. This mukbang video serves as a strange kind of "virtual dinner party", which is the best choice for people who eat alone to release their loneliness.  2) Stress Relief: In modern society, the pressure of competition has increased, and most young women, under the guidance of society's thinness as beauty, go on diets and suppress their appetite, making the pressure of life and work impossible to vent. Bing Eater Mukbang realizes the viewers to watch the anchor eating as if they were enjoying the food themselves through the mood shift effect, which can reasonably ventilate their pent-up appetites and stresses.  3) Increasing Appetite: The behind-the-scenes backdrops are clashing with many bright colors, giving viewers a sense of visual impact and whetting fans' appetites. | | | |
|  | Research method/Tools | NA | | | | |
|  | Characteristics of *mukbang* | NA | | | | |
|  | Health-related elements | NA | | | | |
|  | Summary of key findings related to health | This paper took mukbang as the main research object and summarized the psychology of viewers who love mukbang through the collection of relevant video materials. It has found that viewers watch mukbang mainly to help them release loneliness, get a sense of pleasure, relieve pressure and manage their body. In addition, mukbang also combined all the characteristics of audiovisual media to stimulate the audience's appetite through visual and auditory stimulation, which plays a role in promoting appetite. | | | | |

*Food ads must include images of celebrities eating.

10. Uses and gratifications of problematic mukbang watching - The role of eating and social gratification A pilot study

|  | Items | Data | | | | |
| --- | --- | --- | --- | --- | --- | --- |
| Inclusion criteria | Population/Main research object | This study focused only on eating and social motivations associated with mukbang watching, and the associations between eating gratification and social gratification with problematic mukbang watching. The present preliminary cross-sectional study aimed at providing empirical evidence for the relationship of eating and social gratifications with problematic mukbang watching. | | | | |
|  | Concept (Tick)  Studies focusing on | 1. | *mukbang* watching (Food ads*) | | √ | |
|  |  | 2. | the impact of *mukbang* (Food ads) on health | | √ | |
|  |  | 3. | the relationship between *mukbang* (Food ads) and health | | √ | |
|  |  | 4. | the design, development, or usability of *mukbang* (Food ads) watching interventions to demonstrate the usefulness of *mukbang* in the health field | | NA | |
|  |  | 5. | evaluating the effectiveness of various types of *mukbang* (Food ads) for public health | | √ | |
|  |  | 6. | the challenges and barriers of integrating *mukbang* (Food ads) videos into clinical practice | | NA | |
|  |  | 7. | the advantages and/or disadvantages of *mukbang* (Food ads) watching | | √ | |
|  | Types of evidence source | Cross-sectional questionnaire-based study | | | | |
| Evidence source details and characteristics | Citation details (eg, author/s, date, title, journal, volume, issue, pages) | Title | | Uses and gratifications of problematic mukbang watching - The role of eating and social gratification: A pilot study | | |
|  |  | Journal | | Journal of Psychiatric Research | | |
|  |  | Author/s | | Kircaburun Kagan | | |
|  |  | Date | | 2022-02 | | |
|  | Main content of the evidence | This study is one of the preliminary attempts to investigate problematic mukbang watching and its psychological and motivational correlates. It appears that satisfying real life eating needs by obtaining virtual eating gratification from mukbang watching could be a more serious risk factor for developing problematic mukbang watching and suffering unwanted negative consequences. | | | | |
|  | Participant details (eg, age/sex and number) | Age | | NA | | |
|  |  | Sex | | Both | | |
|  |  | Final enrollment | | A total of 170 students from Yaşar University who watched mukbang in the past year participated in the study. | | |
| Details/results extracted from source of evidence | Review question addressed  (Tick) | 1. | The health-related elements in *mukbang* (Food ads) | | NA | |
|  |  | Source text | NA | | | |
|  |  | 2. | The relationship between *mukbang* (Food ads) and health | | √ | |
|  |  | Source text | 1) Problematic mukbang watching can have negative consequences and/or correlates including disordered eating, distortion of eating and table manners, adolescent obesity, and adult overweight.  2) Watching mukbang for the purpose of obtaining virtual eating satisfaction (vicarious satisfaction) is more likely to lead to problematic mukbang watching.  3) Problematic mukbang watching could be an extension of food addiction.  3) Some food addicts might satisfy their real-life food cravings and eating needs via watching others eat, giving them the virtual pleasures of excessive food consumption and eating. | | | |
|  |  | 3. | Impacts may *mukbang* (Food ads) have on eating habits or eating behaviors | | √ | |
|  |  | Source text | 1) Eating gratification (but not social gratification) was positively associated with problematic mukbang watching. | | | |
|  |  | 4. | The design, development, or usability of *mukbang* (Food ads) watching interventions to demonstrate the usefulness of *mukbang* in the health field | | | NA |
|  |  | Source text | NA | | | |
|  |  | 5. | Evaluation of the health effects of various types of *mukbang* (Food ads) | | | √ |
|  |  | Source text | Different viewing motives may have a certain impact on mukbang watching, with the main purpose of vicarious satisfaction, and uncontrolled viewing is most likely related to problematic mukbang watching. | | | |
|  |  | 6. | The challenges and barriers of integrating *mukbang* (Food ads) videos into clinical practice | | | NA |
|  |  | Source text | NA | | | |
|  |  | 7. | The advantages and/or disadvantages of *mukbang* (Food ads) watching | | | √ |
|  |  | Source text | Advantages: watching mukbang can satisfy cravings for food that is forbidden while dieting, and curb hunger. | | | |
|  | Research method/Tools | 1) Mukbang Addiction Scale  2) Mukbang Gratification Scale | | | | |
|  | Characteristics of *mukbang* | Preblematic mukbang watching (watching mukbang for the following purposes or experiencing the following symptoms): mood modification *(i.e., excessive mukbang watching leading to specific changes in mood states)*, salience *(i.e., total preoccupation with mukbang watching)*, tolerance *(i.e., increasing amounts of time spent watching mukbang)*, withdrawal symptoms *(i.e., negative feelings and psychological symptoms such as irritability, anxiety when mukbang watching is restricted)*, conflict *(i.e., compromising occupation, education, and/or interpersonal relationships as a direct result of mukbang watching)*, and relapse *(i.e., returning to excessive mukbang watching after a period of abstinence)*.  Problematic mukbang watching can be conceptualized as being overly concerned about mukbang watching, to be driven by a strong motivation to watch mukbang, and to spend excessive time and effort for watching mukbang that it causes severe problems with an individual’s mental health and wellbeing. | | | | |
|  | Health-related elements | Food addiction  Obtaining virtual eating gratification  Mitigates loneliness and social isolation (mental health) | | | | |
|  | Summary of key findings related to health |  | | | | |

*Food ads must include images of celebrities eating.

11. The study of ASMR mukbang on network platforms

|  | Items | Data | | | | |
| --- | --- | --- | --- | --- | --- | --- |
| Inclusion criteria | Population/Main research object | NA | | | | |
|  | Concept (Tick)  Studies focusing on | 1. | *mukbang* watching (Food ads*) | | √ | |
|  |  | 2. | the impact of *mukbang* (Food ads) on health | | √ | |
|  |  | 3. | the relationship between *mukbang* (Food ads) and health | | NA | |
|  |  | 4. | the design, development, or usability of *mukbang* (Food ads) watching interventions to demonstrate the usefulness of *mukbang* in the health field | | NA | |
|  |  | 5. | evaluating the effectiveness of various types of *mukbang* (Food ads) for public health | | NA | |
|  |  | 6. | the challenges and barriers of integrating *mukbang* (Food ads) videos into clinical practice | | NA | |
|  |  | 7. | the advantages and/or disadvantages of *mukbang* (Food ads) watching | | √ | |
|  | Types of evidence source | Commentary | | | | |
| Evidence source details and characteristics | Citation details (eg, author/s, date, title, journal, volume, issue, pages) | Title | | The study of ASMR mukbang on network platforms | | |
|  |  | Journal | | Satellite TV & IP Multimedia | | |
|  |  | Author/s | | Xinge Zhong | | |
|  |  | Date | | 2021 | | |
|  | Main content of the evidence | ASMR Mukbang is one of the forms of mukbang, which mainly focuses on the prominent presentation of sound while utilizing the visual expression of the picture to make the viewers experience a stronger sense of being fully immersed in the virtual orgy brought about by the eating podcast video. Based mainly on the binge theory, this thesis conducts an in-depth analysis of the popularity of ASMR Mukbang, and finds that viewers choose to watch ASMR Mukbang because it can provide a sense of virtual companionship and detoxify self-pressure and anxiety; in addition to this, the viewers can also feel the deliberately amplified sound of chewing and swallowing in the ASMR Mukbang video and other In addition, viewers can indirectly satisfy their appetite by feeling the deliberately amplified auditory stimuli such as chewing and swallowing sounds in the ASMR Mukbang video and obtaining a sense of alternative satisfaction. | | | | |
|  | Participant details (eg, age/sex and number) | Age | | NA | | |
|  |  | Sex | | NA | | |
|  |  | Final enrollment | | NA | | |
| Details/results extracted from source of evidence | Review question addressed  (Tick) | 1. | The health-related elements in *mukbang* (Food ads) | |  | |
|  |  | Source text | Vicarious satisfaction | | | |
|  |  | 2. | The relationship between *mukbang* (Food ads) and health | | √ | |
|  |  | Source text | Mukbang is gradually characterized by pan-entertainment, and this pan-entertainment can lead to an imbalance in people's spiritual ecology. | | | |
|  |  | 3. | Impacts may *mukbang* (Food ads) have on eating habits or eating behaviors | | √ | |
|  |  | Source text | This type of ASMR Mukbang, through the visual presentation of images and the auditory transmission of sound, can realize the amplification of people's sense of taste, so that one's own sense of taste to get experiential satisfaction. Many dieters tend to watch mukbangs as if they have eaten, which is a kind of alternative satisfaction. (ASMR mukbang can amplify people's sense of taste through visual presentation of images and auditory transmission of sounds, thus making the viewers develop the bad habit of eating while watching videos and rely on watching mukbang to satisfy their own food cravings, thus achieving the goal of losing weight by eating less.) | | | |
|  |  | 4. | The design, development, or usability of *mukbang* (Food ads) watching interventions to demonstrate the usefulness of *mukbang* in the health field | | | NA |
|  |  | Source text | NA | | | |
|  |  | 5. | Evaluation of the health effects of various types of *mukbang* (Food ads) | | | NA |
|  |  | Source text | NA | | | |
|  |  | 6. | The challenges and barriers of integrating *mukbang* (Food ads) videos into clinical practice | | | NA |
|  |  | Source text | NA | | | |
|  |  | 7. | The advantages and/or disadvantages of *mukbang* (Food ads) watching | | | √ |
|  |  | Source text | 1) ASMR Mukbang maximizes people's sense of virtual companionship through the presentation of video images and the dissemination of sound, and makes up for people's anxiety caused by the "absence of the body" in the process of communication. ASMR Mukbang can alleviate the anxiety and loneliness of the youth group to a certain extent through the visual presentation of food and the 3D communication of sound, satisfy their sense of belonging, and give them a sense of social pleasure in the virtual space.  2) Self-stress and anxiety relief  ASMR mukbang can be a sleep aid for some people. When watching this type of mukbang, the sound transmission can relax the brain to a certain extent, making it easier for people to go to sleep, and to a certain extent, reducing the stress and anxiety in real life. By immersing themselves in the atmosphere created by the unique sound, people can temporarily detach themselves from real life and gain emotional fulfillment. Some people look for an outlet for their appetite when they watch it, and they can no longer suppress their appetite. Therefore, after watching the food broadcast, people will have a feeling as if they have eaten it themselves, which is in fact that people seem to have obtained a kind of alternative satisfaction of tasting the food, which is also a kind of self-relief means when people are bored. | | | |
|  | Research method/Tools | NA | | | | |
|  | Characteristics of *mukbang* | ASMR Mukbang is a form of eating podcasting. The full title of ASMR is Autonomous Sensory Meridian Response, which is a kind of passive sensory, which will make people have a kind of relaxation and reduce the feeling of stress. ASMR Mukbang is mainly through the visual presentation of the picture and the auditory presentation of the sound, which will make the person's scalp, back and other parts of the scalp to produce a tingling sensation. | | | | |
|  | Health-related elements | Self-stress and anxiety resolution | | | | |
|  | Summary of key findings related to health | Based on the carnival theory, this paper conducted an in-depth analysis of the popularity of ASMR Mukbang, and found that viewers choose to watch ASMR Mukbang because it could provide a sense of virtual companion and relieve their stress and anxiety. In addition to this, viewers can obtain a sense of substitutional satisfaction and indirectly satisfy their appetite by feeling the auditory stimuli that are deliberately amplified in the ASMR Mukbang video, such as chewing and swallowing sounds. | | | | |

*Food ads must include images of celebrities eating.

12. Digital Forms of Commensality in the 21st Century

|  | Items | Data | | | |
| --- | --- | --- | --- | --- | --- |
| Inclusion criteria | Population/Main research object | NA | | | |
|  | Concept (Tick)  Studies focusing on | 1. | *mukbang* watching (Food ads*) | | √ |
|  |  | 2. | the impact of *mukbang* (Food ads) on health | | √ |
|  |  | 3. | the relationship between *mukbang* (Food ads) and health | | NA |
|  |  | 4. | the design, development, or usability of *mukbang* (Food ads) watching interventions to demonstrate the usefulness of *mukbang* in the health field | | NA |
|  |  | 5. | evaluating the effectiveness of various types of *mukbang* (Food ads) for public health | | NA |
|  |  | 6. | the challenges and barriers of integrating *mukbang* (Food ads) videos into clinical practice | | NA |
|  |  | 7. | the advantages and/or disadvantages of *mukbang* (Food ads) watching | | √ |
|  | Types of evidence source | Scope review | | | |
| Evidence source details and characteristics | Citation details (eg, author/s, date, title, journal, volume, issue, pages) | Title | | Digital Forms of Commensality in the 21st Century:  A Scoping Review | |
|  |  | Journal | | International journal of Environmental research and public health | |
|  |  | Author/s | | Maína Ribeiro Pereira-Castro | |
|  |  | Date | | 2022 | |
|  | Main content of the evidence | A total of 104 publications that combined commensality and technology in all contexts were included. Most studies were qualitative; from the Design and Technology field; used social media and video platforms or prototypes/augmented reality gadgets; and used different terms to refer to digital forms of commensality, allowing the analysis of the construction of field definitions over time. The intersections with health were observed from impacts on family/community engagement, culinary skills development, and mental health and eating habits.  This paper indicates the consistent growth of these practices and recommends the development of future research for theoretically and longitudinally deeper evaluations of the impacts of these new ways of eating together, especially regarding their effects on human health. | | | |
|  | Participant details (eg, age/sex and number) | Age | | NA | |
|  |  | Sex | | NA | |
|  |  | Final enrollment | | A total of 104 publications that combined commensality and technology in all contexts were included.  Only studies published between January 2001 and December 2021 were included. Studies that were still being conducted were also included, with the objective of mapping the full academic scope of the technologically mediated forms of commensality. Studies could be quantitative, qualitative, mixed, or even opinion articles. Therefore, in addition to selecting scientific articles, the study selected dissertations and theses, conference proceedings, reviews, essays, and gray literature. The electronic databases PubMed, EBSCOHost, SCOPUS, Web of Science, PROQUEST, and Google Scholar were used to search for and identify studies, including publications written in Portuguese, English, Spanish, French, and Italian. The decision to use these languages was motivated by the understanding that (traditional) commensality is a concept that is historically and sociologically investigated in French and Italian studies and due to the interest in identifying possible studies (in Portuguese and Spanish) from Latin America since the researchers are Brazilian. | |
| Details/results extracted from source of evidence | Review question addressed  (Tick) | 1. | The health-related elements in *mukbang* (Food ads) | | √ |
|  |  | Source text | Extracted a theme related to health in the scope review | | |
|  |  | 2. | The relationship between *mukbang* (Food ads) and health | | NA |
|  |  | Source text | NA | | |
|  |  | 3. | Impacts may *mukbang* (Food ads) have on eating habits or eating behaviors | | √ |
|  |  | Source text | （1）Finally, categories were created to group studies on the impact of the use of digital technologies in the food context on mental health and on the eating habits of populations and individuals, in which, respectively, the results reported the advantages of the intersections between the digital world and food, such as reducing loneliness and improving eating habits, and the disadvantages, such as encouraging excessive eating and maintaining behaviors associated with eating disorders.  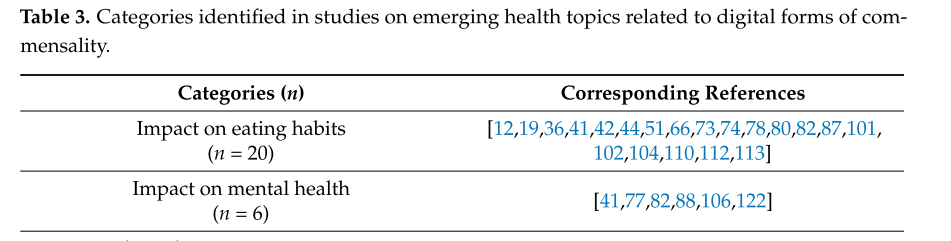 | | |
|  |  | 4. | The design, development, or usability of *mukbang* (Food ads) watching interventions to demonstrate the usefulness of *mukbang* in the health field | | NA |
|  |  | Source text | NA | | |
|  |  | 5. | Evaluation of the health effects of various types of *mukbang* (Food ads) | | NA |
|  |  | Source text | NA | | |
|  |  | 6. | The challenges and barriers of integrating *mukbang* (Food ads) videos into clinical practice | | NA |
|  |  | Source text | NA | | |
|  |  | 7. | The advantages and/or disadvantages of *mukbang* (Food ads) watching | | √ |
|  |  | Source text | （1）On the one hand, it is shown that eating together has a direct relationship with the improvement of health indicators, such as the promotion of healthy eating habits and impacts on mental health [126]. On the other hand, some evidence [127] and food-based dietary guidelines e.g., [128,129] indicate that eating in front of screens should be discouraged because of its negative effects. However, the studies identified in this article indicate and encourage initial discussions of this dichotomy for a theoretical deepening of the associations between health and the other different forms of commensality mediated by technologies.  (2) Based on what has already been studied about health and contemporary commensalities, it is possible to characterize an initial overview of their interactions. The Mukbang, described throughout the sample as the high consumption of ultra-processed foods by its presenters and the regulation of food consumption by its viewers, was sometimes deleterious by increasing the intake of foods high in salt, fat, sugars, and food additives and sometimes had a positive effect by including lonely individuals in communities [94]; in summary, the research showed the potential of a single practice to combine both benefits and harms to health. Nevertheless, considering the differences inherent to the very types of contemporary commensality practices explored in the review, it was observed that publications on mukbang more often indicated health risks, while studies that addressed the use of videoconferencing tools to connect distant diners mostly pointed to the potential of the practice to socially enrich the lives of its participants and to encourage them, with few mentions of negative aspects, such as increased homesickness and feelings of missing family or feelings of guilt for having moved to a location far from their loved ones [86]. | | |
|  | Research method/Tools | NA | | | |
|  | Characteristics of mukbang | six ways of eating in which social relationships were represented in the studies: (1) eating together remotely and solo-eating with technology; (2) family meals with children using an electronic device at the table; (3) virtual communities about food; (4) sharing and gaming; (5) commercial relationships in digital forms of commensality; and (6) eating together in the context of the COVID-19 pandemic. | | | |
|  | Health-related elements | Extracted a theme related to health in the scope review | | | |
|  | Summary of key findings related to health | （1）Finally, categories were created to group studies on the impact of the use of digital technologies in the food context on mental health and on the eating habits of populations and individuals, in which, respectively, the results reported the advantages of the intersections between the digital world and food, such as reducing loneliness and improving eating habits, and the disadvantages, such as encouraging excessive eating and maintaining behaviors associated with eating disorders. | | | |

*Food ads must include images of celebrities eating.

13. Associations between mukbang viewing and disordered eating behaviors

|  | Items | Data | | | |
| --- | --- | --- | --- | --- | --- |
| Inclusion criteria | Population/Main research object | used social media to recruit adults who watched mukbang at least once during the past year (n = 264). | | | |
|  | Concept (Tick)  Studies focusing on | 1. | *mukbang* watching (Food ads*) | √ | |
|  |  | 2. | the impact of *mukbang* (Food ads) on health | √ | |
|  |  | 3. | the relationship between *mukbang* (Food ads) and health | √ | |
|  |  | 4. | the design, development, or usability of *mukbang* (Food ads) watching interventions to demonstrate the usefulness of *mukbang* in the health field | NA | |
|  |  | 5. | evaluating the effectiveness of various types of *mukbang* (Food ads) for public health | NA | |
|  |  | 6. | the challenges and barriers of integrating *mukbang* (Food ads) videos into clinical practice | NA | |
|  |  | 7. | the advantages and/or disadvantages of *mukbang* (Food ads) watching | √ | |
|  | Types of evidence source | cross-sectional study | | | |
| Evidence source details and characteristics | Citation details (eg, author/s, date, title, journal, volume, issue, pages) | Title | Associations between mukbang viewing and disordered eating behaviors | | |
|  |  | Journal | International Journal of Eating Disorder | | |
|  |  | Author/s | von Ash T., Huynh R.,Deng C.,White M.A. | | |
|  |  | Date | 2023 | | |
|  | Main content of the evidence | Objective: We aim to examine the relationship between mukbang viewing characteristics and eating disorders symptoms.  Methods: Eating disorder symptoms were evaluated using the eating disorders examination–questionnaire. Frequency of mukbang viewing, average watch time per occasion, tendency to eat while watching mukbang, and problematic mukbang viewing (using the Mukbang Addiction Scale) were assessed. We used multivariable regressions to estimate associations between mukbang viewing characteristics and eating disorder symptoms, adjusting for gender, race/ethnicity, age, education, and BMI.  Result: A total of 34% of participants reported watching mukbang daily or almost daily, with mean watch time per viewing session being 29.94 min (SD = 1.00). Eating disorder symptoms, especially binge eating and purging, were associated with greater problematic mukbang viewing and a tendency to not consume food while viewing mukbang. Participants with greater body dissatisfaction watched mukbang more frequently and were more likely to eat while watching mukbang, yet they scored lower on the Mukbang Addiction Scale and watched fewer average minutes of mukbang per viewing occasion.  Discussion：our findings linking mukbang viewing and disordered eating may inform clinical diagnoses and treatments of eating disorders. | | | |
|  | Participant details (eg, age/sex and number) | Age | at least 18 years of age | | |
|  |  | Sex | Both: Fifty-one percent of participants identified as male, 46% as female, and 3% as non-binary | | |
|  |  | Final enrollment | Recruitment was conducted from March 23, 2021 to April 16, 2021. Consenting participants were eligible to participate if they were:  (1) had watched mukbang in the past 12 months. Eligibility questions were included at the beginning of the survey and skip logic was used to end the survey for those who were under the age of 18 or had not watched a mukbang video during the past year; 4% of respondents were deemed ineligible. Respondents were entered into a raffle for a gift card. | | |
| Details/results extracted from source of evidence | Review question addressed  (Tick) | 1. | The health-related elements in *mukbang* (Food ads) | NA | |
|  |  | Source text | NA | | |
|  |  | 2. | The relationship between *mukbang* (Food ads) and health |  | |
|  |  | Source text | (1) On the one hand, it is possible that mukbang viewing may encourage out-of-control/overeating by eliciting feelings of hunger among viewers or, through modeling, encourage consumption of hypercaloric foods that are often featured in mukbang videos. Just as exposure to television food advertisements may lead to overeating and consumption of unhealthy foods (Boulos et al., 2012), mukbang may have a similar impact on eating. More than two-thirds of the participants in our sample reported that they at least sometimes eat while watching mukbang, with a third eating very often or always while watching. While the original purpose of mukbang was to address loneliness while eating (Hong & Park, 2017; Spence et al., 2019), it remains unclear how quality and quantity of food consumed may be impacted by mukbang videos.  (2) Finally, both disordered eating and mukbang viewing may share a common cause. For example, feelings of loneliness can trigger or exacerbate eating disorder symptoms (Levine, 2012), and mukbang viewing has been shown to be positively associated with loneliness (Kircaburun, Balta, et al., 2021). Thus, it is possible that those who suffer from eating disorders may turn to mukbang to fulfill feelings of loneliness. Indeed, mukbang originated in Korea as a pop culture “cure” for loneliness while eating (Hong & Park, 2017;Spence et al., 2019) | | |
|  |  | 3. | Impacts may *mukbang* (Food ads) have on eating habits or eating behaviors | √ | |
|  |  | Source text | Rusult：  Table 4 shows the multivariable adjusted associations of disordered eating behaviors with mukbang viewing characteristics. After adjusting for gender, race/ethnicity, age, education, and BMI, participants with greater body dissatisfaction scored lower on the Mukbang Addiction Scale. Six of the seven binge/purge indicators (i.e., eating an unusually large amount of food, experiencing objective and subjective episodes of binge eating, inducing vomiting, using laxatives, and overexercising were associated with a higher score on the Mukbang Addiction Scale. Being upset by lack of control while eating unusually large portions was associated with a higher score in the unadjusted model but not after adjusting for gender, race/ethnicity, age, education, and BMI.  Discussion:  (1) Our study deepens the understanding of associations between disordered eating and mukbang viewing by showing that additional characteristics of mukbang viewing (e.g., frequency of watching, average watch time, and eating while watching), beyond problematic mukbang viewing or mukbang addiction, may be associated with disordered eating. By examining the EDE-Q subscales and binge eating and purging questions separately, our study also demonstrates how particular aspects of disordered eating psychopathology may be differentially correlated with various mukbang viewing characteristics. Our findings are also mutually supportive of a netnographic analysis of comments about mukbang that were left in online forums (Strand & Gustafsson, 2020)  (2) The netnographic analysis found that mukbang viewers expressed a desire to engage in binge eating, lessen the guilt of their own eating behaviors, and restrict caloric intake (Strand & Gustafsson, 2020). Our study showed a positive relationship between binge eating/purging tendencies and problematic mukbang viewing, which may corroborate previous findings on the potential impact of mukbang viewing on individual behavior. Contrary to the dietary-restraint-related comments extracted by Strand & Gustafsson, our study did not find dietary restraint to be positively associated with any mukbang viewing factors.  It is also possible that seeing the amount of and type of food that mukbangers eat may lead viewers to compare their own eating behaviors with those of the mukbangers and possibly change their eating behaviors to more closely emulate the binge-like eating behaviors that are typical of mukbangers (Polivy, 2017). Furthermore, seeing slim, fit mukbangers eat so much may cause people to feel dissatisfied with their own bodies perhaps due to an internalization of a thin ideal (Rühl et al., 2011), which is a precursor to disordered eating (Thompson & Stice, 2001). Additionally, mukbang may glorify binge eating, causing viewers to be more likely to engage in this disordered behavior (Kircaburun, Yurdagül, et al., 2021). On the contrary, some viewers report using mukbang to derive “vicarious pleasure” from eating that may prevent binge eating or eating in general (Kircaburun, Yurdagül, et al., 2021). Viewers have reported that watching mukbang helps them limit their food intake, noting that the large amount of food was “repellant” to them and would thus prevent eating (Strand & Gustafsson, 2020)—a potentially helpful notion for those who replace binge eating with mukbang watching but a counterproductive one for those who have issues with restrictive eating habits. Watching someone eat a certain food may result in a lower desire to eat that food especially when the viewer and the eater share similar identities (Tu & Fishbach, 2017). | | |
|  |  | 4. | The design, development, or usability of *mukbang* (Food ads) watching interventions to demonstrate the usefulness of *mukbang* in the health field | | NA |
|  |  | Source text | NA | | |
|  |  | 5. | Evaluation of the health effects of various types of *mukbang* (Food ads) | | NA |
|  |  | Source text | NA | | |
|  |  | 6. | The challenges and barriers of integrating *mukbang* (Food ads) videos into clinical practice | | NA |
|  |  | Source text | NA | | |
|  |  | 7. | The advantages and/or disadvantages of *mukbang* (Food ads) watching | |  |
|  |  | Source text |  | | |
|  | Research method/Tools | This cross-sectional study used data collected via an online survey about mukbang viewing. The survey was designed to take 20–30 min and administered via Qualtrics. Participants were recruited through online social media platforms including Facebook, Instagram, TikTok, and Reddit.   1. participants completed the Mukbang Addiction Scale, a validated self-report questionnaire used to measure mukbang viewing habits in the context of the six components of addiction: salience, conflict, withdrawal, mood modification, tolerance, and relapse (Kircaburun, Stavropoulos, et al., 2021). 2. Disordered eating symptoms were assessed using a modified brief version of the eating disorders examination–questionnaire (Grilo et al., 2015). The modified EDE-Q has three subscales: dietary restraint, shape/weight overvaluation, and body dissatisfaction. The Cronbach's alpha coefficients for each subscale in the study sample were .88, .81, and .57, respectively.   (3) Sociodemographic characteristics questionnaire | | | |
|  | Characteristics of *mukbang* | This study posted under several broad “genres” of mukbang, including those that feature Autonomous Sensory Meridian Response (ASMR) (audio-visual stimulation that can induce a pleasurable tingling in the head/neck), cookbang (videos that depict the cooking process in addition to eating), messy eating, “exotic” foods, different cultures of food, and so on. | | | |
|  | Health-related elements | Disordered eating symptoms | | | |
|  | Summary of key findings related to health | In this cross-sectional study of mukbang viewers, individuals with binge eating and purging behaviors generally exhibited higher levels of problematic mukbang watching and were less likely to eat while watching mukbang. Dietary restraint, overvaluation of body shape and weight, and body dissatisfaction had mixed associations with mukbang viewing characteristics. Based on Cohen's guidelines, the associations we found represent small local effect sizes (Cohen, 1988).  (1 )Our study deepens the understanding of associations between disordered eating and mukbang viewing by showing that additional characteristics of mukbang viewing (e.g., frequency of watching, average watch time, and eating while watching), beyond problematic mukbang viewing or mukbang addiction, may be associated with disordered eating. By examining the EDE-Q subscales and binge eating and purging questions separately, our study also demonstrates how particular aspects of disordered eating psychopathology may be differentially correlated with various mukbang viewing characteristics. Our findings are also mutually supportive of a netnographic analysis of comments about mukbang that were left in online forums (Strand & Gustafsson, 2020)  Our study showed a positive relationship between binge eating/purging tendencies and problematic mukbang viewing, which may corroborate previous findings on the potential impact of mukbang viewing on individual behavior. Contrary to the dietary-restraint-related comments extracted by Strand & Gustafsson, our study did not find dietary restraint to be positively associated with any mukbang viewing factors.  However, the directional relationship between mukbang viewing and disordered eating remains untested. That is, individuals who exhibit disordered eating behaviors may be more likely to watch mukbang. Those with restrictive dietary behaviors (i.e., starvation, weight loss, symptom of an eating disorder) often become preoccupied with food (Keys et al., 1950; Polivy, 1996), which may cause them to search for food-related content like mukbang. This is consistent with literature on the Internet compensatory theory, which states that people use the internet to fulfill unmet desires (in this case, eating) in real life (Kardefelt-Winther, 2014). Furthermore, it has been shown that individuals with binge eating disorder have greater neural activations in the reward system in response to food images (Schienle et al., 2009); this elevated reward sensitivity may cause a predisposition to search for food-related media, such as mukbang.  Our findings are consistent with the literature demonstrating that mukbang viewing is associated with eating disorder symptoms (Kircaburun, Yurdagül, et al., 2021). While Kircaburun, Yurdagül, et al. | | | |

*Food ads must include images of celebrities eating.

14. Exploring adolescents’ motives for food media consumption using the theory of uses and gratifications

|  | Items | Data | | | | |
| --- | --- | --- | --- | --- | --- | --- |
| Inclusion criteria | Population/Main research object | 31 Flemish adolescents aged 12 to 16. | | | | |
|  | Concept (Tick)  Studies focusing on | 1. | *mukbang* watching (Food ads*) | | √ | |
|  |  | 2. | the impact of *mukbang* (Food ads) on health | | √ | |
|  |  | 3. | the relationship between *mukbang* (Food ads) and health | | √ | |
|  |  | 4. | the design, development, or usability of *mukbang* (Food ads) watching interventions to demonstrate the usefulness of *mukbang* in the health field | | NA | |
|  |  | 5. | evaluating the effectiveness of various types of *mukbang* (Food ads) for public health | | NA | |
|  |  | 6. | the challenges and barriers of integrating *mukbang* (Food ads) videos into clinical practice | | √ | |
|  |  | 7. | the advantages and/or disadvantages of *mukbang* (Food ads) watching | | √ | |
|  | Types of evidence source | Qualitative research | | | | |
| Evidence source details and characteristics | Citation details (eg, author/s, date, title, journal, volume, issue, pages) | Title | | Exploring adolescents’ motives for food media consumption using the theory of uses and gratifications | | |
|  |  | Journal | | Communications | | |
|  |  | Author/s | | Yandisa Ngqangashe, Katrien Maldoy, Charlotte J. S. De Backer, Heidi Vandebosch | | |
|  |  | Date | | 2022-03-28 | | |
|  | Main content of the evidence | This study seeks to explore food media use among adolescents. The food media explored in this study entail traditional food media in the form of TV cooking shows and new media in the form of YouTube cooking channels, social media culinary videos, social media food posts and online recipes. This study seeks to explore incidental consumption in the context of food media. This study also approaches selective consumption of food media from a uses and gratifications perspective.  The main part of the semi-structure interviews was to introduce the topic, and the participants were asked how they would define food media, which media exposed them to food, and how they perceived the influence of this exposure. | | | | |
|  | Participant details (eg, age/sex and number) | Age | | Between the ages of 12 and 16 | | |
|  |  | Sex | | Both | | |
|  |  | Final enrollment | | 31 middle-school adolescents (14 male, 17 female). | | |
| Details/results extracted from source of evidence | Review question addressed  (Tick) | 1. | The health-related elements in *mukbang* (Food ads) | | √ | |
|  |  | Source text | Healthy food, dessert, unhealthy food. | | | |
|  |  | 2. | The relationship between *mukbang* (Food ads) and health | | √ | |
|  |  | Source text | 1) For the purpose of obtaining health information or maintaining health: some participants consumed food media to get motivation and inspiration to improve health and fitness (Fitspiration).  2) Through audio-visual food media such as YouTube and television, adolescents can learn cooking with the video anchors, which can make them love cooking by themselves and eat much healthier without frequently ordering takeouts.  3) TV cooking shows can guide adolescents to try to make healthy food for themselves: I think it’s pretty interesting how they make it. Uh ... sometimes I make something to eat healthy myself.  4) The participants in this study associated food media with “diets” and “healthy lifestyles”.  5) Occasional exposure to food media can also promote the health of the exposed person, but this must be based on the content of the food media itself is healthy: a pop star who posts on social media about healthy foods may influence his or her followers to eat more healthily as well. | | | |
|  |  | 3. | Impacts may *mukbang* (Food ads) have on eating habits or eating behaviors | | √ | |
|  |  | Source text | 1) Often contact with food media online (like YouTube cooking shows), may change the viewers’ eating habits and food choices that food content can generate an immediate desire to eat or cook something similar. Participants who mentioned this type of influence often associated this with sweet foods and advertising.  2) Change one’s food choices to a healthier option: perceived social media food content as a source of inspiration to making healthy food choices as well as being a distraction.  3) Food influencers were found to influence the food choices of their followers: “Then you see someone else making healthy food and then you go make it yourself, consciously or unconsciously, as well.” | | | |
|  |  | 4. | The design, development, or usability of *mukbang* (Food ads) watching interventions to demonstrate the usefulness of *mukbang* in the health field | | | NA |
|  |  | Source text | NA | | | |
|  |  | 5. | Evaluation of the health effects of various types of *mukbang* (Food ads) | | | NA |
|  |  | Source text | NA | | | |
|  |  | 6. | The challenges and barriers of integrating *mukbang* (Food ads) videos into clinical practice | | | √ |
|  |  | Source text | The genre differences in motives are also important for targeted interventions that seek to use food media to endorse healthy eating behaviors. However, the predominant use of online media as a source of information or inspiration, the preference for watching sweet foods, and the association of food media and health warrants concern (challenges and barriers) given that food media are known to predominantly portray restrictive eating behaviors and unhealthy food. | | | |
|  |  | 7. | The advantages and/or disadvantages of *mukbang* (Food ads) watching | | | √ |
|  |  | Source text | Advantage: social support: using YouTube for companionship, and this was primarily with friends, what’s more, others watched TV cooking shows to be in the company of family. | | | |
|  | Research method/Tools | In-depth, semi-structured interviews  A phenomenology approach | | | | |
|  | Characteristics of *mukbang* | Online food content comes in multiple formats such as food blogs, YouTube cooking channels, short-form culinary recipes, online recipes, and social-media posts. The food media in this study mainly refers to YouTube cooking channels, which has been seen as one form of mukbang. | | | | |
|  | Health-related elements | Cook food at home  Be influenced by the healthy food media content to choose healthy food. | | | | |
|  | Summary of key findings related to health | 1) The participants in this study did not consume food media to pass time or to escape but incidentally encountered food media while seeking to pass time or to escape by watching television or using the internet.  2) The entertainment motives that emerged were food porn, laughter, voyeurism, passing time, and escapism.  3) The influence of food media on teenagers is mainly based on the content it presents. If the food shown in the content is healthy, it will make teenagers' food choices tend to be healthy. But if the video features foods that are generally considered unhealthy, such as desserts, it could lead to unhealthy food choices.  4) Food media makes adolescents more willing to try to cook for themselves, so that their eating habits can develop in a healthier direction. | | | | |

*Food ads must include images of celebrities eating.

15. Psychological exploration of “Mukbang” audience from the perspective of structuralism

|  | Items | Data | | | | |
| --- | --- | --- | --- | --- | --- | --- |
| Inclusion criteria | Population/Main research object | NA | | | | |
|  | Concept (Tick)  Studies focusing on | 1. | *mukbang* watching (Food ads*) | | √ | |
|  |  | 2. | the impact of *mukbang* (Food ads) on health | | √ | |
|  |  | 3. | the relationship between *mukbang* (Food ads) and health | | √ | |
|  |  | 4. | the design, development, or usability of *mukbang* (Food ads) watching interventions to demonstrate the usefulness of *mukbang* in the health field | | NA | |
|  |  | 5. | evaluating the effectiveness of various types of *mukbang* (Food ads) for public health | | NA | |
|  |  | 6. | the challenges and barriers of integrating *mukbang* (Food ads) videos into clinical practice | | NA | |
|  |  | 7. | the advantages and/or disadvantages of *mukbang* (Food ads) watching | | √ | |
|  | Types of evidence source | Commentary | | | | |
| Evidence source details and characteristics | Citation details (eg, author/s, date, title, journal, volume, issue, pages) | Title | | Psychological exploration of “Mukbang” audience from the perspective of structuralism | | |
|  |  | Journal | | JOURNAL OF HEBEI UNIVERSITY OF ECONOMICS AND BUSINESS | | |
|  |  | Author/s | | Chuanxi Han | | |
|  |  | Date | | 2018-9 | | |
|  | Main content of the evidence | In recent years, the rapid development of live broadcasting platforms from the initial game live gradually expanded to live electricity and pan-entertainment live in China, which mukbang with its content specificity, coupled with the rapid expansion of the anchor group and the user group, has become a kind of mass culture communication phenomenon worthy of in-depth consideration. Based on structuralist theory and the rapid development of various live broadcasting platforms, it is of unique significance to analyze the psychological motivation of users in choosing to watch mukbang, and to analyze the spiritual condition of contemporary people reflected by the phenomenon of mukbang through the examination of the fulfillment of their different psychological needs and the problems they create. | | | | |
|  | Participant details (eg, age/sex and number) | Age | | NA | | |
|  |  | Sex | | NA | | |
|  |  | Final enrollment | | NA | | |
| Details/results extracted from source of evidence | Review question addressed  (Tick) | 1. | The health-related elements in *mukbang* (Food ads) | | √ | |
|  |  | Source text | Compensatory psychology | | | |
|  |  | 2. | The relationship between *mukbang* (Food ads) and health | | √ | |
|  |  | Source text | 1) Loneliness can lead to unhealthy behaviors such as eating high-calorie junk food. And mukbang videos are just a perfect and best outlet for venting out with both social interaction and detoxifying your appetite. | | | |
|  |  | 3. | Impacts may *mukbang* (Food ads) have on eating habits or eating behaviors | | √ | |
|  |  | Source text | 1) Viewers choose to watch mukbang for the motive of satisfying their different psychological needs. There are many motives, for example, women who are on a diet are restraining their food intake because they have high expectations of their body image. The anchor's reaction makes the viewer feel empathetic, watching the anchor eat as if he or she is also consuming the same amount of food as the anchor, seeking a sense of satiety brought about by visual and auditory stimulation. Some viewers who are unable to eat too much food due to their small appetites want to experience the same feeling of consuming a large amount of food as the anchor. | | | |
|  |  | 4. | The design, development, or usability of *mukbang* (Food ads) watching interventions to demonstrate the usefulness of *mukbang* in the health field | | | NA |
|  |  | Source text | NA | | | |
|  |  | 5. | Evaluation of the health effects of various types of *mukbang* (Food ads) | | | NA |
|  |  | Source text | NA | | | |
|  |  | 6. | The challenges and barriers of integrating *mukbang* (Food ads) videos into clinical practice | | | NA |
|  |  | Source text | NA | | | |
|  |  | 7. | The advantages and/or disadvantages of *mukbang* (Food ads) watching | | | √ |
|  |  | Source text | 1) Many viewers think that watching mukbang is a release of pressure, but also an escape from social pressure.  2) No matter what the audience is doing when they watch mukbang, the sounds and images coming from the electronic devices already make them feel that they are not alone, but that they are surrounded by real people, a warmth that is not felt between the concrete high-rise buildings and the cold neighborhood.  3) Mukbang blocks the viewer's opportunity to interact and communicate more with their family and friends around them, damaging the viewer's social interaction and socialization skills in reality. At the same time, the viewer is lost in the subtle influence of the mass media and is unable to think, indulging in the simplest pleasures and gradually losing the ability to learn on their own and to socialize in real life. This hypnosis-like process makes viewers emotionally stunted.  4) The nature of Mukbang itself leads to the superficial pleasures it offers being desire-venting and lacking in spiritual engagement and practical meaning. Prolonged exposure to mass media, especially at a low level such as mukbang, reduces people's ability to think, and they become addicted to a flood of passive, receptive information, leading to a vicious circle. | | | |
|  | Research method/Tools | NA | | | | |
|  | Characteristics of *mukbang* | NA | | | | |
|  | Health-related elements | psychological factors | | | | |
|  | Summary of key findings related to health | This study believed that mukbang can play a visual decompression effect through its attractive food images, which can satisfy some of the audience's instinctive impulses. The conclusion of this paper pointed out that watching mukbang can make the audience gain a sense of pleasure, release the pressure and loneliness in real life. However, because of this, mukbang blocked them from more communication opportunities with the family and friends around them, damaged the social interaction and social ability in reality, what’s more, mukbang also had the risk of addiction. Therefore, mukbang is a double-edged sword for the audience, both helpful and potentially harmful. | | | | |

*Food ads must include images of celebrities eating.

16. Correlation analysis of watching motivation of mukbang and the influence of mukbang on audience

|  | Items | Data | | | | |
| --- | --- | --- | --- | --- | --- | --- |
| Inclusion criteria | Population/Main research object | Questionnaires were published online for people of all ages | | | | |
|  | Concept (Tick)  Studies focusing on | 1. | *mukbang* watching (Food ads*) | | √ | |
|  |  | 2. | the impact of *mukbang* (Food ads) on health | |  | |
|  |  | 3. | the relationship between *mukbang* (Food ads) and health | | √ | |
|  |  | 4. | the design, development, or usability of *mukbang* (Food ads) watching interventions to demonstrate the usefulness of *mukbang* in the health field | | NA | |
|  |  | 5. | evaluating the effectiveness of various types of *mukbang* (Food ads) for public health | | NA | |
|  |  | 6. | the challenges and barriers of integrating *mukbang* (Food ads) videos into clinical practice | | NA | |
|  |  | 7. | the advantages and/or disadvantages of *mukbang* (Food ads) watching | | √ | |
|  | Types of evidence source | Cross-sectional study | | | | |
| Evidence source details and characteristics | Citation details (eg, author/s, date, title, journal, volume, issue, pages) | Title | | Correlation analysis of watching motivation of mukbang and the influence of mukbang on audience | | |
|  |  | Journal | | PR Magazine | | |
|  |  | Author/s | | Boru Li | | |
|  |  | Date | | 2020 | | |
|  | Main content of the evidence | Based on the preliminary research and summary on the reasons for audience to watch mukbang and the impact of mukbang on audience, this paper adopts a variety of research methods to analyze the correlation between the two factors. Through data collection and collation, we can first see the different role degree, universality and applicability of each viewing motivation, and add new motivation; Then, based on daily observation and reasonable speculation, three mukbang types are selected which may have a great impact, and people are investigated in specific situations to analyze their respective influence degree and influence scope. Finally, the two contents of "motivation" and "influence" are combined for correlation analysis, to see whether there is correlation and causality between various factors that people have not noticed, and to obtain hypotheses and verify them to find out the internal logic of each connection. | | | | |
|  | Participant details (eg, age/sex and number) | Age | | All ages | | |
|  |  | Sex | | Both | | |
|  |  | Final enrollment | | Finally, 84 questionnaires with high reference value were included for analysis | | |
| Details/results extracted from source of evidence | Review question addressed  (Tick) | 1. | The health-related elements in *mukbang* (Food ads) | | NA | |
|  |  | Source text | NA | | | |
|  |  | 2. | The relationship between *mukbang* (Food ads) and health | | √ | |
|  |  | Source text | One noteworthy correspondence is that people who like to listen to the sound of chewing food usually enjoy ASMR Mukbang to help them sleep, and at the same time tend to watch food podcasts that are high in calories and high in fat. In order to study this correspondence in depth, this paper, through an in-depth interview with a mukbang viewer, finds that ASMR and high-calorie food, although seemingly unrelated, have an inherent logic and complementary needs: when viewers watch mukbangs to satisfy their appetite, they will be interested in the more exaggerated mukbangs, especially those that contain high-calorie and high-fat food. The impact and excitement of the mukbang allows the viewer to release the stress she feels in her daily life and gain psychological relief. To maximize this effect, a clear chewing sound is essential, as the sound of chewing and the visual effect of an exaggerated mouthful of food can amplify the impact and excitement of the mukbang and enhance the stress relief effect. | | | |
|  |  | 3. | Impacts may *mukbang* (Food ads) have on eating habits or eating behaviors | | NA | |
|  |  | Source text | NA | | | |
|  |  | 4. | The design, development, or usability of *mukbang* (Food ads) watching interventions to demonstrate the usefulness of *mukbang* in the health field | | | NA |
|  |  | Source text | NA | | | |
|  |  | 5. | Evaluation of the health effects of various types of *mukbang* (Food ads) | | | NA |
|  |  | Source text | NA | | | |
|  |  | 6. | The challenges and barriers of integrating *mukbang* (Food ads) videos into clinical practice | | | NA |
|  |  | Source text | NA | | | |
|  |  | 7. | The advantages and/or disadvantages of *mukbang* (Food ads) watching | | | √ |
|  |  | Source text | Advantages:  1) When users watch mukbang when they "feel lonely and need company" or "have nothing to do to pass the time," the emotional feedback they get is usually "relieving stress, releasing anxiety, relaxing and resting."  2) When the audience opens mukbang out of the motivation of "being lonely and hoping to be accompanied", their choice of mukbang is usually based on the individual host, that is, the "streamer-oriented audience". And such motivation will produce a chain reaction, such as these viewers will pay attention to the activities and videos that the anchors participate in, regardless of whether they are really interested in the content. And tend to probe the daily life of the anchor, and even have a certain degree of emotional dependence on the anchor. Through interviews, this paper finds that for more and more viewers, this is a kind of psychological dependence, people will tend to eat in the company of mukbang, and become a state of life.  3) The second audience is the "food-oriented audience," who tend to increase the frequency of mukbang during weight loss. For them, the motivation for watching mukbang is to satisfy the need for food in the heart, not the individual anchor. Because the method of controlling diet to lose weight makes the audience have a stronger desire for food, mukbang will bring the audience stronger sensory stimulation at this time. | | | |
|  | Research method/Tools | Cross-sectional study | | | | |
|  | Characteristics of *mukbang* | NA | | | | |
|  | Health-related elements | Numerous psychology | | | | |
|  | Summary of key findings related to health | According to this study, people who like to listen to the sound of food chewing often enjoy ASMR Mukbang to help them sleep, and tend to watch high-calorie, high-fat food mukbang. When viewers watch mukbang to satisfy their own appetite, they will be interested in mukbang filled with high-calorie food. This kind of mukbang usually combines the sound of chewing and the visual effect of exaggerated large meals, which virtually amplifies the sense of impact and stimulation brought by mukbang and improves the decompression effect. | | | | |

*Food ads must include images of celebrities eating.

17. Binge Drinking and Obesity-Related Eating The Moderating Roles of the Eating Broadcast Viewing Experience among Korean Adults

|  | Items | Data | | | | |
| --- | --- | --- | --- | --- | --- | --- |
| Inclusion criteria | Population/Main research object | This study analyzed the moderating effect of the eating broadcast viewing experience on the relationship between binge drinking and obesity-related eating behaviors.  Study question: how binge drinking affects obesity-related eating behaviors.  The purpose of this study was to identify the interaction between binge drinking and eating broadcast viewing experience on obesity-related eating behaviors among Korean adults. Besides this, the study also aimed to find out which obesity-related eating behavior was affected by the interaction between binge drinking and eating. | | | | |
|  | Concept (Tick)  Studies focusing on | 1. | *mukbang* watching (Food ads*) | | √ | |
|  |  | 2. | the impact of *mukbang* (Food ads) on health | | √ | |
|  |  | 3. | the relationship between *mukbang* (Food ads) and health | | √ | |
|  |  | 4. | the design, development, or usability of *mukbang* (Food ads) watching interventions to demonstrate the usefulness of *mukbang* in the health field | | √ | |
|  |  | 5. | evaluating the effectiveness of various types of *mukbang* (Food ads) for public health | | √ | |
|  |  | 6. | the challenges and barriers of integrating *mukbang* (Food ads) videos into clinical practice | | NA | |
|  |  | 7. | the advantages and/or disadvantages of *mukbang* (Food ads) watching | | √ | |
|  | Types of evidence source | Cross-sectional self-reported online survey | | | | |
| Evidence source details and characteristics | Citation details (eg, author/s, date, title, journal, volume, issue, pages) | Title | | Binge Drinking and Obesity-Related Eating: The Moderating Roles of the Eating Broadcast Viewing Experience among Korean Adults | | |
|  |  | Journal | | International Journal of Environmental Research and Public Health | | |
|  |  | Author/s | | Jiye Kim, Saegyeol Choi, Hyekyeong Kim, and Soontae An | | |
|  |  | Date | | 2021-07-30 | | |
|  | Main content of the evidence | The goal of this study was to examine whether eating broadcast viewing experiences affected the association between the frequency of binge drinking and obesity-related eating behaviors among Korean adults. This is the first study, to our knowledge, to investigate the influence of eating broadcast viewership as an environmental factor related to binge drinking. To understand the detrimental effects of binge drinking on obesity-related eating behaviors, this study suggests watching broadcasts as an environmental factor associated with binge drinking.  Mukbang watching in this study is only mediate between binge drinking and obesity-related eating behaviors, and it has also been identified as an environmental factor that influences both binge drinking and obesity-related eating behaviors. | | | | |
|  | Participant details (eg, age/sex and number) | Age | | at least 19 years old, | | |
|  |  | Sex | | Both | | |
|  |  | Final enrollment | | A total of 1125 Korean people finished the online questionnaire. | | |
| Details/results extracted from source of evidence | Review question addressed  (Tick) | 1. | The health-related elements in *mukbang* (Food ads) | | √ | |
|  |  | Source text | Mukbang may include drinking alcohol. | | | |
|  |  | 2. | The relationship between *mukbang* (Food ads) and health | | √ | |
|  |  | Source text | Not only does the experience of watching eating broadcasts become external food stimuli, but so does the interaction with binge drinking.  This study had found that people who viewed eating broadcasts by online platforms may had more external eating with more frequent binge drinking, especially among women and those in their 20s. | | | |
|  |  | 3. | Impacts may *mukbang* (Food ads) have on eating habits or eating behaviors | | √ | |
|  |  | Source text | 1) A prior study: preliminary study reported a positive association between watching eating broadcasts and eating disorders.  2) Viewing eating broadcasts online and on the TV had higher scores in restrained eating behaviors than those who had never seen an eating broadcast.  3) People who watched eating broadcasts on both the TV and online channels scored higher for emotional eating and external eating behaviors than those who never watched an eating broadcast and only watched TV broadcasts.  4) The effect of the interaction between binge drinking and binge-watching on sexual eating was significant only in women in their 20s.  5) Online channels were more likely than TV to broadcast more aggressive content, including drinking alcohol, as a visual food stimulus that could induce external eating.  6) Watching online eating broadcasts is likely to influence some eating behaviors in Koreans in their 20s with serious binge drinking issues. | | | |
|  |  | 4. | The design, development, or usability of *mukbang* (Food ads) watching interventions to demonstrate the usefulness of *mukbang* in the health field | | | √ |
|  |  | Source text | Future research should consider various aspects of the eating broadcast viewing experience, media type, and how TV and online eating broadcasts content, particularly those that involve drinking alcohol, affect binge and problem drinking. To prevent the negative effects of eating broadcasts on eating behaviors and obesity-related health problems, the government should provide educational support. | | | |
|  |  | 5. | Evaluation of the health effects of various types of *mukbang* (Food ads) | | | √ |
|  |  | Source text | TV eating broadcast viewing: people in their 20s who have only watched TV eating broadcasts is that the more frequently they binge drink, the lower their level of external eating.  This raises the possibility that watching TV eating broadcasts may protect viewers against the unhealthy relationships between binge drinking frequency and external eating.  Online eating broadcast viewing: for those in their 20s, the more frequently they watch online mukbang videos, the higher level of external eating. | | | |
|  |  | 6. | The challenges and barriers of integrating *mukbang* (Food ads) videos into clinical practice | | | NA |
|  |  | Source text | NA | | | |
|  |  | 7. | The advantages and/or disadvantages of *mukbang* (Food ads) watching | | | √ |
|  |  | Source text |  | | | |
|  | Research method/Tools | A cross-sectional self-reported online questionnaire-based survey  1) Sociodemographic questionnaire  2) Stress levels: question  3) Depression level: Korean version of the Depression Screening Tool (PHQ-9)  4) use two questions to assess eating broadcast viewing experiences  5) Obesity-Related Eating Behaviors: The Dutch Eating Behavior Questionnaire (DEBQ) | | | | |
|  | Characteristics of *mukbang* | These eating broadcasts show various types of food and eating behaviors and are posted in a format that can be accessed by anyone, regardless of age.  This study investigated the health problems caused by watching mukbang concerning binge drinking.  TV eating broadcast viewing & Online eating broadcast viewing  The programs include broadcast cooking, consuming food, or talking about food are identified as mukbang. | | | | |
|  | Health-related elements | Obesity-related eating behaviors: restrained eating behaviors, emotional eating behaviors, and external eating behaviors.  Binge drinking: the person who already has binge drinking, not the mukbang watching cause the binge drinking. | | | | |
|  | Summary of key findings related to health | 1) More frequent binge drinking was associated with a higher level of external eating in participants who only watched online eating broadcasts, especially among women.  2) An eating broadcast viewing experience was one of the environmental factors associated with binge drinking that influences obesity-related eating behaviors.  3) The average stress level was highest in people who watched eating broadcasts on TV and online. —— These people may use mukbang to release stress.  4) Health problems caused by binge drinking should be considered with social and environmental elements, such as the media, rather than focusing solely on causality at the biological level.  5) There was no significant difference in binge drinking frequency depending on the experience of watching eating broadcasts in this study, but the interaction between the two affected external eating.  6) The interaction between binge drinking frequency and eating broadcast viewing experience was not related to attempts to limit food and eating due to unpleasant feelings, but rather to external food-related cues. | | | | |

*Food ads must include images of celebrities eating.

18. Mukbang and Disordered Eating a Netnographic Analysis of Online Eating Broadcasts

|  | Items | Data | | | | |
| --- | --- | --- | --- | --- | --- | --- |
| Inclusion criteria | Population/Main research object | The aim of this study was to explore how viewers of mukbang videos relate their audience experiences to symptoms of disordered eating. | | | | |
|  | Concept (Tick)  Studies focusing on | 1. | *mukbang* watching (Food ads*) | | √ | |
|  |  | 2. | the impact of *mukbang* (Food ads) on health | | √ | |
|  |  | 3. | the relationship between *mukbang* (Food ads) and health | | √ | |
|  |  | 4. | the design, development, or usability of *mukbang* (Food ads) watching interventions to demonstrate the usefulness of *mukbang* in the health field | | NA | |
|  |  | 5. | evaluating the effectiveness of various types of *mukbang* (Food ads) for public health | | √ | |
|  |  | 6. | the challenges and barriers of integrating *mukbang* (Food ads) videos into clinical practice | | NA | |
|  |  | 7. | the advantages and/or disadvantages of *mukbang* (Food ads) watching | | √ | |
|  | Types of evidence source | Qualitative analysis: a netnographic approach | | | | |
| Evidence source details and characteristics | Citation details (eg, author/s, date, title, journal, volume, issue, pages) | Title | | Mukbang and Disordered Eating: A Netnographic Analysis of Online Eating Broadcasts | | |
|  |  | Journal | | Culture, Medicine and Psychiatry | | |
|  |  | Author/s | | Mattias Strand · Sanna Aila Gustafsson | | |
|  |  | Date | | 2020-12 | | |
|  | Main content of the evidence | With the aim of understanding how viewers of online mukbang videos relate their audience experiences to symptoms of disordered eating, such as restrictive eating, binge eating, or purging behaviors.  The main questions of this study:  1) Are mukbang videos perceived as influencing eating and/or purging behaviors in viewers?  2) If so, in what ways are they described as triggering disordered eating behaviors?  3) In what ways are they, on the contrary, described as potentially helpful for viewers in coping with and recovering from disordered eating? | | | | |
|  | Participant details (eg, age/sex and number) | Age | | NA | | |
|  |  | Sex | | NA | | |
|  |  | Final enrollment | | 1) On YouTube, the three most viewed videos from five popular mukbang hosts—i. e., a total of 15 videos—were included in this analysis.  2) Fifteen relevant subreddits that contained discussions about mukbang.  In total, 1316 user comments (986 YouTube user comments and 330 Reddit user comments) were included in the final qualitative content analysis. | | |
| Details/results extracted from source of evidence | Review question addressed  (Tick) | 1. | The health-related elements in *mukbang* (Food ads) | | √ | |
|  |  | Source text | The amounts of food ingested in a typical mukbang are conspicuously large; sometimes this involves large amounts of a single food-item (such as ramen noodles or fried chicken) but a mukbang may, for example, also consist of multiple double portion dishes. | | | |
|  |  | 2. | The relationship between *mukbang* (Food ads) and health | | √ | |
|  |  | Source text | 1) “A double-edged sword”: anecdotal reports — how watching mukbang videos can trigger and reinforce disordered eating behaviors such as binge eating or purging behaviors, but that it may actually also help some individuals to restrain from binge eating or encourage overly restrictive eaters to grow an appetite and become more comfortable in eating in a social setting.  2) Reduces guilt about own eating: but it would help satisfy an urge ig, and these videos kinda helped normalize eating for me I watch these videos to feel better about the amount of food I eat.  3) Viewer perspective (1022 comments): from one of the interviewees “At no point was any of these videos and tv programs made for binge eating. At some point, Mukbang became an international thing and then it transformed into some weird binge eating concept.”  4) Participant perspective (269 comments): watching mukbang may increase eating or restrict eating. Increasing or restricting diet is not necessarily bad for all people.  5) Participant perspective (269 comments): from one of the interviewees “Some days it inspires me to eat more, some days it makes me feel like restricting. I think it does two things for me: 1. I get a vicarious satisfaction out of it (like, they’re eating, so I don’t have to), and 2. It more or less normalizes eating for me– I have a lot of weird shame around eating, like many people with eating disorders, and seeing other people eat without serious concern is vaguely validating.”  6) Help to spread the concept of health among mukbangers and viewers: these supportive comments also often encourage mukbang hosts not to feel obligated to challenge themselves or try to impress viewers with excessive amounts of food, but to instead post videos of themselves eating more balanced meals for the sake of their own health. This can also be understood as the effect of mukbang watching on health perceptions is not necessarily negative, and for people with strong health perceptions, mukbang do not play a significant role.  7) Unhealthy aspect: individuals who appear to be restricting their eating in an unhealthy way recurrently attest to “eating vicariously” through the mukbang host, i.e., watching mukbang makes them feel that they themselves do not need to eat.  8) Users who appear to have a history of binge eating: watching mukbang helps them stave off a binge by finding the amounts of food ingested in the video’s repellent, but more often they too describe a more pleasant experience of calm in seeing someone else eat “for” them.  9) Users appear to have a history of binge eating and attest to how watching mukbang videos may trigger them to relapse into loss-of-control eating.  10) In a context of struggling with low appetite and/or selective eating and finding mukbang helpful as an inspiration in increasing food intake. | | | |
|  |  | 3. | Impacts may *mukbang* (Food ads) have on eating habits or eating behaviors | | √ | |
|  |  | Source text | 1) Mukbang can reduce loneliness and makes people feel like they are having dinner with someone. Mukbang can help get back into the habit of eating together.  2) Users describe how mukbang reduces guilt about their own eating and that watching mukbang videos is helpful in normalizing eating (e.g., demonstrating that eating can be a joyful experience and that occasional overeating does not automatically need to evoke feelings of guilt).  3) Disordered eating: maintain a restrictive diet by watching mukbang. | | | |
|  |  | 4. | The design, development, or usability of *mukbang* (Food ads) watching interventions to demonstrate the usefulness of *mukbang* in the health field | | | NA |
|  |  | Source text | NA | | | |
|  |  | 5. | Evaluation of the health effects of various types of *mukbang* (Food ads) | | | NA |
|  |  | Source text | NA | | | |
|  |  | 6. | The challenges and barriers of integrating *mukbang* (Food ads) videos into clinical practice | | | √ |
|  |  | Source text | Clinicians working with patients with eating disorders should be aware of the mukbang phenomenon as a potentially influencing factor. | | | |
|  |  | 7. | The advantages and/or disadvantages of *mukbang* (Food ads) watching | | | √ |
|  |  | Source text | Mukbang phenomenon is not necessarily seen as either helpful or destructive, but instead as simultaneously useful and hurtful.  The advantages and disadvantages of mukbang are varies from person to person. | | | |
|  | Research method/Tools | Netnographic analysis | | | | |
|  | Characteristics of *mukbang* | Most foods consumed in mukbang videos appear to be relatively cheap; e.g., multiple packs of instant ramen noodles.  ASMR mukbang: the mukbangers in this kind of mukbang videos slurping and chewing in a delicate manner. | | | | |
|  | Health-related elements | Disordered eating is a broad term describing various types of problematic eating behaviors, such as restrictive eating, binge eating, and associated compensatory behaviors (e.g., vomiting, laxative misuse, or excessive physical exercise). | | | | |
|  | Summary of key findings related to health | 1) Watching mukbang is not necessarily experienced as either helpful or destructive, but instead as simultaneously useful and hurtful.  2) Engaging in mukbang as a way of trying to increase food intake in a context of low appetite or selective eating, or as a way of actually preventing a binge eating episode.  3) A comment about how watching mukbang makes the viewer eat more may point to mukbang as a helpful tool for an individual who is struggling with trying to increase the amounts and variety of food accepted and to gain weight. In contrast, the very same comment may be seen as a sign of the potential triggering and unhelpful nature of mukbang if made by an individual with a previous binge eating disorder who has now relapsed into loss-of-control eating.  4) Mukbang watching is not necessarily seen as helpful or destructive, but instead simultaneously useful and hurtful.  5) Mukbang watching is often complex for the individual, for a person watching mukbang may produce a variety of completely different or interconnected emotions  6) Not least, there is often a striking ambivalence in how users are influenced by and make sense of mukbang. For some, mukbang appears to be a constructive tool in increasing food intake, preventing binge eating, or reducing loneliness. For others, it is clearly a destructive force that may motivate restrictive eating or trigger a relapse into loss-of-control eating. Perhaps most notably, the mukbang phenomenon is not necessarily seen as either helpful or destructive, but instead as simultaneously useful and hurtful. | | | | |

*Food ads must include images of celebrities eating.

19. Compensatory Usage of the Internet The Case of Mukbang Watching on YouTube

|  | Items | Data | | | | |
| --- | --- | --- | --- | --- | --- | --- |
| Inclusion criteria | Population/Main research object | Participants were Turkish mukbang viewers who completed an online survey. Participants had to be mukbang viewers (i.e., watched mukbang videos at least once in their lifetime) | | | | |
|  | Concept (Tick)  Studies focusing on | 1. | *mukbang* watching (Food ads*) | | √ | |
|  |  | 2. | the impact of *mukbang* (Food ads) on health | | √ | |
|  |  | 3. | the relationship between *mukbang* (Food ads) and health | | √ | |
|  |  | 4. | the design, development, or usability of *mukbang* (Food ads) watching interventions to demonstrate the usefulness of *mukbang* in the health field | | NA | |
|  |  | 5. | evaluating the effectiveness of various types of *mukbang* (Food ads) for public health | | NA | |
|  |  | 6. | the challenges and barriers of integrating *mukbang* (Food ads) videos into clinical practice | | NA | |
|  |  | 7. | the advantages and/or disadvantages of *mukbang* (Food ads) watching | | √ | |
|  | Types of evidence source | Cross-sectional questionnaire-based study | | | | |
| Evidence source details and characteristics | Citation details (eg, author/s, date, title, journal, volume, issue, pages) | Title | | Compensatory Usage of the Internet: The Case of Mukbang Watching on YouTube | | |
|  |  | Journal | | Psychiatry Investigation | | |
|  |  | Author/s | | Kagan Kircaburun, Sabah Balta, Emrah Emirtekin, Sule Betül Tosunta, Zsolt Demetrovics, and Mark D. Griffiths | | |
|  |  | Date | | 2021-04 | | |
|  | Main content of the evidence | The aim of the present study was to examine the mediating role of problematic mukbang watching (PMW) on the relationships between depression and loneliness with problematic YouTube use (PYU). Results indicated that PMW was positively related to loneliness and PYU. Depression was positively and directly associated with PYU but was not associated with PMW. This was postulated that depression and loneliness would predict PMW and PYU, and that PMW would mediate the relationship between these psychosocial risk factors and PYU. Also hypothesized that those who were depressed and lonely would engage in excessive mukbang watching via using YouTube in order to alleviate negative feelings.  This study mainly explored the relationship between problem mukbang watching and depression and loneliness, and analyzed the psychological factors of watching mukbang videos. In other words, viewers chose mukbang videos to help them relieve negative feelings like depression and loneliness. | | | | |
|  | Participant details (eg, age/sex and number) | Age | | Ages ranged between 18 and 33 years | | |
|  |  | Sex | | Both | | |
|  |  | Final enrollment | | A total of 1,204 students began the survey and 604 of completed it. Of these, 217 were mukbang watchers (watched mukbang at least once before). | | |
| Details/results extracted from source of evidence | Review question addressed  (Tick) | 1. | The health-related elements in *mukbang* (Food ads) | | √ | |
|  |  | Source text | Mukbangers eat messy foods while slurping, chomping, and displaying bad table manners. | | | |
|  |  | 2. | The relationship between *mukbang* (Food ads) and health | | √ | |
|  |  | Source text | 1) Those who love food but do not want to suffer the negative consequences of excessive eating satisfy their needs by watching mukbang, which enables them to experience vicarious eating and satiation. Avoiding overeating to some extent.  2) Those who have different eating disorders (e.g., anorexia nervosa, binge eating) watch mukbang to overcome their problems for eating and food consumption, while some hospital patients who are unable to eat their desired food (e.g., unhealthy fast food) fulfill their eating urges by watching mukbang.  3) Mukbang watching for the purpose of dealing with negative emotions may be considered as problematic mukbang watching.  4) PMW was positively and directly associated with loneliness.  5) Mukbang watching is more prevalent among individuals presenting with eating disorder symptoms. For instance, individuals with bulimia and/or binge-eating disorders might engage in mukbang watching in order to perceive binge-eating massive meals as a normal or even healthy behavior.  6) For some individuals, excessive mukbang watching may constitute a potential addictive like behavior. | | | |
|  |  | 3. | Impacts may *mukbang* (Food ads) have on eating habits or eating behaviors | | NA | |
|  |  | Source text | NA | | | |
|  |  | 4. | The design, development, or usability of *mukbang* (Food ads) watching interventions to demonstrate the usefulness of *mukbang* in the health field | | | NA |
|  |  | Source text | NA | | | |
|  |  | 5. | Evaluation of the health effects of various types of *mukbang* (Food ads) | | | NA |
|  |  | Source text | NA | | | |
|  |  | 6. | The challenges and barriers of integrating *mukbang* (Food ads) videos into clinical practice | | | NA |
|  |  | Source text | NA | | | |
|  |  | 7. | The advantages and/or disadvantages of *mukbang* (Food ads) watching | | | √ |
|  |  | Source text | Advantages: mukbang watching might constitute a dysfunctional coping mechanism for some individuals, especially among individuals displaying high loneliness.  Disadvantages: problematic mukbang watching may cause problematic YouTube usage. | | | |
|  | Research method/Tools | 1) Demographic questionnaire  2) Problematic YouTube Use Scale (PYUS)  3) Mukbang Addiction Scale (MAS26)  4) Short Depression-Happiness Scale (SDHS35)  5) UCLA- Loneliness Scale-Short Form (ULS-436) | | | | |
|  | Characteristics of *mukbang* | NA | | | | |
|  | Health-related elements | Eating disorders  Problematic mukbang watching | | | | |
|  | Summary of key findings related to health | 1) It was found that depression and PMW were positively related to PYU. Loneliness was positively associated with PMW.  2) PMW is more likely to be engaged in to fullfill unattained social needs (and thus to cope with loneliness) than to compensate a depressive mood per se.  3) Mukbang enthusiasts can thus improve their feelings of social connectedness by watching mukbang and interacting with the content creators and viewers via a common interest, which is susceptible to alleviating their perceived feeling of loneliness.  4) This study showed that depression was positively associated with PYU, whereas loneliness was positively related to PMW. Furthermore, PMW was moderately associated with PYU. | | | | |

*Food ads must include images of celebrities eating.

20. Psychological research of mukbang audience from the perspective of use and satisfaction theory

|  | Items | Data | | | | |
| --- | --- | --- | --- | --- | --- | --- |
| Inclusion criteria | Population/Main research object | NA | | | | |
|  | Concept (Tick)  Studies focusing on | 1. | *mukbang* watching (Food ads*) | | √ | |
|  |  | 2. | the impact of *mukbang* (Food ads) on health | | √ | |
|  |  | 3. | the relationship between *mukbang* (Food ads) and health | | √ | |
|  |  | 4. | the design, development, or usability of *mukbang* (Food ads) watching interventions to demonstrate the usefulness of *mukbang* in the health field | | NA | |
|  |  | 5. | evaluating the effectiveness of various types of *mukbang* (Food ads) for public health | | NA | |
|  |  | 6. | the challenges and barriers of integrating *mukbang* (Food ads) videos into clinical practice | | NA | |
|  |  | 7. | the advantages and/or disadvantages of *mukbang* (Food ads) watching | | NA | |
|  | Types of evidence source | Commentary | | | | |
| Evidence source details and characteristics | Citation details (eg, author/s, date, title, journal, volume, issue, pages) | Title | | Psychological research of mukbang audience from the perspective of use and satisfaction theory | | |
|  |  | Journal | | West China Broadcast TV | | |
|  |  | Author/s | | Rong Tian | | |
|  |  | Date | | 2020 | | |
|  | Main content of the evidence | The rapid development of social media has given birth to mukbang, a new form of online video communication. Online mukbang has the characteristics of low entry threshold, spectacular content and instant interaction, which encourages many young groups to participate in the process of producing and sharing mukbang content text. Based on the use and satisfaction theory, this paper intends to deeply analyze the psychological needs of users who watch mukbang, and finally reflect on mukbang after examining the form of network communication. | | | | |
|  | Participant details (eg, age/sex and number) | Age | | NA | | |
|  |  | Sex | | NA | | |
|  |  | Final enrollment | | NA | | |
| Details/results extracted from source of evidence | Review question addressed  (Tick) | 1. | The health-related elements in *mukbang* (Food ads) | | √ | |
|  |  | Source text | Exaggerated eating - poor eating habits; Consuming large amounts of food at once; More flavorful food; Fake eating can induce vomiting. | | | |
|  |  | 2. | The relationship between *mukbang* (Food ads) and health | | √ | |
|  |  | Source text | 1) Mukbang videos can help people who are light and low fat to get a substitute satisfaction of heavy flavor video through substitutive tasting to help them maintain a healthy body.  2) Mukbang videos can bring strong pleasure and satisfaction to viewers, relieving their physical exhaustion and psychological pressure. | | | |
|  |  | 3. | Impacts may *mukbang* (Food ads) have on eating habits or eating behaviors | | NA | |
|  |  | Source text | NA | | | |
|  |  | 4. | The design, development, or usability of *mukbang* (Food ads) watching interventions to demonstrate the usefulness of *mukbang* in the health field | | | NA |
|  |  | Source text | NA | | | |
|  |  | 5. | Evaluation of the health effects of various types of *mukbang* (Food ads) | | | NA |
|  |  | Source text | NA | | | |
|  |  | 6. | The challenges and barriers of integrating *mukbang* (Food ads) videos into clinical practice | | | NA |
|  |  | Source text | NA | | | |
|  |  | 7. | The advantages and/or disadvantages of *mukbang* (Food ads) watching | | | NA |
|  |  | Source text | NA | | | |
|  | Research method/Tools | NA | | | | |
|  | Characteristics of *mukbang* | Mukbang has the characteristics of immediacy, low threshold, attention to sensory experience and novelty challenge. | | | | |
|  | Health-related elements | Mukbang sometimes conveys exaggerated eating and bad eating habits to the audience; Consuming large amounts of food at once; More flavorful food; Fake eating can induce vomiting. | | | | |
|  | Summary of key findings related to health | In this study, mukbang video is narrowly defined as Big Eater Mukbang. The anchor in the video conveys bad eating habits to the audience through exaggerated eating style, one-time intake of a large amount of heavy flavor or unhealthy food, and fake eating and vomiting. The content related to health factors in this paper mainly relates to mental health, and viewers watching mukbang can help them relieve pressure and fatigue in life. At the same time, for people who need to maintain a strict diet, mukbang can help them avoid eating high-calorie foods by substituting sexual satisfaction, so as to maintain a healthy body. | | | | |

21. A study on the motivation and influence of watching network mukbang programs

|  | Items | Data | | | | |
| --- | --- | --- | --- | --- | --- | --- |
| Inclusion criteria | Population/Main research object | Among the audience watching mukbang, the proportion of women is far more than that of men, and the age is generally younger. The student group is the largest, accounting for 60.6%. Office workers accounted for 28%; The freelance and non-working groups accounted for 9.7% and 1.7%, respectively. | | | | |
|  | Concept (Tick)  Studies focusing on | 1. | *mukbang* watching (Food ads*) | | √ | |
|  |  | 2. | the impact of *mukbang* (Food ads) on health | | √ | |
|  |  | 3. | the relationship between *mukbang* (Food ads) and health | | √ | |
|  |  | 4. | the design, development, or usability of *mukbang* (Food ads) watching interventions to demonstrate the usefulness of *mukbang* in the health field | | NA | |
|  |  | 5. | evaluating the effectiveness of various types of *mukbang* (Food ads) for public health | | NA | |
|  |  | 6. | the challenges and barriers of integrating *mukbang* (Food ads) videos into clinical practice | | NA | |
|  |  | 7. | the advantages and/or disadvantages of *mukbang* (Food ads) watching | | √ | |
|  | Types of evidence source | Master’s thesis | | | | |
| Evidence source details and characteristics | Citation details (eg, author/s, date, title, journal, volume, issue, pages) | Title | | A study on the motivation and influence of watching network mukbang programs | | |
|  |  | Journal | | CHENGDU SPORT UNIVERSITY | | |
|  |  | Author/s | | Yajuan Di | | |
|  |  | Date | | 2022-04-22 | | |
|  | Main content of the evidence | The purpose of this study is to systematically understand the viewing reasons, behavior and attitude of mukbang viewers on the background of the fervent development of mukbang and the normalization of weight loss. From the perspective and needs of the audience, this study tries to understand their motivation for watching mukbang and the practical problems behind it. It also makes a quantitative analysis of the impact of audience watching Mukbang, responds to the negative impact of Mukbang in previous studies, and makes an objective and reasonable judgment on Mukbang based on data analysis and research results.  It is found that the female audience watching mukbang is far more than the male audience, and the main audience is 18-34 years old. Understanding food information, relieving pressure, stimulating appetite and helping to lose weight are the four main reasons for audience to watch mukbang. Although losing weight is not the main reason for watching Mukbang, it is the universal willingness tendency of audience. mukbang has a certain degree of influence on the audience in three aspects: media following, eating attitude and purchasing and consumption, and there is a mutual influence relationship between the three behavioral attitudes. Media following behavior is also positively affected by viewing duration and attention duration. | | | | |
|  | Participant details (eg, age/sex and number) | Age | | All ages | | |
|  |  | Sex | | The gender ratio of the audience is 30.3% : 69.7%, and the audience of mukbang is mainly female. | | |
|  |  | Final enrollment | | 175 | | |
| Details/results extracted from source of evidence | Review question addressed  (Tick) | 1. | The health-related elements in *mukbang* (Food ads) | | √ | |
|  |  | Source text | There are problems in mukbang such as waste, falsehood, gimmicks and sensationalism. | | | |
|  |  | 2. | The relationship between *mukbang* (Food ads) and health | | √ | |
|  |  | Source text | 1）14.7% of the mukbang viewers believe that watching mukbang helps them release stress and forget their worries.  2）12.7% of the viewers thought mukbang made them feel full and helped them maintain or lose weight.  3）The audience thought that watching mukbang has slight influence on their opinions, attitudes and behaviors; The longer the audience's attention time and watching time, the greater the change in eating attitude by mukbang.  4）Stress is related to appetite, so watching mukbang videos can relieve stress. When they can't eat on their own, mukbang becomes a logical place to relieve stress, stimulate appetite and satisfy desires. | | | |
|  |  | 3. | Impacts may *mukbang* (Food ads) have on eating habits or eating behaviors | | √ | |
|  |  | Source text | 1）Mukbang is not the source of all evil to change the audience's eating behavior. The influence and mechanism brought by watching mukbang are insufficient, and the conclusion can be inferred that "Mukbang will trigger audience's behavior imitation and lead to overeating".  2）The longer the audience paid attention and watched, the greater the impact of mukbang on eating behavior. | | | |
|  |  | 4. | The design, development, or usability of *mukbang* (Food ads) watching interventions to demonstrate the usefulness of *mukbang* in the health field | | | NA |
|  |  | Source text | NA | | | |
|  |  | 5. | Evaluation of the health effects of various types of *mukbang* (Food ads) | | | NA |
|  |  | Source text | NA | | | |
|  |  | 6. | The challenges and barriers of integrating *mukbang* (Food ads) videos into clinical practice | | | NA |
|  |  | Source text | NA | | | |
|  |  | 7. | The advantages and/or disadvantages of *mukbang* (Food ads) watching | | | √ |
|  |  | Source text | Advantage: Mukbang video has the practical function of providing information and cultural output. Mukbang also has positive functions such as relieving pressure, cultural output and emotional comfort. | | | |
|  | Research method/Tools | Text analysis and questionnaire survey method | | | | |
|  | Characteristics of *mukbang* | The head anchors of mukbang are mainly those who are beautiful in appearance and eat a lot. In this study, mukbang videos are mainly short videos with a duration of 5-10 minutes, which are recorded in advance by anchors and mainly focus on eating. | | | | |
|  | Health-related elements | Mukbang video anchors eat a large amount of food without caring about high calories of the food, which is in sharp contrast to the current aesthetic concept of controlling diet, body shaping and fat reduction, and thinness as beauty. | | | | |
|  | Summary of key findings related to health | The research purpose of this paper is based on the background of the hot development of "eating and broadcasting programs" and the normalization of weight loss, to systematically understand the viewing reasons, viewing behavior and attitude influence of the audience of eating and broadcasting programs, and the quantitative analysis of the impact of the audience watching the eating and broadcasting program, in response to the negative impact of eating and broadcasting considered in previous studies, according to the data analysis and survey results. This article finally believes that mukbang is not the root of all evil that changes the audiences eating behavior. It is more like a "reflector" of real problems, a carrier born with contradictions and needs. It also has the practical function of providing information and cultural output; providing emotional value to relieve stress and troubleshoot. | | | | |

*Food ads must include images of celebrities eating.

22. Cloud-Based Commensality Enjoy the Company of Co-diners Without Social Facilitation of Eating

|  | Items | Data | | | | |
| --- | --- | --- | --- | --- | --- | --- |
| Inclusion criteria | Population/Main research object | 95 healthy Chinese young adults (mean age = 21.3 ± 2.5 years, ranging from 18 to 29 years; 43 males and 52 females) were recruited to take part in this study. | | | | |
|  | Concept (Tick)  Studies focusing on | 1. | *mukbang* watching (Food ads*) | | √ | |
|  |  | 2. | the impact of *mukbang* (Food ads) on health | | √ | |
|  |  | 3. | the relationship between *mukbang* (Food ads) and health | | √ | |
|  |  | 4. | the design, development, or usability of *mukbang* (Food ads) watching interventions to demonstrate the usefulness of *mukbang* in the health field | | NA | |
|  |  | 5. | evaluating the effectiveness of various types of *mukbang* (Food ads) for public health | | NA | |
|  |  | 6. | the challenges and barriers of integrating *mukbang* (Food ads) videos into clinical practice | | NA | |
|  |  | 7. | the advantages and/or disadvantages of *mukbang* (Food ads) watching | | √ | |
|  | Types of evidence source | RCT | | | | |
| Evidence source details and characteristics | Citation details (eg, author/s, date, title, journal, volume, issue, pages) | Title | | Cloud-Based Commensality: Enjoy the Company of Co-diners Without Social Facilitation of Eating | | |
|  |  | Journal | | Frontiers in Psychology | | |
|  |  | Author/s | | Chujun Wang, Yubin Peng, Linbo Qiu and Xiaoang Wan | | |
|  |  | Date | | 2021 | | |
|  | Main content of the evidence | “Digital commensality” to describe scenarios that enable a solo diner to have the feeling of eating with others via digital technologies, such as watching Mukbang or videoconferencing with other diners while eating alone in reality. It remains unclear how Mukbang-based and cloud-based commensality differ from each other, or from in-person commensality, in terms of the consequences on one’s emotional state, food intake, and food choices.  Hypothesis 1: In-person and cloud-based commensalities offer similar benefits of promoting social connectedness, whereas Mukbang-based commensality also has such social benefits but is less effective in reducing loneliness in comparison with in-person and cloud-based commensalities.  Hypothesis 2: Both in-person and Mukbang-based commensalities could potentially increase the probabilities of choosing unhealthy foods compared with solo dining; whereas cloud-based commensality might not have such detrimental effects. | | | | |
|  | Participant details (eg, age/sex and number) | Age | | Mean age = 21.3 ± 2.5 years, ranging from 18 to 29 years | | |
|  |  | Sex | | Both | | |
|  |  | Final enrollment | | Ninety-five healthy Chinese young adults (43 males and 52 females) were recruited to take part in this study. | | |
| Details/results extracted from source of evidence | Review question addressed  (Tick) | 1. | The health-related elements in *mukbang* (Food ads) | | √ | |
|  |  | Source text | Mukbang always maintain large portions of energy-dense foods. | | | |
|  |  | 2. | The relationship between *mukbang* (Food ads) and health | | √ | |
|  |  | Source text | 1) Mukbang-based commensality has been proved to be associated with a number of health risks, such as increasing food intake due to social comparison or mimicry, or underestimating the harmfulness of overindulgence.  2) Both in person and Mukbang-based commensalities were expected to significantly increase food intake compared with solitary eating.  3) Mukbang-based commensality could increase one’s intake of food, decrease the probability of choosing healthy foods, and reduce the pleasantness of eating healthy foods.  4) Reduce loneliness and to enhance the pleasantness of eating unhealthy foods. | | | |
|  |  | 3. | Impacts may *mukbang* (Food ads) have on eating habits or eating behaviors | | √ | |
|  |  | Source text | 1) Many viewers choose to watch Mukbang while they are eating alone in reality.  2) Mukbang watching may lead to imitative eating, which could lead to the formation of poor eating habits. | | | |
|  |  | 4. | The design, development, or usability of *mukbang* (Food ads) watching interventions to demonstrate the usefulness of *mukbang* in the health field | | | NA |
|  |  | Source text | NA | | | |
|  |  | 5. | Evaluation of the health effects of various types of *mukbang* (Food ads) | | | NAA |
|  |  | Source text | NA | | | |
|  |  | 6. | The challenges and barriers of integrating *mukbang* (Food ads) videos into clinical practice | | | NA |
|  |  | Source text | NA | | | |
|  |  | 7. | The advantages and/or disadvantages of *mukbang* (Food ads) watching | | | √ |
|  |  | Source text | Mukbang was not as good at alleviating loneliness as face-to-face meals or video meals based on online meetings.  Mukbang watching can significantly increase food intake compared with solitary eating. | | | |
|  | Research method/Tools | Different versions of photos of a Chinese young adult who was eating Asian noodles.  1) a young woman was eating when alone, 2) when having the company of two co-diners (of the same sex) using individual plates, 3) when watching Mukbang via a tablet computer, 4) when videoconferencing with two co-diners (of the same sex) via a tablet computer.  Food photos were sorted into two groups, including a group of unhealthy foods (i.e., pizza, cheeseburgers, cakes, and biscuits) and a group of healthy foods (i.e., vegetable salads, cod filet, fruit salads, and slices of apple). | | | | |
|  | Characteristics of *mukbang* | Mukbang refers to online broadcasts in which a host or hostess eats large portions of energy-dense foods on camera while interacting with online viewers. | | | | |
|  | Health-related elements |  | | | | |
|  | Summary of key findings related to health | 1) Mukbang can release loneliness when a person eats alone, but still carries the risk of increasing food intake and/or switching to unhealthy food choices.  2) if Mukbang viewers try to obtain vicarious satisfaction of a desire to eat unhealthy foods and urge themselves to engage in healthy eating, our results suggest that the outcomes are more likely to be opposite to what they wish for. | | | | |

*Food ads must include images of celebrities eating.

23. The differential effects of viewing short-form online culinary videos of fruits and vegetables versus sweet snacks on adolescents' appetites

|  | Items | Data | | | | |
| --- | --- | --- | --- | --- | --- | --- |
| Inclusion criteria | Population/Main research object | This study was conducted on adolescents between the ages of 12 and 14 years sampled from mainstream local schools. This age group was selected because they are developed enough cognitively to understand the effects of food choices on future health, even though their food choices are still largely socially influenced. | | | | |
|  | Concept (Tick)  Studies focusing on | 1. | *mukbang* watching (Food ads*) | | √ | |
|  |  | 2. | the impact of *mukbang* (Food ads) on health | | √ | |
|  |  | 3. | the relationship between *mukbang* (Food ads) and health | | √ | |
|  |  | 4. | the design, development, or usability of *mukbang* (Food ads) watching interventions to demonstrate the usefulness of *mukbang* in the health field | | √ | |
|  |  | 5. | evaluating the effectiveness of various types of *mukbang* (Food ads) for public health | | √ | |
|  |  | 6. | the challenges and barriers of integrating *mukbang* (Food ads) videos into clinical practice | | √ | |
|  |  | 7. | the advantages and/or disadvantages of *mukbang* (Food ads) watching | | NA | |
|  | Types of evidence source | A pre- and posttest study | | | | |
| Evidence source details and characteristics | Citation details (eg, author/s, date, title, journal, volume, issue, pages) | Title | | The differential effects of viewing short-form online culinary videos of fruits and vegetables versus sweet snacks on adolescents' appetites | | |
|  |  | Journal | | Appetite | | |
|  |  | Author/s | | Yandisa Ngqangashe, Charlotte J.S. De Backer | | |
|  |  | Date | | 2021-11-01 | | |
|  | Main content of the evidence | Given that there is a link between exposure to food content through food media and nutrient intake, our study aimed to investigate the effects of exposure to social media culinary videos on adolescents’ appetites. Conducted a pre- and posttest study to show a social media culinary video either demonstrating the preparation of a sweet snack (n = 50) or a fruit and vegetable snack (n = 76).  Examined hunger, general desire to eat, liking of the foods portrayed, intentions to eat and prepare the portrayed foods, and actual food choice behavior as dependent variables.  The findings showed that the videos had no effects on hunger or general desire to eat but influenced food choice behavior, liking of the foods, and intentions to eat and prepare the foods portrayed. The sweet snacks video reduced the liking of fruits and vegetables and indirectly reduced the odds of choosing a fruit over a cookie, through intentions to eat sweet snacks. The fruits and vegetables video reduced the liking of sweet snacks and resulted in higher intentions to prepare healthy snacks.  First research question (RQ1) of this study is: Does exposure to food content through social media culinary videos increase self-reported hunger levels?  The research questions below pertain to the effect of the videos on the general desire to eat:  RQ2: Does exposure to food cues through social media culinary videos induce a general desire to eat?  RQ3: Does the self-reported general desire to eat reflect the content of the social media culinary video that was watched? | | | | |
|  | Participant details (eg, age/sex and number) | Age | | The mean age was 13.9 years, (SD = 1.2) | | |
|  |  | Sex | | Both, of which 62% of the participants were females. | | |
|  |  | Final enrollment | | 126 middle school children | | |
| Details/results extracted from source of evidence | Review question addressed  (Tick) | 1. | The health-related elements in *mukbang* (Food ads) | | √ | |
|  |  | Source text | 1) The foods portrayed on these platforms are not healthy.  2) Food cue reactivity theory: exposure to food cues increases food intake (Jansen, 1998), even in the absence of hunger (Passamonti et al., 2009). Mukbang contains food cues, so mukbang watching may cause unexpected food intake. | | | |
|  |  | 2. | The relationship between *mukbang* (Food ads) and health | | √ | |
|  |  | Source text | 1) Unhealthy items: social media images of food encourage distorted perceptions of food.  2) Other short-form culinary videos may be following similar trends, as demonstrated by a nutrient content analysis of recipes portrayed on BuzzFeed’s Tasty videos, which fall short of the recommendations for healthy diets (Ngqangashe, 2019).  3) Watching TV cooking shows increases children's intake, and the increased intake is often unhealthy food.  4) The notion that short-form social media culinary videos may be a potential platform to endorse healthy eating behaviors.  5) Exposure to the food advertising which contains unhealthy contents has potential harmful effects.  6) While the effects of the fruits and vegetables video were not as extensive as those of the sweet snacks video, they demonstrate the potential of short-form culinary videos as platforms to reduce the liking of unhealthy food and spark interest in preparing healthy food. | | | |
|  |  | 3. | Impacts may *mukbang* (Food ads) have on eating habits or eating behaviors | | √ | |
|  |  | Source text | 1) The dominant portrayal of unhealthy food on these platforms is concerning, given that exposure to food cues influences eating behaviors.  2) Online food content has effects on food choice.  3) The positive effects of healthier food content related to TV cooking shows are different than those found in studies on advergames and ad placements, which claim that healthier food content did not significantly shift eating behaviors (Folkvord et al., 2013; Naderer et al., 2018).  4) Exposure to food cues from advertising, food placements, and TV cooking shows have effects on immediate food choice behavior.  5) The sweet snacks video not only affected intentions to eat sweet snacks but also reduced the liking of fruits and vegetables. | | | |
|  |  | 4. | The design, development, or usability of *mukbang* (Food ads) watching interventions to demonstrate the usefulness of *mukbang* in the health field | | | √ |
|  |  | Source text | 1) Public health practitioners and media content creators must cocreate healthy food content that promotes food literacy while preserving entertaining and visual elements.  2) Short-form culinary videos can be used to stimulate interest in cooking and the appeal of healthy food. This approach is supported by earlier studies on the positive effects of video programs designed by nutritionists on food choice behavior and recent studies on the effects of TV cooking shows that endorse fruits and vegetables. | | | |
|  |  | 5. | Evaluation of the health effects of various types of *mukbang* (Food ads) | | | √ |
|  |  | Source text | 1) The fruits and vegetables video had no effect on food choice behavior, while the sweet snacks video indirectly reduced the odds of choosing a fruit, mediated by intentions to eat sweet snacks. | | | |
|  |  | 6. | The challenges and barriers of integrating *mukbang* (Food ads) videos into clinical practice | | | √ |
|  |  | Source text | Food media is dominated by sweet snacks/ unhealthier content, which has more appeal for younger audiences. So if scholars want to use food related videos into clinical practice, they need to change the video content to promote healthy eating content. | | | |
|  |  | 7. | The advantages and/or disadvantages of *mukbang* (Food ads) watching | | | NA |
|  |  | Source text | NA | | | |
|  | Research method/Tools | A school-based experimental study.  Randomization was at the school level; the schools were randomly assigned to either the group that watched a video with fruits and vegetables or a group that watched a video on sweet snacks.  Stimuli:  Short, fast-paced video clips that demonstrate food preparation with the camera focused on the food and utensils.  Sweet snacks: a Tasty clip that portrayed preparation of “The Best Fudgy Brownies Ever,” “Chocolate Peanut Brownies (Buckeye Brownies),” and “6 Ways to Make Better Boxed Brownies.”  Fruits and vegetable condition: a Tasty video that showed the preparation of “4 Make-Ahead Vegetable-Packed Smoothies,” “4 Healthier Desserts,” and “Fruit Salad Four Ways.”  For both conditions, the videos were kept as they were posted on Tasty, including the sound and visuals, and were compiled to be the same duration (4 min 17 s). | | | | |
|  | Characteristics of *mukbang* | Short-form social media culinary videos, the platforms include including Facebook, Instagram, YouTube, and Twitter. | | | | |
|  | Health-related elements | Fruit and vegetables  Dessert refers to unhealthy food | | | | |
|  | Summary of key findings related to health | 1) A number of previous studies have shown that exposure to food cues through various media platforms has effects on food related outcomes.  2) Exposure to short-form culinary videos has effects on food choice behavior, liking the foods portrayed, and intentions to eat and prepare the foods portrayed.  3) Exposure to the fruits and vegetables video reduced liking of sweet snacks.  4) People exposed to food videos featuring fruits and vegetables were more likely to choose fruits and vegetables than those exposed to food videos featuring desserts. This means the content of food related videos have effects on food choices in real life. | | | | |

*Food ads must include images of celebrities eating.

24. The rise of Digital Table A review of foreign researches on mukbang

|  | Items | Data | | | | |
| --- | --- | --- | --- | --- | --- | --- |
| Inclusion criteria | Population/Main research object | NA | | | | |
|  | Concept (Tick)  Studies focusing on | 1. | mukbang watching (Food ads*) | | √ | |
|  |  | 2. | the impact of mukbang (Food ads) on health | | √ | |
|  |  | 3. | the relationship between mukbang (Food ads) and health | | √ | |
|  |  | 4. | the design, development, or usability of mukbang (Food ads) watching interventions to demonstrate the usefulness of mukbang in the health field | | NA | |
|  |  | 5. | evaluating the effectiveness of various types of mukbang (Food ads) for public health | | √ | |
|  |  | 6. | the challenges and barriers of integrating mukbang (Food ads) videos into clinical practice | | NA | |
|  |  | 7. | the advantages and/or disadvantages of mukbang (Food ads) watching | | √ | |
|  | Types of evidence source | A systematic review | | | | |
| Evidence source details and characteristics | Citation details (eg, author/s, date, title, journal, volume, issue, pages) | Title | | The rise of Digital Table: A review of foreign researches on mukbang | | |
|  |  | Journal | | News Research | | |
|  |  | Author/s | | Mengjie Ma, Jiani Wang | | |
|  |  | Date | | 2021 | | |
|  | Main content of the evidence | By combing through foreign literatures about mukbang, this study analyzed the audience's motivation to watch mukbang from the aspects of society, entertainment and diet. The essence of mukbang is a "digital table". People can narrow the social isolation space by watching mukbang videos. The entertainment satisfaction of mukbang is mainly reflected in helping the audience get sensory satisfaction and fun through sharing the eating experience. The positive impact of watching mukbang is that through the establishment of online virtual community, the audience can overcome the physical distance, form the feeling of common presence, and promote the emotional and psychological connection with others; The negative effect is to change the audience's food preferences, eating habits, table manners, etc., leading to disordered eating. | | | | |
|  | Participant details (eg, age/sex and number) | Age | | NA | | |
|  |  | Sex | | NA | | |
|  |  | Final enrollment | | NA | | |
| Details/results extracted from source of evidence | Review question addressed  (Tick) | 1. | The health-related elements in mukbang (Food ads) | | √ | |
|  |  | Source text | 1）On YouTube, about 83.5% of mukbang videos fall into the category of binge eating, and 90% of the food is take-out food purchased from convenience stores and restaurants.  2）In terms of food choices, about 15.7% of mukbang hosts chose to eat fast food and junk food, and about 5.6% of mukbang hosts chose to eat extremely spicy or stimulating food. | | | |
|  |  | 2. | The relationship between mukbang (Food ads) and health | | √ | |
|  |  | Source text | 1）Psychological health: People can relieve the stress of social isolation - feelings of loneliness, stress, and negative emotions - by watching mukbang videos. Positive comments about mukbang and the food can lead to better feelings of community and positive emotions in people who watch mukbang.  2）As a means of physical health, avoiding overeating: Watching mukbang can satisfy these people's food cravings, experience the pleasure of overeating, and gain vicarious satisfaction through visual and auditory stimuli. The key role of watching mukbang is to replace eating. Some dieters watch mukbang to curb their desire to consume while avoiding actually eating.  3）Mukbang unconsciously promotes overeating, and its content may subtly beautify the behavior of overeating, leading to blind imitation by the audience, resulting in obesity and different degrees of eating disorders.  4）The popularity of Mukbang may have certain negative effects on society, such as increasing overweight/obesity rates across the region.  5）The contrast between mukbang host’s slim figure and huge food intake distorts viewers' perceptions of food consumption and weight loss, creating an impression of its traditional health tube. | | | |
|  |  | 3. | Impacts may mukbang (Food ads) have on eating habits or eating behaviors | | √ | |
|  |  | Source text | 1）Watching mukbang may lead viewers to imitate the poor eating behaviors of mukbang anchors, thereby changing viewers' perceptions of food culture, table manners, etc.  2）Some mukbang videos may have problems promoting unhealthy eating habits and foods to viewers.  3）The mukbang video influenced viewers' food choices. The food in mukbang is sometimes nutrient-poor food such as fast food, junk food, frozen food, which can easily lead to the audience's banditry.  4）Mukbang anchor's eating behavior could subtly affect the audience, making them believe that excessive eating is normal, and then start to imitate mukbang anchor's eating habits. | | | |
|  |  | 4. | The design, development, or usability of mukbang (Food ads) watching interventions to demonstrate the usefulness of mukbang in the health field | | | NA |
|  |  | Source text | NA | | | |
|  |  | 5. | Evaluation of the health effects of various types of mukbang (Food ads) | | | √ |
|  |  | Source text | Mukbang anchors often exhibit bad eating styles and behaviors, grabbing food, and communicating with viewers while their mouths are full of food. | | | |
|  |  | 6. | The challenges and barriers of integrating mukbang (Food ads) videos into clinical practice | | | NA |
|  |  | Source text | NA | | | |
|  |  | 7. | The advantages and/or disadvantages of mukbang (Food ads) watching | | | √ |
|  |  | Source text | Advantages：By establishing online virtual communities, audiences can overcome physical distance, form a sense of common presence, and promote emotional and psychological connection with others;  Disadvantages：Changing the audience's food preferences, eating habits, table manners, etc., leading to disordered eating. | | | |
|  | Research method/Tools | A systematic review | | | | |
|  | Characteristics of mukbang | Mukbang originally referred to live food shows, where hosts eat large amounts of food while interacting with the audience. Mukbang focuses on the food experience, including visual displays of biting food, amplification of eating sounds, and more. | | | | |
|  | Health-related elements | NA | | | | |
|  | Summary of key findings related to health | This paper has analyzed the motives of mukbang viewers from the aspects of society, entertainment, diet et al. by sorting out the foreign literatures on mukbang. This study regarded mukbang as a "digital dining table" where people can watch it to reduce the space for social isolation; The entertainment satisfaction of mukbang was mainly reflected in helping the audience to obtain sensory satisfaction and fun by sharing the eating experience. Watching mukbang also promotes emotional and psychological connection with other. The negative effect of mukbang in this study was to change the audience's food preferences, eating habits and table manners, etc., leading to disordered eating. | | | | |

25. Problematic Mukbang Watching and Its Relationship to Disordered Eating and Internet Addiction A Pilot Study Among Emerging Adult Mukbang Watchers

|  | Items | Data | | | | |
| --- | --- | --- | --- | --- | --- | --- |
| Inclusion criteria | Population/Main research object | This study tested a structural equation model to examine the predictive role of problematic mukbang watching on disordered eating and internet addiction.  Hypothesis: being frequently exposed to visual and audio stimuli of excessive eating (with the mukbangers’ augmented demonstration of pleasure and satisfaction received from eating) would manipulate viewers’ real-life practices of eating and relationship with food, which would exacerbate disordered eating. | | | | |
|  | Concept (Tick)  Studies focusing on | 1. | *mukbang* watching (Food ads*) | | √ | |
|  |  | 2. | the impact of *mukbang* (Food ads) on health | | √ | |
|  |  | 3. | the relationship between *mukbang* (Food ads) and health | | √ | |
|  |  | 4. | the design, development, or usability of *mukbang* (Food ads) watching interventions to demonstrate the usefulness of *mukbang* in the health field | | NA | |
|  |  | 5. | evaluating the effectiveness of various types of *mukbang* (Food ads) for public health | | √ | |
|  |  | 6. | the challenges and barriers of integrating *mukbang* (Food ads) videos into clinical practice | | NA | |
|  |  | 7. | the advantages and/or disadvantages of *mukbang* (Food ads) watching | | √ | |
|  | Types of evidence source | Cross-sectional study, questionnaire survey. | | | | |
| Evidence source details and characteristics | Citation details (eg, author/s, date, title, journal, volume, issue, pages) | Title | | Problematic Mukbang Watching and Its Relationship to Disordered Eating and Internet Addiction: A Pilot Study Among Emerging Adult Mukbang Watchers | | |
|  |  | Journal | | International Journal of Mental Health and Addiction | | |
|  |  | Author/s | | Kagan Kircaburun & Cemil Yurdagül & Daria Kuss & Emrah Emirtekin & Mark D. Griffiths | | |
|  |  | Date | | 2021-12-01 | | |
|  | Main content of the evidence | This study mainly investigated the relationship of problematic mukbang watching with disordered eating and internet addiction, found that problematic mukbang watching was positively associated with both disordered eating and internet addiction.  This study involves two different concepts regarding mukbang watching, one is recreational mukbang watching, another is problematic mukbang watching. What’s more, the research has pointed out that problematic mukbang watching is more likely to be associated with more severe negative consequences of mukbang watching compared with recreational mukbang watching, problematic mukbang watching is also more likely to relate to increased real-life problematic eating practices (i.e., disordered eating). | | | | |
|  | Participant details (eg, age/sex and number) | Age | | 19‐29 years | | |
|  |  | Sex | | Both | | |
|  |  | Final enrollment | | Inclusion criteria: mukbang viewers who watched mukbang at least once in the past 30 days and completed an online survey.  A total of 952 students began the survey and 312 of them completed it (response rate 33%). Of these, 140 were mukbang viewers (66% female, Mage = 21.66, SD = 1.88, range = 19‐29 years). | | |
| Details/results extracted from source of evidence | Review question addressed  (Tick) | 1. | The health-related elements in *mukbang* (Food ads) | | √ | |
|  |  | Source text | The sharp contrast between slim figure of mukbangers and their huge food intake. | | | |
|  |  | 2. | The relationship between *mukbang* (Food ads) and health | | √ | |
|  |  | Source text | Psychological health: mukbang viewers can obtain a sense of relief and pleasure from listening to eating sounds such as chewing and devouring noises.  Addiction-like symptoms: some individuals might demonstrate addiction-like symptoms associated with their mukbang watching (i.e., salience, tolerance, relapse, conflict, mood modification, withdrawal), and that the frequency of daily time spent watching mukbang was moderately related to problematic mukbang watching.  Overeating: individuals that frequently watch mukbang may consume more than they normally would because individuals’ consumption norms could easily be affected by others’ consumption and mukbangers typically eat very large portions of food during a single broadcast.  Problematic mukbang watching was positively correlated with disordered eating and internet addiction. | | | |
|  |  | 3. | Impacts may *mukbang* (Food ads) have on eating habits or eating behaviors | | √ | |
|  |  | Source text | Reduce the likelihood of developing poor eating habits: watching mukbang enables viewers to satisfy their food cravings, experience the feeling of binge eating themselves, and experiencing vicarious satiation via visual and audio stimulation from the mukbangers themselves.  Producing bad eating behaviors: watching mukbang was damaging teenagers’ and younger viewers’ eating behaviors by modeling maladaptive behavior (e.g., binge eating) and perceiving it socially acceptable. | | | |
|  |  | 4. | The design, development, or usability of *mukbang* (Food ads) watching interventions to demonstrate the usefulness of *mukbang* in the health field | | | NA |
|  |  | Source text | NA | | | |
|  |  | 5. | Evaluation of the health effects of various types of *mukbang* (Food ads) | | | √ |
|  |  | Source text | Recreational mukbang watching & problematic mukbang watching. | | | |
|  |  | 6. | The challenges and barriers of integrating *mukbang* (Food ads) videos into clinical practice | | | NA |
|  |  | Source text | NA | | | |
|  |  | 7. | The advantages and/or disadvantages of *mukbang* (Food ads) watching | | | √ |
|  |  | Source text | Advantages: mukbang can fulfill several psychological needs among individuals, watching mukbang alleviated viewers’ real-life loneliness and social isolation by making them feel emotionally connected to other viewers and the mukbanger.  Disadvantages: watching mukbang as a maladaptive coping strategy适应不良的应对方式 to deal with real-life eating urges may also result in adverse consequences. | | | |
|  | Research method/Tools | Mukbang Addiction Scale (MAS): using to assess problematic mukbang watching.  Internet Addiction Scale (IAS): using to assess internet addiction (e.g., “How often during the last year have you tried to cut down on the use of internet without success?”).  SCOFF Eating Disorders Scale: assess risk of eating disorder symptoms including anorexia nervosa, bulimia nervosa, body dissatisfaction, and unspecified eating disorders. | | | | |
|  | Characteristics of *mukbang* | Watching others eat food and socialize via eating broadcast that are known as “mukbang”. Mukbang is a portmanteau term that comprises the South Korean words “eating” (“meokneun”) and “broadcast” (“bangsong”). At present (2019), mukbang videos are watched by millions of viewers across the world via a variety of internet applications including different social networking sites and online streaming channels. | | | | |
|  | Health-related elements | Disordered eating: e.g., anorexia nervosa, bulimia nervosa, and binge eating disorder. One of the notable health problems among adolescents and emerging adults. Any type of eating disorder has detrimental health consequences.  Internet addiction: internet addiction has well established adverse consequences upon individuals’ mental health and physical wellbeing similar to the negative effects that arise from substance-related addictions including psychopathology, insomnia, poor sleep quality, academic failure, and experiencing family and relationship problems  Problematic mukbang watching: the excessive mukbang watching based on obtaining satisfaction or entertainment. | | | | |
|  | Summary of key findings related to health | 1) The double-sided nature of mukbang videos: mukbang provides some viewers with the vicarious pleasure of eating the desired and fantasized food so that they could avoid actually eating the food (i.e., watching mukbang as a dieting tool). For those who are fat and need to lose weight to keep health, mukbang is a good way to provide vicarious pleasure, which may make losing weight much easier for the people who cannot stop from eating. But for those who strive for the ultimate slim figure, this effect may cause excessive diet and lead to malnutrition.  2) Watching mukbang unduly can lead to binge-eating, bulimia nervosa and anorexia nervosa, as well as to viewers' biased notions of healthy eating, such as believing that binge-eating does not cause health problems, which can lead to eating disorders such as overeating.  3) Another detrimental impact of dealing with the real-life eating urges by watching others eat food may be that it negatively affects an individual’s relationship with real-life eating.  4) Unrealistic visual and audio stimuli demonstrated in mukbang videos (augmented acts of pleasure and satisfaction shown by the mukbangers while eating can lead to diminished pleasure received from actual eating, and in turn, promote disordered eating (e.g., anorexia nervosa) through the displacement of real eating with virtual eating.  5) Spending excessive time watching mukbang and being preoccupied with watching mukbang to facilitate mood modification could lead to the onset, development, and maintenance of different eating disorders and generalized internet addiction among a small minority of individuals. | | | | |

*Food ads must include images of celebrities eating.

26. Watching a remote-video confederate eating facilitates perceived taste and consumption of food

|  | Items | Data | | | | |
| --- | --- | --- | --- | --- | --- | --- |
| Inclusion criteria | Population/Main research object | All participants were naïve to the experiment ’s purpose. They had good health, no food allergies, no history of eating disorders, no special dietary restrictions, and reported being within normal weight.  The videos depicted three conditions: 1) the eating condition, 2) the calling condition, and 3) the absence condition. The videos were not mukbang videos in the actual sense, but have same characteristics with mukbang and can be likened to mukbang videos. | | | | |
|  | Concept (Tick)  Studies focusing on | 1. | *mukbang* watching (Food ads*) | | NA | |
|  |  | 2. | the impact of *mukbang* (Food ads) on health | | √ | |
|  |  | 3. | the relationship between *mukbang* (Food ads) and health | | √ | |
|  |  | 4. | the design, development, or usability of *mukbang* (Food ads) watching interventions to demonstrate the usefulness of *mukbang* in the health field | | NA | |
|  |  | 5. | evaluating the effectiveness of various types of *mukbang* (Food ads) for public health | | NA | |
|  |  | 6. | the challenges and barriers of integrating *mukbang* (Food ads) videos into clinical practice | | NA | |
|  |  | 7. | the advantages and/or disadvantages of *mukbang* (Food ads) watching | | √ | |
|  | Types of evidence source | Clinical intervention study | | | | |
| Evidence source details and characteristics | Citation details (eg, author/s, date, title, journal, volume, issue, pages) | Title | | Watching a remote-video confederate eating facilitates perceived taste and consumption of food | | |
|  |  | Journal | | Physiology & Behavior | | |
|  |  | Author/s | | Nobuyuki Kawai, Zhuogen Guo a, Ryuzaburo Nakata | | |
|  |  | Date | | 2021-09-01 | | |
|  | Main content of the evidence | This study compared three types of silent videos: 1) a stranger eating potato chips, 2) the stranger calling on the phone, or 3) only objects (food and cellphone). Participants perceived popcorn to taste better only when they watched the video of others eating. Watching others eating induced the participants to eat more than when watching the other two videos. This study indicates that remote-video confederates enhance not only food intake but also the perceived taste of food.  In this study, the researchers have investigated whether watching a silent video of others eating amplified preferences for food and food intake compared to watching silent videos of others’ non-food related behavior or objects. | | | | |
|  | Participant details (eg, age/sex and number) | Age | | Age ranged from 19 to 26 years (mean = 20.75, SD = 1.92). | | |
|  |  | Sex | | Both | | |
|  |  | Final enrollment | | Twenty-four students (13 women) participated in this experiment. | | |
| Details/results extracted from source of evidence | Review question addressed  (Tick) | 1. | The health-related elements in *mukbang* (Food ads) | | NA | |
|  |  | Source text | NA | | | |
|  |  | 2. | The relationship between *mukbang* (Food ads) and health | | √ | |
|  |  | Source text | 1) Mukbang-like-videos watching can get a person to eat more, which reflects a food intake adjustment to match that of one’s dining companion.  2) Digital commensality could have some health-compromising effects, especially for other age groups like adolescent, because young adulthood is a sensitive developmental period with regard to weight gain.  3) Social facilitation of eating may lead to overeating that facilitates weight gain.  4) Mukbang watching has benefits on feeling of loneliness.  5) Watching others eat increases intake and makes food taste better. | | | |
|  |  | 3. | Impacts may *mukbang* (Food ads) have on eating habits or eating behaviors | | √ | |
|  |  | Source text | 1) Watching another person eat on the media/screen can increase the viewer's perception of the food they are eating, making it more palatable. | | | |
|  |  | 4. | The design, development, or usability of *mukbang* (Food ads) watching interventions to demonstrate the usefulness of *mukbang* in the health field | | | NA |
|  |  | Source text | NA | | | |
|  |  | 5. | Evaluation of the health effects of various types of *mukbang* (Food ads) | | | NA |
|  |  | Source text | NA | | | |
|  |  | 6. | The challenges and barriers of integrating *mukbang* (Food ads) videos into clinical practice | | | NA |
|  |  | Source text | NA | | | |
|  |  | 7. | The advantages and/or disadvantages of *mukbang* (Food ads) watching | | | √ |
|  |  | Source text | Advantages: release loneliness, increases food intake (for those who eat too little) and makes food taste better.  Disadvantages: lead to overeating that facilitates weight gain, or even become obesity. | | | |
|  | Research method/Tools | In the first, a person ate potato chips with a cellphone placed on a desk. In the second, the same person made a call instead of eating potato chips on a desk. In the third, a bag of potato chips and the cellphone on a desk were shown without a person. The videos depicted three conditions: 1) the eating condition, 2) the calling condition, and 3) the absence condition. | | | | |
|  | Characteristics of *mukbang* | This may be one reason for the rise of digital commensality such as mukbang, which refers to an online video broadcast in which a person (a broadcast jockey) consumes food alone while the audience observes and also eats alone. | | | | |
|  | Health-related elements | NA | | | | |
|  | Summary of key findings related to health | 1)Previous research has suggested that an image of a person eating strongly influences eating behavior.  2) This study demonstrated that watching others eating enhanced not only food intake but also perceived food taste. | | | | |

*Food ads must include images of celebrities eating.

27. Get out of the emotional hunger——Psychological and behavioral analysis of the popularity of mukbang

|  | Items | Data | | | | |
| --- | --- | --- | --- | --- | --- | --- |
| Inclusion criteria | Population/Main research object | Taking the current popular mukbang video as the main object of review, this paper analyzes the reasons for the hot mukbang video in China, and points out the unhealthy information revealed behind the mukbang video. | | | | |
|  | Concept (Tick)  Studies focusing on | 1. | mukbang watching (Food ads*) | | √ | |
|  |  | 2. | the impact of mukbang (Food ads) on health | | √ | |
|  |  | 3. | the relationship between mukbang (Food ads) and health | | √ | |
|  |  | 4. | the design, development, or usability of mukbang (Food ads) watching interventions to demonstrate the usefulness of mukbang in the health field | | NA | |
|  |  | 5. | evaluating the effectiveness of various types of mukbang (Food ads) for public health | | NA | |
|  |  | 6. | the challenges and barriers of integrating mukbang (Food ads) videos into clinical practice | | NA | |
|  |  | 7. | the advantages and/or disadvantages of mukbang (Food ads) watching | | √ | |
|  | Types of evidence source | Journal paper | | | | |
| Evidence source details and characteristics | Citation details (eg, author/s, date, title, journal, volume, issue, pages) | Title | | Get out of the emotional hunger——Psychological and behavioral analysis of the popularity of mukbang | | |
|  |  | Journal | | Psychology and Health | | |
|  |  | Author/s | | Yu Li | | |
|  |  | Date | | 2020 | | |
|  | Main content of the evidence | This study has analyzed the reasons for the popularity of mukbang video in China, which are as follows: (1) Activating taste buds and obtaining the experience of liberating appetite; (2) Self-projection, the experience of vicarious satisfaction; ③ Intimate interaction, obtain the experience of belonging and companionship, and ④ audience perspective, obtain the experience of novelty judgment. At the same time, it also points out that there is harmful information in mukbang videos popular in China, such as fake eating to induce vomiting, wasting food and damaging health at the same time, including overeating, etc. This harmful information will imperceptively affect mukbang viewers, destroy their original eating habits, and harm their health in the long run. | | | | |
|  | Participant details (eg, age/sex and number) | Age | | NA | | |
|  |  | Sex | | NA | | |
|  |  | Final enrollment | | NA | | |
| Details/results extracted from source of evidence | Review question addressed  (Tick) | 1. | The health-related elements in mukbang (Food ads) | | √ | |
|  |  | Source text | The content of mukbang is rich and varied, including many unhealthy and spicy stunts, such as the host's incredible appetite, a few minutes "storm" to inhale a large pot of ramen, and eat 100 sausages in one sitting; Some imagination surprise, eat raw octopus sea urchin, dry to eat hot pot bottom material.  Big Eater Mukbang: fake eating, vomiting and severe obesity. | | | |
|  |  | 2. | The relationship between mukbang (Food ads) and health | | √ | |
|  |  | Source text | 1）The vicariously satisfying experience gained from watching mukbang can help viewers with special dietary needs maintain their special diets (such as low salt and low fat diets for people with high blood pressure, low bacteria diets for patients undergoing chemotherapy for blood disorders, low fat diets for people who are losing weight, etc.) so as to avoid damaging their health by craving certain foods.  2）The alternative satisfaction mechanism can also help the viewer maintain psychological balance and prevent excessive conflict and struggle when the actual needs cannot be met.  3）Receiving harmful health information: The audience in front of the screen is watching the video to meet their own needs, and it is easy to accept the "harmful" information conveyed by such videos in the ear and eyes. Some mukbang videos have had a negative impact on viewers with their unhealthy views on eating.  4）Unhealthy dietary behaviors | | | |
|  |  | 3. | Impacts may mukbang (Food ads) have on eating habits or eating behaviors | | √ | |
|  |  | Source text | 1）The victual satisfaction gained from watching mukbang can help viewers maintain healthier eating habits: mukbang videos replace their own junk food/fast food, while maintaining healthy eating habits, thus satisfying their cravings for junk food/heavy food/fast food while maintaining a healthy diet.  2）People usually gain vicarious experience by observing others' behavior, and change their own cognition and behavior imperceptibly. When watching bad mukbang videos becomes the norm, people will naturally unconsciously form bad habits. | | | |
|  |  | 4. | The design, development, or usability of mukbang (Food ads) watching interventions to demonstrate the usefulness of mukbang in the health field | | | NA |
|  |  | Source text | NA | | | |
|  |  | 5. | Evaluation of the health effects of various types of mukbang (Food ads) | | | NA |
|  |  | Source text | NA | | | |
|  |  | 6. | The challenges and barriers of integrating mukbang (Food ads) videos into clinical practice | | | NA |
|  |  | Source text | NA | | | |
|  |  | 7. | The advantages and/or disadvantages of mukbang (Food ads) watching | | | √ |
|  |  | Source text | Advantages：1）Promote appetite and increase the audience's taste perception: Many people like to open mukbang at the meal point, watching the anchor eat a lot of high-calorie food, and they also feel that the appetite increases, and the light food becomes more delicious. Some anchors will deliberately amplify the sound of tearing and chewing food, stimulating the audience's senses in a full range, and the activation of appetite is more obvious.  2）Virtual companionship: In mukbang, the audience can send real-time comments and communicate with the host, and the communication content is very life, just like chatting with family and friends at the dinner table. Watching mukbang has become an important way for some people to get company. | | | |
|  | Research method/Tools | NA | | | | |
|  | Characteristics of mukbang | The mukbang mentioned in this paper are mainly Big Eater Mukbang and curiosity hunting mukbang. Both of these mukbang videos contain unhealthy information, such as fake eating, vomiting, overeating, and eating some abnormal and extremely irritating food. | | | | |
|  | Health-related elements | Mental health: vicarious satisfaction — maintaining mental balance and avoiding excessive conflict and struggle;  Physical health: vicarious satisfaction — maintaining the audience's own healthy diet and satisfying their cravings for junk food through "watching" and "listening";  Fake eating, vomiting, overeating, promoting unhealthy eating behaviors and concepts, causing potential health threats to the audience. | | | | |
|  | Summary of key findings related to health | This article analyzed the reasons for the popularity of mukbang in China, such as activating viewers’ gustation, obtaining the experience of alternative satisfaction and intimating interaction. Meanwhile, this article also pointed out that the harmful information in the popular mukbang video in China, such as fake eating and vomiting, not only wasted food but also costed to one’s health. What’s more, this harmful information also invisibly affected the mukbang viewers, destroyed their original eating habits and health of the audience over time. | | | | |

*Food ads must include images of celebrities eating.

28. Watching a food-related television show and caloric intake. A laboratory study

|  | Items | Data | | | | |
| --- | --- | --- | --- | --- | --- | --- |
| Inclusion criteria | Population/Main research object | Food shows; Eating behaviors; Sweet consumption  RCT  Participants (N = 80) were recruited from psychology classes at a small college in the northeast. | | | | |
|  | Concept (Tick)  Studies focusing on | 1. | *mukbang* watching (Food ads*) | | √ | |
|  |  | 2. | the impact of *mukbang* (Food ads) on health | | √ | |
|  |  | 3. | the relationship between *mukbang* (Food ads) and health | | √ | |
|  |  | 4. | the design, development, or usability of *mukbang* (Food ads) watching interventions to demonstrate the usefulness of *mukbang* in the health field | | NA | |
|  |  | 5. | evaluating the effectiveness of various types of *mukbang* (Food ads) for public health | | NA | |
|  |  | 6. | the challenges and barriers of integrating *mukbang* (Food ads) videos into clinical practice | | NA | |
|  |  | 7. | the advantages and/or disadvantages of *mukbang* (Food ads) watching | | √ | |
|  | Types of evidence source | An experimental study | | | | |
| Evidence source details and characteristics | Citation details (eg, author/s, date, title, journal, volume, issue, pages) | Title | | Watching a food-related television show and caloric intake. A laboratory study | | |
|  |  | Journal | | Appetite | | |
|  |  | Author/s | | Jamie S. Bodenlos, Bernadette M. Wormuth | | |
|  |  | Date | | 2013-02 | | |
|  | Main content of the evidence | Based upon previous research, if food advertisements can affect children and adult’s eating behaviors then food television programs should have a similar impact. In the present study, an experimental design was used to examine if watching a television program on food, a cooking show, affected caloric intake. It was hypothesized that participants who were exposed to the cooking show would consume more food than the control condition. Besides this, the study also hypothesized that those who watched the food program would consume a greater amount of sweet foods.  Procedure: Participants were randomly assigned to one of two conditions: Planet Earth or the Food Network. After providing consent, participants completed a demographic questionnaire and levels of hunger and desirability were assessed. The research assistant confirmed that participants had not eaten in the last hour. Weight and height measurements were taken. They were told to spend the next 10-min watching either a clip from Planet Earth or the Food Network prior to being brought into the lab room for the taste-testing portion of the study. After watching the 10-minute video, the participant was taken to another room, where he was told to eat whatever he wants for the next 10 minutes. After the 10-min elapsed, the participant was asked to complete a post-taste-testing questionnaire and the TFEQ. The research assistant weighed each bowl of food to determine the amount of cheese curls, chocolate covered candies, and carrots each participant consumed.  Results: Although many studies have analyzed the effects of television habits on eating behavior, this study was the only one that has assessed the effect of a cooking program on eating behavior. Our results indicate that watching food programs increases consumption of sweet foods, which may be explained by the effects of priming. Perhaps the recent popularity and influx of cooking and food shows is a factor that contributes to the obesogenic environment and can affect weight gain and obesity. More research is needed to further elucidate the role that food programs have on the obesity pandemic. | | | | |
|  | Participant details (eg, age/sex and number) | Age | | Between the ages of 18–22 (M = 19.5 SD = 1.00). | | |
|  |  | Sex | | The majority of the sample were female (72.5%), 27.5% were male. | | |
|  |  | Final enrollment | | N=80 | | |
| Details/results extracted from source of evidence | Review question addressed  (Tick) | 1. | The health-related elements in *mukbang* (Food ads) | | √ | |
|  |  | Source text | Mukbang videos (food television programs) glamorize eating without discussing the health consequences associated with excess consumption of food. | | | |
|  |  | 2. | The relationship between *mukbang* (Food ads) and health | | √ | |
|  |  | Source text | 1) Increase intake of the viewers which can be both health and unhealth for the viewers: both children and adults who were exposed to food advertisements consumed more foods than those who were not exposed to these advertisements, the results of this study are consistent with those literature.  2) Watching food programs increases consumption of sweet foods, perhaps the recent popularity and influx of cooking and food shows is a factor that contributes to the obesogenic environment and can affect weight gain and obesity. | | | |
|  |  | 3. | Impacts may *mukbang* (Food ads) have on eating habits or eating behaviors | | √ | |
|  |  | Source text | 1) Watching this type of food-related program (in this study was cooking show) led to an increased consumption of sweet foods.  2) Food information in food-related videos can influence viewers' food choices: it is likely that the participants’ memory for the sweet food in the video served as a primer that led to more sweet food consumption during the experiment. | | | |
|  |  | 4. | The design, development, or usability of *mukbang* (Food ads) watching interventions to demonstrate the usefulness of *mukbang* in the health field | | | NA |
|  |  | Source text | NA | | | |
|  |  | 5. | Evaluation of the health effects of various types of *mukbang* (Food ads) | | | NA |
|  |  | Source text | NA | | | |
|  |  | 6. | The challenges and barriers of integrating *mukbang* (Food ads) videos into clinical practice | | | NA |
|  |  | Source text | NA | | | |
|  |  | 7. | The advantages and/or disadvantages of *mukbang* (Food ads) watching | | | √ |
|  |  | Source text | Disadvantages: Excessive exposure to food-related videos may lead to excessive intake of junk food, which can lead to overweight and obesity. | | | |
|  | Research method/Tools | 1) Demographic information questionnaire and BMI (using Seca scale to record height and weight). BMI was calculated using the standard formula.  2) A pre- and post- taste-testing questionnaire, which asked about their desirability to eat food and their level of hunger.  3) A Visual Analogue Scale (VAS): determine hunger and palatability of foods.  4) Using cheese curls, chocolate covered candies and carrots to measure the amount of food each participant consumed.  5) Using an EatSmart scale to do the taste-testing experiment.  6) Three Factor Eating Questionnaire (TFEQ-51): to measure three different aspects of eating behaviors: cognitive restraint, disinhibition, and hunger. | | | | |
|  | Characteristics of *mukbang* | The “mukbang videos” in this study was a 10-min cooking show with Rachel Ray from The Food Network. This clip showed a variety of different foods: Prosciutto Wrapped Cod, ‘‘Peasto’’ Pasta, asparagus drizzled with balsamic, and a fruit tart for dessert. | | | | |
|  | Health-related elements | Sweet food (unhealthy food) vs. fruit and vegetable (healthy food) | | | | |
|  | Summary of key findings related to health | 1) Findings suggest that watching food-related television programs may affect eating behavior and has implications for obesity prevention and intervention efforts.  2) This study has demonstrated an effect with such a brief exposure, which suggests that longer amounts of exposure may lead to greater amounts of calories consumed. This means the extent to which viewers' eating behavior is affected by food-related videos may be related to the length of time spent watching them.  3) Research gap: the viewer's own factors, such as weight, BMI, and gender, may also influence the impact of food-related videos on their intake. So further research can focus on overweight or obesity people.  4) Implications for weight loss programs would be to encourage obese patients who to minimize exposure to such television programs to avoid the overpowering biological drive to consume these foods. | | | | |

*Food ads must include images of celebrities eating.

29. ‘Food porn’ or intimate sociality committed celebrity and cultural performances of overeating in meokbang

|  | Items | Data | | | | |
| --- | --- | --- | --- | --- | --- | --- |
| Inclusion criteria | Population/Main research object | Talked about “food porn”, meokbang and the significance of eating for modern people.  Food porn, an inadequate metaphor to describe either the unattainable food-images produced or the modes of viewing these encourage.  Meokbang seemingly reduces ‘food porn’ to its embodied, fleshy, messy essential. It orgiastically embraces ‘ordinary’ foods, with unattainability instead represented by overabundance and excess. It engenders divergent audience affects and ambivalent modes of viewing, including pleasure, desire, longing, horror, disgust and shame. | | | | |
|  | Concept (Tick)  Studies focusing on | 1. | *mukbang* watching (Food ads*) | | √ | |
|  |  | 2. | the impact of *mukbang* (Food ads) on health | | √ | |
|  |  | 3. | the relationship between *mukbang* (Food ads) and health | | √ | |
|  |  | 4. | the design, development, or usability of *mukbang* (Food ads) watching interventions to demonstrate the usefulness of *mukbang* in the health field | | NA | |
|  |  | 5. | evaluating the effectiveness of various types of *mukbang* (Food ads) for public health | | NA | |
|  |  | 6. | the challenges and barriers of integrating *mukbang* (Food ads) videos into clinical practice | | NA | |
|  |  | 7. | the advantages and/or disadvantages of *mukbang* (Food ads) watching | | NA | |
|  | Types of evidence source | A review. | | | | |
| Evidence source details and characteristics | Citation details (eg, author/s, date, title, journal, volume, issue, pages) | Title | | ‘Food porn’ or intimate sociality: committed celebrity and cultural performances of overeating in *meokbang* | | |
|  |  | Journal | | Celebrity Studies | | |
|  |  | Author/s | | Glen Donnar | | |
|  |  | Date | | 2017-01-02 | | |
|  | Main content of the evidence | This study aimed to examine how these popular broadcasted performances of overeating and committed DIY (do-it-yourself) celebrity navigate cultural and economic tensions, anxieties and passions specific to Korean society and culture.  This research mainly analyzed the role of mukbang in the social environment and culture of Korea, explored the cultural connotation of mukbang, and the interaction and relationship between mukbang and society. At the same time, the health-related information contained in mukbang was also be explored. It reveals the reasons for mukbang's existence and popularity under the influence of society and culture, as well as its impact on Korean society and people. | | | | |
|  | Participant details (eg, age/sex and number) | Age | | NA | | |
|  |  | Sex | | NA | | |
|  |  | Final enrollment | | NA | | |
| Details/results extracted from source of evidence | Review question addressed  (Tick) | 1. | The health-related elements in *mukbang* (Food ads) | | NA | |
|  |  | Source text | NA | | | |
|  |  | 2. | The relationship between *mukbang* (Food ads) and health | | √ | |
|  |  | Source text | 1) Mukbang watching may cause growing obesity and food disorders through to an ever-greater proportion of Koreans living and eating alone.  2) In satisfying the desire for vicarious consumption, some dieting female fans not only enviously celebrate a performer’s capacity to eat to excess while remaining thin, but also watch to avoid actual eating.  3) Reduce body anxiety: on the contrary, mukbang watching can reduce women's strict requirements for their own figures to a certain extent, as well as reduce the restraint of diet, and reduce the self-denial of the figure. | | | |
|  |  | 3. | Impacts may *mukbang* (Food ads) have on eating habits or eating behaviors | | √ | |
|  |  | Source text | Meokbang may influence its viewers to maintain pathological eating and eating habits. | | | |
|  |  | 4. | The design, development, or usability of *mukbang* (Food ads) watching interventions to demonstrate the usefulness of *mukbang* in the health field | | | NA |
|  |  | Source text | NA | | | |
|  |  | 5. | Evaluation of the health effects of various types of *mukbang* (Food ads) | | | NA |
|  |  | Source text | NA | | | |
|  |  | 6. | The challenges and barriers of integrating *mukbang* (Food ads) videos into clinical practice | | | NA |
|  |  | Source text | NA | | | |
|  |  | 7. | The advantages and/or disadvantages of *mukbang* (Food ads) watching | | | NA |
|  |  | Source text | NA | | | |
|  | Research method/Tools | Describe and evaluate social phenomena. | | | | |
|  | Characteristics of *mukbang* | The study argues that mukbang presents ordinary food that is easily available to anyone, but that ordinary people usually can't eat as much food in one sitting as mukbang hosts.  Intimacy, immediacy and community are integral to meokbang. | | | | |
|  | Health-related elements | NA | | | | |
|  | Summary of key findings related to health | 1) South Korea's social orientation and culture has led many young women to use mukbang as a substitute diet to prevent themselves from eating too much food and gaining weight. However, under the influence of the extreme social atmosphere of thinness in South Korea, young women in South Korea are generally in a state of excessive weight loss. This, in turn, can be an unhealthy way in using mukbang.  2) Mukbang watching can make people ignore expectations of healthy everyday practices they equally refuse directives of self-governance. | | | | |

*Food ads must include images of celebrities eating.

30. The impacts and countermeasure of the novelty seeking live videos chaos on teenagers

|  | Items | Data | | | | |
| --- | --- | --- | --- | --- | --- | --- |
| Inclusion criteria | Population/Main research object | Novelty-seeking live streaming | | | | |
|  | Concept (Tick)  Studies focusing on | 1. | *mukbang* watching (Food ads*) | | √ | |
|  |  | 2. | the impact of *mukbang* (Food ads) on health | | √ | |
|  |  | 3. | the relationship between *mukbang* (Food ads) and health | | √ | |
|  |  | 4. | the design, development, or usability of *mukbang* (Food ads) watching interventions to demonstrate the usefulness of *mukbang* in the health field | | NA | |
|  |  | 5. | evaluating the effectiveness of various types of *mukbang* (Food ads) for public health | | NA | |
|  |  | 6. | the challenges and barriers of integrating *mukbang* (Food ads) videos into clinical practice | | NA | |
|  |  | 7. | the advantages and/or disadvantages of *mukbang* (Food ads) watching | | √ | |
|  | Types of evidence source | Commentary | | | | |
| Evidence source details and characteristics | Citation details (eg, author/s, date, title, journal, volume, issue, pages) | Title | | The impacts and countermeasure of the novelty seeking live videos chaos on teenagers | | |
|  |  | Journal | | Science & Technology Information | | |
|  |  | Author/s | | Zixuan Gao | | |
|  |  | Date | | 2021 | | |
|  | Main content of the evidence | This paper takes Novelty-seeking live streaming as the main research object, classifies existing network live streaming through its definition, and classifies Big Eater Mukbang and Novelty-seeking Mukbang into the scope. Firstly, the characteristics and prevalence of this kind of live broadcast are summarized.  The core of this paper is to analyze the causes of Novelty-seeking live streaming disorder, its impact on adolescents' health outlook, values, consumption outlook, career outlook, and future management methods. | | | | |
|  | Participant details (eg, age/sex and number) | Age | | NA | | |
|  |  | Sex | | NA | | |
|  |  | Final enrollment | | NA | | |
| Details/results extracted from source of evidence | Review question addressed  (Tick) | 1. | The health-related elements in *mukbang* (Food ads) | | √ | |
|  |  | Source text | The overeating, waste of food, abnormal psychology, reckless consumption and abnormal career outlook transmitted by the novelty-seeking live streaming have a negative impact on the formation of teenagers' values, resulting in the distortion of teenagers' values.  The mukbang video mentioned in this article: anchors eat dark cuisine, eat hot pot base directly, or drink oil and vinegar and other strange forms. | | | |
|  |  | 2. | The relationship between *mukbang* (Food ads) and health | | √ | |
|  |  | Source text | Negative impact on health: The strong contrast between mukbang anchors' special ability to "eat extremely well" and their beautiful body and appearance causes the envy of teenagers, and leads to their blind imitation, distorting their health and food outlook virtually, constantly encouraging teenagers to overeat indiscriminately, and seriously endangering their health. | | | |
|  |  | 3. | Impacts may *mukbang* (Food ads) have on eating habits or eating behaviors | | √ | |
|  |  | Source text |  | | | |
|  |  | 4. | The design, development, or usability of *mukbang* (Food ads) watching interventions to demonstrate the usefulness of *mukbang* in the health field | | | NA |
|  |  | Source text | NA | | | |
|  |  | 5. | Evaluation of the health effects of various types of *mukbang* (Food ads) | | | NA |
|  |  | Source text | NA | | | |
|  |  | 6. | The challenges and barriers of integrating *mukbang* (Food ads) videos into clinical practice | | | NA |
|  |  | Source text | NA | | | |
|  |  | 7. | The advantages and/or disadvantages of *mukbang* (Food ads) watching | | | √ |
|  |  | Source text | Disadvantages：In this study, Big Eater Mukbang belongs to Novelty-seeking videos. The author believes that the images of anchors eating a large amount of food at one time in Big Eater Mukbang will stimulate teenagers to overeat incontinently. And the contrast between the huge food intake and the slim figure of the anchor will also invisibly distort the health view of teenagers.  At the same time, the proliferation of "fake eating" or "vomiting" in the mukbang industry has led to more and more serious food waste. If the extravagant behavior of anchors is not restricted, teenagers' food outlook will also be affected and they will no longer save food. | | | |
|  | Research method/Tools | NA | | | | |
|  | Characteristics of *mukbang* | Novelty-seeking live streaming: It is a form of network broadcast that caters to people's curiosity about social scandals and new things, and satisfies their desire for curiosity and snooping. This article classifies the Big Eater Mukbang as novelty-seeking live streaming  Meanwhile, the mukbang video in this study only includes the form of live broadcast. | | | | |
|  | Health-related elements | Health concept; Health vs. distorted eating habits and behaviors | | | | |
|  | Summary of key findings related to health | By analyzing the characteristics and popular status of Big Eater Mukbang and Heavy Taste mukbang videos, this paper focused on the impact of this type of webcast on adolescents' health views and values. The study found that this type of mukbang mainly promoted binge eating, waste of food, abnormal psychology and wanton consumption, which had a negative impact on the correct formation of adolescents' health views and values, continuously stimulated adolescents to binge eating without restraint, seriously harmed the health of adolescents, and led to the distortion of adolescents' health views and values. | | | | |

*Food ads must include images of celebrities eating.

31. See the cake and have it too Investigating the effect of watching a TV cooking show on unhealthy food choices

|  | Items | Data | | | | |
| --- | --- | --- | --- | --- | --- | --- |
| Inclusion criteria | Population/Main research object | The participants’ unhealthy food choices were not related to age, gender, handedness, BMI, or hunger. | | | | |
|  | Concept (Tick)  Studies focusing on | 1. | *mukbang* watching (Food ads, food related videos*) | | √ | |
|  |  | 2. | the impact of *mukbang* (Food ads, food related videos) on health | | √ | |
|  |  | 3. | the relationship between *mukbang* (Food ads, food related videos) and health | | √ | |
|  |  | 4. | the design, development, or usability of *mukbang* (Food ads, food related videos) watching interventions to demonstrate the usefulness of *mukbang* in the health field | | √ | |
|  |  | 5. | evaluating the effectiveness of various types of *mukbang* (Food ads, food related videos) for public health | | NA | |
|  |  | 6. | the challenges and barriers of integrating *mukbang* (Food ads, food related videos) videos into clinical practice | | √ | |
|  |  | 7. | the advantages and/or disadvantages of *mukbang* (Food ads, food related videos) watching | | NA | |
|  | Types of evidence source | A single factor between-subjects experiment | | | | |
| Evidence source details and characteristics | Citation details (eg, author/s, date, title, journal, volume, issue, pages) | Title | | See the cake and have it too? Investigating the effect of watching a TV cooking show on unhealthy food choices | | |
|  |  | Journal | | Physiology & Behavior | | |
|  |  | Author/s | | Alblas, M. C. | | |
|  |  | Date | | 2021-07-01 | | |
|  | Main content of the evidence | This study tested whether exposure to food-related (vs. non-food related) TV content would increase unhealthy food choices in unsuccessful restrained eaters (i.e., chronic dieters with low perceived self-regulatory success; PSRS), decrease unhealthy food choices in successful restrained eaters (i.e., chronic dieters with high PSRS), and would not affect food choices in unrestrained eaters (i.e., non-dieters).  Based on the discussed theoretical and empirical research, it was expected that unsuccessful restrained eaters – due to their low self-regulatory success – would be particularly susceptible to the influences of watching food-related (vs. non-food related) TV content, and such exposure would for them result in more unhealthy food choices. In contrast, successful restrained eaters were expected to make less unhealthy food choices after watching food related TV content due to facilitative links between tempting food situations and automatic activation of their dieting goals. As unrestrained eaters have been found relatively insensitive to external food cues, their food choices were expected to be unaffected by exposure to food-related TV content. | | | | |
|  | Participant details (eg, age/sex and number) | Age | | Mage = 20.93, SDage = 2.16 | | |
|  |  | Sex | | 90 females, 22 males | | |
|  |  | Final enrollment | | 112 participants | | |
| Details/results extracted from source of evidence | Review question addressed  (Tick) | 1. | The health-related elements in *mukbang* (Food ads) | | √ | |
|  |  | Source text | Two high-calorie cakes | | | |
|  |  | 2. | The relationship between *mukbang* (Food ads, food related videos) and health | | √ | |
|  |  | Source text | 1) People who had watched the cooking segment tended to make a higher percentage of unhealthy food choices compared to people who had watched the non-food related segment.  2) Participants who perceived themselves lower in self-regulatory success made more unhealthy food choices compared to participants who perceived themselves to be higher in self-regulatory success.  3) Watching a cooking segment (vs. a non-food segment) of a TV show did not result in more unhealthy food choices in unsuccessful restrained eaters. | | | |
|  |  | 3. | Impacts may *mukbang* (Food ads, food related videos) have on eating habits or eating behaviors | | NA | |
|  |  | Source text | NA | | | |
|  |  | 4. | The design, development, or usability of *mukbang* (Food ads, food related videos) watching interventions to demonstrate the usefulness of *mukbang* in the health field | | | √ |
|  |  | Source text | Food-related videos (such as cooking videos) are a complex form of food presentation because they don't just include food, but also stars or a lot of other content that may distract the viewer from the food to some extent. Therefore, when applying food-related videos (such as eating broadcast) to the clinic to promote appetite or eating, attention should be paid to the specificity of the video in presenting food, and too much other content unrelated to food should be avoided. At the same time, attention should be paid to choosing healthy food. | | | |
|  |  | 5. | Evaluation of the health effects of various types of *mukbang* (Food ads, food related videos) | | | NA |
|  |  | Source text | NA | | | |
|  |  | 6. | The challenges and barriers of integrating *mukbang* (Food ads, food related videos) videos into clinical practice | | | √ |
|  |  | Source text | For unsuccessful restrained eaters only highly salient or attentively viewed food cues (like mukbang videos focus on food) may result in food intake. | | | |
|  |  | 7. | The advantages and/or disadvantages of *mukbang* (Food ads, food related videos) watching | | | NA |
|  |  | Source text | NA | | | |
|  | Research method/Tools | Questionnaires and Scales:  1) Eating restraint was assessed with the Concern for Dieting subscale of the Restraint Scale  2) To measure perceived dieting success, PSRS was assessed with three items.  Unhealthy food choices were assessed in a computerized choice task (based on Van der Laan, De Ridder, Charbonnier, Viergever, & Smeets, 2014) which was programmed using Inquisit 4. | | | | |
|  | Characteristics of *mukbang* | The preparation of food in these programs is often presented in an amusing and visually appealing way, and the dishes that are displayed are often high in calories, and added fat, sugar, and/ or salt, and low in nutritional value (hereafter referred to as unhealthy. | | | | |
|  | Health-related elements | Unhealthy food in the food related videos (cooking videos).  Most studies have concluded that foods shown in food-related videos are unhealthy, but no studies have explored what types of food are commonly shown in food-related videos in a given region (especially mukbang videos). | | | | |
|  | Summary of key findings related to health | 1) The percentage of unhealthy food choices was not related to age, gender, handedness, BMI, or hunger.  2) Differences between successful and unsuccessful restrained eaters are particularly found on trials in which the unhealthy food is perceived as tastier than the healthy food, because these trials require self-control (i.e., to choose the healthy, but less palatable food over the unhealthy, palatable food).  3) Both successful and unsuccessful restrained eaters try to restrict their food intake, unsuccessful restrained eaters often fail to succeed in their dieting attempts because of their heightened susceptibility to exposure to palatable food cues. | | | | |

*Food ads and food related videos must include images of celebrities eating.

32. Obesity and food-related content aimed at children on YouTube

|  | Items | Data | | | | |
| --- | --- | --- | --- | --- | --- | --- |
| Inclusion criteria | Population/Main research object | These 304 videos are feature or target children and include the presence of food products and/or food brands: videos on the official channels of the 13 Spanish food brands featuring or targeting children (n = 82 videos), and videos by the 15 Spanish child YouTubers that feature food products and/or food brands (n = 222 videos).  The video footage that met all of the following selection criteria: presence (verbal or visual) of food products or brands, and the presence of child protagonists/ actors/influencers.  In child YouTuber videos, the challenges in which food products generally appear (with or without reference to a specific brand) are varied: ‘pause challenge’; ‘24-hour challenge’; ‘other YouTubers choose my food’; ‘24 hours eating food of one color’; ‘truth versus lie with food’; ‘if you spell it, I'll buy it for you’; ‘I throw a dart and they buy me whatever I hit’; ‘morning, afternoon or night-time routine’; and ‘ordering the same thing as the last customer’. | | | | |
|  | Concept (Tick)  Studies focusing on | 1. | *mukbang* watching (Food ads*)  (The videos in this study were presented in the form of mukbang) | | √ | |
|  |  | 2. | the impact of *mukbang* (Food ads) on health | | √ | |
|  |  | 3. | the relationship between *mukbang* (Food ads) and health | | √ | |
|  |  | 4. | the design, development, or usability of *mukbang* (Food ads) watching interventions to demonstrate the usefulness of *mukbang* in the health field | | NA | |
|  |  | 5. | evaluating the effectiveness of various types of *mukbang* (Food ads) for public health | | √ | |
|  |  | 6. | the challenges and barriers of integrating *mukbang* (Food ads) videos into clinical practice | | NA | |
|  |  | 7. | the advantages and/or disadvantages of *mukbang* (Food ads) watching | | NA | |
|  | Types of evidence source | An exploratory qualitative-quantitative study | | | | |
| Evidence source details and characteristics | Citation details (eg, author/s, date, title, journal, volume, issue, pages) | Title | | Obesity and food-related content aimed at children on YouTube | | |
|  |  | Journal | | Clinical Obesity | | |
|  |  | Author/s | | Araceli Castelló-Martínez, Victoria Tur-Viñes | | |
|  |  | Date | | 2020 | | |
|  | Main content of the evidence | The aim of this study is to identify elements or arguments in YouTube food-content aimed at children, to confirm the presence of negative communication features above the presence of positive trends on YouTube contents aimed at children.  The research questions were: ①Do ultra-processed foods predominate over healthy foods in media content? ②Which food brands appear most often? ③How are children presented? And ④What characterizes the communicative presence of products and brands? | | | | |
|  | Participant details (eg, age/sex and number) | Age | | NA | | |
|  |  | Sex | | NA | | |
|  |  | Final enrollment | | The final sample consisted of 304 videos published by both types of channels in YouTube in 2019. | | |
| Details/results extracted from source of evidence | Review question addressed  (Tick) | 1. | The health-related elements in *mukbang* (Food ads) | | √ | |
|  |  | Source text | Chocolate and chocolate derivatives, fast-food establishments, and confections and candies were the food products which often presented in the TV programs or YouTube videos. Thus, food products presented in the videos towards children were with high-calorie.  These were the unhealthy things in the food related videos. | | | |
|  |  | 2. | The relationship between *mukbang* (Food ads) and health | | √ | |
|  |  | Source text | 1) A negative impact on child obesity: the strategies used to advertise products with low nutritional value have a negative impact on child obesity prevention policies, as they encourage children to consume such products, affecting their calorie intake, and to associate them with positive emotions and experiences as a purchasing benefit.  2) Healthy eating habits are also promoted by the food ads made by brands but receive minimal attention from the child YouTubers. | | | |
|  |  | 3. | Impacts may *mukbang* (Food ads) have on eating habits or eating behaviors | | NA | |
|  |  | Source text | NA | | | |
|  |  | 4. | The design, development, or usability of *mukbang* (Food ads) watching interventions to demonstrate the usefulness of *mukbang* in the health field | | | NA |
|  |  | Source text | NA | | | |
|  |  | 5. | Evaluation of the health effects of various types of *mukbang* (Food ads) | | | √ |
|  |  | Source text | The brands are clearly more committed to promoting healthy lifestyles (walking for 30 minutes a day; eating breakfast every day; using the stairs; exercising and maintaining a healthy diet). However, in the videos by child YouTubers, there are hardly any proactive messages, which constitutes a regrettable waste of a format with a bigger influence on and greater credibility for children. | | | |
|  |  | 6. | The challenges and barriers of integrating *mukbang* (Food ads) videos into clinical practice | | | NA |
|  |  | Source text | NA | | | |
|  |  | 7. | The advantages and/or disadvantages of *mukbang* (Food ads) watching | | | NA |
|  |  | Source text | NA | | | |
|  | Research method/Tools | A content analysis method. | | | | |
|  | Characteristics of *mukbang* | This research was not about mukbang that we always say, but about TV food ads and YouTube videos refer to children and food products or brands.  The videos mentioned in this study contained images of children eating, or they were be shown and eaten through challenges and stories. Therefore, the videos involved in this paper were in line with the characteristics of mukbang and can be regarded as mukbang. | | | | |
|  | Health-related elements | NA | | | | |
|  | Summary of key findings related to health | 1) The presentation of child YouTuber videos was more similar to mukbang videos, which contain more negative content which may has a worse impact on children’s health and eating behaviors: the negative features are more prevalent in child YouTuber videos, while having only an incidental presence in the brand videos. Healthy eating habits are also promoted by the brands but receive minimal attention from the child YouTubers.  2) This study reveals some troubling media practices associated with obesogenic issues. A responsible commitment is required from these spaces, with the decisive promotion of healthy eating habits to combat the pandemic of obesity, and its associated pathologies, in a population as prone to high risk as children.  3) Existing research has focused on the effects of adult-oriented mukbang videos (or food-related videos) on the diet and health of adult viewers, but few scholars have focused on children. | | | | |

*Food ads must include images of celebrities eating.

33. Flavor-Videos Enhancing the Flavor Perception of Food while Eating with Videos

|  | Items | Data | | | | |
| --- | --- | --- | --- | --- | --- | --- |
| Inclusion criteria | Population/Main research object |  | | | | |
|  | Concept (Tick)  Studies focusing on | 1. | *mukbang* watching (Food ads*) | | √ | |
|  |  | 2. | the impact of *mukbang* (Food ads) on health | | √ | |
|  |  | 3. | the relationship between *mukbang* (Food ads) and health | | √ | |
|  |  | 4. | the design, development, or usability of *mukbang* (Food ads) watching interventions to demonstrate the usefulness of *mukbang* in the health field | | √ | |
|  |  | 5. | evaluating the effectiveness of various types of *mukbang* (Food ads) for public health | | NA | |
|  |  | 6. | the challenges and barriers of integrating *mukbang* (Food ads) videos into clinical practice | | NA | |
|  |  | 7. | the advantages and/or disadvantages of *mukbang* (Food ads) watching | | √ | |
|  | Types of evidence source | Experimental study | | | | |
| Evidence source details and characteristics | Citation details (eg, author/s, date, title, journal, volume, issue, pages) | Title | | Flavor-Videos: Enhancing the Flavor Perception of Food while Eating with Videos | | |
|  |  | Journal | | ACM International Conference on Interactive Media Experiences | | |
|  |  | Author/s | | Meetha Nesam James, Nimesha Ranasinghe, Anthony Tang, Lora Oehlberg | | |
|  |  | Date | | 2022-11-22 | | |
|  | Main content of the evidence | This study presented a user study to investigate the influence of six types of videos, including mukbang - a new food video genre, on flavor perceptions (taste sensations, liking, and emotions) while eating plain white rice.  The findings revealed that participants perceived positive emotional changes and reported significant differences in their augmented taste sensations (e.g., spicy and salty) with different food-based videos. These findings provided insights into using food-related videos (like mukbang videos) to promote healthier eating (digital augmentation without altering the food). This study has hypothesized that videos watched while eating using digital technology could positively enhance an enjoyable eating experience in terms of taste sensations, liking, and emotions.  Our study explored the following research question - “Do food-based videos watched while eating augment viewers’ flavor perceptions in terms of taste sensations, liking, and emotions?”. The two main findings of our study are: 1) food-based videos augment flavors when watched while eating, especially in terms of taste sensations, and 2) food-based videos enhance the eating experience by producing positive emotional changes. | | | | |
|  | Participant details (eg, age/sex and number) | Age | | 18 - 50 years of age (M =26.1, S.D = 6.9). | | |
|  |  | Sex | | 46% were female, 51% were male, and 3% gender fluid. | | |
|  |  | Final enrollment | | 35 participants from different parts of the United States via social media. | | |
| Details/results extracted from source of evidence | Review question addressed  (Tick) | 1. | The health-related elements in *mukbang* (Food ads) | |  | |
|  |  | Source text |  | | | |
|  |  | 2. | The relationship between *mukbang* (Food ads) and health | | √ | |
|  |  | Source text | 1) This approach can be applied to deliver a pleasurable food-eating experience without restrictions (while still keeping it healthy).  2) This finding also displayed the potential for digitally enhancing taste sensation with videos, which can be beneficial for people to satisfy their cravings virtually by watching videos (without indulging in overeating or unhealthy eating habits).  3) Positive and cheerful mood: in the category of food-based videos, the conversational mukbang video was linked to a wide number of emotions such as ‘happy’, ‘energetic’, ’friendly’, and ‘joyful’. There were positive emotional changes while watching food-based videos, especially mukbang videos, which promised an enjoyable eating experience. | | | |
|  |  | 3. | Impacts may *mukbang* (Food ads) have on eating habits or eating behaviors | | NA | |
|  |  | Source text | NA | | | |
|  |  | 4. | The design, development, or usability of *mukbang* (Food ads) watching interventions to demonstrate the usefulness of *mukbang* in the health field | | | √ |
|  |  | Source text | 1) The future implications of this research work extend into a wide range of fields, including food and beverage customization for people on restricted diets due to their medical conditions (e.g., diabetes, high blood pressure, other chronic illnesses).  2) People on restricted diets (e.g., people with diabetes who cannot have high levels of sugar, people with high blood pressure who should limit their salt intake) may benefit by choosing appropriate mukbang content to watch during their mealtime to enhance their salty or sweet sensations without actually adding the physical ingredient to the food. | | | |
|  |  | 5. | Evaluation of the health effects of various types of *mukbang* (Food ads) | | | NA |
|  |  | Source text | NA | | | |
|  |  | 6. | The challenges and barriers of integrating *mukbang* (Food ads) videos into clinical practice | | | NA |
|  |  | Source text | NA | | | |
|  |  | 7. | The advantages and/or disadvantages of *mukbang* (Food ads) watching | | | √ |
|  |  | Source text | Advantages: 1) People like astronauts, or people with dietary restrictions, who have restricted flavor access, can have enhanced flavor perception using our visual content based digital flavor augmentation approach.  Disadvantages: The anchor's eating style was unsightly and he ate too much food at once. | | | |
|  | Research method/Tools | Video-based intervention  Using different questionnaires to measure. | | | | |
|  | Characteristics of *mukbang* | The mukbangers: 1) actively describe the flavor associations of food-related to smell, taste, texture while eating, 2) eat the food without talking, 3) have a story-line (such as a podcast), or their life events shared while eating, 4) eat with other people in the broadcast, and 5) give live feedback to the comments, and some of them even get rewards (e.g., star balloon emojis) from their audience for an enjoyable experience. | | | | |
|  | Health-related elements | NA | | | | |
|  | Summary of key findings related to health | 1) The data was proposed to be helpful to understand which video content can be played to enhance a particular taste sensation for people. The finding also promotes digital augmentation of the taste and thereby avoids the need to physically or chemically alter the food, which makes it a healthier alternative.  2) It is evident from the findings that, with the videos watched while eating, especially food-based videos, positive flavor augmentation was possible.  3) This research has showed that watching food-based videos, especially mukbang videos, fosters digital commensality and delivers an enjoyable dining experience with enhanced flavor sensations. | | | | |

*Food ads must include images of celebrities eating.

34. DOES WATCHING MUKBANGS HELP YOU DIET THE EFFECT OF THE MUKBANG ON THE DESIRE TO EAT

|  | Items | Data | | | | |
| --- | --- | --- | --- | --- | --- | --- |
| Inclusion criteria | Population/Main research object | In the first two studies (one in-person and one online), female participants were recruited and randomized to watch either a non-food content video or a mukbang. The third study added a third option: participants could also be randomized to watch a cooking show.  In the US, 24.8% participants reported that they have viewed mukbangs to diet and 56% of them agree it is a useful diet strategy. In the China study, 19.2% reported that they tried it before and 46.7% of these motivated dieters found it effective. | | | | |
|  | Concept (Tick)  Studies focusing on | 1. | *mukbang* watching (Food ads, food related videos*) | | √ | |
|  |  | 2. | the impact of *mukbang* (Food ads, food related videos) on health | | √ | |
|  |  | 3. | the relationship between *mukbang* (Food ads, food related videos) and health | | √ | |
|  |  | 4. | the design, development, or usability of *mukbang* (Food ads, food related videos) watching interventions to demonstrate the usefulness of *mukbang* in the health field | | NA | |
|  |  | 5. | evaluating the effectiveness of various types of *mukbang* (Food ads, food related videos) for public health | | √ | |
|  |  | 6. | the challenges and barriers of integrating *mukbang* (Food ads, food related videos) videos into clinical practice | | √ | |
|  |  | 7. | the advantages and/or disadvantages of *mukbang* (Food ads, food related videos) watching | | NA | |
|  | Types of evidence source | A Thesis includes two in-person and one online randomized treatment and control trials. | | | | |
| Evidence source details and characteristics | Citation details (eg, author/s, date, title, journal, volume, issue, pages) | Title | | DOES WATCHING MUKBANGS HELP YOU DIET? THE EFFECT OF THE MUKBANG ON THE DESIRE TO EAT | | |
|  |  | Journal | | NA | | |
|  |  | Author/s | | Wenzhuo Xu | | |
|  |  | Date | | 2019-12 | | |
|  | Main content of the evidence | In this paper, the following questions are addressed:  1) what is the effect of mukbangs on viewer’s desire to eat, especially for dieters;  2) does watching mukbangs help control calorie intake among those who believe that watching mukbang is an effective diet aid;  3) is the effect of mukbangs specific to viewing people eating food, or do cooking videos without eating have a similar effect.  First, the paper examines the effect of mukbangs on dieters and investigates the difference between mukbangs and cooking shows. Second, this paper sheds insight on the behavioral phenomenon in which people intentionally view food cues to control their appetites. | | | | |
|  | Participant details (eg, age/sex and number) | Age | | NA | | |
|  |  | Sex | | Only women were recruited in all studies | | |
|  |  | Final enrollment | | Mukbang versus Non-Food Content Video (US): 114 women;  Effect of Different Food Stimuli (US): an online survey and recruited 286 workers from Amazon Mechanical Turk;  Mukbang versus Cooking Show (China): 234 women. | | |
| Details/results extracted from source of evidence | Review question addressed  (Tick) | 1. | The health-related elements in *mukbang* (Food ads, food related videos) | | NA | |
|  |  | Source text | NA | | | |
|  |  | 2. | The relationship between *mukbang* (Food ads, food related videos) and health | | √ | |
|  |  | Source text | 1) Considering the possibility that only by presenting unhealthy food can mukbang play a role in stimulating the viewer's appetite: the online study further compared the effects of different food stimuli in mukbangs and showed that people only increased their desire to eat the foods viewed in the mukbang in addition to fruit.  2) Some people may have the behavioral phenomenon in intentionally view food cues to control their appetites.  3) Some people regard watching mukbang as a substitute for actual high calorie consumption, and they watch the mukbang with the hope to control their appetites.  4) Mukbang may make binge eating videos could mislead the public and encourage people to eat more. Mukbangs provide entertainment, but also make eating high calorie food more socially acceptable.  5) Relieve the social pressure brought about by the strict beauty of the body: the social trend of pursuing a slim figure has led to an increased number of people under the pressure of diet control. The mukbang, contrary to this social pressure, emphasizes the enjoyment of eating food without worrying about weight control.  6) Mukbangs could make people increase their desire to eat and give audiences a misconception that eating large amounts of food is not harmful.  7) It is possible for dieters to obtain the expected feeling of disgust through mukbang viewing.  8)Mukbang can encourage dieters with strong self-control to consume more healthy foods, such as fruit: successful mukbang-diet strategy users even had 0.668 unit more desire to eat fruit.  8) People who successfully use mukbang to lose weight are able to gain a higher level of self-control through the process.  9) The mukbang had a larger effect on the dieters and motivated such participants who believe watching mukbang can help increase satiety and reduce food intake and have a strong self-control to eat more fruits.  10) Junk food (they use pizza) is likely to make people feel disgust and control their appetites. | | | |
|  |  | 3. | Impacts may *mukbang* (Food ads, food related videos) have on eating habits or eating behaviors | | √ | |
|  |  | Source text | 1) The Desire to Eat Food: mukbang could increase the desire eat dim sum as well as fruits or vegetables and that dieters had less desire to eat.  2) Watching mukbang increased the desire to eat the food shown in the video as well as fruits, no matter the food is healthy or not. But there’s no evidence shows the food in the videos also trigger the viewer's desire for other food (not the food in the video).  3) Experienced mukbang viewers, no matter whether their mukbang-diet strategy worked or not, were more likely to agree that the mukbang increases their satiation level, which helps them diet. (Or helps them to eat healthier).  4) Both mukbang and cooking show can increase the desire to eat the food showed in the videos.  5) Since mukbang hosts usually eat high-calorie and unhealthy foods, mukbang audiences might be misled into underestimating the consequences of such indulgence. | | | |
|  |  | 4. | The design, development, or usability of *mukbang* (Food ads, food related videos) watching interventions to demonstrate the usefulness of *mukbang* in the health field | | | NA |
|  |  | Source text | NA | | | |
|  |  | 5. | Evaluation of the health effects of various types of *mukbang* (Food ads, food related videos) | | | √ |
|  |  | Source text | The mukbang had significantly stronger effects on satiation than the cooking show but weaker effect on the change of desire to eat. | | | |
|  |  | 6. | The challenges and barriers of integrating *mukbang* (Food ads, food related videos) videos into clinical practice | | | √ |
|  |  | Source text | More research is needed to test whether some particular types of mukbangs might be useful for dieting and whether mukbangs with fast food or sweet foods is the most harmful given these likely increase unhealthy food intake. | | | |
|  |  | 7. | The advantages and/or disadvantages of *mukbang* (Food ads, food related videos) watching | | | NA |
|  |  | Source text | NA | | | |
|  | Research method/Tools | This study conducted two experiments in the U.S. and one experiment in China. Participants were randomized to watch either a mukbang or a non-food content video in the two U.S. studies. The China study added one cooking show as the third option. | | | | |
|  | Characteristics of *mukbang* | Mukbang emphasizes the enjoyment of eating and the interaction with its audience.  Hotpot mukbang: generally consists of her introducing the food and her enjoyment while eating well.  A ramen mukbang or a donut mukbang. | | | | |
|  | Health-related elements | The variables explored in this study: satiety, disgust and eating intentions. | | | | |
|  | Summary of key findings related to health | 1) Mukbangs made people feel less satiation compared to cooking shows.  2) Dieters had less interest in eating after watching videos compared to non-dieters.  3) Overall, all studies have found that mukbangs caused dieting women to have a stronger desire to consume food.  4) Increase food intake: although dieters were less motivated to eat the food appearing in the mukbang, overall, the effect of mukbangs on dieters was to increase their food intake.  5) Both mukbang and the cooking show can increased people’s desire to eat and decreased their satiation level.  6) Compared with cooking shows, mukbang can reduce viewers' sense of revenge and promote appetite.  7) When comparing the mukbang with a general cooking show, this study has found that the intention to eat triggered by mukbangs is stronger than that of a cooking show.  8) To sum up, motivated dieters watch mukbangs with the expectation to change their level of disgust or satiation, while only people with strong selfcontrol could successfully achieve this goal.  9) The experience of using mukbangs to diet, regardless of perceptions of effectiveness, was associated with higher satiation level and higher disgust level.  10) The desire to eat could depend on both the attractiveness of the food presented in video and the interactive and enjoyment features.  11) Our randomized controlled studies prove the casual effect of watching mukbangs on the desire to eat, which could lead to more calorie intake and thereby, obesity. Moreover, since the mukbang does not decrease the desire to eat for dieters believing in the mukbang-diet strategy, viewing mukbang could generally encourage dieters with the expectation to diet to eat more food. | | | | |

*Food ads and food related videos must include images of celebrities eating.

35. The Psychology of Mukbang Watching A Scoping Review of the Academic and Non-academic Literature

|  | Items | Data | | | | |
| --- | --- | --- | --- | --- | --- | --- |
| Inclusion criteria | Population/Main research object | All scientific literature including published papers, conference presentations, commentaries, content analyses, critical reviews, literature reviews, case reports, dissertations, and empirical studies that have examined mukbang phenomenon was searched for. | | | | |
|  | Concept (Tick)  Studies focusing on | 1. | *mukbang* watching (Food ads, food related videos*) | | √ | |
|  |  | 2. | the impact of *mukbang* (Food ads, food related videos) on health | | √ | |
|  |  | 3. | the relationship between *mukbang* (Food ads, food related videos) and health | | √ | |
|  |  | 4. | the design, development, or usability of *mukbang* (Food ads, food related videos) watching interventions to demonstrate the usefulness of *mukbang* in the health field | | NA | |
|  |  | 5. | evaluating the effectiveness of various types of *mukbang* (Food ads, food related videos) for public health | | NA | |
|  |  | 6. | the challenges and barriers of integrating *mukbang* (Food ads, food related videos) videos into clinical practice | | NA | |
|  |  | 7. | the advantages and/or disadvantages of *mukbang* (Food ads, food related videos) watching | | √ | |
|  | Types of evidence source | A scoping review | | | | |
| Evidence source details and characteristics | Citation details (eg, author/s, date, title, journal, volume, issue, pages) | Title | | The Psychology of Mukbang Watching: A Scoping Review of the Academic and Non-academic Literature | | |
|  |  | Journal | | International Journal of Mental Health and Addiction | | |
|  |  | Author/s | | Kagan Kircaburun1 & Andrew Harris1 & Filipa Calado1 & Mark D. Griffiths | | |
|  |  | Date | | 08/2021 | | |
|  | Main content of the evidence | The present study aimed to scope the literature in order to identify existing publications that have empirically investigated and/or theoretically examined the mukbang phenomenon and conceptualized the psychological characteristics of mukbang viewers and possible consequences of mukbang watching.  Results from the scoping review indicated that viewers use mukbang watching for social reasons, sexual reasons, entertainment, eating reasons, and/or as an escapist compensatory strategy. Furthermore, mukbang watching appears to have both beneficial consequences (e.g., diminishing feelings of loneliness and social isolation, constructing a virtual social community,) and non-beneficial consequences (e.g., altering food preferences, eating habits, and table manners, promoting disordered eating, potential excess, and ‘addiction’). | | | | |
|  | Participant details (eg, age/sex and number) | Age | | NA | | |
|  |  | Sex | | NA | | |
|  |  | Final enrollment | | 1053 publications were identified, but only 13 records remained, two of the outputs were theoretical and 11 of them were empirical publications providing both theoretical discussions and empirical findings concerning the mukbang phenomenon. A further 20 non-academic articles in British newspapers were also identified. | | |
| Details/results extracted from source of evidence | Review question addressed  (Tick) | 1. | The health-related elements in *mukbang* (Food ads, food related videos) | | √ | |
|  |  | Source text | Mukbang often includes instant meals, frozen food, and poor nourishing foods that were spicy and oily with a high caloric content. | | | |
|  |  | 2. | The relationship between *mukbang* (Food ads, food related videos) and health | | √ | |
|  |  | Source text | 1) Promote positive emotions in the audience: positive remarks about the mukbanger and food advanced higher feelings of community and positive emotions among those who watched mukbang. Viewers obtained pleasure from different sensations including listening to eating and cooking sounds such as chewing noises, preparing foods, and sounds from opening up food packages.  2) Mukbang can help alleviate stress. Mukbang videos apparently helped viewers relieve stress and to have pleasure and happiness.  3) Viewers’ vicarious pleasure demanded a large quantity of unhealthy food to be consumed.  4) Individuals were susceptible to consuming more than they normally would if they see another individual consuming a large high-calorie meal because of social comparison or mimicry. Watching mukbang videos where mukbangers eat very large portions of food might easily leads mukbang viewers to higher than normal consumption.  5) Elevated consumption promoted by mukbang could further contribute to the problems that South Korean society was already going through including growing obesity, food disorders, and real-life social isolation.  6) Mukbang helped those who were on a diet to have vicarious satisfaction of eating and those who had eating disorders.  7) Some young teenagers challenging themselves to eat as much as the mukbang broadcaster, some parents who have young children have defended government’s plan because they believed mukbang could negatively influence teenagers.  8) Mukbang glamorizes binge eating and makes a large audience think that binge eating is a popular behavior worth promoting.  9) Mukbang watching could even turn into an addictive behaviour for lonely individuals because they could communicate with thousands of people at home via mukbang. | | | |
|  |  | 3. | Impacts may *mukbang* (Food ads, food related videos) have on eating habits or eating behaviors | | √ | |
|  |  | Source text | 1) Slurping while eating is usually a sign of bad eating habits: making loud sounds while eating and showing the food in an appetizing way on camera attracted the viewers, especially the ones who were on a diet.  2) Mukbang videos affected viewers’ table manners because mukbangers usually exhibit bad eating and table manners by snatching or scooping food, and eating it up carelessly while conversing with their viewers with their mouths full.  3) They also emphasized their eating sounds in order to stimulate viewers’ senses in which all these behaviours contributed to disruption of traditional eating manners and habits that viewers had.  4) Mukbang could lead to decrease in homemade food production and an increase in fast food consumption.  5) Watching mukbang might be dangerous especially for younger viewers through modelling bad behaviour (e.g., binge eating) and perceiving it socially acceptable. | | | |
|  |  | 4. | The design, development, or usability of *mukbang* (Food ads, food related videos) watching interventions to demonstrate the usefulness of *mukbang* in the health field | | | NA |
|  |  | Source text | NA | | | |
|  |  | 5. | Evaluation of the health effects of various types of *mukbang* (Food ads, food related videos) | | | NA |
|  |  | Source text | NA | | | |
|  |  | 6. | The challenges and barriers of integrating *mukbang* (Food ads, food related videos) videos into clinical practice | | | NA |
|  |  | Source text | NA | | | |
|  |  | 7. | The advantages and/or disadvantages of *mukbang* (Food ads, food related videos) watching | | | √ |
|  |  | Source text | Advantages: 1) Mukbang videos had the potential to counteract loneliness and isolation by connecting and sharing a similar interest with a virtual community.  2) Watching mukbang can facilitate subjective closeness and a sense of community and help overcome loneliness and alienation for those who live alone and seek companionship and a dinner partner. | | | |
|  | Research method/Tools | Several electronic databases were used for this review including, but not limited to, Academic Search Elite, PsychArticles, PsychInfo, Science Direct,andScopus via using the research team’s Library One Search electronic search engine. Furthermore, Google Scholar was used as a secondary source search engine.  A snowballing method was also used to identify relevant publications from scientific literature via examining the reference lists of found studies.  In the second step, non-academic ‘grey’ literature was reviewed via examining the national newspapers in the UK as information sources. | | | | |
|  | Characteristics of *mukbang* | ‘Mukbang’ (i.e. a portmanteau of the South Korean words for ‘eating’ [‘meokneun’] and ‘broadcast’ [‘bangsong’] that refers to online broadcasts where individuals eat food and interact with the viewers). | | | | |
|  | Health-related elements | Addiction, binge eating, unhealthy eating behaviours, alleviate loneliness, vicarious satiation and vicarious consumption. | | | | |
|  | Summary of key findings related to health | 1) The sounds produced during mukbang may provide an autonomous sensory meridian response experience for some of the viewers that may lead to happiness and relief and have entertainment value.  2) The present review is a first attempt to scope the literature from the lens of psychology and associated disciplines and present information on what has been theorized and discussed concerning the psychological characteristics of mukbang viewers and possible consequences of mukbang watching. One of the most important aspects of mukbang viewing was that individuals appeared to use mukbang to compensate for their unattained real-life social needs.  3) Mukbang watching might have negative consequences for the viewers including (i) increased consumption of food because of social comparison or mimicry; (ii) alteration of viewers’ perception of food consumption and thinness, eating, health, table manners, and eating manners because of modelling of bad behaviours; and (iii) obesity and different eating disorders because of glorifying binge eating. | | | | |

*Food ads and food related videos must include images of celebrities eating.

36. Research on the Relieving Effect of ASMR Chewing Sounds on Anxiety in Food Video

|  | Items | Data | | | | |
| --- | --- | --- | --- | --- | --- | --- |
| Inclusion criteria | Population/Main research object | The main research object is ASMR mukbang. | | | | |
|  | Concept (Tick)  Studies focusing on | 1. | *mukbang* watching (Food ads, food related videos*) | | √ | |
|  |  | 2. | the impact of *mukbang* (Food ads, food related videos) on health | | √ | |
|  |  | 3. | the relationship between *mukbang* (Food ads, food related videos) and health | | √ | |
|  |  | 4. | the design, development, or usability of *mukbang* (Food ads, food related videos) watching interventions to demonstrate the usefulness of *mukbang* in the health field | | NA | |
|  |  | 5. | evaluating the effectiveness of various types of *mukbang* (Food ads, food related videos) for public health | | √ | |
|  |  | 6. | the challenges and barriers of integrating *mukbang* (Food ads, food related videos) videos into clinical practice | | √ | |
|  |  | 7. | the advantages and/or disadvantages of *mukbang* (Food ads, food related videos) watching | | √ | |
|  | Types of evidence source | A review | | | | |
| Evidence source details and characteristics | Citation details (eg, author/s, date, title, journal, volume, issue, pages) | Title | | Research on the Relieving Effect of ASMR Chewing Sounds on Anxiety in Food Video | | |
|  |  | Journal | | International Journal of Education and Humanities | | |
|  |  | Author/s | | Jinge Zhou | | |
|  |  | Date | | 2023 | | |
|  | Main content of the evidence | This article expounds the development characteristics of short food videos in the new media environment, and studies the relieving effect of ASMR chewing sound on anxiety in food videos. In the main text, the authors introduce the definition of mukbang and the definition and characteristics of ASMR mukbang, and explain in detail how the general ASMR is triggered. | | | | |
|  | Participant details (eg, age/sex and number) | Age | | NA | | |
|  |  | Sex | | NA | | |
|  |  | Final enrollment | | NA | | |
| Details/results extracted from source of evidence | Review question addressed  (Tick) | 1. | The health-related elements in *mukbang* (Food ads, food related videos) | | NA | |
|  |  | Source text | NA | | | |
|  |  | 2. | The relationship between *mukbang* (Food ads, food related videos) and health | | √ | |
|  |  | Source text | Mukbang has a positive impact on mental health ASMR mukbang can alleviate the anxiety and loneliness of young people to, and satisfy their sense of belonging. | | | |
|  |  | 3. | Impacts may *mukbang* (Food ads, food related videos) have on eating habits or eating behaviors | | NA | |
|  |  | Source text | NA | | | |
|  |  | 4. | The design, development, or usability of *mukbang* (Food ads, food related videos) watching interventions to demonstrate the usefulness of *mukbang* in the health field | | | NA |
|  |  | Source text | NA | | | |
|  |  | 5. | Evaluation of the health effects of various types of *mukbang* (Food ads, food related videos) | | | √ |
|  |  | Source text | This article was mainly about ASMR mukbang. ASMR mukbang mainly makes people's scalp, back and other parts feel numb through the visual presentation of pictures and the auditory presentation of sounds. | | | |
|  |  | 6. | The challenges and barriers of integrating *mukbang* (Food ads, food related videos) videos into clinical practice | | | √ |
|  |  | Source text | Video content lacks innovation and practicality, and video release lacks supervision. | | | |
|  |  | 7. | The advantages and/or disadvantages of *mukbang* (Food ads, food related videos) watching | | | √ |
|  |  | Source text | Advantages: 1. ASMR mukbang helps some people to sleep.  2. ASMR mukbang helps some people to relieve people's stress and anxiety in real life. | | | |
|  | Research method/Tools | review | | | | |
|  | Characteristics of *mukbang* | ASMR mukbang mainly makes people's scalp, back and other parts feel numb through the visual presentation of pictures and the auditory presentation of sounds. | | | | |
|  | Health-related elements | Anxiety, sleep quality | | | | |
|  | Summary of key findings related to health | 1. Immersed in the mukbang atmosphere created by food and sound communication, people can temporarily get rid of real life and get emotional satisfaction. 2. ASMR mukbang can alleviate the anxiety and loneliness of young people to some extent and satisfy their sense of belonging through the visual presentation of food and the 3D transmission of sound. | | | | |

37. Watch Your Flavors Augmenting People’s Flavor Perceptions and Associated Emotions Based on Videos Watched while Eating

|  | Items | Data | | | | |
| --- | --- | --- | --- | --- | --- | --- |
| Inclusion criteria | Population/Main research object | Thirty-five participants aged between 18 and 50 years were participant in this study. | | | | |
|  | Concept (Tick)  Studies focusing on | 1. | *mukbang* watching (Food ads, food related videos*) | | √ | |
|  |  | 2. | the impact of *mukbang* (Food ads, food related videos) on health | | √ | |
|  |  | 3. | the relationship between *mukbang* (Food ads, food related videos) and health | | √ | |
|  |  | 4. | the design, development, or usability of *mukbang* (Food ads, food related videos) watching interventions to demonstrate the usefulness of *mukbang* in the health field | | NA | |
|  |  | 5. | evaluating the effectiveness of various types of *mukbang* (Food ads, food related videos) for public health | | √ | |
|  |  | 6. | the challenges and barriers of integrating *mukbang* (Food ads, food related videos) videos into clinical practice | | NA | |
|  |  | 7. | the advantages and/or disadvantages of *mukbang* (Food ads, food related videos) watching | | NA | |
|  | Types of evidence source | Experimental study | | | | |
| Evidence source details and characteristics | Citation details (eg, author/s, date, title, journal, volume, issue, pages) | Title | | Watch Your Flavors: Augmenting People’s Flavor Perceptions and Associated Emotions based on Videos Watched while Eating | | |
|  |  | Conference | | CHI Conference on Human Factors in Computing Systems Extended Abstracts | | |
|  |  | Author/s | | MEETHA NESAM JAMES, NIMESHA RANASINGHE, ANTHONY TANG, LORA OEHLBERG | | |
|  |  | Date | | 2022-04-27 | | |
|  | Main content of the evidence | This paper presents a user study to evaluate the influence of six different types of video content (including nature, cooking, and a new food video genre known as mukbang) on people’s flavor perceptions in terms of taste sensations, liking, and emotions while eating plain white rice. Our findings revealed that the participants’ flavor perceptions are augmented based on different video content, indicating significant differences in their perceived taste sensations (e.g., increased perception of salty and spicy sensations). Furthermore, potential future implications are revealed to promote digital commensality and healthier eating habits.  This study hypothesized that different video content watched while eating positively influences participants’ flavor perception in terms of taste sensations, liking, and emotions.  The primary research question explored in this study was “Do Videos being watched while eating augment people’s flavors perceptions in terms of taste sensations, liking, and emotions?”. We conducted a user study with 35 participants who evaluated changes in their flavor perception with six different videos watched while eating white rice. | | | | |
|  | Participant details (eg, age/sex and number) | Age | | in the age range of 18 - 50 years (M = 26.1, S.D = 6.9) | | |
|  |  | Sex | | 46% Males, 51% females, 3% gender fluid | | |
|  |  | Final enrollment | | Thirty-five participants | | |
| Details/results extracted from source of evidence | Review question addressed  (Tick) | 1. | The health-related elements in *mukbang* (Food ads, food related videos) | | NA | |
|  |  | Source text | NA | | | |
|  |  | 2. | The relationship between *mukbang* (Food ads, food related videos) and health | | √ | |
|  |  | Source text | 1) It is hoped that viewers can improve their taste perception by watching mukbang, without having to eat the same food as the anchor in the video. This could also lead people to avoid indulgent or overeating habits just by watching a video to satisfy their cravings.  2) For people who need to eat very light, clean and healthy, like the patients, they could use mukbang to augments taste sensations, which avoid the need to physically or chemically alter the food, making this a healthier approach.  3) Related to positive emotions: conversational mukbang was correlated to ‘energetic’, ‘friendly’, ‘happy’, and ‘joyful’ emotions. Almost all the emotions reported while eating and watching videos were positive.  4) Mukbang videos could induce a calm mood, which were related to the calming effect of the ASMR induced by mukbangs. | | | |
|  |  | 3. | Impacts may *mukbang* (Food ads, food related videos) have on eating habits or eating behaviors | | NA | |
|  |  | Source text | NA | | | |
|  |  | 4. | The design, development, or usability of *mukbang* (Food ads, food related videos) watching interventions to demonstrate the usefulness of *mukbang* in the health field | | | NA |
|  |  | Source text | NA | | | |
|  |  | 5. | Evaluation of the health effects of various types of *mukbang* (Food ads, food related videos) | | | √ |
|  |  | Source text | 1) Other sensations, such as mouthwatering, craving, and virtual satiation also showed a significant difference with respect to different videos before tasting the sample.  2) The cooking video was related to ‘tender’, ‘good-natured’, ‘understanding’, and ‘polite’ emotions, which depicted that people could emote themselves with how the chef handled the food ingredients and prepared the food. Mukbang with sound video was associated with ‘good’, ‘quiet’, and ‘tame’ emotions. | | | |
|  |  | 6. | The challenges and barriers of integrating *mukbang* (Food ads, food related videos) videos into clinical practice | | | √ |
|  |  | Source text | The future work can explore potential health applications to reduce calorie intake while increasing the enjoyment of food and beverages. | | | |
|  |  | 7. | The advantages and/or disadvantages of *mukbang* (Food ads, food related videos) watching | | | NA |
|  |  | Source text | NA | | | |
|  | Research method/Tools | Using questionnaire to evaluate. | | | | |
|  | Characteristics of *mukbang* | There are four main styles of mukbang videos:  1) The mukbanger eating the food silently,  2) The mukbanger eating and talking about their flavor experience (taste, smell, appearance, texture),  3) The mukbanger sharing a story or about their life during eating, and  4) The mukbanger providing live responses to online viewers while broadcasting. | | | | |
|  | Health-related elements | NA | | | | |
|  | Summary of key findings related to health | This study was aim to investigate possible augmentations on taste sensations, liking, and emotions based on different videos watched while eating. Our findings revealed that 1) videos watched while eating augment the taste sensations without the need for physical or chemical flavorings, 2) different types of food-based videos influenced different types of taste sensations, 3) elicited emotions were different based on types of videos watched while eating, and 4) positive emotional changes were reported when food-based videos were watched (especially mukbang videos elicited an enjoyable eating experience). | | | | |

38. Dietary life and mukbang- and cookbang-watching status of university students majoring in food and nutrition before and after COVID-19 outbreak

|  | Items | Data | | | |
| --- | --- | --- | --- | --- | --- |
| Inclusion criteria | Population/Main research object | All students of the FN department of a college in Gyeonggi, Korea, participated in an online survey. | | | |
|  | Concept (Tick)  Studies focusing on | 1. | *mukbang* watching (Food ads, food related videos*) | | √ |
|  |  | 2. | the impact of *mukbang* (Food ads, food related videos) on health | | √ |
|  |  | 3. | the relationship between *mukbang* (Food ads, food related videos) and health | | √ |
|  |  | 4. | the design, development, or usability of *mukbang* (Food ads, food related videos) watching interventions to demonstrate the usefulness of *mukbang* in the health field | | NA |
|  |  | 5. | evaluating the effectiveness of various types of *mukbang* (Food ads, food related videos) for public health | | √ |
|  |  | 6. | the challenges and barriers of integrating *mukbang* (Food ads, food related videos) videos into clinical practice | | NA |
|  |  | 7. | the advantages and/or disadvantages of *mukbang* (Food ads, food related videos) watching | | √ |
|  | Types of evidence source | Quantitative research with an online survey. | | | |
| Evidence source details and characteristics | Citation details (eg, author/s, date, title, journal, volume, issue, pages) | Title | | Dietary life and mukbang- and cookbang-watching status of university students majoring in food and nutrition before and after COVID-19 outbreak | |
|  |  | Journal | | Journal of Nutrition and Health | |
|  |  | Author/s | | Hyunjoo Kang, Sowon Yun, and Hongmie Lee | |
|  |  | Date | | 2021 | |
|  | Main content of the evidence | this study sought to determine how the COVID-19 pandemic has impacted dietary life and the mukbang- and cookbang-watching patterns of college students, especially majoring in Food Nutrition, who equipped with a stronger orientation toward health. In this study, COVID-19 was the main influencing factor. | | | |
|  | Participant details (eg, age/sex and number) | Age | | College students | |
|  |  | Sex | | M/F = 37/130 | |
|  |  | Final enrollment | | A total of 167 students. | |
| Details/results extracted from source of evidence | Review question addressed  (Tick) | 1. | The health-related elements in *mukbang* (Food ads, food related videos) | | NA |
|  |  | Source text | NA | | |
|  |  | 2. | The relationship between *mukbang* (Food ads, food related videos) and health | | √ |
|  |  | Source text | 1) There is a growing awareness that mukbang is associated with unhealthy eating: more participants reported spending less time per day in watching mukbang in 2020 than in 2019, especially given that mukbang-watching has been related to an unhealthy diet.  2) About twice more FN-major students answered that viewing mukbang made them eat more from undesirable foods. | | |
|  |  | 3. | Impacts may *mukbang* (Food ads, food related videos) have on eating habits or eating behaviors | | √ |
|  |  | Source text | 1) In 2019 and 2020, 18.1% and 14.6% of participants, respectively, reported that watching mukbang worsened their diet while only 3.4% and 4.6% said that it improved their diet.  2) More people are spending more time on cookbang because of COVID-19, and more people say watching cookbang has significantly improved their eating habits compared to 2019.  3) Because of COVID-19, the frequency of watching mukbang has significantly higher, although the duration of viewing has declined. Compared with 2019, the number of viewers who believe that mukbang makes their diet worse decreased, and the number of viewers who believe that mukbang improves their diet increased relatively, but the extent was lower than that of cookbang.  4) Cookbang increases the frequency with which viewers cook for themselves, promoting healthier eating behaviors: the frequencies of eating alone and cooking were not different according to frequency of watching mukbang in both years, while frequent cookbang watchers more often cooked.  5) Mukbang-watching can prompt viewers to eat a certain kind of food. | | |
|  |  | 4. | The design, development, or usability of *mukbang* (Food ads, food related videos) watching interventions to demonstrate the usefulness of *mukbang* in the health field | | NA |
|  |  | Source text | NA | | |
|  |  | 5. | Evaluation of the health effects of various types of *mukbang* (Food ads, food related videos) | | √ |
|  |  | Source text | 1) According to this study, the results indicate that cookbang improved the participants’ overall diet rather than worsening it, while even more participants felt that mukbang worsened rather than improved it.  2) Undesirable diets were associated with frequently watching mukbang but not with cookbang from the selfevaluation. | | |
|  |  | 6. | The challenges and barriers of integrating *mukbang* (Food ads, food related videos) videos into clinical practice | | NA |
|  |  | Source text | NA | | |
|  |  | 7. | The advantages and/or disadvantages of *mukbang* (Food ads, food related videos) watching | | √ |
|  |  | Source text | Advantages of cookbang: watching cookbang increases the viewer's desire to try cooking for themselves, while also increasing the number of times to try to cook for themselves actually. | | |
|  | Research method/Tools | Questionnaires | | | |
|  | Characteristics of *mukbang* | Mukbang and cookbang | | | |
|  | Health-related elements | NA | | | |
|  | Summary of key findings related to health | 1) Our results are consistent to the previous studies in that COVID-19 pandemic could have improved the diets of certain groups of people.  2) Despite some limitations, this is the first report that the COVID-19 pandemic could have had positive impacts on the diet and lifestyle behaviors of young adults with a FN background. Furthermore, our work highlights the possibility of improving diet and health behaviors through proper education and the need to define groups that may be at a higher risk of unhealthy behaviors. | | | |

*Food ads and food related videos must include images of celebrities eating.

39. A study on the effect of one-person media viewing on diet due to binge eating - For women in their 20s and 30s

|  | Items | Data | | | |
| --- | --- | --- | --- | --- | --- |
| Inclusion criteria | Population/Main research object | 175 women in their 20s and 30s who had diet experience were asked to investigate the relationship between one-person media mukbang and diet, and to investigate the psychological effect of watching mukbang during the diet period on those who are on a diet and the behaviors that follow. | | | |
|  | Concept (Tick)  Studies focusing on | 1. | *mukbang* watching (Food ads, food related videos*) | | √ |
|  |  | 2. | the impact of *mukbang* (Food ads, food related videos) on health | | √ |
|  |  | 3. | the relationship between *mukbang* (Food ads, food related videos) and health | | √ |
|  |  | 4. | the design, development, or usability of *mukbang* (Food ads, food related videos) watching interventions to demonstrate the usefulness of *mukbang* in the health field | | NA |
|  |  | 5. | evaluating the effectiveness of various types of *mukbang* (Food ads, food related videos) for public health | | NA |
|  |  | 6. | the challenges and barriers of integrating *mukbang* (Food ads, food related videos) videos into clinical practice | | NA |
|  |  | 7. | the advantages and/or disadvantages of *mukbang* (Food ads, food related videos) watching | | √ |
|  | Types of evidence source | Thesis | | | |
| Evidence source details and characteristics | Citation details (eg, author/s, date, title, journal, volume, issue, pages) | Title | | A study on the effect of one-person media viewing on diet due to binge eating - For women in their 20s and 30s | |
|  |  | Journal | | Chung-Ang University Graduate School of Arts | |
|  |  | Author/s | | Dasom Lee | |
|  |  | Date | | 2019.8 | |
|  | Main content of the evidence | One of the purposes of this study is to find out the effect of viewing time and frequency of single-person media mukbang on binge eating. The study analyzed the information characteristics provided by these broadcast cooking programs, and the effects of mukbang on the changes in dietary habits were discussed. | | | |
|  | Participant details (eg, age/sex and number) | Age | | in their 20s and 30s | |
|  |  | Sex | | women | |
|  |  | Final enrollment | | A total of 175 people were sampled for this study. | |
| Details/results extracted from source of evidence | Review question addressed  (Tick) | 1. | The health-related elements in *mukbang* (Food ads, food related videos) | | √ |
|  |  | Source text | Most of the food handled in cooking and eating shows is designed to be cooked or eaten quickly, and is far from well-being. | | |
|  |  | 2. | The relationship between *mukbang* (Food ads, food related videos) and health | | √ |
|  |  | Source text | 1) When watching mukbangs for less than 10 minutes, 72.3% of those who did not lead to binge eating were higher at 72.3%, but when watching mukbangs for less than 30-1 hours, 70.6% responded that watching mukbangs led to binge eating. appeared as It can be seen that there is an experience that leads to impulsive food intake or binge eating according to the increase in the viewing time of watching mukbang.  2) Mukbang can be a great appetite enhancer, but these appetizing videos are not necessarily healthy, mukbang watching may led to impulsive food intake or binge eating, which has also found that mukbang not only gave viewers vicarious satisfaction, but also had negative effects such as inducing binge eating.  3) Watching mukbang leads to late-night binge eating, and mukbang watchers who experience this type of binge eating also experience negative emotions such as severe guilt and depression as a result of binge eating, which can lead to repeated dieting. | | |
|  |  | 3. | Impacts may *mukbang* (Food ads, food related videos) have on eating habits or eating behaviors | | √ |
|  |  | Source text |  | | |
|  |  | 4. | The design, development, or usability of *mukbang* (Food ads, food related videos) watching interventions to demonstrate the usefulness of *mukbang* in the health field | | NA |
|  |  | Source text |  | | |
|  |  | 5. | Evaluation of the health effects of various types of *mukbang* (Food ads, food related videos) | | NA |
|  |  | Source text |  | | |
|  |  | 6. | The challenges and barriers of integrating *mukbang* (Food ads, food related videos) videos into clinical practice | | NA |
|  |  | Source text |  | | |
|  |  | 7. | The advantages and/or disadvantages of *mukbang* (Food ads, food related videos) watching | | √ |
|  |  | Source text |  | | |
|  | Research method/Tools | Questionnaire survey | | | |
|  | Characteristics of *mukbang* | In mukbang videos, the food is close-up, attracting viewers with stimulating colors and sounds, and making them immerse themselves in the act of eating. | | | |
|  | Health-related elements | Fast food in mukbang videos, binge eating, sense of guilt. | | | |
|  | Summary of key findings related to health | 1) 66.7% of those who watched mukbang more than twice a day experienced binge eating, and 65.5% of those who watched the first episode of Mukbang said that they had an experience that led to binge eating. As the average number of watching Mukbang increased, it led to impulsive food intake or binge eating.  2) As a motivation for watching single-person media mukbangs, they answered that they refrain from eating while on a diet and watch for the purpose of feeling vicarious satisfaction.  3) Mukbang watching reasons:  ① You don't know what to eat.  ② Obtain vicarious satisfaction to ensure that you do not eat while on a diet;  ③ 68.4% of the people saw that the food in the video was delicious, and they would have the impulse to eat.  ④ 58.4 percent of the respondents said that although they were not hungry, they still had an appetite after watching the movie.  4) In a one-person media mukbang, the food is close-up, attracting viewers with stimulating colors and sounds, and making them immerse themselves in the act of eating. When watching such a video, it can be concluded that viewers do not stop at watching by being substituted for the actor eating food, but induce the action of eating it themselves. | | | |

*Food ads and food related videos must include images of celebrities eating.

40. A Comparative Study on the Eating Behavior of Adults over 20 According to the Viewing time of Eating Shows

|  | Items | Data | | | |
| --- | --- | --- | --- | --- | --- |
| Inclusion criteria | Population/Main research object | 800 adults (400 men and 400 women) aged between 20 to 50 were participant in this study. | | | |
|  | Concept (Tick)  Studies focusing on | 1. | *mukbang* watching (Food ads, food related videos*) | | √ |
|  |  | 2. | the impact of *mukbang* (Food ads, food related videos) on health | | √ |
|  |  | 3. | the relationship between *mukbang* (Food ads, food related videos) and health | | √ |
|  |  | 4. | the design, development, or usability of *mukbang* (Food ads, food related videos) watching interventions to demonstrate the usefulness of *mukbang* in the health field | | √ |
|  |  | 5. | evaluating the effectiveness of various types of *mukbang* (Food ads, food related videos) for public health | | NA |
|  |  | 6. | the challenges and barriers of integrating *mukbang* (Food ads, food related videos) videos into clinical practice | | NA |
|  |  | 7. | the advantages and/or disadvantages of *mukbang* (Food ads, food related videos) watching | | √ |
|  | Types of evidence source | Cross-sectional study | | | |
| Evidence source details and characteristics | Citation details (eg, author/s, date, title, journal, volume, issue, pages) | Title | | A Comparative Study on the Eating Behavior of Adults over 20 According to the Viewing time of Eating Shows | |
|  |  | Journal | | Korean Journal of Community Nutrition | |
|  |  | Author/s | | Ha-Yan Nam, Bok-Mi Jung | |
|  |  | Date | | 2021 | |
|  | Main content of the evidence | This study was conducted to investigate the relationship between watching mukbang (eating broadcasts) and dietary and health behavior in adults who watch mukbang.  This study investigated mukbang watching time through a survey targeting 800 adult men and women who had experience in mukbang watching, and found out whether the mukbang watching time was correlated with eating behavior, and developed and developed programs to cope with the effects on mukbang viewers. It was intended to provide basic data to solve the problem of obesity. | | | |
|  | Participant details (eg, age/sex and number) | Age | | in their 20s and 50s | |
|  |  | Sex | | Both | |
|  |  | Final enrollment | | 800 adults (400 men and 400 women) | |
| Details/results extracted from source of evidence | Review question addressed  (Tick) | 1. | The health-related elements in *mukbang* (Food ads, food related videos) | | NA |
|  |  | Source text | NA | | |
|  |  | 2. | The relationship between *mukbang* (Food ads, food related videos) and health | | √ |
|  |  | Source text | 1) It was suggested that the longer it was used, the higher the probability of obesity-related eating habit problems.  2) More frequent mukbang viewing predicted more frequent skipping of breakfast, increased intake, and increased late-night snacks.  3) It was found that those who spent a lot of time watching mukbang had a low interest in health, and the number of times of exercise was also very low compared to those who watched a low amount of time. | | |
|  |  | 3. | Impacts may *mukbang* (Food ads, food related videos) have on eating habits or eating behaviors | | √ |
|  |  | Source text | 1) Since mukbangs are based on food, they can affect the food choices and intake of people who watch mukbangs, leading to obesity.  2) Previous studies have found that mukbang watching can form undesirable consumption patterns and relatively poor eating habits. | | |
|  |  | 4. | The design, development, or usability of *mukbang* (Food ads, food related videos) watching interventions to demonstrate the usefulness of *mukbang* in the health field | | √ |
|  |  | Source text | From the perspective of mukbang broadcasting media, efforts are required for healthy broadcasting for the health of viewers rather than focusing only on popularity or marketing. | | |
|  |  | 5. | Evaluation of the health effects of various types of *mukbang* (Food ads, food related videos) | | NA |
|  |  | Source text | NA | | |
|  |  | 6. | The challenges and barriers of integrating *mukbang* (Food ads, food related videos) videos into clinical practice | | NA |
|  |  | Source text | NA | | |
|  |  | 7. | The advantages and/or disadvantages of *mukbang* (Food ads, food related videos) watching | | √ |
|  |  | Source text | Disadvantages: Watching mukbang videos for long periods of time led to weight gain in both men and women. | | |
|  | Research method/Tools | Online and offline survey | | | |
|  | Characteristics of *mukbang* |  | | | |
|  | Health-related elements | Skipping breakfast, binge eating, easier to become overweight or obesity. | | | |
|  | Summary of key findings related to health | 1) Most of the participants (39.7%) watched mukbang less than 7 hours per week, with 241 people watched more than 7 hours per week.  2) Watching mukbang for a long period of time may affect viewers' dietary preferences, making them prefer staple food, sweet foods with high calories and single nutrients.  3) In this study, people who watched mukbangs for a high number of hours per week showed a higher preference for rice, noodles, bread, and meat, but lower preference for vegetables and fruits, compared to those with a low number of watching hours per week.  4) In the study, people who watched mukbang for a long time had a high rate of skipping breakfast, but it is difficult to conclude that these results lead to obesity.  5) In the case of people who spend a lot of time watching mukbangs, it can be thought that the frequency of eating delivered food and late-night snacks may increase due to an increase in the desire to eat menus aired in mukbangs.  6) When the weekly mukbang watching time was high, the preference for carbohydrate foods and meat was high, whereas when the mukbang watching time was low, the preference for vegetables and fruits was high.  7) The intake of delivery food and the frequency of eating food for late-night snacking were also higher in cases where the watching time for mukbang was high compared to cases in which it was low.  8) As a result of this study, viewers who spend a lot of time watching Mukbang show undesirable health and eating behaviors, so it seems necessary to raise awareness of Mukbang viewers on proper eating habits and proper nutrition education for lifestyle. | | | |

*Food ads and food related videos must include images of celebrities eating.

41. A Study on the Use of YouTube Food Contents and Delivery Food Intake of University Students in Gwangju

|  | Items | Data | | | |
| --- | --- | --- | --- | --- | --- |
| Inclusion criteria | Population/Main research object | A total of 247 college students living in Gwangju were surveyed and analyzed with 86 male students and 161 female students, and the main findings were as follows. | | | |
|  | Concept (Tick)  Studies focusing on | 1. | *mukbang* watching (Food ads, food related videos*) | | √ |
|  |  | 2. | the impact of *mukbang* (Food ads, food related videos) on health | | √ |
|  |  | 3. | the relationship between *mukbang* (Food ads, food related videos) and health | | √ |
|  |  | 4. | the design, development, or usability of *mukbang* (Food ads, food related videos) watching interventions to demonstrate the usefulness of *mukbang* in the health field | | NA |
|  |  | 5. | evaluating the effectiveness of various types of *mukbang* (Food ads, food related videos) for public health | | NA |
|  |  | 6. | the challenges and barriers of integrating *mukbang* (Food ads, food related videos) videos into clinical practice | | NA |
|  |  | 7. | the advantages and/or disadvantages of *mukbang* (Food ads, food related videos) watching | | NA |
|  | Types of evidence source | Thesis | | | |
| Evidence source details and characteristics | Citation details (eg, author/s, date, title, journal, volume, issue, pages) | Title | | A Study on the Use of YouTube Food Contents and Delivery Food Intake of University Students in Gwangju | |
|  |  | Journal | | Chosun University | |
|  |  | Author/s | | Park Somi | |
|  |  | Date | | 2022.4 | |
|  | Main content of the evidence | This study was conducted from September 15, 2021 to September 29, 2021 to find out the use of YouTube content and the intake of delivery food. The main content of this paper is to study the eating habits of college students in Guangzhou, South Korea, including their awareness and use of take-away food, and an analysis of their satisfaction when watching YouTube food content. The study also explores the impact of these factors on college students' eating behaviors and puts forward some relevant policy recommendations. | | | |
|  | Participant details (eg, age/sex and number) | Age | | Covers the characteristics of different ages, grades, majors, etc. | |
|  |  | Sex | | Both | |
|  |  | Final enrollment | | A total of 247 participants were recruited from college students living in Gwangju area, including 86 males and 161 females, aged from 19 to 29 years. | |
| Details/results extracted from source of evidence | Review question addressed  (Tick) | 1. | The health-related elements in *mukbang* (Food ads, food related videos) | | √ |
|  |  | Source text | 1) The health-related content in this paper includes college students' health awareness, health behaviors, eating habits, etc. | | |
|  |  | 2. | The relationship between *mukbang* (Food ads, food related videos) and health | | √ |
|  |  | Source text | 1. The study has analyzed college students' awareness and use of takeaway food, as well as their concern about food safety and nutrition. 2. After watching mukbang, men were more likely to order high-oil, high-calorie takeout of fried chicken than women. Women tended to choose carbohydrates or sweets. The study also found that men had a higher awareness of healthy eating than women. 3. There was a positive correlation between the satisfaction of watching food content and health awareness, takeaway food awareness, and diet guide, respectively. The higher the satisfaction of food culture information, the higher the level of health awareness, takeaway food awareness, and diet guide. | | |
|  |  | 3. | Impacts may *mukbang* (Food ads, food related videos) have on eating habits or eating behaviors | | √ |
|  |  | Source text | 1. The study also explored college students’ viewing of YouTube food content and analyzed the impact of this content on college students’ eating behaviors. 2. The popularity of mukbang has made it widely concerned and blindly loved by college students, which has a certain degree of influence on their eating behavior, the frequency of ordering takeout and food choice. | | |
|  |  | 4. | The design, development, or usability of *mukbang* (Food ads, food related videos) watching interventions to demonstrate the usefulness of *mukbang* in the health field | | NA |
|  |  | Source text | NA | | |
|  |  | 5. | Evaluation of the health effects of various types of *mukbang* (Food ads, food related videos) | | √ |
|  |  | Source text | The study involved mukbang and cooking shows, as well as other YouTube videos with food content. | | |
|  |  | 6. | The challenges and barriers of integrating *mukbang* (Food ads, food related videos) videos into clinical practice | | NA |
|  |  | Source text | NA | | |
|  |  | 7. | The advantages and/or disadvantages of *mukbang* (Food ads, food related videos) watching | | √ |
|  |  | Source text |  | | |
|  | Research method/Tools | The main research method of this paper is questionnaire survey. The study used a questionnaire developed by the research team, which covered multiple aspects, including personal characteristics, eating behavior, take-away food consumption, YouTube food content viewing, etc. The survey was conducted through an online self-administered questionnaire, and the survey period was from September 15, 2021 to September 29, 2021. The study also conducted statistical analysis and descriptive analysis on the questionnaire data. | | | |
|  | Characteristics of *mukbang* | In this paper, YouTube food content refers to food-related video content published on the YouTube platform, including food preparation, food reviews, restaurant recommendations, etc. The study explored college students’ viewing of YouTube food content and analyzed the impact of this content on college students’ eating behaviors. | | | |
|  | Health-related elements | Health awareness, takeaway food awareness, and diet guide | | | |
|  | Summary of key findings related to health | 1) This study found that college students consume higher amounts of take-away food, and they are more inclined to choose food that tastes good and is reasonably priced.  2) Watching YouTube food content has a certain impact on college students' eating behaviors, but it is not a decisive factor.  3) There are certain differences in college students' awareness and usage of take-out food, and male and female students also differ in this regard.  4) The eating behavior of college students is closely related to their personal characteristics, lifestyle and other factors. | | | |

*Food ads and food related videos must include images of celebrities eating.

42. A Study on the Use of YouTube Food Contents and Delivery Food Intake of University Students in Gwangju

|  | Items | Data | | | |
| --- | --- | --- | --- | --- | --- |
| Inclusion criteria | Population/Main research object | College students from a certain area in South Korea, who mainly live in that area. There is no restriction on gender, and it covers the characteristics of different ages, different grades, different majors, etc. | | | |
|  | Concept (Tick)  Studies focusing on | 1. | *mukbang* watching (Food ads, food related videos*) | | √ |
|  |  | 2. | the impact of *mukbang* (Food ads, food related videos) on health | | √ |
|  |  | 3. | the relationship between *mukbang* (Food ads, food related videos) and health | | √ |
|  |  | 4. | the design, development, or usability of *mukbang* (Food ads, food related videos) watching interventions to demonstrate the usefulness of *mukbang* in the health field | | NA |
|  |  | 5. | evaluating the effectiveness of various types of *mukbang* (Food ads, food related videos) for public health | | NA |
|  |  | 6. | the challenges and barriers of integrating *mukbang* (Food ads, food related videos) videos into clinical practice | | NA |
|  |  | 7. | the advantages and/or disadvantages of *mukbang* (Food ads, food related videos) watching | | NA |
|  | Types of evidence source | Thesis | | | |
| Evidence source details and characteristics | Citation details (eg, author/s, date, title, journal, volume, issue, pages) | Title | | A Study on the Use of YouTube Food Contents and Delivery Food Intake of University Students in Gwangju | |
|  |  | Journal | | Chosun University | |
|  |  | Author/s | | Park Somi | |
|  |  | Date | | 2022.4 | |
|  | Main content of the evidence | The main content of this paper is to study the eating habits of college students in Guangzhou, South Korea, including their awareness and use of take-away food, and an analysis of their satisfaction when watching YouTube food content. The study also explores the impact of these factors on college students' eating behaviors and puts forward some relevant policy recommendations. | | | |
|  | Participant details (eg, age/sex and number) | Age | | Covers the characteristics of different ages, grades, majors, etc. | |
|  |  | Sex | | Both | |
|  |  | Final enrollment | | A total of 247 subjects were recruited from college students living in Gwangju area, including 86 males and 161 females, aged from 19 to 29 years. | |
| Details/results extracted from source of evidence | Review question addressed  (Tick) | 1. | The health-related elements in *mukbang* (Food ads, food related videos) | | √ |
|  |  | Source text | 1) The health-related content in this paper includes college students' health awareness, health behaviors, eating habits, etc. | | |
|  |  | 2. | The relationship between *mukbang* (Food ads, food related videos) and health | | √ |
|  |  | Source text | 1) The study has analyzed college students' awareness and use of takeaway food, as well as their concern about food safety and nutrition. | | |
|  |  | 3. | Impacts may *mukbang* (Food ads, food related videos) have on eating habits or eating behaviors | | √ |
|  |  | Source text | The study also explored college students’ viewing of YouTube food content and analyzed the impact of this content on college students’ eating behaviors. | | |
|  |  | 4. | The design, development, or usability of *mukbang* (Food ads, food related videos) watching interventions to demonstrate the usefulness of *mukbang* in the health field | | NA |
|  |  | Source text | NA | | |
|  |  | 5. | Evaluation of the health effects of various types of *mukbang* (Food ads, food related videos) | | NA |
|  |  | Source text | NA | | |
|  |  | 6. | The challenges and barriers of integrating *mukbang* (Food ads, food related videos) videos into clinical practice | | NA |
|  |  | Source text | NA | | |
|  |  | 7. | The advantages and/or disadvantages of *mukbang* (Food ads, food related videos) watching | | NA |
|  |  | Source text | NA | | |
|  | Research method/Tools | The main research method of this paper is questionnaire survey. The study used a questionnaire developed by the research team, which covered multiple aspects, including personal characteristics, eating behavior, take-away food consumption, YouTube food content viewing, etc. The survey was conducted through an online self-administered questionnaire, and the survey period was from September 15, 2021 to September 29, 2021. The study also conducted statistical analysis and descriptive analysis on the questionnaire data. | | | |
|  | Characteristics of *mukbang* | In this paper, YouTube food content refers to food-related video content published on the YouTube platform, including food preparation, food reviews, restaurant recommendations, etc. The study explored college students’ viewing of YouTube food content and analyzed the impact of this content on college students’ eating behaviors. | | | |
|  | Health-related elements |  | | | |
|  | Summary of key findings related to health | 1) This study found that college students consume higher amounts of take-away food, and they are more inclined to choose food that tastes good and is reasonably priced.  2) Watching YouTube food content has a certain impact on college students' eating behaviors, but it is not a decisive factor.  3) There are certain differences in college students' awareness and usage of take-out food, and male and female students also differ in this regard.  4) The eating behavior of college students is closely related to their personal characteristics, lifestyle and other factors. | | | |

*Food ads and food related videos must include images of celebrities eating.

43. A Study on the Cookbang YouTube Program Use and Dietary Change based on the Technology Acceptance Model

|  | Items | Data | | | | |
| --- | --- | --- | --- | --- | --- | --- |
| Inclusion criteria | Population/Main research object | The research participants in this study were 214 Korean adults who had experience using the Cookbang YouTube program. The participants' education levels varied, with 62.1% having completed college, and 51.8% were office workers. | | | | |
|  | Concept (Tick)  Studies focusing on | 1. | *mukbang* watching (Food ads, food related videos*) | | √ | |
|  |  | 2. | the impact of *mukbang* (Food ads, food related videos) on health | | √ | |
|  |  | 3. | the relationship between *mukbang* (Food ads, food related videos) and health | | √ | |
|  |  | 4. | the design, development, or usability of *mukbang* (Food ads, food related videos) watching interventions to demonstrate the usefulness of *mukbang* in the health field | | √ | |
|  |  | 5. | evaluating the effectiveness of various types of *mukbang* (Food ads, food related videos) for public health | | NA | |
|  |  | 6. | the challenges and barriers of integrating *mukbang* (Food ads, food related videos) videos into clinical practice | | NA | |
|  |  | 7. | the advantages and/or disadvantages of *mukbang* (Food ads, food related videos) watching | | NA | |
|  | Types of evidence source | cross-sectional study | | | | |
| Evidence source details and characteristics | Citation details (eg, author/s, date, title, journal, volume, issue, pages) | Title | | A Study on the Cookbang YouTube Program Use and Dietary Change based on the Technology Acceptance Model | | |
|  |  | Journal | | Culinary Science & Hospitality Research | | |
|  |  | Author/s | | Sung-Jin Jo, Ho-Jung Choi | | |
|  |  | Date | | 2021 | | |
|  | Main content of the evidence | In this study, the author used the Cookbang YouTube program as a research object to explore the impact of watching the program on eating habits, and analyzed the impact of viewers' perceived interest and usefulness of the program on their continued use and changes in eating habits.  Through a survey and empirical analysis of consumers who watch Cookbang YouTube programs, the study found that perceived enjoyment and perceived usefulness have a significant positive impact on the intention to continue using and changes in eating habits. In addition, intention to continue use also had a significant positive impact on changes in dietary habits. These results can provide useful data for analyzing the relationship between perceived enjoyment and usefulness of consumers using Cookbang YouTube programs, thereby providing a useful reference for building detailed marketing strategies for each customer. | | | | |
|  | Participant details (eg, age/sex and number) | Age | | an age range of 20-49 years | | |
|  |  | Sex | | the final sample included 93 males and 121 females | | |
|  |  | Final enrollment | | The researchers used a convenience sampling method to distribute 240 questionnaires and excluded those that were incomplete or did not meet the research purpose, resulting in a final sample size of 214. | | |
| Details/results extracted from source of evidence | Review question addressed  (Tick) | 1. | The health-related elements in *mukbang* (Food ads, food related videos) | | √ | |
|  |  | Source text |  | | | |
|  |  | 2. | The relationship between *mukbang* (Food ads, food related videos) and health | | √ | |
|  |  | Source text | Cookbang YouTube programs can be a useful tool for promoting healthy eating habits and improving cooking skills. | | | |
|  |  | 3. | Impacts may *mukbang* (Food ads, food related videos) have on eating habits or eating behaviors | | √ | |
|  |  | Source text | 1) According to the study, perceived pleasure and perceived usefulness have a significant positive effect on dietary changes. In other words, people who enjoy and find Cookbang YouTube programs useful are more likely to make changes to their dietary habits.  2) This suggests that people who plan to keep watching Cookbang YouTube programs are more likely to make changes to their diets.  3) the study found that Cookbang YouTube programs can positively influence dietary changes. | | | |
|  |  | 4. | The design, development, or usability of *mukbang* (Food ads, food related videos) watching interventions to demonstrate the usefulness of *mukbang* in the health field | | | √ |
|  |  | Source text | 1) For future studies: organizations could consider partnering with Cookbang YouTube programs or creating similar content to promote healthy eating habits.  2) For future studies: organizations could focus on creating enjoyable and useful content to encourage people to continue watching and making dietary changes. | | | |
|  |  | 5. | Evaluation of the health effects of various types of *mukbang* (Food ads, food related videos) | | | NA |
|  |  | Source text | NA | | | |
|  |  | 6. | The challenges and barriers of integrating *mukbang* (Food ads, food related videos) videos into clinical practice | | | NA |
|  |  | Source text | NA | | | |
|  |  | 7. | The advantages and/or disadvantages of *mukbang* (Food ads, food related videos) watching | | | NA |
|  |  | Source text | NA | | | |
|  | Research method/Tools | a survey-based empirical analysis.  the researchers conducted a correlation analysis and hypothesis testing to examine the causal relationship between variables. | | | | |
|  | Characteristics of *mukbang* | Cookbang YouTube refers to a cooking YouTube program that is very popular in Korea. The program shows cooking processes and techniques through videos, and introduces viewers to the preparation methods of various Korean traditional and modern dishes.  The research object of this paper is the Cookbang YouTube program, which is a popular cooking program in Korea that introduces various traditional and modern Korean dishes and their preparation methods through videos. | | | | |
|  | Health-related elements | The study also suggests that frequent eating out and skipping breakfast can lead to nutrient overconsumption, imbalanced diets, and obesity. The service industry, including the food and nutrition sectors, is evolving rapidly in Korea, and the trend towards eating out and convenience foods is increasing. | | | | |
|  | Summary of key findings related to health | 1) The technology acceptance model is a theoretical framework that explains how users come to accept and use new technologies. It suggests that perceived usefulness and perceived ease of use are key factors that influence users' attitudes towards technology and their intention to use it. In the context of this study, the technology acceptance model was used to explore the relationship between users' perceptions of Cookbang YouTube programs and their dietary changes. The study found that perceived pleasure and perceived usefulness have a significant positive effect on intention to continue use, and that intention to continue use has a significant positive effect on dietary changes. Therefore, the technology acceptance model is relevant to this study because it helps explain how users' perceptions of Cookbang YouTube programs influence their behavior and dietary habits. | | | | |

*Food ads and food related videos must include images of celebrities eating.

44. A Study on Food and Nutrition-related Media Consumption and Its Influence on Dietary Habits of Adolescents and Adults in Daegu and Gyeongbuk Regio

|  | Items | Data | | | | |
| --- | --- | --- | --- | --- | --- | --- |
| Inclusion criteria | Population/Main research object | The study surveyed adolescents and adults in the Daegu and Gyeongbuk regions. The number of male and female participants was almost the same, most of them were 20-30 years old, and most of the adolescents were junior and senior high school students. | | | | |
|  | Concept (Tick)  Studies focusing on | 1. | *mukbang* watching (Food ads, food related videos*) | | √ | |
|  |  | 2. | the impact of *mukbang* (Food ads, food related videos) on health | | √ | |
|  |  | 3. | the relationship between *mukbang* (Food ads, food related videos) and health | | √ | |
|  |  | 4. | the design, development, or usability of *mukbang* (Food ads, food related videos) watching interventions to demonstrate the usefulness of *mukbang* in the health field | | NA | |
|  |  | 5. | evaluating the effectiveness of various types of *mukbang* (Food ads, food related videos) for public health | | √ | |
|  |  | 6. | the challenges and barriers of integrating *mukbang* (Food ads, food related videos) videos into clinical practice | | NA | |
|  |  | 7. | the advantages and/or disadvantages of *mukbang* (Food ads, food related videos) watching | | NA | |
|  | Types of evidence source | master's thesis, major in Nutrition Education | | | | |
| Evidence source details and characteristics | Citation details (eg, author/s, date, title, journal, volume, issue, pages) | Title | | A Study on Food and Nutrition-related Media Consumption and Its Influence on Dietary Habits of Adolescents and Adults in Daegu and Gyeongbuk Regio | | |
|  |  | Journal | | NA | | |
|  |  | Author/s | | Kwon Young Shin | | |
|  |  | Date | | 2021.6 | | |
|  | Main content of the evidence | This thesis has investigated the relationship between media consumption and dietary habits and nutrition knowledge among adolescents and adults in the Daegu-Gyeongbuk region. The study investigated the effects of media consumption on dietary habits, food preferences, and nutritional knowledge. The study also found that there is excessive promotion of certain foods in the media, which may have a negative impact on people's food choices. Therefore, the thesis suggests that there is a need for more scientific and careful regulation and management of media to improve public health and nutrition. | | | | |
|  | Participant details (eg, age/sex and number) | Age | | 10-50 years old. | | |
|  |  | Sex | | Both | | |
|  |  | Final enrollment | | A total of 341 participants have joined in the study. | | |
| Details/results extracted from source of evidence | Review question addressed  (Tick) | 1. | The health-related elements in *mukbang* (Food ads, food related videos) | | NA | |
|  |  | Source text | NA | | | |
|  |  | 2. | The relationship between *mukbang* (Food ads, food related videos) and health | | √ | |
|  |  | Source text | Excessive stimulant content and promotion of specific foods in the media can negatively impact people's dietary choices. | | | |
|  |  | 3. | Impacts may *mukbang* (Food ads, food related videos) have on eating habits or eating behaviors | | √ | |
|  |  | Source text | The media has a significant influence on people's eating habits and nutritional knowledge, especially among teenagers who are in their 20s. | | | |
|  |  | 4. | The design, development, or usability of *mukbang* (Food ads, food related videos) watching interventions to demonstrate the usefulness of *mukbang* in the health field | | | √ |
|  |  | Source text | For future studies: it is of great importance that nutrition educations through schools, workplaces, and communities are expanded in order to prevent individuals from being affected by misled information or unhealthy eating habits. | | | |
|  |  | 5. | Evaluation of the health effects of various types of *mukbang* (Food ads, food related videos) | | | NA |
|  |  | Source text | NA | | | |
|  |  | 6. | The challenges and barriers of integrating *mukbang* (Food ads, food related videos) videos into clinical practice | | | NA |
|  |  | Source text | NA | | | |
|  |  | 7. | The advantages and/or disadvantages of *mukbang* (Food ads, food related videos) watching | | | NA |
|  |  | Source text | NA | | | |
|  | Research method/Tools | The research methods of this paper include questionnaire survey and statistical analysis. | | | | |
|  | Characteristics of *mukbang* | nutrition-related media.  “Mukbang” content, which is also known as an eating show or food intake show.  food and nutrition-related media shown in YouTube (Internet) and TV | | | | |
|  | Health-related elements | NA | | | | |
|  | Summary of key findings related to health | 1) The study also found that there is excessive promotion of certain foods in the media, which may have a negative impact on people's food choices.  2) The thesis concludes that media has a significant impact on people's dietary habits and nutrition knowledge, especially among young people and those in their 20s.  3) In the case of “Mukbang” content, which is also known as an eating show or food intake show, there was no significant difference in the frequency of viewing by gender, age, or food spendings.  4) More than the majority of the people surveyed answered that they are watching“Mukbang” content on account of their interests in cooking, food, and information about must-eat restaurants.  5) The younger the respondents’ age, the more they were seeking this content to gain vicarious satisfaction. Especially, for teenagers, the motivation to watch this content was to soothe hunger.  6) A considerable number of the surveyed answered that they are watching these instructive videos to acquire new and useful information when buying food or cooking.  7) In terms of the awareness of how much media with nutritional information affects their eating habits as well as how beneficial this content is, women compared with men, and also subjects with higher nutritional knowledge scores had higher awareness, regardless of the types of programs provided.  8) The result exhibits that a great number of respondents had experienced purchasing exactly the same or similar food after watching eating shows (87.0%) and shows with nutritional guidance (72.0%), which indicates that food-related media consumption might have a crucial impact on people’s dietary habits.  9) A great number of teens and 20s watch food and nutrition-related media to get the information necessary to maintain their ideal body shape. Meanwhile, many teenagers search for eating shows to soothe their hunger. Taking these into considerations, education on how to selectively purchase or cook food for healthy eating is imperative. | | | | |

*Food ads and food related videos must include images of celebrities eating.

45. The Effects of Watching Mukbang and Inducing Anxiety on Eating Behavior.

|  | Items | Data | | | | |
| --- | --- | --- | --- | --- | --- | --- |
| Inclusion criteria | Population/Main research object | 51 college students and graduate students attending a university were randomly assigned to each experimental condition. Among these, 8 people who came without eating or gave insincere answers to the manipulation check questions were excluded from the analysis. | | | | |
|  | Concept (Tick)  Studies focusing on | 1. | *mukbang* watching (Food ads, food related videos*) | | √ | |
|  |  | 2. | the impact of *mukbang* (Food ads, food related videos) on health | | NA | |
|  |  | 3. | the relationship between *mukbang* (Food ads, food related videos) and health | | √ | |
|  |  | 4. | the design, development, or usability of *mukbang* (Food ads, food related videos) watching interventions to demonstrate the usefulness of *mukbang* in the health field | | NA | |
|  |  | 5. | evaluating the effectiveness of various types of *mukbang* (Food ads, food related videos) for public health | | NA | |
|  |  | 6. | the challenges and barriers of integrating *mukbang* (Food ads, food related videos) videos into clinical practice | | NA | |
|  |  | 7. | the advantages and/or disadvantages of *mukbang* (Food ads, food related videos) watching | | NA | |
|  | Types of evidence source | A literary master's thesis | | | | |
| Evidence source details and characteristics | Citation details (eg, author/s, date, title, journal, volume, issue, pages) | Title | | The Effects of Watching Mukbang and Inducing Anxiety on Eating Behavior. | | |
|  |  | Journal | | Daegu University | | |
|  |  | Author/s | | Soyeon Kim | | |
|  |  | Date | | 2020.08 | | |
|  | Main content of the evidence | The thesis examines the impact of watching mukbang and inducing anxiety on eating behavior, and tests the following hypotheses:  1. Watching mukbang has a positive effect on eating behavior.  2. Inducing anxiety has a positive effect on eating behavior.  3. Watching mukbang and inducing anxiety have a positive interaction effect on eating behavior.  Participants were randomly assigned to the anxiety-induced group and the neutral-induced group. In the first phase of the experiment, according to the different groups, the participants watched different emotion-induced videos. Then, the participants were randomly assigned to the food video group and the general video group. Among them, the food video played a French fries mukbang video. Finally, the participants were given chips and an evaluation sheet to eat the chips naturally and give a taste evaluation. After the assessment, participants were brought back to the laboratory to fill out a questionnaire and answer questions. | | | | |
|  | Participant details (eg, age/sex and number) | Age | | NA | | |
|  |  | Sex | | Both | | |
|  |  | Final enrollment | | a total of 43 people participated in the analysis | | |
| Details/results extracted from source of evidence | Review question addressed  (Tick) | 1. | The health-related elements in *mukbang* (Food ads, food related videos) | | NA | |
|  |  | Source text | NA | | | |
|  |  | 2. | The relationship between *mukbang* (Food ads, food related videos) and health | | √ | |
|  |  | Source text |  | | | |
|  |  | 3. | Impacts may *mukbang* (Food ads, food related videos) have on eating habits or eating behaviors | | √ | |
|  |  | Source text | 1) Anxiety significantly lowers appetite and food intake, so when anxiety is high, it can be expected that people will not consume food even when exposed to external food-related cues such as mukbang.  2) Mukbang watching had higher appetite and food intake when they were in a neutral mood compared to those in an anxious mood. This means that when you have a neutral emotion, you can expect to eat based on external cues, and when you have a negative emotion, you can think of the possibility that you will be more affected by emotions than external cues such as mukbang. | | | |
|  |  | 4. | The design, development, or usability of *mukbang* (Food ads, food related videos) watching interventions to demonstrate the usefulness of *mukbang* in the health field | | | NA |
|  |  | Source text | NA | | | |
|  |  | 5. | Evaluation of the health effects of various types of *mukbang* (Food ads, food related videos) | | | NA |
|  |  | Source text | NA | | | |
|  |  | 6. | The challenges and barriers of integrating *mukbang* (Food ads, food related videos) videos into clinical practice | | | NA |
|  |  | Source text | NA | | | |
|  |  | 7. | The advantages and/or disadvantages of *mukbang* (Food ads, food related videos) watching | | | NA |
|  |  | Source text | NA | | | |
|  | Research method/Tools | Customized videos and questionnaires | | | | |
|  | Characteristics of *mukbang* | French fries mukbang video | | | | |
|  | Health-related elements | NA | | | | |
|  | Summary of key findings related to health | 1) Food videos watching did not lead to greater appetite and food intake.  2) Watching neither food videos nor videos in general resulted in emotional effects on appetite and food intake.  3) Combined with previous studies, there is no established conclusion on the effect of mukbang on appetite. Watching mukbang videos may promote appetite, but it may also reduce or have no effect on appetite.  4) This suggests that the appropriate video length for stimulating desire or focusing attention through video is about 10 to 15 minutes. Therefore, it is necessary to adjust the video duration to be longer in future studies.  5) The main effect of emotion on intake was statistically significant, but the relationship between emotion and appetite was not significant.  6) The anxiety-eliciting group would show a higher appetite and more food intake than the neutral emotion-eliciting group, the anxiety-eliciting group showed a lower appetite and less food intake than the control group. This is thought to show that inducing anxious emotions actually reduced eating behavior.  7) In this study, contrary to the hypothesis, it can be said that anxiety lowered appetite and food intake.  8) These results predict that depression, anxiety, and anger, which are representative negative emotions, will have different effects on eating behavior.  9) Although this study showed that anxiety reduces food intake, there are many research results showing that depression affects binge eating or overeating. Therefore, it can be expected that the impact on eating behavior will vary depending on the type of negative emotion.  10) For both mukbang videos and general videos, the anxiety-inducing condition had low appetite and food intake. Therefore, Hypothesis 3 was not supported. | | | | |

*Food ads and food related videos must include images of celebrities eating.

46. A study on the influence of information characteristics of TV cooking program on its information acceptance and dietary change

|  | Items | Data | | | | |
| --- | --- | --- | --- | --- | --- | --- |
| Inclusion criteria | Population/Main research object | This study surveyed individuals who watch cooking programs on television. Of 220 respondents, 31 (14.1%) were men and 189 (85.9%) were women, most were in their 40s, and most were married. Most of the participants had college degrees, did not have regular jobs, and lived in various regions of Korea. | | | | |
|  | Concept (Tick)  Studies focusing on | 1. | *mukbang* watching (Food ads, food related videos*) | | √ | |
|  |  | 2. | the impact of *mukbang* (Food ads, food related videos) on health | | √ | |
|  |  | 3. | the relationship between *mukbang* (Food ads, food related videos) and health | | √ | |
|  |  | 4. | the design, development, or usability of *mukbang* (Food ads, food related videos) watching interventions to demonstrate the usefulness of *mukbang* in the health field | | NA | |
|  |  | 5. | evaluating the effectiveness of various types of *mukbang* (Food ads, food related videos) for public health | | NA | |
|  |  | 6. | the challenges and barriers of integrating *mukbang* (Food ads, food related videos) videos into clinical practice | | NA | |
|  |  | 7. | the advantages and/or disadvantages of *mukbang* (Food ads, food related videos) watching | | √ | |
|  | Types of evidence source | Thesis, major in The Graduate School of Management | | | | |
| Evidence source details and characteristics | Citation details (eg, author/s, date, title, journal, volume, issue, pages) | Title | | A study on the influence of information characteristics of TV cooking program on its information acceptance and dietary change | | |
|  |  | Journal | | Sejong Cyber University | | |
|  |  | Author/s | | Sunyoung Yoon | | |
|  |  | Date | | 2017.02 | | |
|  | Main content of the evidence | This is a study on the impact of the information characteristics of TV cooking programs on information reception and dietary changes. The study investigates the influence of TV cooking programs on our eating habits and provides very useful information. This study has taken cooking shows among TV cooking programs as the subjects of study and has analyzed the factors of information characteristics to study the influence of acceptance of information at home on a change in dietary life. | | | | |
|  | Participant details (eg, age/sex and number) | Age | | NA  most people were in their 40s and 50s, and respondents in their 40s or older accounted for almost half. | | |
|  |  | Sex | | Both | | |
|  |  | Final enrollment | | The valid sample size used for analysis was 220 individuals. | | |
| Details/results extracted from source of evidence | Review question addressed  (Tick) | 1. | The health-related elements in *mukbang* (Food ads, food related videos) | | NA | |
|  |  | Source text |  | | | |
|  |  | 2. | The relationship between *mukbang* (Food ads, food related videos) and health | | √ | |
|  |  | Source text | 1) The TV cooking programs had the potential effects to influence our dietary choices and improve our health.  2) TV cooking programs can be used as a tool to promote healthy eating habits and improve our health. | | | |
|  |  | 3. | Impacts may *mukbang* (Food ads, food related videos) have on eating habits or eating behaviors | | √ | |
|  |  | Source text | TV cooking programs can have a significant impact on our eating habits and can be used as a tool to promote healthy eating habits. | | | |
|  |  | 4. | The design, development, or usability of *mukbang* (Food ads, food related videos) watching interventions to demonstrate the usefulness of *mukbang* in the health field | | | NA |
|  |  | Source text | NA | | | |
|  |  | 5. | Evaluation of the health effects of various types of *mukbang* (Food ads, food related videos) | | | NA |
|  |  | Source text | NA | | | |
|  |  | 6. | The challenges and barriers of integrating *mukbang* (Food ads, food related videos) videos into clinical practice | | | NA |
|  |  | Source text | NA | | | |
|  |  | 7. | The advantages and/or disadvantages of *mukbang* (Food ads, food related videos) watching | | | √ |
|  |  | Source text | Advantages:   1. Cooking videos can provide viewers with relevant information about food. 2. Cooking videos can help us make more informed decisions about our dietary choices and promote healthier eating habits. | | | |
|  | Research method/Tools | quantitative research; using questionnaires to conduct this study. | | | | |
|  | Characteristics of *mukbang* | Cookbang: Broadcast cooking shows  The broadcast cuisine covered in this study is based on ‘food’ and is broadcast on terrestrial and comprehensive broadcasts.  In this study, it was mentioned that cooking broadcasts are broadcasts that focus on the situation of making food, unlike mukbangs. | | | | |
|  | Health-related elements | Dietary behaviors | | | | |
|  | Summary of key findings related to health | 1) The information characteristics of TV cooking programs showed a positive significant influence on the acceptance of information, and second, the acceptance of information of TV cooking programs showed a positive significant influence on the change in dietary life.  2) This study could have a significant impact on our eating habits by providing valuable insights into the influence of TV cooking programs on our dietary choices. By understanding the information characteristics that influence the acceptance of information from TV cooking programs, we can make more informed decisions about the types of programs we watch and the information we accept. This study also provides insights into the potential risks associated with using TV cooking programs as a source of dietary information and suggests ways to minimize these risks. Overall, this study can help us make more informed decisions about our dietary choices and promote healthier eating habits.  3) -The purpose of the study is to minimize the health risks that may arise when cooking is used in dietary life by verifying the professionalism and reliability of broadcast cooking information.  - The study found that the characteristics of information provided in cooking programs affect dietary changes. As a result, the study concluded that further research should be conducted on the development of cooking programs that consider nutritional and medical aspects, as well as their satisfaction and impact. | | | | |

*Food ads and food related videos must include images of celebrities eating.

47. Actual Status of Mukbang Viewing and Food Habits of University Students in Wonju Area

|  | Items | Data | | | |
| --- | --- | --- | --- | --- | --- |
| Inclusion criteria | Population/Main research object | The subjects of this study were current students at a university located in a city in Korea, 354 participants finally finished this survey. | | | |
|  | Concept (Tick)  Studies focusing on | 1. | *mukbang* watching (Food ads, food related videos*) | | √ |
|  |  | 2. | the impact of *mukbang* (Food ads, food related videos) on health | | √ |
|  |  | 3. | the relationship between *mukbang* (Food ads, food related videos) and health | | √ |
|  |  | 4. | the design, development, or usability of *mukbang* (Food ads, food related videos) watching interventions to demonstrate the usefulness of *mukbang* in the health field | | NA |
|  |  | 5. | evaluating the effectiveness of various types of *mukbang* (Food ads, food related videos) for public health | | NA |
|  |  | 6. | the challenges and barriers of integrating *mukbang* (Food ads, food related videos) videos into clinical practice | | NA |
|  |  | 7. | the advantages and/or disadvantages of *mukbang* (Food ads, food related videos) watching | | √ |
|  | Types of evidence source | Journal Paper | | | |
| Evidence source details and characteristics | Citation details (eg, author/s, date, title, journal, volume, issue, pages) | Title | | Actual Status of Mukbang Viewing and Food Habits of University Students in Wonju Area | |
|  |  | Journal | | Korean Journal of Community Living Science | |
|  |  | Author/s | | Seung-Lim Lee, Sun Hee Lee | |
|  |  | Date | | 2022.11 | |
|  | Main content of the evidence | This study investigated mukbang viewing status among Korean college students, and also assessed the association between mukbang viewing and students' eating habits. Studies have shown that substitution satisfaction is the most common reason for viewing. mukbang viewing can affect the food choices and eating habits of viewers, and the effects are mostly negative. | | | |
|  | Participant details (eg, age/sex and number) | Age | | College students; average age was 21 | |
|  |  | Sex | | Both (169 male, 185 female) | |
|  |  | Final enrollment | | A total of 354 participants finally finished the questionnaire survey. | |
| Details/results extracted from source of evidence | Review question addressed  (Tick) | 1. | The health-related elements in *mukbang* (Food ads, food related videos) | | √ |
|  |  | Source text | 1. The popular mukbang presents an intimacy and usually presents an unhealthy and excessive diet.  2. Most studies agree that mukbang provides bad eating behaviors such as binge eating, and high-calorie foods. | | |
|  |  | 2. | The relationship between *mukbang* (Food ads, food related videos) and health | | √ |
|  |  | Source text | 1. After watching mukbang, the proportion of choosing eating outside, delivery food, late-night snacks and convenience food increased significantly.  2. The more frequent mukbang viewing, the higher the probability of forming unhealthy eating habits, and may lead to overweight and obesity.  3. Studies have shown that mukbang has a great impact on appetite, but it can only produce a substitute satisfaction effect, and excessive craving for food is likely to cause binge eating.  4. The longer the mukbang watching, the higher the snack intake and the frequency of eating outside, and the corresponding increase in the consumption of junk food such as fast food. | | |
|  |  | 3. | Impacts may *mukbang* (Food ads, food related videos) have on eating habits or eating behaviors | | √ |
|  |  | Source text | People who watched mukbang videos more frequently were more likely to eat out or eat ready-to-eat meals like takeout.  In summary, university students who frequently watched eating shows showed a higher percentage of undesirable eating habits. | | |
|  |  | 4. | The design, development, or usability of *mukbang* (Food ads, food related videos) watching interventions to demonstrate the usefulness of *mukbang* in the health field | | NA |
|  |  | Source text | NA | | |
|  |  | 5. | Evaluation of the health effects of various types of *mukbang* (Food ads, food related videos) | | NA |
|  |  | Source text | NA | | |
|  |  | 6. | The challenges and barriers of integrating *mukbang* (Food ads, food related videos) videos into clinical practice | | NA |
|  |  | Source text | NA | | |
|  |  | 7. | The advantages and/or disadvantages of *mukbang* (Food ads, food related videos) watching | | √ |
|  |  | Source text | NA | | |
|  | Research method/Tools | Questionnaire survey | | | |
|  | Characteristics of *mukbang* | Mukbang published on TV, YouTube, focus on showing, introducing and eating food. | | | |
|  | Health-related elements | Eating behaviors, weight control | | | |
|  | Summary of key findings related to health | In the future, the use of mukbang to promote healthy food rich in nutrients and the insertion of nutrition education in mukbang could be considered to increase the awareness of healthy eating among adolescents. Among Korean college students who watch mukbang regularly, especially those with weak awareness of healthy eating and bad eating habits, mukbang usually has a negative impact on their eating habits. | | | |

*Food ads and food related videos must include images of celebrities eating.

48. A study on the prevalence of watching Mukbang and factors related food behaviors in adults

|  | Items | Data | | | |
| --- | --- | --- | --- | --- | --- |
| Inclusion criteria | Population/Main research object | The participants of this study were adults of all genders with mukbang viewing experience. Questionnaires were distributed mainly to adults living in Gwangju and South Jeolla Province. Finally, 506 online questionnaires and 303 offline questionnaires were collected, and 800 participants were included after screening. | | | |
|  | Concept (Tick)  Studies focusing on | 1. | *mukbang* watching (Food ads, food related videos*) | | √ |
|  |  | 2. | the impact of *mukbang* (Food ads, food related videos) on health | | √ |
|  |  | 3. | the relationship between *mukbang* (Food ads, food related videos) and health | | √ |
|  |  | 4. | the design, development, or usability of *mukbang* (Food ads, food related videos) watching interventions to demonstrate the usefulness of *mukbang* in the health field | | NA |
|  |  | 5. | evaluating the effectiveness of various types of *mukbang* (Food ads, food related videos) for public health | | NA |
|  |  | 6. | the challenges and barriers of integrating *mukbang* (Food ads, food related videos) videos into clinical practice | | NA |
|  |  | 7. | the advantages and/or disadvantages of *mukbang* (Food ads, food related videos) watching | | √ |
|  | Types of evidence source | Thesis | | | |
| Evidence source details and characteristics | Citation details (eg, author/s, date, title, journal, volume, issue, pages) | Title | | A study on the prevalence of watching Mukbang and factors related food behaviors in adults | |
|  |  | Journal | | NA | |
|  |  | Author/s | | Nam Ha yan | |
|  |  | Date | | 2020.08 | |
|  | Main content of the evidence | This study was to investigate the relationship between the reality of watching 'Mukbang' and eating habits of 800 people (including men and women) in their 20s and older who have experience in watching 'Mukbang'. The study has found that 'Mukbang' viewing time was the most correlated with body mass index (BMI), followed by order of delivery food order, weight, age, and number of family members. According to the results of these studies, there was a clear positive correlation between 'Mukbang' viewing time and body mass index (BMI). The higher the viewing time, the higher the intake of late-night snacks and delivered food, and undesirable health behaviors and eating behaviors, whereas the frequency of breakfast was lower. | | | |
|  | Participant details (eg, age/sex and number) | Age | | In their 20s and older | |
|  |  | Sex | | Both | |
|  |  | Final enrollment | | A total of 800 participants who have watched mukbang videos have joined in the study. | |
| Details/results extracted from source of evidence | Review question addressed  (Tick) | 1. | The health-related elements in *mukbang* (Food ads, food related videos) | |  |
|  |  | Source text |  | | |
|  |  | 2. | The relationship between *mukbang* (Food ads, food related videos) and health | | √ |
|  |  | Source text | 1. Viewers with fewer family members, younger age, and higher BMI watched mukbang significantly more frequently per week.  2. The longer the weekly mukbang viewing time, the more inclined the audience to choose meat food, while the frequency of eating vegetables and fruits decreased significantly, and the higher the frequency of skipping breakfast and eating late-night snacks and takeaway.  3. The longer the viewing time of mukbang, the more the viewer consumed per meal. | | |
|  |  | 3. | Impacts may *mukbang* (Food ads, food related videos) have on eating habits or eating behaviors | | √ |
|  |  | Source text | 1. Mukbang will make the viewer want to eat the food that the anchor eats in the video, changing the viewer's food choice.  2. The results of the investigation of the eating behavior of mukbang viewers showed that the consumption of fish, fruits, vegetables and legumes was significantly higher with shorter viewing time per week, while the choice of snacks or eating out, ready-to-eat or fast food was less. In addition, viewers who watched mukbang for less time per week were more likely to choose foods through their nutrition labels, and they would try to avoid carbonated drinks, overly salty foods, sweet foods, and fatty foods. | | |
|  |  | 4. | The design, development, or usability of *mukbang* (Food ads, food related videos) watching interventions to demonstrate the usefulness of *mukbang* in the health field | | NA |
|  |  | Source text | NA | | |
|  |  | 5. | Evaluation of the health effects of various types of *mukbang* (Food ads, food related videos) | | NA |
|  |  | Source text | NA | | |
|  |  | 6. | The challenges and barriers of integrating *mukbang* (Food ads, food related videos) videos into clinical practice | | NA |
|  |  | Source text | NA | | |
|  |  | 7. | The advantages and/or disadvantages of *mukbang* (Food ads, food related videos) watching | | √ |
|  |  | Source text | Disadvantages: The increase in 'Mukbang' viewing time per week as the age was lower, the number of family members was smaller, the body mass index (BMI), the frequency of ordering food, and the weight of food orders were higher. | | |
|  | Research method/Tools | Questionnaires；Random Forest | | | |
|  | Characteristics of *mukbang* | Mukbang published on TV, YouTube, focus on showing, introducing and eating food. | | | |
|  | Health-related elements | Dietary behaviors, food preference, weight control | | | |
|  | Summary of key findings related to health | 1. Weekly mukbang viewing time was associated with higher BMI, vicinal satisfaction, loneliness, and boredom. The study found that viewers mostly watch mukbang to alleviate boredom and the loneliness of not being able to share food with their families.  2. In this study, it was suggested that watching mukbang for longer and more times would increase the viewer's choice of unhealthy food and lead to poor eating behavior.  3. Attention should be paid in society to raise awareness of proper eating habits among viewers, especially viewers who rely on mukbang to relieve loneliness and obtain vicissitysatisfaction, and the authors of this study believe that adequate nutrition education is very necessary for long-term viewers of mukbang.  4. More family members, higher income levels, lower BMI, more weekly working hours and health concern levels, and lower weekly eating and drinking hours.  5. Watching food videos was associated with worse food choices and eating habits. People who watched less food videos paid more attention to food nutrition and health. | | | |

*Food ads and food related videos must include images of celebrities eating.

49. A Study on Eating Habits and Health Behavior in Their 20s and 30s According to Social Media Food Content Usage

|  | Items | Data | | | |
| --- | --- | --- | --- | --- | --- |
| Inclusion criteria | Population/Main research object | Most of the participants were in their 20s, and most were female students. Most participants used Instagram to obtain food-related contents on social media. | | | |
|  | Concept (Tick)  Studies focusing on | 1. | *mukbang* watching (Food ads, food related videos*) | | NA |
|  |  | 2. | the impact of *mukbang* (Food ads, food related videos) on health | | √ |
|  |  | 3. | the relationship between *mukbang* (Food ads, food related videos) and health | | √ |
|  |  | 4. | the design, development, or usability of *mukbang* (Food ads, food related videos) watching interventions to demonstrate the usefulness of *mukbang* in the health field | | NA |
|  |  | 5. | evaluating the effectiveness of various types of *mukbang* (Food ads, food related videos) for public health | | NA |
|  |  | 6. | the challenges and barriers of integrating *mukbang* (Food ads, food related videos) videos into clinical practice | | NA |
|  |  | 7. | the advantages and/or disadvantages of *mukbang* (Food ads, food related videos) watching | | √ |
|  | Types of evidence source | Thesis | | | |
| Evidence source details and characteristics | Citation details (eg, author/s, date, title, journal, volume, issue, pages) | Title | | A Study on Eating Habits and Health Behavior in Their 20s and 30s According to Social Media Food Content Usage | |
|  |  | Journal | | NA | |
|  |  | Author/s | | Bang Seo Yeon | |
|  |  | Date | | 2023.02 | |
|  | Main content of the evidence | This study aimed to investigate the current status of food content on social media, the extent of food content use, the purpose of food use, and satisfaction with food content, as well as the impact of food content on users' eating habits and health behaviors. The results of the study found that obtaining information about famous restaurants or well-known coffee shops was the top reason for viewing, and participants had an overall positive attitude towards the use of social media food content. As for the influence of food content on viewers on social media, the study pointed out that after using food-related information, most subjects could feel hunger, and most people would choose to eat occasionally after watching food content, and the food choice was mainly fast food, indicating that food content is more likely to lead to unhealthy food choices and eating behaviors. In addition, the results of this study show that most people use food related content on social media to relieve daily stress and are more inclined to eat when they are feeling depressed. | | | |
|  | Participant details (eg, age/sex and number) | Age | | Aged between 20~30 years old | |
|  |  | Sex | | Both | |
|  |  | Final enrollment | | A total of 452 participants joined in this study. | |
| Details/results extracted from source of evidence | Review question addressed  (Tick) | 1. | The health-related elements in *mukbang* (Food ads, food related videos) | | NA |
|  |  | Source text | NA | | |
|  |  | 2. | The relationship between *mukbang* (Food ads, food related videos) and health | | √ |
|  |  | Source text | 1. The study showed that 81.6% of participants felt hungry after using food content; 71.5 percent said food content on social media would attract them to eat, with fast food being the most popular choice.  2. When watching mukbang and other food content, there may be an unplanned impulse to eat, which virtually increases the amount of food consumed by the viewer, leading to excessive eating over consumption.  3. Although the study did not specifically look at the relationship between mukbang and BMI, in combination with other related studies, prolonged use of food content increases the risk of overweight or obesity among viewers. | | |
|  |  | 3. | Impacts may *mukbang* (Food ads, food related videos) have on eating habits or eating behaviors | | √ |
|  |  | Source text | 1. Food content on social media is similar to mukbang. In most cases, convenience store food or convenience food is used to replace meals, and late-night snacks are also more common, which have been proved to have a negative impact on the eating habits of viewers, leading to excessive eating.  2. The study found that using food content on social media achieved similar effects as watching mukbang, with the shorter duration of use having less impact on users' eating habits and the more desirable their eating habits. | | |
|  |  | 4. | The design, development, or usability of *mukbang* (Food ads, food related videos) watching interventions to demonstrate the usefulness of *mukbang* in the health field | | NA |
|  |  | Source text | NA | | |
|  |  | 5. | Evaluation of the health effects of various types of *mukbang* (Food ads, food related videos) | | NA |
|  |  | Source text | NA | | |
|  |  | 6. | The challenges and barriers of integrating *mukbang* (Food ads, food related videos) videos into clinical practice | | NA |
|  |  | Source text | NA | | |
|  |  | 7. | The advantages and/or disadvantages of *mukbang* (Food ads, food related videos) watching | | √ |
|  |  | Source text |  | | |
|  | Research method/Tools | Online questionnaire survey. | | | |
|  | Characteristics of *mukbang* | Mukbang usually contains high calorie foods and slim hosts who can make huge food intake at one time. In this study, mukbang were considered as unhealthy food content. | | | |
|  | Health-related elements | Eating habits.  Health-related behaviors, which contains the level of effort to maintain good health, check nutrition facts tables, hand washing, exercise, subjective health, and alcohol consumption. | | | |
|  | Summary of key findings related to health | 1. The study showed that participants followed food content on social media mainly to get information about restaurants or famous cafes.  2. The main variable in this study was food content on social media, also known as ‘mukstragram’. Research suggested that consuming food content on social media had similar effects to watching mukbang, activating the brain's appetite and reward centers, inducing real hunger and increasing appetite.  3. Young women are significantly more influenced by food content on Instagram than men of the same age or older.  4. Negative emotions may affect users' eating habits through food content on social media, leading not only to excessive use of social media, but also to bad eating habits. Therefore, the impact of negative emotions such as stress and anxiety should be taken into account in healthy eating education, and users should be guided through health education on how to cope with negative emotions together, rather than escape through overeating.  5. Food content in social media is dominated by medium and low-calorie healthy foods, which is complete opposite to mukbang, cause mukbang attracts viewers with high calorie foods and huge food intake. | | | |

*Food ads and food related videos must include images of celebrities eating.

50. Mukbang viewing status and eating behavior of some elementary school students in Ulsan

|  | Items | Data | | | |
| --- | --- | --- | --- | --- | --- |
| Inclusion criteria | Population/Main research object | This study was conducted among fifth to sixth grade students from five elementary schools in a city in Korea. 318 participants aged between 10~12 years old were joined in this study. | | | |
|  | Concept (Tick)  Studies focusing on | 1. | *mukbang* watching (Food ads, food related videos*) | | √ |
|  |  | 2. | the impact of *mukbang* (Food ads, food related videos) on health | | √ |
|  |  | 3. | the relationship between *mukbang* (Food ads, food related videos) and health | | √ |
|  |  | 4. | the design, development, or usability of *mukbang* (Food ads, food related videos) watching interventions to demonstrate the usefulness of *mukbang* in the health field | | NA |
|  |  | 5. | evaluating the effectiveness of various types of *mukbang* (Food ads, food related videos) for public health | | NA |
|  |  | 6. | the challenges and barriers of integrating *mukbang* (Food ads, food related videos) videos into clinical practice | | NA |
|  |  | 7. | the advantages and/or disadvantages of *mukbang* (Food ads, food related videos) watching | | √ |
|  | Types of evidence source | Thesis | | | |
| Evidence source details and characteristics | Citation details (eg, author/s, date, title, journal, volume, issue, pages) | Title | | Mukbang viewing status and eating behavior of some elementary school students in Ulsan | |
|  |  | Journal | | NA | |
|  |  | Author/s | | Dayoon Kang | |
|  |  | Date | | 2022.06 | |
|  | Main content of the evidence | This study was intended to identify the current conditions of elementary school students’ Mukbang-watching and its influence on their negative eating habits and obesity. According to the results of this study, the higher the number of Mukbang-watching, the more greatly Mukbang had an effect on the viewers’ actual eating behaviors, such as getting the food shown in Mukbang. In addition, many of the food consumed by them after watching Mukbang was fast food and instant food, which shows that Mukbang-watching leads to the viewers’ intake of obesity-causing food. In addition, the students who often watched Mukbang had lower scores on the positive eating habits but higher scores on the negative habits, which demonstrated that frequent Mukbang watching is closely related to undesirable eating habits. | | | |
|  | Participant details (eg, age/sex and number) | Age | | Students in grades 5 and 6 | |
|  |  | Sex | | Both (143male students，175female students) | |
|  |  | Final enrollment | | A total of 318 participants have completed this survey. | |
| Details/results extracted from source of evidence | Review question addressed  (Tick) | 1. | The health-related elements in *mukbang* (Food ads, food related videos) | | √ |
|  |  | Source text | Mukbang always contains irritating and high-calorie foods. | | |
|  |  | 2. | The relationship between *mukbang* (Food ads, food related videos) and health | | √ |
|  |  | Source text | 1. According to 61.2% of the participants in this study, the ornate, large amount of food in mukbang was related to appetite and hunger, it led to excessive energy intake and contributes to obesity. 2. Strong stimulating and high-calorie foods were more likely to arouses the appetite of the viewers, making it more inclined to eat foods with high sugar or extreme spiciness, leading to unhealthy eating habits. 3. About 78% of the respondents reported that they felt significantly hungry after watching mukbang. The more times they watched mukbang, the longer they watched it, the more likely they felt hungry. 4. Mukbang watching resulted in a high proportion of actual eating behaviors, which could easily lead to binge eating. 5. Watching mukbang more frequently and for a much long time might make it easier to consume unhealthy fast food, snacks and other foods with single nutrition or low nutritional value, and the intake of healthy foods such as fruits, vegetables, and milk was also significantly reduced. Such inappropriate eating behaviors and eating habits may contribute to the increased prevalence of childhood obesity in Korea. 6. The study has found that the more viewers watched mukbang, the more irregular the diet was. Adolescents with the ability to buy would be more likely to choose foods presented in mukbang regardless of health. | | |
|  |  | 3. | Impacts may *mukbang* (Food ads, food related videos) have on eating habits or eating behaviors | | √ |
|  |  | Source text | In particular, mukbang might have an impact on the eating habits of adolescents, who are less experienced in identifying online information and can easily imitate the eating behaviors displayed in mukbang. | | |
|  |  | 4. | The design, development, or usability of *mukbang* (Food ads, food related videos) watching interventions to demonstrate the usefulness of *mukbang* in the health field | | NA |
|  |  | Source text | NA | | |
|  |  | 5. | Evaluation of the health effects of various types of *mukbang* (Food ads, food related videos) | | NA |
|  |  | Source text | NA | | |
|  |  | 6. | The challenges and barriers of integrating *mukbang* (Food ads, food related videos) videos into clinical practice | | NA |
|  |  | Source text | NA | | |
|  |  | 7. | The advantages and/or disadvantages of *mukbang* (Food ads, food related videos) watching | | √ |
|  |  | Source text | Advantages: improve appetite, release the loneliness when eating alone.  Disadvantages: develop bad eating habit, increase the times of eating snacks and midnight snacks. | | |
|  | Research method/Tools | Questionnaire survey | | | |
|  | Characteristics of *mukbang* | NA | | | |
|  | Health-related elements | NA | | | |
|  | Summary of key findings related to health | 1. It demonstrated that the higher the number of Mukbang-watching, the more appetite increased after watching Mukbang. 2. People who watched mukbang more often reported that it helped them control their weight, increased their appetite, and significantly increased their frequency of snacking. At the same time, watching mukbang videos alone was thought to be more interesting than spending time or eating together with friends or family, and the viewers were more willing to try the food or restaurant presented in mukbang videos. 3. People who watched mukbang more often were more likely to choose unhealthy foods such as fast food, delivery food, drinks and snacks. They were more likely to eat sweet or heavy foods and had worse eating habits than those who watched mukbang occasionally. 4. Frequent viewing of mukbang videos increases the risk of becoming overweight or obese. 5. People who watched mukbang more frequently were more likely to feel hungry after watching and to consume more food. Meanwhile, the food they consumed were mainly unhealthy foods such as fast food and ramen, and their intake of milk, vegetables and fruits was significantly lower than those who watched mukbang videos infrequently. | | | |

*Food ads and food related videos must include images of celebrities eating.

51. Study on emotional and physiological changes according to food content types and scenes - ASMR Mukbang VS Normal Mukbang -

|  | Items | Data | | | |
| --- | --- | --- | --- | --- | --- |
| Inclusion criteria | Population/Main research object | This study was conducted on people in their 20s or older who have experience watching mukbang broadcasts in Seoul and Gyeonggi Province. The conditions for participation were that the participant had experience watching mukbang broadcasts and was right-handed to select subjects who showed the same mechanism when measuring EEG. | | | |
|  | Concept (Tick)  Studies focusing on | 1. | *mukbang* watching (Food ads, food related videos*) | | √ |
|  |  | 2. | the impact of *mukbang* (Food ads, food related videos) on health | | √ |
|  |  | 3. | the relationship between *mukbang* (Food ads, food related videos) and health | | √ |
|  |  | 4. | the design, development, or usability of *mukbang* (Food ads, food related videos) watching interventions to demonstrate the usefulness of *mukbang* in the health field | | NA |
|  |  | 5. | evaluating the effectiveness of various types of *mukbang* (Food ads, food related videos) for public health | | √ |
|  |  | 6. | the challenges and barriers of integrating *mukbang* (Food ads, food related videos) videos into clinical practice | | NA |
|  |  | 7. | the advantages and/or disadvantages of *mukbang* (Food ads, food related videos) watching | | √ |
|  | Types of evidence source | Journal paper  An experimental design | | | |
| Evidence source details and characteristics | Citation details (eg, author/s, date, title, journal, volume, issue, pages) | Title | | Study on emotional and physiological changes according to food content types and scenes - ASMR Mukbang VS Normal Mukbang - | |
|  |  | Journal | | The Journal of Image and Cultural Contents | |
|  |  | Author/s | | Yejin Han | |
|  |  | Date | | 2022-06 | |
|  | Main content of the evidence | This study examined viewers' emotional and physiological responses when watching regular mukbangs and mukbangs with ASMR added. It is predicted that there will be differences, and based on the discussion so far, the following proposes a research question. This study investigated the differences between before and after watching a mukbang video and the same video or sound. Aiming to determine physical and physiological differences, and physiological differences of individuals according to the development of content over time. | | | |
|  | Participant details (eg, age/sex and number) | Age | | The age of the participants ranges from 23 to 40. | |
|  |  | Sex | | Both | |
|  |  | Final enrollment | | There were 28 subjects who participated in this experiment, and 14 each were randomly assigned to a regular mukbang viewing group and an ASMR mukbang viewing group. | |
| Details/results extracted from source of evidence | Review question addressed  (Tick) | 1. | The health-related elements in *mukbang* (Food ads, food related videos) | | √ |
|  |  | Source text | The emotion of pleasure provided by mukbang appears to be related to the pleasure through the experience of excessive arousal rather than the pleasure of experiencing relaxation. | | |
|  |  | 2. | The relationship between *mukbang* (Food ads, food related videos) and health | | √ |
|  |  | Source text | ASMR videos are generally watched to rest the brain and feel stable and comfortable. | | |
|  |  | 3. | Impacts may *mukbang* (Food ads, food related videos) have on eating habits or eating behaviors | | √ |
|  |  | Source text | 1) Impacts may *mukbang* (Food ads, food related videos) have on eating habits or eating behaviors.  2) The excessively increasing viewing activity of food content videos can change the audience's perception. | | |
|  |  | 4. | The design, development, or usability of *mukbang* (Food ads, food related videos) watching interventions to demonstrate the usefulness of *mukbang* in the health field | | NA |
|  |  | Source text | NA | | |
|  |  | 5. | Evaluation of the health effects of various types of *mukbang* (Food ads, food related videos) | | √ |
|  |  | Source text | The purpose of ASMR content is to experience stimulation through specific sounds and to experience pleasant psychological stability. | | |
|  |  | 6. | The challenges and barriers of integrating *mukbang* (Food ads, food related videos) videos into clinical practice | | √ |
|  |  | Source text | The excessively increasing viewing activity of food content videos can change the audience's perception. | | |
|  |  | 7. | The advantages and/or disadvantages of *mukbang* (Food ads, food related videos) watching | | √ |
|  |  | Source text | Advantages: Gain the happiness and enjoyment (The 'sense of happiness' increases just by watching people eat, as it is said to be experienced when watching content.). Mukbang and ASMR content are new ways to experience pleasure. | | |
|  | Research method/Tools | An experimental design: using mukbang and ASMR mukbang videos, test the participants’ HRV and EEG.  In conjunction with questionnaire survey | | | |
|  | Characteristics of *mukbang* | In the early days of mukbang content, mukbangs focused on ‘how to eat,’ focusing on the amount and speed of eating. However, as Mukbang evolved into a specialized channel, its content became increasingly more advanced. | | | |
|  | Health-related elements | Emotions, dietary behaviors | | | |
|  | Summary of key findings related to health | 1) It can be inferred that the audio-visual video stimulation of the mukbang may have provided an excessive arousal response of the autonomic nervous system.  2) From the results of this study, sensory stimulation is perceived as more important than auditory stimulation.  3) ASMR mukbang videos were perceived as more meaningful visual stimulation than regular mukbang videos.  4) Mukbang and ASMR content are a new cultural form for experiencing pleasure.  5) As a result of this study, the emotion of pleasure provided by mukbang appears to be related to the pleasure through the experience of excessive arousal rather than the pleasure of experiencing relaxation. | | | |

*Food ads and food related videos must include images of celebrities eating.

52. Does Mukbang Watching Really Affect Obesity? Focusing on the Factors Related to Health and Mukbang Watching

|  | Items | Data | | | |
| --- | --- | --- | --- | --- | --- |
| Inclusion criteria | Population/Main research object | A total of 668 people joined this survey. Of the respondents, 252 (37.7 percent) were obese and 416 (62.2 percent) were non-obese. | | | |
|  | Concept (Tick)  Studies focusing on | 1. | *mukbang* watching (Food ads, food related videos*) | | √ |
|  |  | 2. | the impact of *mukbang* (Food ads, food related videos) on health | | √ |
|  |  | 3. | the relationship between *mukbang* (Food ads, food related videos) and health | | √ |
|  |  | 4. | the design, development, or usability of *mukbang* (Food ads, food related videos) watching interventions to demonstrate the usefulness of *mukbang* in the health field | | √ |
|  |  | 5. | evaluating the effectiveness of various types of *mukbang* (Food ads, food related videos) for public health | | NA |
|  |  | 6. | the challenges and barriers of integrating *mukbang* (Food ads, food related videos) videos into clinical practice | | NA |
|  |  | 7. | the advantages and/or disadvantages of *mukbang* (Food ads, food related videos) watching | | √ |
|  | Types of evidence source | Cross-sectional study | | | |
| Evidence source details and characteristics | Citation details (eg, author/s, date, title, journal, volume, issue, pages) | Title | | Does Mukbang Watching Really Affect Obesity? Focusing on the Factors Related to Health and Mukbang Watching | |
|  |  | Journal | | Korean Journalism Journal | |
|  |  | Author/s | | Sunwook Yoo, Giha Shin, Soojin Kim | |
|  |  | Date | | 2021.04 | |
|  | Main content of the evidence | As mukbang watching could make viewers feel vicarious satisfaction and trigger cravings. By analyzing and conducting cognitive surveys on mukbang content and mukbang-related articles, previous studies have confirmed that the more provocative content Youtube mukbang contains, such as binge eating, the more popular the content is. Thus, this study attempted to explore the degree of viewing and satisfaction with viewing, and whether the imitative eating behavior after viewing is the result of obesity, and its causal relationship.  The results showed that health awareness affected the degree of obesity, and the higher the health awareness, the lower the degree of obesity. Secondly, people with more general health information had lower obesity, and the more people sought information about obesity, the higher their obesity. Thirdly, the more time and frequency of watching the broadcast, the higher the degree of obesity. | | | |
|  | Participant details (eg, age/sex and number) | Age | | The average age was 47.73 years old; the largest number was in the 50-59 age group. The participants were all above 20 years old. | |
|  |  | Sex | | 325 male (48.7%) and 343 female (51.3%) | |
|  |  | Final enrollment | | A total of 668 people joined this survey. | |
| Details/results extracted from source of evidence | Review question addressed  (Tick) | 1. | The health-related elements in *mukbang* (Food ads, food related videos) | | √ |
|  |  | Source text | Mukbang should be able to change the way people think about the possibility of obesity in food. | | |
|  |  | 2. | The relationship between *mukbang* (Food ads, food related videos) and health | | √ |
|  |  | Source text | 1) Obesity was more likely to be observed with more time and more frequent watching of Mukbang, but the influence of imitative eating behavioral intention after watching Mukbang on obesity was not significant.  2) Many scholars believed that mukbang can promote obesity.  3) The degree of mukbang watching and satisfaction of mukbang watching had an effect on obesity.  4) The more time and frequency of watching the broadcast, the higher the degree of obesity. | | |
|  |  | 3. | Impacts may *mukbang* (Food ads, food related videos) have on eating habits or eating behaviors | | √ |
|  |  | Source text | 1) Mukbang viewing promotes food cravings and intake, suggesting that mukbang viewing as a media use behavior is associated with satisfying viewing and mimicking eating behaviors.  2) This study has found that the imitation behavior of mukbang had no effect on obesity.  3) If viewers were not satisfied with the food, you will not imitate the behavior.  4) The more satisfied the viewers were with mukbang watching, the more likely they were to imitate, especially for people who have been identified as obese. | | |
|  |  | 4. | The design, development, or usability of *mukbang* (Food ads, food related videos) watching interventions to demonstrate the usefulness of *mukbang* in the health field | | √ |
|  |  | Source text | Future application:  1) Mukbang should be used as a preventive and educational factor rather than an inducement of obesity. The content planning should include elements of interest to the recipients that can improve their satisfaction with mukbang programs or content, including information about healthy ingredients in the diet and health precautions, which is also an effective way to use mukbang as an educational factor.  2) If the narrative approach is applied to mukbang, realizing narrative in mukbang can be an effective way to naturally guide diet and ideal attitudes or behaviors such as diet and health. | | |
|  |  | 5. | Evaluation of the health effects of various types of *mukbang* (Food ads, food related videos) | | NA |
|  |  | Source text | NA | | |
|  |  | 6. | The challenges and barriers of integrating *mukbang* (Food ads, food related videos) videos into clinical practice | | NA |
|  |  | Source text | NA | | |
|  |  | 7. | The advantages and/or disadvantages of *mukbang* (Food ads, food related videos) watching | | √ |
|  |  | Source text | Mukbang may not be harmful to viewers’ body shape: In reality, enjoying mukbang content and actually imitating behavior are different dimensions. | | |
|  | Research method/Tools | Questionnaire survey | | | |
|  | Characteristics of *mukbang* | Mukbang now broadly refers to food-themed TV programs, Internet, social media, and YouTube broadcasts, and includes content about eating and cooking. | | | |
|  | Health-related elements | Binge eating, obesity | | | |
|  | Summary of key findings related to health | 1) More specifically, this study has drawn a strategic plan for raising health consciousness and promoting health information behavior for solving the obesity problem and practical implications for the use of Mukbang was presented.  2) What mukbang watching really affects is eating behavior, mukbang watching can not directly cause obesity. Behaviors such as eating snacks and late-night snacks during watching may be the cause of obesity.  3) Future application: Mukbang should be used as a preventive and educational factor rather than an inducement of obesity. The content planning should include elements of interest to the recipients that can improve their satisfaction with mukbang programs or content, including information about healthy ingredients in the diet and health precautions, which is also an effective way to use mukbang as an educational factor.  4) Future application: If the narrative approach is applied to mukbang, realizing narrative in mukbang can be an effective way to naturally guide diet and ideal attitudes or behaviors such as diet and health. | | | |

*Food ads and food related videos must include images of celebrities eating.

53. A Study of Viewers' Comments on Online Mukbang Videos: A Big-Data Analysis of Perceptions toward Eating Behavior

|  | Items | Data | | | |
| --- | --- | --- | --- | --- | --- |
| Inclusion criteria | Population/Main research object | A total of 72,721 viewer comments on 36 popular YouTube Mukbang videos uploaded between July 2018 and June 2019 were collected and analyzed. | | | |
|  | Concept (Tick)  Studies focusing on | 1. | *mukbang* watching (Food ads, food related videos*) | | √ |
|  |  | 2. | the impact of *mukbang* (Food ads, food related videos) on health | | √ |
|  |  | 3. | the relationship between *mukbang* (Food ads, food related videos) and health | | √ |
|  |  | 4. | the design, development, or usability of *mukbang* (Food ads, food related videos) watching interventions to demonstrate the usefulness of *mukbang* in the health field | | NA |
|  |  | 5. | evaluating the effectiveness of various types of *mukbang* (Food ads, food related videos) for public health | | NA |
|  |  | 6. | the challenges and barriers of integrating *mukbang* (Food ads, food related videos) videos into clinical practice | | NA |
|  |  | 7. | the advantages and/or disadvantages of *mukbang* (Food ads, food related videos) watching | | NA |
|  | Types of evidence source | Qualitative research: textual analysis | | | |
| Evidence source details and characteristics | Citation details (eg, author/s, date, title, journal, volume, issue, pages) | Title | | A Study of Viewers' Comments on Online Mukbang Videos: A Big-Data Analysis of Perceptions toward Eating Behavior | |
|  |  | Journal | | Korean Journal of Journalism & Communication Studies | |
|  |  | Author/s | | Soontae An, Yujin Lim, Hannah Lee | |
|  |  | Date | | 2020.04 | |
|  | Main content of the evidence | The purpose of this study was to examine viewers' comments on how popular online eating show (Mukbang) videos influence viewers' perceptions and lead to unhealthy eating behaviors. | | | |
|  | Participant details (eg, age/sex and number) | Age | | NA | |
|  |  | Sex | | NA | |
|  |  | Final enrollment | | A total of 72,721 viewer comments on 36 popular YouTube Mukbang videos were collected and analyzed. | |
| Details/results extracted from source of evidence | Review question addressed  (Tick) | 1. | The health-related elements in *mukbang* (Food ads, food related videos) | |  |
|  |  | Source text | The study revealed that the majority of mukbang videos contained unhealthy eating behaviors, such as eating too fast or eating more than one serving at a time. | | |
|  |  | 2. | The relationship between *mukbang* (Food ads, food related videos) and health | | √ |
|  |  | Source text | 1. In the case of exposure to the contents of high-calorie, spicy foods, Food &gt; &lt; I want to eat. The frequency of simultaneous occurrences is high. This suggested that mukbang videos may predispose viewers to unhealthy food choices that could affect their physical health. 2. The relatively healthy food in the Mukbang video is more likely to arouse viewers' appetite and interest in food. 3. It could be summarized as that Mukbang content including healthy eating behavior is more popular with viewers. 4. Viewers tend to feel sorry for YouTube creators who overeat during live broadcasts, rather than wanting to eat when watching eating content that displays unhealthy eating behaviors. This proves that most mukbang viewers have good health awareness, and they can accurately identify unhealthy content. | | |
|  |  | 3. | Impacts may *mukbang* (Food ads, food related videos) have on eating habits or eating behaviors | | √ |
|  |  | Source text | 1. It could be assumed that popular YouTubers' food decisions and eating behavior scenes could influence viewers' eating behavior. 2. Viewers of mukbang videos that contain high-calorie or spicy foods are more likely to feel hungry, and both appetite and food intake may increase. Watching mukbang content without high-calorie or spicy food is relatively easy to generate vicarious satisfaction. 3. Differences in favorability and eating behavior responses were determined by exposure to high-calorie and spicy foods. 4. The results of this study suggested that the unhealthy eating behaviors observed in most eating broadcasts do not stimulate viewers' eating behaviors, and instead, exposure to common sense and eating behaviors in daily life may be highly correlated with stimulating viewers' eating behaviors. | | |
|  |  | 4. | The design, development, or usability of *mukbang* (Food ads, food related videos) watching interventions to demonstrate the usefulness of *mukbang* in the health field | |  |
|  |  | Source text |  | | |
|  |  | 5. | Evaluation of the health effects of various types of *mukbang* (Food ads, food related videos) | |  |
|  |  | Source text | 1. Through these results, it can be seen that content revealing unhealthy eating behaviors is rated as disliked and uncomfortable by viewers, and overly reckless eating behaviors will also cause viewers' disgust and discomfort. 2. Mukbang videos containing unhealthy eating behaviors had a greater impact on viewers, with significantly more expressions of 'I want to try that' and 'I don't want to try it' about the food in the videos. 3. Considering the negative behaviors expressed by viewers when unhealthy eating behaviors are exposed, it is possible to lead to unhealthy perceptions of abnormal eating habits or eating habits. This can be explained by the fact that eating content that stimulates unhealthy eating behaviors actually leads to negative reactions from viewers, while eating content that features healthy eating behaviors is gaining more viewers' favor and sympathy. | | |
|  |  | 6. | The challenges and barriers of integrating *mukbang* (Food ads, food related videos) videos into clinical practice | | NA |
|  |  | Source text |  | | |
|  |  | 7. | The advantages and/or disadvantages of *mukbang* (Food ads, food related videos) watching | | NA |
|  |  | Source text |  | | |
|  | Research method/Tools | Big data analytics and text analytics | | | |
|  | Characteristics of *mukbang* | The content includes unhealthy or healthy eating behaviors. | | | |
|  | Health-related elements | Unhealthy and/or healthy eating behaviors | | | |
|  | Summary of key findings related to health | 1. The most common comments on mukbang videos by the viewers are words such as <food>, <sounds delicious>, <good>, <me too>, <cute>, and <I want to eat>. 2. Opinions about mukbang content are likely to be primarily divided into “food-related attitudes” and “attitudes toward YouTube creators.” 3. According to the most common comments, mukbang can arouse viewers' appetite and interest in the food in the video, and viewers often think that the food in the video 'looks delicious'. 4. Comments involving food often involve viewers having a strong desire for food, especially the food presented in the video. 5. "Please eat it" comments were more frequent when exposed to high-calorie, spicy food, while "looks delicious" comments were more common when the food was not exposed to high-calorie, spicy food. 6. At the same time, there were a lot of negative comments on content that exposed high-calorie, spicy foods, such as "I'm not jealous" and "This must be difficult for youtubers." 7. Scenes of mukbang videos containing healthy foods or healthy eating behaviors were highly correlated with viewers' appetites and willingness to eat, and therefore more likely to influence viewers' eating behaviors and food choices. Scenes of unhealthy foods or eating behaviors, on the other hand, were related to food or youtubers, showing viewers' negative responses to unhealthy content, as well as concerns and positive comments about the anchors. 8. The conclusions of this study suggest that exposure to media promoted food or eating behavior scenarios can have a negative impact on unhealthy eating habits. | | | |

*Food ads and food related videos must include images of celebrities eating.
