# Supplementary material for: Use of Mukbang in Health Promotion: Scoping Review
Source: J Med Internet Res. 2025 Mar 27;27:e56147. doi: 10.2196/56147 (PMC11986381; doi:10.2196/56147)
Supplement: Multimedia Appendix 5 [file jmir_v27i1e56147_app5.zip › Multimedia Appendix 5. Quality evaluation of part of the included articles/[64] Research on the Relieving Effect of ASMR Chewing Sounds on Anxiety in Food Video.docx]

JBI Critical Appraisal Checklist for
systematic reviews and research syntheses

**Research on the Relieving Effect of ASMR Chewing Sounds on Anxiety in Food Video**

Reviewer __X.W. and Y.X.X._________________ Date___2024.06.26_________

Author_Zhou J_

Year__2022__ Record Number___64____

|  | Yes | No | Unclear | Not applicable |
| --- | --- | --- | --- | --- |
| 1. Is the review question clearly and explicitly stated? | ☑ | □ | □ | □ |
| 1. Were the inclusion criteria appropriate for the review question? | □ | □ | ☑ | □ |
| 1. Was the search strategy appropriate? | ☑ | □ | □ | □ |
| 1. Were the sources and resources used to search for studies adequate? | ☑ | □ | □ | □ |
| 1. Were the criteria for appraising studies appropriate? | □ | □ | □ | ☑ |
| 1. Was critical appraisal conducted by two or more reviewers independently? | □ | □ | □ | ☑ |
| 1. Were there methods to minimize errors in data extraction? | ☑ | □ | □ | □ |
| 1. Were the methods used to combine studies appropriate? | ☑ | □ | □ | □ |
| 1. Was the likelihood of publication bias assessed? | □ | □ | □ | ☑ |
| 1. Were recommendations for policy and/or practice supported by the reported data? | ☑ | □ | □ | □ |
| 1. Were the specific directives for new research appropriate? | ☑ | □ | □ | □ |

Overall appraisal: Include ☑ Exclude □ Seek further info □

Comments (Including reason for exclusion)

As_JBI has not yet established specific evaluation criteria for scoping reviews, so we use evaluation indicators of systematic reviews for evaluation.
