# Supplementary material for: Use of Mukbang in Health Promotion: Scoping Review
Source: J Med Internet Res. 2025 Mar 27;27:e56147. doi: 10.2196/56147 (PMC11986381; doi:10.2196/56147)
Supplement: Multimedia Appendix 5 [file jmir_v27i1e56147_app5.zip › Multimedia Appendix 5. Quality evaluation of part of the included articles/[60] Watching a remote-video confederate eating facilitates perceived taste and consumption of food.docx]

| **RoB Assessor: X.W. and Y.X.X.** | | **Date of Appraisal: 2024.06.26** | | **Record Number: 60** | | | | |
| --- | --- | --- | --- | --- | --- | --- | --- | --- |
| **Study Author:** Nobuyuki Kawai | | **Study Title:** Watching a remote-video confederate eating facilitates perceived taste and consumption of food | | **Study Year: 2021** | | | | |
|  | |  | |  | | | | |
| **Internal Validity** | | | **Choice - Comments/Justification** | | **Yes** | **No** | **Unclear** | **N/A** |
| **Bias related to temporal precedence** | | | | | | | | |
| **1** | **Is it clear in the study what is the “cause” and what is the “effect” (i.e. there is no confusion about which variable comes first)?** | | watching a silent video of others eating amplified preferences for food and food intake | |  |  |  |  |
| **Bias related to selection and allocation** | | | | | | | | |
| **2** | **Was there a control group?** | |  | |  |  |  |  |
| **Bias related to confounding factors** | | | | | | | | |
| **3** | **Were participants included in any comparisons similar?** | | All participants were naïve to the experiment ’s purpose They had good health, no food allergies, no history of eating disorders, no special dietary restrictions, and reported being within normal weight. | |  |  |  |  |
| **Bias related to administration of intervention/exposure** | | | | | | | | |
| **4** | **Were the participants included in any comparisons receiving similar treatment/care, other than the exposure or intervention of interest?** | |  | |  |  |  |  |

| **Bias related to assessment, detection and measurement of the outcome** | | | | | | |
| --- | --- | --- | --- | --- | --- | --- |
| **5** | **Were there multiple measurements of the outcome, both pre and post the intervention/exposure?** |  | **Yes** | **No** | **Unclear** | **N/A** |
|  | **Outcome 1** | A short questionnaire containing eight questions on a 6-point scale (from “not at all ” to “extremely”) reflected participants’ subjective evaluations of the popcorn |  |  |  |  |
|  | **Outcome 2** | The consumption ratio of each bowlof popcorn |  |  |  |  |
|  |  |  |  |  |  |  |
| **6** | **Were the outcomes of participants included in any comparisons measured in the same way?** |  | **Yes** | **No** | **Unclear** | **N/A** |
|  | **Outcome 1** | A short questionnaire containing eight questions on a 6-point scale (from “not at all ” to “extremely”) reflected participants’ subjective evaluations of the popcorn |  |  |  |  |
|  | **Outcome 2** | The consumption ratio of each bowlof popcorn |  |  |  |  |
|  |  |  |  |  |  |  |
| **7** | **Were outcomes measured in a reliable way?** |  | **Yes** | **No** | **Unclear** | **N/A** |
|  | **Outcome 1** | A short questionnaire containing eight questions on a 6-point scale (from “not at all ” to “extremely”) reflected participants’ subjective evaluations of the popcorn |  |  |  |  |
|  | **Outcome 2** | The consumption ratio of each bowlof popcorn |  |  |  |  |

| **Bias related to participant retention** | | | | | | | | | | | | | |
| --- | --- | --- | --- | --- | --- | --- | --- | --- | --- | --- | --- | --- | --- |
| **8** | **Was follow-up complete and if not, were differences between groups in terms of their follow-up adequately described and analyzed?** | | | | | |  | |  | | | | |
|  | **Outcome 1** | | | | | | No follow-up and control group | | **Yes** | **No** | | **Unclear** | **N/A** |
|  |  | Result 1 | | | | |  | |  |  |  | |  |
|  |  | Result 2 | | | | |  | |  |  |  | |  |
|  |  | Result 3 | | | | |  | |  |  |  | |  |
|  | **Outcome 2** | | | | | | No follow-up and control group | | **Yes** | **No** | **Unclear** | | **N/A** |
|  |  | Result 1 | | | | |  | |  |  |  | |  |
|  |  | Result 2 | | | | |  | |  |  |  | |  |
|  |  | Result 3 | | | | |  | |  |  |  | |  |
|  |  |  | | | | | | |  |  |  | |  |
|  | **Statistical Conclusion Validity** | | | | | | | |  |  |  | |  |
| **9** | **Was appropriate statistical analysis used?** | | | | | | |  |  | | | | |
|  | **Outcome 1** | | |  | | | |  | **Yes** | **No** | **Unclear** | | **N/A** |
|  |  | Result 1 | | | | | | Descriptive analysis |  |  |  | |  |
|  |  | Result 2 | | | | | | A oneway ANOVA and Post hoc analysis |  |  |  | |  |
|  | **Outcome 2** | | |  | | | |  | **Yes** | **No** | **Unclear** | | **N/A** |
|  |  | Result 1 | | | | | | A oneway ANOVA and Post hoc analysis |  |  |  | |  |
|  | | | | | | | | | | | | | |
| **Overall appraisal:** | | | **Include:** | | **Exclude:** | **Seek Further Info:** | | | | | | | |
| **Comments:** | | | | | | | | | | | | | |
