# Supplementary material for: Use of Mukbang in Health Promotion: Scoping Review
Source: J Med Internet Res. 2025 Mar 27;27:e56147. doi: 10.2196/56147 (PMC11986381; doi:10.2196/56147)
Supplement: Multimedia Appendix 5 [file jmir_v27i1e56147_app5.zip › Multimedia Appendix 5. Quality evaluation of part of the included articles/[54] A study on the use of YouTube food content and the actual consumption of delivery food by university students in Gwangju.docx]

JBI Critical Appraisal Checklist for
analytical cross sectional studies

A study on the use of YouTube food content and the actual consumption of delivery food by university students in Gwangju_评价

Reviewer _X.W. and Y.X.X.______________________ Date___2024.06.28____________

Author_Somi Park______________________________ Year__2022__ Record Number___54____

|  | Yes | No | Unclear | Not applicable |
| --- | --- | --- | --- | --- |
| 1. Were the criteria for inclusion in the sample clearly defined? | □ | □ | ☑ | □ |
| 1. Were the study subjects and the setting described in detail? | ☑ | □ | □ | □ |
| 1. Was the exposure measured in a valid and reliable way? | ☑ | □ | □ | □ |
| 1. Were objective, standard criteria used for measurement of the condition? | □ | ☑ | □ | □ |
| 1. Were confounding factors identified? | □ | ☑ | □ | □ |
| 1. Were strategies to deal with confounding factors stated? | ☑ | □ | □ | □ |
| 1. Were the outcomes measured in a valid and reliable way? | ☑ | □ | □ | □ |
| 1. Was appropriate statistical analysis used? | ☑ | □ | □ | □ |

Overall appraisal: Include □ Exclude □ Seek further info ☑

Comments (Including reason for exclusion)

________________________________________________________________________________________________________________________________________________________________________________________________________________________________________________________________________________________________
