# Supplementary material for: Use of Mukbang in Health Promotion: Scoping Review
Source: J Med Internet Res. 2025 Mar 27;27:e56147. doi: 10.2196/56147 (PMC11986381; doi:10.2196/56147)
Supplement: Multimedia Appendix 5 [file jmir_v27i1e56147_app5.zip › Multimedia Appendix 5. Quality evaluation of part of the included articles/[48] Does Watching Mukbangs Help You Diet The Effect of The Mukbang on The Desire to Eat.docx]

[9]DOES WATCHING MUKBANGS HELP YOU DIET? THE EFFECT OF THE MUKBANG ON THE

DESIRE TO EAT

| **Assessor:** X.W. and Y.X.X. | | | | | | **Date of Appraisal: 2024.06.18** | | | **Record Number: 48** | | | | | | | | | | | | |
| --- | --- | --- | --- | --- | --- | --- | --- | --- | --- | --- | --- | --- | --- | --- | --- | --- | --- | --- | --- | --- | --- |
| **Study Author:** Wenzhuo Xu | | | | | | **Study Title:** Does Watching Mukbangs Help You Diet? The Effect of the Mukbang on the Desire to Eat | | | **Study Year: 2019** | | | | | | | | | | | | |
|  | | | | | |  | | |  | | | | | | | | | | | | |
| **Internal Validity** | | | | | | | | **Choice - Comments/Justification** | | **Yes** | **No** | | | | **Unclear** | | | **N/A** | | | |
| **Bias related to selection and allocation** | | | | | | | | | | | | | | | | | | | | | |
| **1** | **Was true randomization used for assignment of participants to treatment groups?** | | | | | | |  | |  |  | | | |  | | |  | | | |
| **2** | **Was allocation to treatment groups concealed?** | | | | | | |  | |  |  | | | |  | | |  | | | |
| **3** | **Were treatment groups similar at the baseline?** | | | | | | |  | |  |  | | | |  | | |  | | | |
| **Bias related to administration of intervention/exposure** | | | | | | | | | | | | | | | | | | | | | |
| **4** | **Were participants blind to treatment assignment?** | | | | | | |  | |  |  | | | |  | | |  | | | |
| **5** | **Were those delivering the treatment blind to treatment assignment?** | | | | | | |  | |  |  | | | |  | | |  | | | |
| **6** | **Were treatment groups treated identically other than the intervention of interest?** | | | | | | |  | |  |  | | | |  | | |  | | | |
| **Bias related to assessment, detection and measurement of the outcome** | | | | | | | | | | | | | | | | | | | | | |
| **7** | **Were outcome assessors blind to treatment assignment?** | | | | | | |  | | **Yes** | **No** | | | | **Unclear** | | | **N/A** | | | |
|  | **Outcome 1** | | | | | | |  | |  |  | | | |  | | |  | | | |
|  | **Outcome 2** | | | | | | |  | |  |  | | | |  | | |  | | | |
|  | **Outcome 3** | | | | | | |  | |  |  | | | |  | | |  | | | |
| **8** | **Were outcomes measured in the same way for treatment groups?** | | | | | | |  | | **Yes** | **No** | | | | **Unclear** | | | **N/A** | | | |
|  | **Outcome 1** | | | | | | |  | |  |  | | | |  | | |  | | | |
|  | **Outcome 2** | | | | | | |  | |  |  | | | |  | | |  | | | |
|  | **Outcome 3** | | | | | | |  | |  |  | | | |  | | |  | | | |
| **9** | **Were outcomes measured in a reliable way** | | | | | | |  | | **Yes** | **No** | | | | **Unclear** | | | **N/A** | | | |
|  | **Outcome 1** | | | | | | |  | |  |  | | | |  | | |  | | | |
|  | **Outcome 2** | | | | | | |  | |  |  | | | |  | | |  | | | |
|  | **Outcome 3** | | | | | | |  | |  |  | | | |  | | |  | | | |
| **Bias related to participant retention** | | | | | | | | | | | | | | | | | | | | | |
| **10** | **Was follow up complete and if not, were differences between groups in terms of their follow up adequately described and analysed?** | | | | | | |  | |  | | | | | | | | | | | |
|  | **Outcome 1** | | | | | | |  | | **Yes** | **No** | | | **Unclear** | | | | **N/A** | | | |
|  |  | Result 1 | | | | | |  | |  |  | | | |  | | |  | | | |
|  | **Outcome 2** | | | | | | |  | | **Yes** | **No** | | | | **Unclear** | | | **N/A** | | | |
|  |  | Result 1 | | | | | |  | |  |  | | | |  | | |  | | | |
|  | **Outcome 3** | | | | | | |  | | **Yes** | **No** | | | | **Unclear** | | | **N/A** | | | |
|  |  | Result 1 | | | | | |  | |  |  | | | |  | | |  | | | |
| **11** | **Were participants analysed in the groups to which they were randomized?** | | | | | | |  | |  | | | | | | | | | | | |
|  | **Outcome 1** | | |  | | | |  | | **Yes** | **No** | | | | **Unclear** | | | **N/A** | | | |
|  |  | Result 1 | | | | | |  | |  |  | | | |  | | |  | | | |
|  | **Outcome 2** | | |  | | | |  | | **Yes** | **No** | | | | **Unclear** | | | **N/A** | | | |
|  |  | Result 1 | | | | | |  | |  |  | | | |  | | |  | | | |
|  | **Outcome 3** | | |  | | | |  | | **Yes** | **No** | | | | **Unclear** | | | **N/A** | | | |
|  |  | Result 1 | | | | | |  | |  |  | | | |  | | |  | | | |
| **12** | **Was appropriate statistical analysis used?** | | | | | | |  | |  | |  | | | | |  | |  | | |
|  | **Outcome 1** | | | | | | |  | | **Yes** | | **No** | | | | | **Unclear** | | **N/A** | | |
|  |  | Result 1 | | | | | |  | |  | |  | | | | |  | |  | | |
|  | **Outcome 2** | | | | | | |  | | **Yes** | | **No** | | | | **Unclear** | | | | **N/A** | |
|  |  | Result 1 | | | | | |  | |  | |  | | | |  | | | |  | |
|  | **Outcome 3** | | | | | | |  | | **Yes** | | **No** | | | | **Unclear** | | | | **N/A** | |
|  |  | Result 1 | | | | | |  | |  | |  | | | |  | | | |  | |
|  |  | | | | | | |  | | **Yes** | | | **No** | | | **Unclear** | | | | | **N/A** |
| **13** | **Was the trial design appropriate and any deviations from the standard RCT design (individual randomization, parallel groups) accounted for in the conduct and analysis of the trial?** | | | | | | |  | |  | | |  | | |  | | | | |  |
| **Overall appraisal:** | | | **Include:** | | **Exclude:** | | **Seek Further Info:** | | | | | | | | | | | | | | |
| **Comments:** | | | | | | | | | | | | | | | | | | | | | |
| Table 3 – The JBI Critical Appraisal Tool for RCTs | | | | | | | | | | | | | | | | | | | | | |
