# Supplementary material for: Use of Mukbang in Health Promotion: Scoping Review
Source: J Med Internet Res. 2025 Mar 27;27:e56147. doi: 10.2196/56147 (PMC11986381; doi:10.2196/56147)
Supplement: Multimedia Appendix 5 [file jmir_v27i1e56147_app5.zip › Multimedia Appendix 5. Quality evaluation of part of the included articles/[63] Psychological exploration of “Mukbang” audience from the perspective of structuralism.docx]

**Psychological exploration of “Mukbang” audience from the perspective of structuralism**

Reviewer __X.W. and Y.X.X.__________________ Date___2024.06.18________________

Author__Chuanxi Han_____________________Year__2018__Record Number____63____

|  | Yes | No | Unclear | Not applicable |
| --- | --- | --- | --- | --- |
| 1. Is the generator of the narrative a credible or appropriate source? | ☑ | □ | □ | □ |
| 1. Is the relationship between the text and its context explained? (where, when, who with, how) | ☑ | □ | □ | □ |
| 1. Does the narrative present the events using a logical sequence so the reader or listener can understand how it unfolds? | ☑ | □ | □ | □ |
| 1. Do you, as reader or listener of the narrative, arrive at similar conclusions to those drawn by the narrator? | ☑ | □ | □ | □ |
| 1. Do the conclusions flow from the narrative account? | ☑ | □ | □ | □ |
| 1. Do you consider this account to be a narrative? | ☑ | □ | □ | □ |

Overall appraisal: Include ☑ Exclude □ Seek further info □

Comments (Including reason for exclusion)

________________________________________________________________________________________________________________________________________________________________________________________________________________________________________________________________________________________________
