# Supplementary material for: Use of Mukbang in Health Promotion: Scoping Review
Source: J Med Internet Res. 2025 Mar 27;27:e56147. doi: 10.2196/56147 (PMC11986381; doi:10.2196/56147)
Supplement: Multimedia Appendix 5 [file jmir_v27i1e56147_app5.zip › Multimedia Appendix 5. Quality evaluation of part of the included articles/[24] A STUDY ON POTENTIAL HEALTH ISSUES BEHIND THE POPULARITY OF “MUKBANG” IN CHINA.docx]

A STUDY ON POTENTIAL HEALTH ISSUES BEHIND THE POPULARITY OF “MUKBANG” IN CHINA

Reviewer __X.W. and Y.X.X. ____________Date__2024.06.20_________________

Author___Wang, Shan _____________Year__2020___ Record Number___24___

|  | Yes | No | Unclear | Not applicable |
| --- | --- | --- | --- | --- |
| 1. Is there congruity between the stated philosophical perspective and the research methodology? | ☑ | □ | □ | □ |
| 1. Is there congruity between the research methodology and the research question or objectives? | ☑ | □ | □ | □ |
| 1. Is there congruity between the research methodology and the methods used to collect data? | ☑ | □ | □ | □ |
| 1. Is there congruity between the research methodology and the representation and analysis of data? | ☑ | □ | □ | □ |
| 1. Is there congruity between the research methodology and the interpretation of results? | ☑ | □ | □ | □ |
| 1. Is there a statement locating the researcher culturally or theoretically? | □ | □ | ☑ | □ |
| 1. Is the influence of the researcher on the research, and vice- versa, addressed? | □ | □ | ☑ | □ |
| 1. Are participants, and their voices, adequately represented? | □ | □ | ☑ | □ |
| 1. Is the research ethical according to current criteria or, for recent studies, and is there evidence of ethical approval by an appropriate body? | □ | □ | ☑ | □ |
| 1. Do the conclusions drawn in the research report flow from the analysis, or interpretation, of the data? | ☑ | □ | □ | □ |

Overall appraisal: Include ☑ Exclude □ Seek further info □
