# Supplementary material for: Use of Mukbang in Health Promotion: Scoping Review
Source: J Med Internet Res. 2025 Mar 27;27:e56147. doi: 10.2196/56147 (PMC11986381; doi:10.2196/56147)
Supplement: Multimedia Appendix 5 [file jmir_v27i1e56147_app5.zip › Multimedia Appendix 5. Quality evaluation of part of the included articles/[22] Cloud-Based Commensality Enjoy the Company of Co-diners Without Social Facilitation of Eating.docx]

| **RoB Assessor: X.W. and Y.X.X.** | | **Date of Appraisal: 2024.06.22** | | **Record Number: 22** | | | | |
| --- | --- | --- | --- | --- | --- | --- | --- | --- |
| **Study Author:** Chujun Wang | | **Study Title: Cloud-Based Commensality: Enjoy the Company of Co-diners Without Social Facilitation of Eating** | | **Study Year: 2021** | | | | |
|  | |  | |  | | | | |
| **Internal Validity** | | | **Choice - Comments/Justification** | | **Yes** | **No** | **Unclear** | **N/A** |
| **Bias related to temporal precedence** | | | | | | | | |
| **1** | **Is it clear in the study what is the “cause” and what is the “effect” (i.e. there is no confusion about which variable comes first)?** | |  | |  |  |  |  |
| **Bias related to selection and allocation** | | | | | | | | |
| **2** | **Was there a control group?** | |  | |  |  |  |  |
| **Bias related to confounding factors** | | | | | | | | |
| **3** | **Were participants included in any comparisons similar?** | |  | |  |  |  |  |
| **Bias related to administration of intervention/exposure** | | | | | | | | |
| **4** | **Were the participants included in any comparisons receiving similar treatment/care, other than the exposure or intervention of interest?** | |  | |  |  |  |  |

| **Bias related to assessment, detection and measurement of the outcome** | | | | | | |
| --- | --- | --- | --- | --- | --- | --- |
| **5** | **Were there multiple measurements of the outcome, both pre and post the intervention/exposure?** |  | **Yes** | **No** | **Unclear** | **N/A** |
|  | **Outcome 1** |  |  |  |  |  |
|  |  |  |  |  |  |  |
| **6** | **Were the outcomes of participants included in any comparisons measured in the same way?** |  | **Yes** | **No** | **Unclear** | **N/A** |
|  | **Outcome 1** |  |  |  |  |  |
|  |  |  |  |  |  |  |
| **7** | **Were outcomes measured in a reliable way?** |  | **Yes** | **No** | **Unclear** | **N/A** |
|  | **Outcome 1** |  |  |  |  |  |

| **Bias related to participant retention** | | | | | | | | | | | | | |
| --- | --- | --- | --- | --- | --- | --- | --- | --- | --- | --- | --- | --- | --- |
| **8** | **Was follow-up complete and if not, were differences between groups in terms of their follow-up adequately described and analyzed?** | | | | | |  | |  | | | | |
|  | **Outcome 1** | | | | | |  | | **Yes** | **No** | | **Unclear** | **N/A** |
|  |  | Result 1 | | | | |  | |  |  |  | |  |
|  |  | Result 2 | | | | |  | |  |  |  | |  |
|  |  | Result 3 | | | | |  | |  |  |  | |  |
|  | **Statistical Conclusion Validity** | | | | | | | |  |  |  | |  |
| **9** | **Was appropriate statistical analysis used?** | | | | | | |  |  | | | | |
|  | **Outcome 1** | | |  | | | |  | **Yes** | **No** | **Unclear** | | **N/A** |
|  |  | Result 1 | | | | | |  |  |  |  | |  |
|  |  | Result 2 | | | | | |  |  |  |  | |  |
|  |  | Result 3 | | | | | |  |  |  |  | |  |
| **Overall appraisal:** | | | **Include:** | | **Exclude:** | **Seek Further Info:** | | | | | | | |
| **Comments:** | | | | | | | | | | | | | |

© JBI, 2022. All rights reserved. JBI grants use of these

 tools for research purposes only. All other enquiries

 should be sent to[jbisynthesis@adelaide.edu.au](mailto:jbisynthesis@adelaide.edu.au)
