# Supplementary material for: Use of Mukbang in Health Promotion: Scoping Review
Source: J Med Internet Res. 2025 Mar 27;27:e56147. doi: 10.2196/56147 (PMC11986381; doi:10.2196/56147)
Supplement: Multimedia Appendix 5 [file jmir_v27i1e56147_app5.zip › Multimedia Appendix 5. Quality evaluation of part of the included articles/[15] Study on emotional and physiological changes according to food content types and scenes.docx]

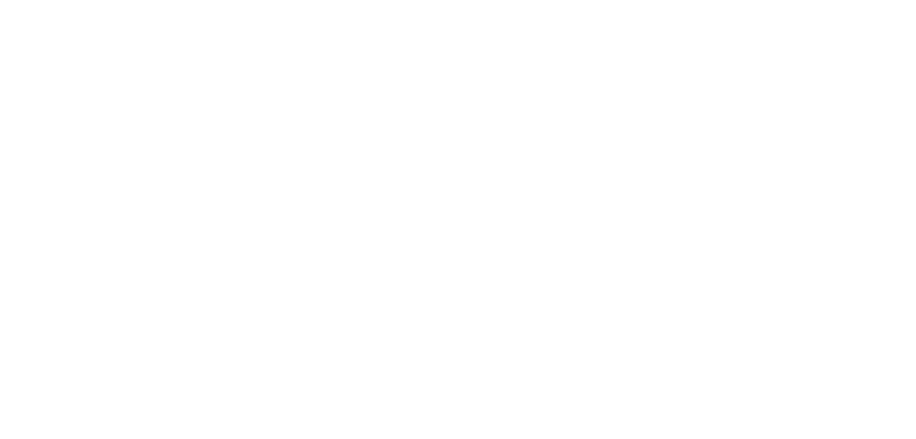


| **RoB Assessor: X.W. and Y.X.X.** | | **Date of Appraisal: 2024.06.26** | | **Record Number: 15** | | | | |
| --- | --- | --- | --- | --- | --- | --- | --- | --- |
| **Study Author: Han, Ye-jin** | | **Study Title: Study on emotional and physiological changes according to food content types and scenes**  **- ASMR Mukbang VS Nomal Mukbang -** | | **Study Year: 2022** | | | | |
|  | |  | |  | | | | |
| **Internal Validity** | | | **Choice - Comments/Justification** | | **Yes** | **No** | **Unclear** | **N/A** |
| **Bias related to temporal precedence** | | | | | | | | |
| **1** | **Is it clear in the study what is the “cause” and what is the “effect” (i.e. there is no confusion about which variable comes first)?** | |  | |  |  |  |  |
| **Bias related to selection and allocation** | | | | | | | | |
| **2** | **Was there a control group?** | | ASMR mukbang video and normal mukbang video | |  |  |  |  |
| **Bias related to confounding factors** | | | | | | | | |
| **3** | **Were participants included in any comparisons similar?** | |  | |  |  |  |  |
| **Bias related to administration of intervention/exposure** | | | | | | | | |
| **4** | **Were the participants included in any comparisons receiving similar treatment/care, other than the exposure or intervention of interest?** | |  | |  |  |  |  |

| **Bias related to assessment, detection and measurement of the outcome** | | | | | | |
| --- | --- | --- | --- | --- | --- | --- |
| **5** | **Were there multiple measurements of the outcome, both pre and post the intervention/exposure?** |  | **Yes** | **No** | **Unclear** | **N/A** |
|  | **Outcome 1** | Questionnaire |  |  |  |  |
|  | **Outcome 2** | Electrocardiogram |  |  |  |  |
|  | **Outcome 3** | Brain wave |  |  |  |  |
|  |  |  |  |  |  |  |
| **6** | **Were the outcomes of participants included in any comparisons measured in the same way?** |  | **Yes** | **No** | **Unclear** | **N/A** |
|  | **Outcome 1** | Questionnaire |  |  |  |  |
|  | **Outcome 2** | Electrocardiogram |  |  |  |  |
|  | **Outcome 3** | Brain wave |  |  |  |  |
|  |  |  |  |  |  |  |
| **7** | **Were outcomes measured in a reliable way?** |  | **Yes** | **No** | **Unclear** | **N/A** |
|  | **Outcome 1** | Questionnaire |  |  |  |  |
|  | **Outcome 2** | Electrocardiogram |  |  |  |  |
|  | **Outcome 3** | Brain wave |  |  |  |  |

| **Bias related to participant retention** | | | | | | | | | | | | | |
| --- | --- | --- | --- | --- | --- | --- | --- | --- | --- | --- | --- | --- | --- |
| **8** | **Was follow-up complete and if not, were differences between groups in terms of their follow-up adequately described and analyzed?** | | | | | |  | |  | | | | |
|  | **Outcome 1** | | | | | | Questionnaire | | **Yes** | **No** | | **Unclear** | **N/A** |
|  |  | Result 1 | | | | | The subjective questionnaire showed no difference between the two groups. There was no significant difference between the two groups of subjects in their perceived emotional changes after watching different types of mukbang. | |  |  |  | |  |
|  | **Outcome 2** | | | | | | Brain wave | | **Yes** | **No** | **Unclear** | | **N/A** |
|  |  | Result 1 | | | | | The EEG showed significant difference between the two groups before and after the experiment. | |  |  |  | |  |
|  |  | Result 2 | | | | | The EEG collected while watching the video showed that the EEG of the subjects in different time periods was different; There were differences in brain waves between the two groups when they watched the videos. | |  |  |  | |  |
|  | **Outcome 3** | | | | | | Electrocardiogram | | **Yes** | **No** | **Unclear** | | **N/A** |
|  |  | Result 1 | | | | | The ECG showed significant changes before and after the scan | |  |  |  | |  |
|  | **Statistical Conclusion Validity** | | | | | | | |  |  |  | |  |
| **9** | **Was appropriate statistical analysis used?** | | | | | | |  |  | | | | |
|  | **Outcome 1** | | |  | | | | Questionnaire | **Yes** | **No** | **Unclear** | | **N/A** |
|  |  | Result 1 | | | | | | The subjective questionnaire showed no difference between the two groups. There was no significant difference between the two groups of subjects in their perceived emotional changes after watching different types of mukbang. |  |  |  | |  |
|  | **Outcome 2** | | |  | | | | Brain wave | **Yes** | **No** | **Unclear** | | **N/A** |
|  |  | Result 1 | | | | | | The EEG showed significant difference between the two groups before and after the experiment. |  |  |  | |  |
|  |  | Result 2 | | | | | | The EEG collected while watching the video showed that the EEG of the subjects in different time periods was different; There were differences in brain waves between the two groups when they watched the videos. |  |  |  | |  |
|  | **Outcome 3** | | |  | | | | Electrocardiogram | **Yes** | **No** | **Unclear** | | **N/A** |
|  |  | Result 1 | | | | | | The ECG showed significant changes before and after the scan |  |  |  | |  |
|  | | | | | | | | | | | | | |
| **Overall appraisal:** | | | **Include:** | | **Exclude:** | **Seek Further Info:** | | | | | | | |
| **Comments:** | | | | | | | | | | | | | |
