# Supplementary material for: Use of Mukbang in Health Promotion: Scoping Review
Source: J Med Internet Res. 2025 Mar 27;27:e56147. doi: 10.2196/56147 (PMC11986381; doi:10.2196/56147)
Supplement: Multimedia Appendix 5 [file jmir_v27i1e56147_app5.zip › Multimedia Appendix 5. Quality evaluation of part of the included articles/[17] Watching a food-related television show and caloric intake. A laboratory study.docx]

| **Assessor: X.W. and Y.X.X.** | | | | | **Date of Appraisal: 2024.06.18** | | **Record Number: 17** | | | | | | | | | | | | |
| --- | --- | --- | --- | --- | --- | --- | --- | --- | --- | --- | --- | --- | --- | --- | --- | --- | --- | --- | --- |
| **Study Author: Jamie S. Bodenlos** | | | | | **Study Title: Watching a food-related television show and caloric intake. A laboratory study** | | **Study Year: 2013** | | | | | | | | | | | | |
|  | | | | |  | |  | | | | | | | | | | | | |
| **Internal Validity** | | | | | | **Choice - Comments/Justification** | | **Yes** | **No** | | | **Unclear** | | | | **N/A** | | | |
| **Bias related to selection and allocation** | | | | | | | | | | | | | | | | | | | |
| **1** | **Was true randomization used for assignment of participants to treatment groups?** | | | | |  | |  |  | | |  | | | |  | | | |
| **2** | **Was allocation to treatment groups concealed?** | | | | |  | |  |  | | |  | | | |  | | | |
| **3** | **Were treatment groups similar at the baseline?** | | | | |  | |  |  | | |  | | | |  | | | |
| **Bias related to administration of intervention/exposure** | | | | | | | | | | | | | | | | | | | |
| **4** | **Were participants blind to treatment assignment?** | | | | |  | |  |  | | |  | | | |  | | | |
| **5** | **Were those delivering the treatment blind to treatment assignment?** | | | | |  | |  |  | | |  | | | |  | | | |
| **6** | **Were treatment groups treated identically other than the intervention of interest?** | | | | |  | |  |  | | |  | | | |  | | | |
| **Bias related to assessment, detection and measurement of the outcome** | | | | | | | | | | | | | | | | | | | |
| **7** | **Were outcome assessors blind to treatment assignment?** | | | | |  | | **Yes** | **No** | | | **Unclear** | | | | **N/A** | | | |
|  | **Outcome 1** | | | | | A post-taste-testing questionnaire | |  |  | | |  | | | |  | | | |
|  | **Outcome 2** | | | | | Feeding experiment | |  |  | | |  | | | |  | | | |
|  |  | | | | |  | |  |  | | |  | | | |  | | | |
| **8** | **Were outcomes measured in the same way for treatment groups?** | | | | |  | | **Yes** | **No** | | | **Unclear** | | | | **N/A** | | | |
|  | **Outcome 1** | | | | | A post-taste-testing questionnaire | |  |  | | |  | | | |  | | | |
|  | **Outcome 2** | | | | | Feeding experiment | |  |  | | |  | | | |  | | | |
|  |  | | | | |  | |  |  | | |  | | | |  | | | |
| **9** | **Were outcomes measured in a reliable way** | | | | |  | | **Yes** | **No** | | | **Unclear** | | | | **N/A** | | | |
|  | **Outcome 1** | | | | | A post-taste-testing questionnaire | |  |  | | |  | | | |  | | | |
|  | **Outcome 2** | | | | | Feeding experiment | |  |  | | |  | | | |  | | | |
|  |  | | | | |  | |  | | | | | | | | | | | |
| **Bias related to participant retention** | | | | | | | | | | | | | | | | | | | |
| **10** | **Was follow up complete and if not, were differences between groups in terms of their follow up adequately described and analysed?** | | | | |  | |  | | | | | | | | | | | |
|  | **Outcome 1** | | | | | A post-taste-testing questionnaire | | **Yes** | **No** | | | | **Unclear** | | | **N/A** | | | |
|  |  | Result 1 | | | | Hunger and desirability | |  |  | | |  | | | |  | | | |
|  |  | Result 2 | | | | Eating behaviors | |  |  | | |  | | | |  | | | |
|  |  | Result 3 | | | | Three Factor Eating Questionnaire (TFEQ-51) | |  |  | | |  | | | |  | | | |
|  | **Outcome 2** | | | | |  | | **Yes** | **No** | | | **Unclear** | | | | **N/A** | | | |
|  |  | Result 1 | | | | Feeding experiment | |  |  | | |  | | | |  | | | |
|  |  |  | | | | | |  |  | | |  | | | |  | | | |
|  | **Statistical Conclusion Validity** | | | | | | |  |  | | |  | | | |  | | | |
| **11** | **Were participants analysed in the groups to which they were randomized?** | | | | | | | | | | | | | | | | | | |
|  | **Outcome 1** | | | | | A post-taste-testing questionnaire | | **Yes** | **No** | | | **Unclear** | | | | **N/A** | | | |
|  |  | Result 1 | | | | Hunger and desirability | |  |  | | |  | | | |  | | | |
|  |  | Result 2 | | | | Eating behaviors | |  |  | | |  | | | |  | | | |
|  |  | Result 3 | | | | Three Factor Eating Questionnaire (TFEQ-51) | |  |  | | |  | | | |  | | | |
|  | **Outcome 2** | | | | |  | | **Yes** | **No** | | | **Unclear** | | | | **N/A** | | | |
|  |  | Result 1 | | | | Feeding experiment | |  |  | | |  | | | |  | | | |
|  |  | | | | | | |  | |  | | | | |  | |  | | |
| **12** | **Was appropriate statistical analysis used?** | | | | |  | |  | |  | | | | |  | |  | | |
|  | **Outcome 1** | | | | | A post-taste-testing questionnaire | | **Yes** | | **No** | | | | | **Unclear** | | **N/A** | | |
|  |  | Result 1 | | | | Hunger and desirability | |  | |  | | | | |  | |  | | |
|  |  | Result 2 | | | | Eating behaviors | |  | |  | | | |  | | | |  | |
|  |  | Result 3 | | | | hree Factor Eating Questionnaire (TFEQ-51) | |  | |  | | | |  | | | |  | |
|  | **Outcome 2** | | | | |  | | **Yes** | | **No** | | | | **Unclear** | | | | **N/A** | |
|  |  | Result 1 | | | | Feeding experiment | |  | |  | | | |  | | | |  | |
|  |  | | | | |  | | **Yes** | | | **No** | | | **Unclear** | | | | | **N/A** |
| **13** | **Was the trial design appropriate and any deviations from the standard RCT design (individual randomization, parallel groups) accounted for in the conduct and analysis of the trial?** | | | | |  | |  | | |  | | |  | | | | |  |
| **Overall appraisal:** | | | **Include:** | **Exclude:** | | **Seek Further Info:** | | | | | | | | | | | | | |
| **Comments:** | | | | | | | | | | | | | | | | | | | |
| Table 3 – The JBI Critical Appraisal Tool for RCTs | | | | | | | | | | | | | | | | | | | |

question Guidance

**How to use the JBI Tools for the Assessment of Risk of Bias**

Each question presented in a JBI tool for the assessment of risk of bias for quantitative study designs answers a question related to certain *categories of validity* and *domains of bias*. The concept of validity is often used when referring to the soundness or rigour in which a study was conducted, and whether the results of the study are likely to be true and generalizable. At JBI we have broken this down to include three separate categories that constitute *validity*, these include internal validity, external validity, statistical conclusion validity. In addition, we have also included comprehensiveness of reporting.

Questions categorised as “Internal Validity” are then further organised to specific domains of bias in which they relate. The domains of bias that are used as an indicator of internal validity include bias related to selection and allocation, bias related to administration of the intervention/exposure, bias related to assessment, detection and measurement of the outcome, bias related to participant retention, bias related to temporal precedence, bias related to classification of the exposure, bias related to confounding factors and bias related to selective reporting and/or publication bias.

For more information, please see [Barker et al. 2022](https://journals.lww.com/jbisrir/Abstract/9900/Revising_the_JBI_quantitative_critical_appraisal.85.aspx)

**Question 1: Was true randomization used for assignment of participants to treatment groups?**

*Category: Internal validity*

*Domain: Bias related to selection and allocation*

*Appraisal: Study level*

If participants are not allocated to treatment and control groups by random assignment there is a risk that this assignment to groups can be influenced by the known characteristics of the participants themselves. These known characteristics of the participants may distort the comparability of the groups (i.e. does the intervention group contain more people over the age of 65 as compared to the control?). A true random assignment of participants to the groups means that a procedure is used that allocates the participants to groups purely based on chance, not influenced by any known characteristics of the participants. Reviewers should check the details about the randomization procedure used for allocation of the participants to study groups. Was a true chance (random) procedure used? For example, was a list of random numbers used? Was a computer-generated list of random numbers used? Was a statistician, external to the research team consulted for the randomization sequence generation? Additionally, reviewers should check that the authors are not stating they have used random approaches when they have instead used systematic approaches (such as allocating by days of the week).

**Question 2: Was allocation to groups concealed?**

*Category: Internal validity*

*Domain: Bias related to selection and allocation*

*Appraisal: Study level*

If those allocating participants to the compared groups are aware of which group is next in the allocation process, (i.e., the treatment or control group) there is a risk that they may deliberately and purposefully intervene in the allocation of patients. This may result in the preferential allocation of patients to the treatment group or to the control group. This may directly distort the results of the study, as participants no longer have an equal and random chance to belong to each group compared. Concealment of allocation refers to procedures that prevent those allocating patients from knowing before allocation which treatment or control is next in the allocation process. Reviewers should check the details about the procedure used for allocation concealment. Was an appropriate allocation concealment procedure used? For example, was central randomization used? Were sequentially numbered, opaque and sealed envelopes used? Were coded drug packs used?

**Question 3: Were treatment groups similar at the baseline?**

*Category: Internal validity*

*Domain: Bias related to selection and allocation*

*Appraisal: Study level*

As with question 1, any differences between the known characteristics of participants included in compared groups constitutes a threat to internal validity. If differences in these characteristics do exist, then there is potential that the ‘effect’ cannot be attributed to the potential ‘cause’ (the examined intervention or treatment). This is because the ‘effect’ may be explained by the differences between participant characteristics and not due to the intervention/treatment of interest. Reviewers should check the characteristics reported for participants. Are the participants from the compared groups similar with regards to the characteristics that may explain the effect even in the absence of the ‘cause’, for example, age, severity of the disease, stage of the disease, co-existing conditions and so on? Reviewers should check the proportions of participants with specific relevant characteristics in the compared groups. [Note: **Do NOT** only consider the P-value for the statistical testing of the differences between groups with regards to the baseline characteristics.]

**Question 4: Were participants blind to treatment assignment?**

*Category: Internal validity*

*Domain: Bias related to administration of intervention/exposure*

*Appraisal: Study level*

Participants that are aware of their allocation to either the treatment or the control may behave, respond, or react differently to their assigned treatment (or control) than compared to participants that remain unaware of their allocation. Blinding of participants is a technique used to minimize this risk. Blinding refers to procedures that prevent participants from knowing which group they are allocated. If blinding has been followed, participants are not aware if they are in the group receiving the treatment of interest or if they are in any other group receiving the control interventions. Reviewers should check the details reported in the article about the blinding of participants with regards to treatment assignment. Was an appropriate blinding procedure used? For example, were identical capsules or syringes used? Were identical devices used? Be aware of different terms used, blinding is sometimes also called masking.

**Question 5: Were those delivering the treatment blind to treatment assignment?**

*Category: Internal validity*

*Domain: Bias related to administration of intervention/exposure*

*Appraisal: Study level*

Like question 4, those delivering the treatment that are aware of participant allocation to either treatment or control, may treat participants differently than compared to those that remain unaware of participant allocation. There is the risk that any potential change in behaviour may influence the implementation of the compared treatments and the results of the study may be distorted. Blinding of those delivering treatment is used to minimize this risk. When this level of blinding has been achieved, those delivering the treatment are not aware if they are treating the group receiving the treatment of interest or if they are treating any other group receiving the control interventions. Reviewers should check the details reported in the article about the blinding of those delivering treatment with regards to treatment assignment. Is there any information in the article about those delivering the treatment? Were those delivering the treatment unaware of the assignments of participants to the compared groups?

**Question 6: Were treatment groups treated identically other than the intervention of interest?**

*Category: Internal validity*

*Domain: Bias related to administration of intervention/exposure*

*Appraisal: Study level*

To attribute the ‘effect’ to the ‘cause’, (assuming no bias related to selection and allocation) there should be no other difference between the groups in terms of treatment or care received, other than the treatment or intervention controlled by the researchers. If there are other exposures or treatments occurring at the same time with the ‘cause’ (the treatment or intervention of interest), then the ‘effect’ can potentially not be attributed to the examined ‘cause’ (the investigated treatment). This is because it is plausible that the ‘effect’ may be explained by these other exposures or treatments that occurred at the same time with the ‘cause’. Reviewers should check the reported exposures or interventions received by the compared groups. Are there other exposures or treatments occurring at the same time with the ‘cause’? Is it plausible that the ‘effect’ may be explained by other exposures or treatments occurring at the same time with the ‘cause’? Is it clear that there is no other difference between the groups in terms of treatment or care received, other than the treatment or intervention of interest?

**Question 7: Were outcome assessors blind to treatment assignment?**

*Category: Internal validity*

*Domain: Bias related to assessment, detection and measurement of the outcome*

*Appraisal: Outcome level*

Like question 4 and 5, those assessing the outcomes that are aware of participant allocation to either treatment or control, may treat participants differently than compared to those that remain unaware of participant allocation. Therefore, there is a risk that the measurement of the outcomes between groups may be distorted, and the results of the study may themselves be distorted. Blinding of outcomes assessors is used in order to minimize this risk. Reviewers should check the details reported in the article about the blinding of outcomes assessors with regards to treatment assignment. Is there any information in the article about outcomes assessors? Were those assessing the treatment’s effects on outcomes unaware of the assignments of participants to the compared groups?

**Question 8: Were outcomes measured in the same way for treatment groups?**

*Category: Internal validity*

*Domain: Bias related to assessment, detection and measurement of the outcome*

*Appraisal: Outcome level*

If the outcome is not measured in the same way in the compared groups, there is a threat to the internal validity of a study. Any differences in outcome measurements may be due to the method of measurement employed between the two groups, and not due to the intervention/treatment of interest. Reviewers should check if the outcomes were measured in the same way. Same instrument or scale used? Same measurement timing? Same measurement procedures and instructions?

**Question 9: Were outcomes measured in a reliable way?**

*Category: Internal validity*

*Domain: Bias related to assessment, detection and measurement of the outcome*

*Appraisal: Outcome level*

Unreliability of outcome measurements is one threat that weakens the validity of inferences about the statistical relationship between the ‘cause’ and the ‘effect’ estimated in a study exploring causal effects. Unreliability of outcome measurements is one of the different plausible explanations for errors of statistical inference with regards to the existence and the magnitude of the effect determined by the treatment (‘cause’). Reviewers should check the details about the reliability of the measurement used, such as the number of raters, training of raters, the intra-rater and the inter-raters reliability within the study (not as reported in external sources). This question is about the reliability of the measurement performed in the study, it is not about the validity of the measurement instruments/scales used in the study. Finally, some outcomes may not rely on instruments or scales (e.g. death) and reliability of the measurements may need to be assessed in the context of the study being reviewed. [Note: Two other important threats that weaken the validity of inferences about the statistical relationship between the ‘cause’ and the ‘effect’ are low statistical power and the violation of the assumptions of statistical tests. These other two threats are explored within Question 12).]

**Question 10: Was follow up complete and if not, were differences between groups in terms of their follow up adequately described and analysed?**

*Category: Internal validity*

*Domain: Bias related to participant retention*

*Appraisal: Result level*

For this question, follow up refers to the period from the moment of randomization to any point in which the groups are compared during the trial. This question asks if there is complete knowledge (measurements, observations etc.) for the entire duration of the trial for all randomly allocated participants. If there is incomplete follow up from all randomly allocated participants, this is known as post-assignment attrition. As RCTs are not perfect, there is almost always post-assignment attrition, and the focus of this question is on the appropriate exploration of post-assignment attrition. If differences do exist with regards to the post-assignment attrition between the compared groups of an RCT, then there is a threat to the internal validity of that study. This is because these differences may provide a plausible alternative explanation for the observed ‘effect’ even in the absence of the ‘cause’ (the treatment or intervention of interest). It is important to note that with regards post-assignment attrition, it is not enough to know the number of participants and the proportions of participants with incomplete data; the reasons for loss to follow up are essential in the analysis of risk of bias.

Reviewers should check if there were differences with regards to the loss to follow up between the compared groups. If follow up was incomplete (incomplete information on all participants), examine the reported details about the strategies used to address incomplete follow up. This can include descriptions of loss to follow up (absolute numbers; proportions; reasons for loss to follow up) and impact analyses (the analyses of the impact of loss to follow up on results). Was there a description of the incomplete follow up including the number of participants and the specific reasons for loss to follow up? Even if follow up was incomplete, but balanced between groups, if the reasons for loss to follow up are different (e.g., side effects caused by the intervention of interest), these may impose a risk of bias if not appropriately explored in the analysis. If there are differences between groups with regards to the loss to follow up (numbers/proportions and reasons), was there an analysis of patterns of loss to follow up? If there are differences between the groups with regards to the loss to follow up, was there an analysis of the impact of the loss to follow up on the results? [Note: Question 10 is NOT about intention-to-treat (ITT) analysis; question 11 is about ITT analysis.]

**Question 11: Were participants analysed in the groups to which they were randomized?**

*Category: Statistical conclusion validity*

*Appraisal: Result level*

This question is about the intention-to-treat (ITT) analysis. There are different statistical analysis strategies available for the analysis of data from RCTs, such as intention-to-treat analysis (known also as intent to treat; abbreviated, ITT), per-protocol analysis, and as-treated analysis. In the ITT analysis the participants are analysed in the groups to which they were randomized. This means that regardless of whether participants received the intervention or control as assigned, were complaint with their planned assignment or participated for the entire study duration, they are still included in the analysis. The ITT analysis compares the outcomes for participants from the initial groups created by the initial random allocation of participants to those groups. Reviewers should check if an ITT analysis was reported; check the details of the ITT. Were participants analysed in the groups to which they were initially randomized, regardless of whether they participated in those groups, and regardless of whether they received the planned interventions?

[Note: The ITT analysis is a type of statistical analysis recommended in the Consolidated Standards of Reporting Trials (CONSORT) statement on best practices in trials reporting, and it is considered a marker of good methodological quality of the analysis of results of a randomized trial. The ITT is estimating the effect of offering the intervention, that is, the effect of instructing the participants to use or take the intervention; the ITT it is not estimating the effect of receiving the intervention of interest.]

**Question 12: Was appropriate statistical analysis used?**

*Category: Statistical conclusion validity*

*Appraisal: Result level*

Inappropriate statistical analysis may cause errors of statistical inference with regards to the existence and the magnitude of the effect determined by the treatment (‘cause’). Low statistical power and the violation of the assumptions of statistical tests are two important threats that weaken the validity of inferences about the statistical relationship between the ‘cause’ and the ‘effect’. Reviewers should check the following aspects: were the assumptions of the statistical tests were respected; if appropriate statistical power analysis was performed; if appropriate effect sizes were used; if appropriate statistical methods were used given the nature of the data and the objectives of statistical analysis (association between variables; prediction; survival analysis etc.).

**Question 13: Was the trial design appropriate and any deviations from the standard RCT design (individual randomization, parallel groups) accounted for in the conduct and analysis of the trial?**

*Category: Statistical conclusion validity*

*Appraisal: Study level*

The typical, parallel group RCT may not always be appropriate depending on the nature of the question being asked. Therefore, some additional RCT designs may have been employed that each come with their own additional considerations.

Crossover trials should only be conducted in people with a chronic, stable condition, where the intervention produces a short-term effect (i.e. relief in symptoms). Crossover trials should ensure there is an appropriate period of washout between treatments. This may also be considered under question 6.

Cluster RCTs randomize groups individuals or groups (e.g. communities, wards etc.) , forming ‘clusters.’ When we are assessing outcomes on an individual level in cluster trials, there are unit-of-analysis issues, as individuals within a cluster are correlated. This should be considered by the study authors when conducting analysis, and ideally authors will report the intra-cluster correlation coefficient. This may also be considered under question 12.

Stepped wedge RCTs may be appropriate to establish when and how a beneficial intervention may be best implemented within a defined setting, or due to logistical, practical, or financial considerations in the roll out of a new treatment/intervention. Data analysis in these trials should be conducted appropriately, considering the effects of time. This may also be considered under question 12.
