# Supplementary material for: Use of Mukbang in Health Promotion: Scoping Review
Source: J Med Internet Res. 2025 Mar 27;27:e56147. doi: 10.2196/56147 (PMC11986381; doi:10.2196/56147)
Supplement: Multimedia Appendix 5 [file jmir_v27i1e56147_app5.zip › Multimedia Appendix 5. Quality evaluation of part of the included articles/[18] Obesity and food-related content aimed at children on YouTube.docx]

Obesity and food-related content aimed at children on YouTube

|  | 评价条目 | 评价结果 | | |
| --- | --- | --- | --- | --- |
|  |  | 是 | 否 | 不清楚 |
| 筛选问题 | S1.有明确的研究问题吗？ | 1 |  |  |
|  | S2.收集的数据是否可以回答研究问题？ | 1 |  |  |
| 定性研究 | 1.1定性方法是否适合回答该研究问题？ | 1 |  |  |
|  | 1.2定性资料收集方法是否足以解答该研究问题？ | 1 |  |  |
|  | 1.3所收集的资料是否足以提炼出研究发现？ | 1 |  |  |
|  | 1.4结果的解释是否有足够的资料支持？ | 1 |  |  |
|  | 1.5定性资料的来源、收集、分析和解释之间是否存在一致性？ | 1 |  |  |
| 定量描述性研究 | 4.1抽样方法对于回答研究问题是否合适？ | 1 |  |  |
|  | 4.2样本对目标人群有代表性吗？ | 1 |  |  |
|  | 4.3测量方法是否合适？ | 1 |  |  |
|  | 4.4无应答偏倚的风险是否很低？ | 1 |  |  |
|  | 4.5统计分析方法是否恰当？ | 1 |  |  |
| 混合研究评级 | 5.1是否有足够的理由使用混合方法设计来回答研究问题？ | 1 |  |  |
|  | 5.2研究的不同组成部分是否有效地整合在一起以回答研究问题？ | 1 |  |  |
|  | 5.3是否充分解释了定性和定量两个部分的结果？ | 1 |  |  |
|  | 5.4是否充分解决了定量和定性结果之间的分歧和异质性问题？ |  |  | 0 |
|  | 5.5研究的不同组成部分是否符合每个既往常规方法中涉及到的质量标准？ | 1 |  |  |

总分:17分，得分:16分 符合94%质量评价标准。
