# Supplementary material for: Use of Mukbang in Health Promotion: Scoping Review
Source: J Med Internet Res. 2025 Mar 27;27:e56147. doi: 10.2196/56147 (PMC11986381; doi:10.2196/56147)
Supplement: Multimedia Appendix 5 [file jmir_v27i1e56147_app5.zip › Multimedia Appendix 5. Quality evaluation of part of the included articles/[27] Dietary life and mukbang- and cookbang-watching status of university students majoring in food and nutrition before and after COVID-19 outbreak.docx]

**Dietary life and mukbang- and cookbang-watching status of university students majoring in food and nutrition before and after COVID-19 outbreak**

Reviewer __X.W. and Y.X.X.______________Date__2024.06.22________________

Author___Hyunjoo Kang________________Year__2021__ Record Number___27____

|  | Yes | No | Unclear | Not applicable |
| --- | --- | --- | --- | --- |
| 1. Were the criteria for inclusion in the sample clearly defined? | □ | □ | ☑ | □ |
| 1. Were the study subjects and the setting described in detail? | ☑ | □ | □ | □ |
| 1. Was the exposure measured in a valid and reliable way? | ☑ | □ | □ | □ |
| 1. Were objective, standard criteria used for measurement of the condition? | □ | □ | ☑ | □ |
| 1. Were confounding factors identified? | ☑ | □ | □ | □ |
| 1. Were strategies to deal with confounding factors stated? | ☑ | □ | □ | □ |
| 1. Were the outcomes measured in a valid and reliable way? | ☑ | □ | □ | □ |
| 1. Was appropriate statistical analysis used? | ☑ | □ | □ | □ |

Overall appraisal: Include ☑ Exclude □ Seek further info □
