# Supplementary material for: Use of Mukbang in Health Promotion: Scoping Review
Source: J Med Internet Res. 2025 Mar 27;27:e56147. doi: 10.2196/56147 (PMC11986381; doi:10.2196/56147)
Supplement: Multimedia Appendix 6 [file jmir_v27i1e56147_app6.docx]

| No. | Authors | Title | Country | Output type / Methodology/ Language | Sample | Main findings |
| --- | --- | --- | --- | --- | --- | --- |
| [2] | Glen Donnar (2017) | ‘Food porn’ or intimate sociality: committed celebrity and cultural performances of overeating in *meokbang* | Australia | Journal paper: Commentary, written in English | Not applicable | This research mainly analyzed the role of mukbang in the social environment and culture of Korea, explored the cultural connotation of mukbang, and the interaction and relationship between mukbang and society. At the same time, the health-related information contained in mukbang was also explored. It reveals the reasons for mukbang's existence and popularity under the influence of society and culture, as well as its impact on Korean society and people. |
| [13] | Strand M, Gustafsson SA (2020) | Mukbang and disordered eating: a netnographic analysis of online eating broadcasts. | Sweden | Journal paper: Qualitative study, written in Korean | In total, 1316 user comments (986 YouTube user comments and 330 Reddit user comments) were included in the final qualitative content analysis. | The aim of this study was to explore how viewers of mukbang videos relate their audience experiences to symptoms of disordered eating. Authors have concluded two overarching themes were identified: a viewer perspective, and a participant perspective. Meanwhile, several topical categories emerged, describing how watching mukbang can both limit and increase eating, reduce loneliness and guilt, and become self-destructive. |
| [14] | James M, Ranasinghe N, Tang A, Oehlberg L (2022) | Watch your flavors: augmenting people's flavor perceptions and associated  emotions based on videos watched while eating | America | Conference: Experimental study, written in English | A total of 35 participants aged between 18-50 years were joined in this study. | This study was conducted with 35 participants who evaluated changes in their flavor perception with six different videos watched while eating white rice. This study revealed that the participants’ flavor perceptions are augmented based on different video content, while eating positively influences participants’ flavor perception in terms of taste sensations, liking, and emotions, indicating significant differences in their perceived taste sensations (e.g., increased perception of salty and spicy sensations). |
| [15] | Han Y (2022) | Study on emotional and physiological changes according to food content types and scenes—ASMR mukbang vs  nomal mukbang | Korea | Journal paper: Experimental study, written in Korean | This study was conducted on people in their 20s or older who have experience watching mukbang broadcasts in Korean. A total of 28 people have participated in the study. | This study aimed to confirm emotional and physiological changes (EEG, HRV) according to the type of food content image and scene development, and specifically, how individuals' emotional and physiological changes occur according to the content composition of general and ASMR eating video contents. The study has confirmed that higher emotional and arousal reactions were induced when a person appeared and showed eating than when cooking. As a result of this study, it was confirmed that mukbang content induces excessive arousal rather than relaxation experiences. |
| [17] | Bodenlos JS, Wormuth BM  (2013) | Watching a food-related television show and caloric intake | America | Journal paper: RCT, written in English | A total of 80 participants aged between 18-22 were recruited from psychology classes at a small college in the northeast. | In the present study, an experimental design was used to examine if watching a television program on food, a cooking show, affected caloric intake. The study has found that watching food programs increases consumption of sweet foods, which may indicate that the recent popularity and influx of cooking and food shows is a factor that contributes to the obesogenic environment and can affect weight gain and obesity. |
| [18] | Castelló-Martínez A, Tur-Viñes V (2020) | Obesity and food-related content aimed at children on YouTube | Spain | Journal paper: An exploratory qualitative-quantitative study, written in English | The final sample consisted of 304 videos published by both types of channels in YouTube in 2019. | This study has analyzed and compared the advertising by food brands on regular TV networks (traditional advertising) with videos by child YouTubers (influencing) in Spanish. This study reveals some troubling media practices associated with obesogenic issues. A responsible commitment is required from these spaces, with the decisive promotion of healthy eating habits to combat the pandemic of obesity, and its associated pathologies, in a population as prone to high risk as children. |
| [22] | Wang C, Peng Y, Qiu L, Wan X (2021) | Cloud-based commensality: enjoy the company of co-diners without social facilitation  of eating | China | Journal paper: Quasi-experimental study, written in English | 95 healthy Chinese young adults ranging from 18 to 29 years were recruited to take part in this study. | The purpose of this study was to explore the effects of different meal styles on loneliness, appetite, food intake and food choice when eating alone.  This study has found that mukbang can release loneliness when a person eats alone, but still carries the risk of increasing food intake and/or switching to unhealthy food choices. If mukbang viewers try to obtain vicarious satisfaction of a desire to eat unhealthy foods and urge themselves to engage in healthy eating, our results suggest that the outcomes are more likely to be opposite to what they wish for. |
| [24] | Wang S (2020) | A Study on Potential Health Issues Behind the Popularity of "Mukbang" in China | America | Thesis: Qualitative study, written in English | The texts of mukbang videos, the follow up comments, and the intersection between mukbangers and viewers | The study has found that mukbang has caused a certain harmful impact on the perception of eating norms and eating behaviors of some viewers. By constantly promoting this unhealthy diet as well as intentionally or unintentionally promoting the way mukbanger eats, viewers may ignore the existing concept of healthy eating so as to imitate the dietary habits that are full of novelty and pleasure. |
| [25] | Teng S, Khong KW, Pahlevan Sharif S, Ahmed A (2020) | YouTube video comments on healthy eating: descriptive and predictive  analysis | Malaysia | Journal paper: Qualitative study, written in English | 10 videos, 5756 comments and replies posted under the 10 videos | According to this study, people’s attitudes toward healthy eating are generally positive. A principal finding of this study is that people hold complex and multifaceted beliefs about healthy eating in the context of YouTube videos.  Another conclusion that can be drawn is that the more health-related information presented in mukbang, the more health-related knowledge viewers can gain from the video, and the viewers' health beliefs will also be correspondingly enhanced. |
| [26] | James MN, Ranasinghe N, Tang A, Oehlberg L (2022) | Flavor-videos: Enhancing the flavor perception of food while eating with  videos | America | Conference: Experimental study, written in English | A total of 35 participants from different parts of the United States via social media. | This study conducted an user trial to analyze the influences of different video types watched while eating on flavor perceptions, especially related to taste sensations, liking, and emotions. The findings revealed that participants perceived positive emotional changes and reported significant differences in their augmented taste sensations (e.g., spicy and salty) with different food-based videos. These findings provided insights into using food-related videos (like mukbang videos) to promote healthier eating (digital augmentation without altering the food). |
| [27] | Kang H, Yun S, Lee H (2021) | Dietary life and mukbang- and cookbang-watching status of university students majoring in food  and nutrition before and after COVID-19 outbreak | Korea | Journal paper: Cross-sectional study, written in English | A total of 167 students of a college in Gyeonggi, Korea, participated in an online survey. | This study sought to determine how the COVID-19 pandemic has impacted dietary life and the mukbang- and cookbang-watching patterns of college students, especially majoring in Food Nutrition, who are equipped with a stronger orientation toward health. In this study, COVID-19 was the main influencing factor. The study has found that COVID-19 pandemic could have had positive impacts on the diet and lifestyle behaviors of young adults with a FN background. |
| [28] | Yun S, Kang H, Lee H (2020) | Mukbang- and cookbang-watching status and dietary life of university students who are not food  and nutrition majors | Korea | Journal paper: Cross-sectional study, written in English | A total of 380 students who were not majoring in food and nutrition at a university in Gyeonggi, Korea participated in this study. | This study sought to identify patterns of Mukbang- or Cookbang-watching and their potential association with the dietary life of university students who are not food and nutrition majors. The study found that mukbang was more likely to lead to overeating or make viewers' eating habits worse. Cookbang, on the other hand, usually makes the audience more willing to try cooking their own food, thus reducing take-out or eating out, which in turn makes the audience eat healthier. |
| [29] | Kircaburun K, Savcı M, Emirtekin E, Griffiths MD (2022) | Uses and gratifications of problematic mukbang watching—the role  of eating and social gratification: a pilot study | United Kingdom | Journal paper: Cross-sectional questionnaire-based study, written in English | A total of 170 students from Yaşar University who watched mukbang in the past year participated in the study. | This study is one of the preliminary attempts to investigate problematic mukbang watching and its psychological and motivational correlates. It appears that satisfying real life eating needs by obtaining virtual eating gratification from mukbang watching could be a more serious risk factor for developing problematic mukbang watching and suffering unwanted negative consequences. |
| [30] | Kircaburun K, Balta S, Emirtekin E, Tosuntas, Demetrovics Z, Griffiths MD  (2021) | Compensatory usage of the internet: the case  of mukbang watching on youTube | United Kingdom | Journal paper: Cross-sectional questionnaire-based study, written in English | A total of 604 students ages ranged between 10 and 33 years for both sexes completed the survey. Of these, 217 were mukbang watchers (watched mukbang at least once before). | The aim of the present study was to examine the mediating role of problematic mukbang watching (PMW) on the relationships between depression and loneliness with problematic YouTube use (PYU). Results indicated that PMW was positively related to loneliness and PYU. Depression was positively and directly associated with PYU but was not associated with PMW, and that PMW would mediate the relationship between these psychosocial risk factors and PYU. It was also hypothesized that depressed and lonely people would engage in excessive mukbang watching via YouTube to alleviate negative feelings. |
| [31] | Alblas MC, Mollen S, Fransen ML, van den Putte B (2021) | See the cake and have it too? Investigating the effect of watching a  TV cooking show on unhealthy food choices | Netherlands | Journal paper: Intervention study, written in English | A total of 112 participants (90 females, 22 males) were joined in this study. The average age was 21 years old. Participants were exposed to a cooking segment (n = 50) or a non-food segment (n = 62) of a TV show. | This study used an interventional method to test the effects of the food-related (vs. non-food related) TV content on restrained (vs. unrestrained eaters) eaters. The results have showed that unsuccessful restrained eaters were particularly susceptible to the influences of watching food-related (vs. non-food related) TV content, and such exposure would for them result in more unhealthy food choices. In contrast, successful restraint eaters were expected to make less unhealthy food choices after watching food-related television content, due to the facilitative link between tempting food situations and automatic activation of their dieting goals. |
| [45] | Sultana SFS, Das P  (2022) | Content analysis of mukbang videos: preferences, attitudes and concerns | India | Journal paper: Qualitative study, written in English | The primary subject of this study is mukbang videos, a total of fifty videos have been analyzed; of the mukbangers, two are Indian (MaddyEats and Spice ASMR), two are South Korean (Hamzy and Tzuyang) and one is American (Zach Choi ASMR) in nationality. | This study clearly shows the preference of the type of content of the mukbang videos among the viewers and thus the more views garnered. Through the comment analysis, positive and negative attitudes and concerns were clearly captured. For the viewer perspective comments were categorized into five categories which included envy and amazement, body shaming, supportive, explanations and trend development whereas for the participant perspective, the comments were categorized into limits eating, increases eating, ambivalence, reduces loneliness, reduces guilt about own eating, and obsessive and selfdestructive categories. |
| [47] | Shin K (2021) | A Study on Food and Nutrition-Related Media Consumption and its Influence on Dietary Habits of Adolescents  and Adults in Daegu and Gyeongbuk Regio | Korea | Thesis: Cross-sectional study, written in Korean | The study surveyed a total of 341 adolescents and adults in the Daegu and Gyeongbuk regions. | This study conducted a cross-sectional survey to understand the food and nutrition-related media viewing and its impact on dietary habits of subjects in their 10s to 50s living in Korea. The study has found that there is excessive promotion of certain foods in the media, which may have a negative impact on people's food choices. Therefore, this paper suggests that there is a need for more scientific and careful regulation and management of media to improve public health and nutrition. |
| [48] | Xu W  (2019) | Does Watching Mukbangs Help You Diet? The Effect of the Mukbang on the Desire to Eat | China | Thesis: RCT, written in English | 1) Mukbang versus Non-Food Content Video (US): 114 women;  2) Effect of Different Food Stimuli (US): an online survey and recruited **286 workers** from Amazon Mechanical Turk;  3) Mukbang versus Cooking Show (China): 234 women. | This study included 3 experiments, two in-person and one online, to explore the effects of mukbang on appetite, calorie intake and diet. The study found that viewers invariably showed a strong desire for the food presented in the mukbang, except for the fruit. Conversely, however, mukbang can also encourage dieters with strong self-control to consume more healthy foods such as fruit. As a result, appetite, caloric intake, and diet are affected not only by mukbang watching, but also by the individual's control over food consumption. |
| [49] | Kang D (2022) | Some Elementary School Students in Ulsan Mukbang Viewing Status and Eating Behavior | Korea | Thesis: Cross-sectional study, written in Korean | This study was conducted among fifth to sixth grade students from five elementary schools in a city in Korea. 318 participants aged between 10~12 years old were joined in this study. | ​This study was designed to identify the current conditions of mukbang-watching among elementary school students and its impact on their negative eating habits and obesity. According to the results of this study, the higher the number of Mukbang-watching, the more greatly Mukbang had an effect on the viewers’ actual eating behaviors, such as getting the food shown in Mukbang. In addition, many of the food consumed by them after watching Mukbang was fast food and instant food, which shows that Mukbang-watching leads to the viewers’ intake of obesity-causing food. In addition, the students who often watched Mukbang had lower scores on the positive eating habits but higher scores on the negative habits, which demonstrated that frequent Mukbang watching is closely related to undesirable eating habits. |
| [50] | Yoon S (2017) | A Study on the Influence of Information Characteristics of TV Cooking Program on its Information Acceptance  and Dietary Change | Korea | Thesis: Cross-sectional study, written in Korean | The participants of this study were people who were interested in cooking and had watched food programs. A total of 220 people has joined this survey. | This is a study on the impact of the information characteristics of TV cooking programs on information reception and dietary changes. The study investigates the influence of TV cooking programs on viewers’ eating habits and provides very useful information. The result has shown the acceptance of information of TV cooking programs showed a positive significant influence of the TV cooking program on the change in dietary life. This study found that receiving food information on the radio had a positive impact on dietary changes. |
| [51] | Bang SY (2023) | A Study on the Eating Habits and Health Behavior of People in Their 20s and 30s According to the Use of Social  Media Food Content | Korea | Thesis: Cross-sectional study, written in Korean | The main subjects of this study were men and women in their 20s to 30s who used social media. Finally, a total of 452 participants completed the survey questionnaire. | The study was conducted to investigate the use of social media food content, eating habits, and health behaviors by dividing groups according to the use of social media food content. As a result, the study has found that the participants usually used social media food content to get information about restaurants and famous cafes, most of them might feel hungry or eat after using the food content. However, the study also showed that the use of food content through social media as well as mukbang showed a negative effect on eating habits. |
| [52] | Di Y  (2022) | A Study on the Motivation and Influence of Watching Network Mukbang Programs | China | Thesis: Cross-sectional questionnaire-based study, written in Chinese | In this study, mukbang audience were the research object, not gave the detailed inclusion or exclusion criteria. | The research purpose of this paper is based on the background of the hot development of "eating and broadcasting programs" and the normalization of weight loss, to systematically understand the viewing reasons, viewing behavior and attitude influence of the audience of eating and broadcasting programs, and the quantitative analysis of the impact of the audience watching the eating and broadcasting program, in response to the negative impact of eating and broadcasting considered in previous studies, according to the data analysis and survey results. This article finally believes that mukbang is not the root of all evil that changes the audience's eating behavior. It is more like a "reflector" of real problems, a carrier born with contradictions and needs. It also has the practical function of providing information and cultural output; providing emotional value to relieve stress and troubleshoot. |
| [53] | Kircaburun K, Yurdagül C, Kuss D, Emirtekin E, Griffiths MD (2021) | Problematic mukbang watching and its relationship to disordered eating and internet addiction: a pilot study among emerging adult mukbang watchers | United Kindom | Journal paper: Cross-sectional study, written in Korean | A total of 952 students began the survey and 312 of them completed it. Of these, 140 were mukbang viewers (66% female), aged between 19 and 29 years old. | This study mainly investigated the relationship of problematic mukbang watching with disordered eating and internet addiction, found that problematic mukbang watching was positively associated with both disordered eating and internet addiction. What’s more, the research has pointed out that problematic mukbang watching was more likely to be associated with more severe negative consequences of mukbang watching compared with recreational mukbang watching, problematic mukbang watching was also more likely to relate to increased real-life problematic eating practices (i.e., disordered eating). |
| [54] | Park S (2022) | A Study on the Use of YouTube Food Content and the Actual Consumption of Delivery Food by University Students  in Gwangju | Korea | Thesis: Cross-sectional study, written in Korean | The participants covered the characteristics of different ages, grades, majors, etc. | The main content of this paper is to study the eating habits of college students in Guangzhou, South Korea, including their awareness and use of take-away food, and an analysis of their satisfaction when watching YouTube food content. There was a positive correlation between the satisfaction of watching food content and health awareness, takeaway food awareness, and diet guide, respectively. |
| [55] | Yoo S, Shin G, Kim S (2021) | Does mukbang watching really affect obesity? : Focusing on the factors related to health and mukbang  watching | Korea | Journal paper: Cross-sectional study, written in Korean | A total of 668 people joined this survey. Of the respondents, 252 (37.7 percent) were obese and 416 (62.2 percent) were non-obese. | This study attempted to explore the degree of viewing and satisfaction with viewing, and whether the imitative eating behavior after viewing is the result of obesity, and its causal relationship.  The results showed that health awareness affected the degree of obesity, and the higher the health awareness, the lower the degree of obesity. Secondly, people with more general health information had lower obesity, and the more people sought information about obesity, the higher their obesity. Thirdly, the additional time and frequency of watching the broadcast, the higher the degree of obesity. |
| [56] | von Ash T, Huynh R, Deng C, White MA (2023) | Associations between mukbang viewing and disordered eating behaviors | America | Journal paper: Cross-sectional study, written in English | 264 participants who were at least 18 years of age, and had watched mukbang in the past 12 months were included in this study | The study deepened the understanding of associations between disordered eating and mukbang viewing by showing that additional characteristics of mukbang viewing (e.g., frequency of watching, average watch time, and eating while watching), beyond problematic mukbang viewing or mukbang addiction, may be associated with disordered eating. Eating disorder symptoms, especially binge eating and purging, were associated with greater problematic mukbang viewing and a tendency to not consume food while viewing mukbang. Participants with greater body dissatisfaction watched mukbang more frequently and were more likely to eat while watching mukbang, yet they scored lower on the Mukbang Addiction Scale and watched fewer average minutes of mukbang per viewing occasion. This study has found that mukbang viewing may encourage out-of-control/overeating by eliciting feelings of hunger among viewers or, through modeling, encourage consumption of hypercaloric foods that are often featured in mukbang videos. |
| [57] | Kang E, Lee J, Kim KH, Yun YH (2020) | The popularity of eating broadcast: content analysis of "mukbang" YouTube videos,  media coverage, and the health impact of "mukbang" on public | Korea | Journal paper: Content analysis (Qualitative study), written in English | YouTube videos, news articles, the items of a previous health tax study of 1200 Koreans | Similar to previous understanding that the creators of mukbang videos are predominantly eating unhealthy food or overeat, it was confirmed that numerous mukbang creators showed overeating, as well as other unhealthy eating behaviors. Mukbang videos have substantial content showing harmful eating habits, and watching mukbang is negatively related to healthy eating habits. |
| [58] | Li B  (2020) | Correlation analysis of watching motivation of mukbang and the influence of mukbang on audience | China | Journal paper: Cross-sectional study, written in Chinese | 84 people of all ages were investigated. | According to this study, people who like to listen to the sound of food chewing often enjoy ASMR Mukbang to help them sleep, and tend to watch high-calorie, high-fat food mukbang. When viewers watch mukbang to satisfy their own appetite, they will be interested in mukbang filled with high-calorie food. This kind of mukbang usually combines the sound of chewing and the visual effect of exaggerated large meals, which virtually amplifies the sense of impact and stimulation brought by mukbang and improves the decompression effect. |
| [59] | Zhang L, Cui L  (2020) | What satisfaction can you get from watching others eat?—A qualitative study based on the audience of  Bilibili | China | Journal paper: Semi-structured interview (cross-sectional study), written in Chinese | The participants were viewers who have seen the mukbang on the short video platform called Bilibili, and a total of 24 interviewees were recruited. | Through interviews, this paper tried to understand the satisfaction and psychology of viewers watching mukbang. It has been found that although some viewers watch mukbang without any purpose, most viewers watch mukbang for compensatory satisfaction, curiosity satisfaction, virtual companionship and pleasure. Viewers who chose to watch mukbang in order to achieve this satisfaction were likely to be facing some mental health issues in real life, and they watched mukbang to escape from problems. |
| [60] | Kawai N, Guo Z, Nakata R (2021) | Watching a remote-video confederate eating facilitates perceived taste and consumption of  food | Japan | Journal paper: Experimental study, written in English | 24 students who age ranged from 19 to 26 years (13 women) has participated in this experiment. | This study compared three types of silent videos: 1) a stranger eating potato chips, 2) the stranger calling on the phone, or 3) only objects (food and cellphone). Participants perceived popcorn to taste better only when they watched the video of others eating. Watching others eating induced the participants to eat more than when watching the other two videos. This study indicates that remote-video confederates enhance not only food intake but also the perceived taste of food.  In this study, the researchers have investigated whether watching a silent video of others eating amplified preferences for food and food intake compared to watching silent videos of others’ non-food related behavior or objects. |
| [61] | Ngqangashe Y, Backer CJSD  (2021) | The differential effects of viewing short-form online culinary videos of fruits and vegetables  versus sweet snacks on adolescents' appetites | Australia | Journal paper: Experimental study, written in English | Adolescents between the ages of 12 and 14 years sampled from mainstream local schools. | This study conducted a pre- and posttest study, aiming to investigate the effects of exposure to social media culinary videos on adolescents’ appetites. The findings showed that the videos had no effects on hunger or general desire to eat but influenced food choice behavior, liking of the foods, and intentions to eat and prepare the foods portrayed. The sweet snacks video reduced the liking of fruits and vegetables and indirectly reduced the odds of choosing a fruit over a cookie, through intentions to eat sweet snacks. The fruits and vegetables video reduced the liking of sweet snacks and resulted in higher intentions to prepare healthy snacks. |
| [62] | Ma M, Yang J  (2021) | The rise of digital table: a review of foreign researches on mukbang | China | Journal paper: Commentary, written in Chinese | Not applicable | This paper has analyzed the motives of mukbang viewers from the aspects of society, entertainment, diet et al. by sorting out the foreign literatures on mukbang. This study regarded mukbang as a "digital dining table" where people can watch it to reduce the space for social isolation; the entertainment satisfaction of mukbang was mainly reflected in helping the audience to obtain sensory satisfaction and fun by sharing the eating experience. Watching mukbang also promotes emotional and psychological connection with others. The negative effect of mukbang in this study was to change the audience's food preferences, eating habits and table manners, etc., leading to disordered eating. |
| [63] | Han C, Yin X  (2018) | Psychological exploration of "Mukbang" audience from the perspective of structuralism | China | Journal paper: Review, written in Chinese | Not applicable | This study believed that mukbang can play a visual decompression effect through its attractive food images, which can satisfy some of the audience's instinctive impulses. The conclusion of this paper pointed out that watching mukbang can make the audience gain a sense of pleasure, release the pressure and loneliness in real life. However, because of this, mukbang blocked them from further communication opportunities with the family and friends around them, damaged the social interaction and social ability in reality, what’s more, mukbang also had the risk of addiction. Therefore, mukbang is a double-edged sword for the audience, both helpful and potentially harmful. |
| [64] | Zhou J  (2023) | Research on the relieving effect of ASMR chewing sounds on anxiety in food video | China | Journal paper: Review, written in English | Not applicable | The main focus of this study is to examine the impact of ASMR mukbang on negative emotions through a comprehensive analysis. ASMR mukbang mainly makes people's scalp, back and other parts feel numb through the visual presentation of pictures and the auditory presentation of sounds. This kind of mukbang does not put the visual presentation of food in the dominant position of communication, but infinitely magnifies the auditory elements, thus stimulating people's cognitive response. Based on the study results, the spread of sound in ASMR mukbang can relax the brain to make people fall asleep more easily, and relieve people's stress and anxiety in real life to some extent. |
| [65] | Ngqangashe Y, Maldoy K, De BC, Vandebosch H  (2022) | Exploring adolescents' motives for food media consumption using the theory of uses and gratifications | Australia | Journal paper: Semi-structured interview (cross-sectional study), written in English | 31 middle-school adolescents (14 male, 17 female) aged between 12 and 16. | This study seeks to explore food media use among adolescents and incidental consumption in the context of food media, also approaches selective consumption of food media from a uses and gratifications perspective. The food media explored in this study entail traditional food media in the form of TV cooking shows and new media in the form of YouTube cooking channels, social media culinary videos, social media food posts and online recipes. According to the conclusions of this study, occasional exposure to food media can also promote the health of the exposed person, but this must be based on the content of the food media itself is healthy. |
| [66] | Kim J, Choi S, Kim H, An S  (2021) | Binge drinking and obesity-related eating: the moderating roles of the eating broadcast viewing experience among Korean adults | Korea | Journal paper: Cross-sectional study, written in Korean | A total of 1125 people were sampled to represent the Korean people by allocating people living in 17 metropolitan and provincial governments by sex, age group, and region. | The goal of this study was to examine whether eating broadcast viewing experiences affected the association between the frequency of binge drinking and obesity-related eating behaviors among Korean adults. The study has found that mukbang can be identified as an environmental factor that influences both binge drinking and obesity-related eating behaviors. |
| [67] | Jo S, Choi H (2021) | A study on the cookbang YouTube program use and dietary change based on the technology acceptance model | Korea | Journal paper: Cross-sectional study, written in Korean | The research participants in this study were 214 Korean adults who had experience using the Cookbang YouTube program. | Through a survey and empirical analysis of consumers who watch Cookbang YouTube programs, the study found that perceived enjoyment and perceived usefulness have a significant positive impact on the intention to continue using and changes in eating habits. In addition, intention to continue use also had a significant positive impact on changes in dietary habits. In other words, people who enjoy and find Cookbang YouTube programs useful are more likely to make changes to their dietary habits. |
| [68] | Lee D (2019) | A Research on the Causal Factor to Binge Eating That Affects Dieting Deriving From One Person Media Viewership—Focused on Females Between the Ages of 20-30 | Korea | Thesis: Cross-sectional study, written in Korean | A total of 175 women in their 20s and 30s were sampled for this study. | The main purposes of this study are to discover the effect of viewing time and frequency of single-person media mukbang on binge eating. The study analyzed the information characteristics provided by these broadcast cooking programs, and the effects of mukbang on the changes in dietary habits were discussed. |
| [69] | Lee S, Lee SH  (2022) | Actual status of mukbang viewing and food habits of university students in Wonju area | Korea | Journal paper: Cross-sectional study, written in Korean | The subjects of this study were current students at a university located in a city in Korea, 354 participants finally finished this survey. | The purpose of this study was to investigate the viewing status of the online audiovisual food-related broadcast Mukbang by university students in Wonju (Gangwon, Republic of Korea). The association between the viewing degree of Mukbang and students’ eating habits was also evaluated. The results of this study demonstrate the impact of mukbang on a suboptimal diet life. Moreover, watching mukbang was likely to form undesirable consumption patterns and poor eating habits, which may have negative effects on obesity and other factors. |
| [70] | Alblas MC (2021) | Consuming media, consuming food? Reactivity to palatable food cues in television content | Netherlands | Thesis:  Experimental study, written in Korean | A total of 800 adults (400 men and 400 women) in their 20s and 30s participated in this study. | This study found that the influence of palatable food cues on TV is less evident than is often thought, and that reactivity to exposure to food on TV may be highly dependent on a range of factors relating to individual differences, the type of content, and other factors as well. With the current knowledge there is no compelling evidence for the harmful effects of exposure to food cues in TV content on food intake, nor on processes that are thought to result in such behavior. This does not mean that the frequent exposure to food on TV may not contribute to increased food intake at all. However, other influences are likely better able to explain the association between TV viewing and increased food intake, such as distraction (i.e., paying attention to the TV content may reduce awareness of what and how much is eaten, which may result in overconsumption), habits (e.g., learned associations between TV viewing as leisure-time activity and eating snacks) and/or a positive mood induced by TV viewing. It is therefore recommended to look beyond the influence of food cues in order to reduce overconsumption, and subsequent obesity, resulting from TV viewing. |
| [71] | Kim SY (2020) | The Effect of Mukbang Show and Induced Anxiety on Eating Behavior | Korea | Thesis: Intervention study, written in Korean | A total of 43 college students and graduate students attending a university were randomly assigned to each experimental condition. | This study examines the impact of watching mukbang and inducing anxiety on eating behavior. The study did not find the relationship between mukbang watching and appetite or food intake. However, it was confirmed that negative emotions reduced appetite and intake. What’s more, appetite and intake were high after watching mukbang when in normal emotion, which suggests that appetite and intake may be increased following exposure to mukbang as an external cue. In both the regular video group and the mukbang group, negative emotion could lead to a decrease, not an increase, in appetite and intake. However, according to this study, as an external environmental stimulus, mukbang video did not alleviate the decreased appetite and intake due to negative emotions. |
| [72] | Li Y (2020) | Get out of the emotional hunger—psychological and behavioral analysis of the popularity of mukbang | China | Magazine article: Commentary, written in Chinese | Not applicable | This article analyzed the reasons for the popularity of mukbang in China, such as activating viewers’ gustation, obtaining the experience of alternative satisfaction and intimating interaction. Meanwhile, this article also pointed out that the harmful information in the popular mukbang video in China, such as fake eating and vomiting, not only wasted food but also cost one’s health. What’s more, this harmful information also invisibly affected the mukbang viewers, destroyed their original eating habits and health of the audience over time. |
| [73] | Pan M (2020) | An analysis of mukbang watching and watching behaviors of restricted eating groups: based on vicarious gratification theory | China | Journal paper: Commentary, written in Chinese | Not applicable | According to this study, mukbang may help weight loss, but relying too much on mukbang for losing weight can lead to the wrong behaviors, which can harm the viewers’ health. People who take an unhealthy approach to weight control and diet restriction would rely more on mukbang for vicarious satisfaction, which is actually an unhealthy way to use mukbang and is more likely to lead to emotional eating and overeating due to the gap between illusion and reality. |
| [74] | Wei J. (2018) | The reflect on the negative impact of "mukbang fever" on teenagers | China | Magazine article: Commentary, written in Chinese | Not applicable | Mukbang can distort adolescents’ concept of health. In adult mukbang, most of the anchors have slim bodies and beautiful faces, but they can "tolerate" huge food with their slim bodies. The strong contrast between body and appetite attracts the envy of teenagers. Adult anchors convey the signal that they can eat and not be fat to teenagers, which attracts their blind worship and follow the trend. This can impair teenagers' eating behaviors and habits, leading to overeating. |
| [75] | Gao Z (2021) | The impacts and countermeasure of the novelty seeking live videos chaos on teenagers | China | Magazine article: Commentary, written in Chinese | Strong flavor mukbang and Big Eater mukbang videos and adolescents | By analyzing the characteristics and popular status of Big Eater Mukbang and Heavy Taste mukbang videos, this paper focused on the impact of this type of webcast on adolescents' health views and values. The study found that this type of mukbang mainly promoted binge eating, waste of food, abnormal psychology and wanton consumption, which had a negative impact on the correct formation of adolescents' health views and values, continuously stimulated adolescents to binge eating without restraint, seriously harmed the health of adolescents, and led to the distortion of adolescents' health views and values. |
| [76] | Nam NH (2020) | A Study on the Prevalence of Watching Mukbang and Factors Related Food Behaviors in Adults | Korea | Thesis: Cross-sectional study, written in Korean | The participants of this study were adults of all genders with mukbang viewing experience. A total of 800 participants who have watched mukbang videos have joined in the study. | This study was to investigate the relationship between the reality of watching 'Mukbang' and eating habits. The study has found that mukbang watching was associated with worse dietary choices and eating habits. People who watched less mukbang videos paid more attention to food nutrition and food health. According to the results of these studies, there was a clear positive correlation between 'Mukbang' viewing time and body mass index (BMI). The higher the viewing time, the higher the intake of late-night snacks and delivered food, and undesirable health behaviors and eating behaviors, whereas the frequency of breakfast was lower. |
| [77] | Kircaburun K, Harris A, Calado F, Griffiths MD (2021) | The psychology of mukbang watching: a scoping review of the academic and non-academic literature | United Kindom | Journal paper: Scoping Review, written in English | Not applicable | Results from the scoping review indicated that viewers use mukbang watching for social reasons, sexual reasons, entertainment, eating reasons, and/or as an escapist compensatory strategy. Furthermore, mukbang watching appears to have both beneficial consequences (e.g., diminishing feelings of loneliness and social isolation, constructing a virtual social community,) and non-beneficial consequences (e.g., altering food preferences, eating habits, and table manners, promoting disordered eating, potential excess, and ‘addiction’). |
| [78] | Pereira-Castro MR, Pinto AG, Caixeta TR, Monteiro RA, Bermúdez XPD, Mendonça AVM (2022) | Digital forms of commensality in the 21st century: a scoping review | Brazil | Journal paper: Scoping review, written in English | Not applicable | The advantages of the intersections between the digital world and food, such as reducing loneliness and improving eating habits, and the disadvantages, such as encouraging excessive eating and maintaining behaviors associated with eating disorders. The Mukbang, described throughout the sample as the excessive consumption of ultra-processed foods by its presenters and the regulation of food consumption by its viewers, was occasionally deleterious by increasing the intake of foods high in salt, fat, sugars, and food additives and sometimes had a positive effect by including lonely individuals in communities. |
| [79] | Fangxiu L (2018) | Research on audience psychology and profit model of mukbang from the perspective of communication | China | Magazine article: Commentary, written in Chinese | Not applicable | This paper took mukbang as the main research object and summarized the psychology of viewers who love mukbang through the collection of relevant video materials. It has found that viewers watch mukbang mainly to help them release loneliness, get a sense of pleasure, relieve pressure and manage their body. In addition, mukbang also combined all the characteristics of audiovisual media to stimulate the audience's appetite through visual and auditory stimulation, which plays a role in promoting appetite. |
| [80] | Tian R. (2020) | Psychological research of mukbang audience from the perspective of use and satisfaction theory | China | Journal paper: Commentary, written in Chinese | Not applicable | In this study, mukbang video is narrowly defined as Big Eater Mukbang. The anchor in the video conveys bad eating habits to the audience through exaggerated eating style, one-time intake of a large amount of heavy flavor or unhealthy food, and fake eating and vomiting. The content related to health factors in this paper mainly relates to mental health, and viewers watching mukbang can help them relieve pressure and fatigue in life. At the same time, for people who need to maintain a strict diet, mukbang can help them avoid eating high-calorie foods by substituting sexual satisfaction, so as to maintain a healthy body. |
| [81] | Zhong X (2021) | The study of ASMR mukbang on network platforms | China | Magazine article: Commentary, written in Chinese | Not applicable | Based on the carnival theory, this paper conducted an in-depth analysis of the popularity of ASMR Mukbang, and found that viewers choose to watch ASMR Mukbang because it could provide a sense of virtual companion and relieve their stress and anxiety. In addition to this, viewers can obtain a sense of substitutional satisfaction and indirectly satisfy their appetite by feeling the auditory stimuli that are deliberately amplified in the ASMR Mukbang video, such as chewing and swallowing sounds. |
| [82] | Wu M (2019) | The reason of "Mukbang Heat" phenomenon from the perspective of communication science | China | Magazine article: Commentary, written in Chinese | Not applicable | ​The contents of the mukbang tend to show the slenderness of the anchors and the astonishing amount of food they eat. The videos of the anchors eating extreme foods have spread unhealthy eating behaviors to the public. Studies have found that watching mukbang relieves stress and loneliness, soothes the mind and seeks solace and happiness. At the same time, this article also emphasized that the positive effect on psychology was based on the viewer's full respect for their body in real life. If the viewer blindly imitated the anchor's eating behaviors, it could only erase the function and significance of the mukbang in healing negative emotions and relieving pressure. |
| [83] | An S, Lim Y, Lee H (2020) | A study of viewers' comments on online mukbang videos: a big-data analysis of perceptions toward eating behavior | Korea | Journal paper: Qualitative study, written in Korean | A total of 72,721 viewer comments on 36 popular YouTube Mukbang videos uploaded between July 2018 and June 2019 were collected and analyzed. | The purpose of this study was to examine viewers' comments on how popular online eating show (Mukbang) videos influence viewers' perceptions and lead to unhealthy eating behaviors. This study found that viewer comments showed an overall positive response regarding Mukbang videos' influence on eating behavior and appetite. The viewer comments were negative as well as positive on both Mukbang videos including unhealthy eating behavior and food. It could be summarized as that Mukbang content including healthy eating behavior is more popular with viewers. |
